# Genome-Wide Characterization of Major Intrinsic Proteins in Four Grass Plants and Their Non-Aqua Transport Selectivity Profiles with Comparative Perspective

**DOI:** 10.1371/journal.pone.0157735

**Published:** 2016-06-21

**Authors:** Abul Kalam Azad, Jahed Ahmed, Md. Asraful Alum, Md. Mahbub Hasan, Takahiro Ishikawa, Yoshihiro Sawa, Maki Katsuhara

**Affiliations:** 1 Department of Genetic Engineering and Biotechnology, Shahjalal University of Science and Technology, Sylhet 3114, Bangladesh; 2 Forensic DNA Laboratory of Bangladesh Police, Malibagh, Dhaka, Bangladesh; 3 Department of Genetic Engineering and Biotechnology, University of Chittagong, Chittagong 4331, Bangladesh; 4 Department of Life Science and Biotechnology, Shimane University, Shimane 690–8504, Japan; 5 Institute of Plant Science and Resources, Okayama University, Chuo-2-chome, Kurashiki 710–0046, Japan; University of Minho, PORTUGAL

## Abstract

Major intrinsic proteins (MIPs), commonly known as aquaporins, transport not only water in plants but also other substrates of physiological significance and heavy metals. In most of the higher plants, MIPs are divided into five subfamilies (PIPs, TIPs, NIPs, SIPs and XIPs). Herein, we identified 68, 42, 38 and 28 full-length MIPs, respectively in the genomes of four monocot grass plants, specifically *Panicum virgatum*, *Setaria italica*, *Sorghum bicolor* and *Brachypodium distachyon*. Phylogenetic analysis showed that the grass plants had only four MIP subfamilies including PIPs, TIPs, NIPs and SIPs without XIPs. Based on structural analysis of the homology models and comparing the primary selectivity-related motifs [two NPA regions, aromatic/arginine (ar/R) selectivity filter and Froger's positions (FPs)] of all plant MIPs that have been experimentally proven to transport non-aqua substrates, we predicted the transport profiles of all MIPs in the four grass plants and also in eight other plants. Groups of MIP subfamilies based on ar/R selectivity filter and FPs were linked to the non-aqua transport profiles. We further deciphered the substrate selectivity profiles of the MIPs in the four grass plants and compared them with their counterparts in rice, maize, soybean, poplar, cotton, *Arabidopsis thaliana*, *Physcomitrella patens* and *Selaginella moellendorffii*. In addition to two NPA regions, ar/R filter and FPs, certain residues, especially in loops B and C, contribute to the functional distinctiveness of MIP groups. Expression analysis of transcripts in different organs indicated that non-aqua transport was related to expression of MIPs since most of the unexpressed MIPs were not predicted to facilitate the transport of non-aqua molecules. Among all *MIP*s in every plant, *TIP* (*BdTIP1;1*, *SiTIP1;2*, *SbTIP2;1* and *PvTIP1;2*) had the overall highest mean expression. Our study generates significant information for understanding the diversity, evolution, non-aqua transport profiles and insight into comparative transport selectivity of plant MIPs, and provides tools for the development of transgenic plants.

## Introduction

Aquaporins (AQPs), water channel proteins, are channel-forming integral membrane proteins that are found in all living organisms [1,2]. Plant AQPs are involved in many physiological processes such as motor cell movement, root and leaf hydraulic conductance, diurnal regulation of leaf movements, rapid internode elongation, responses to numerous abiotic stresses, temperature-dependent petal movement and petal development [1,3,4,5,6,7,8,9].

AQPs belong to the ancient major intrinsic proteins (MIPs) super family. Although 13 different AQPs have been identified in mammals [10], the genomes of plants encode 2–5 folds more AQP homologues [11,12,13,14,15,16,17,18,19]. On the basis of sequence homology and cellular localization, plant AQPs are classified into four subfamilies: (1) plasma membrane intrinsic proteins (PIPs), which are usually localized in the plasma membrane (PM); (2) tonoplast intrinsic proteins (TIPs), which are generally localized in the vacuolar membranes; (3) nodulin-26-like intrinsic proteins (NIPs); and (4) small basic intrinsic proteins (SIPs) [2]. Recently, a fifth subfamily of uncharacterised X intrinsic proteins (XIPs) [20] has been reported in the PM [21].

Plant AQPs have been reported recently to transport not only water but also a wide range of substrates such as ammonia, antimony, arsenite, boron, carbon dioxide, glycerol, hydrogen peroxide, silicon, urea etc [2,22,23,24,25]. Almost all of these molecules are important for plant growth and development, plant nutrition, photosynthesis, structures of biological membranes and cell walls, tolerance to biotic and abiotic stresses, stomatal movement and senescence [26,27,28,29]. These physiological roles as well as the chance of heavy metalloids such as arsenic and antimony to enter into the food chain through plant AQPs suggest that it is important to understand their transport selectivity profiles. Despite the discovery of more than 400 AQPs in plants, very few studies have been done to compare their transport profiles and the molecular determinants for the substrate selectivity.

AQPs consist of six transmembrane (TM) α-helices (helix H1–H6) and five loops (loops A–E). The N- and C-termini are located on the cytoplasmic side of the membrane. In the pore of the channel, two regions of constriction have been proposed to specify the transport selectivity profile. The first constriction is formed at the centre of the pore by oppositely juxtaposing two Asn-Pro-Ala (NPA) motifs in loops B and E [30]. This constriction is supposed to be involved in proton exclusion [31]. Consensus sequences are suggested for the first (SGXHXNPAVT) [32] and second (GXXXNPAR(S/D)XG) [33] NPA motifs. The second constriction known as the aromatic/arginine (ar/R) selectivity filter is formed at the extracellular mouth of the pore by four residues from H2, H5, and loop E (LE1 and LE2), respectively [34,35]. Variability at the ar/R selectivity filter is thought to form the basis of the broad spectrum of substrate conductance in plant AQPs [11,14,36,37]. Up to five relatively conserved amino acid residues known as the Froger’s positions (FPs) and those designated P1-P5 play roles in substrate selectivity [32,38]. Recently, some specificity-determining positions have been suggested by analyzing the protein sequences of MIPs transporting non-aqua substrates in wet-lab experiments [23].

Identification and characterization of the *MIP* gene family is the first step in investigating the role of MIPs in plant water relationships or transporting physiologically important small molecules. Grasses, plants of the Poaceae family, the largest plant family in the world, afford the bulk of human nutrition, and highly productive grasses are potential sources of sustainable biofuels [39,40]. Phytozome (www.phytozome.net), which facilitates comparative genomic studies among green plants, provides access to six grass plants. The MIPs in rice and maize, among these six grass plants, have been reported [12,14,17]. There has been no study for MIPs in the remaining four grass plants namely switchgrass (*Panicum virgatum*), foxtail millet (*Setaria italica*), sorghum (*Sorghum bicolor*) and *Brachypodium distachyon*. *P*. *virgatum*, which exists at multiple ploidies, is a drought tolerant plant and has been intensively studied as a source of lignocellulosic biomass to produce renewable energy [41,42]. *S*. *italica* is closely related to *P*. *virgatum*. It is a small diploid C4 panicoid crop species and a more tractable experimental model because of its small genome [43]. *S*. *bicolor*, related to sugar cane and maize, is grown for food, feed, fibre and biofuels [44]. *B*. *distachyon*, related to rice, maize, wheat, barley, sorghum and millet, has several advantages as an experimental model organism for understanding genetic, cellular and molecular biology of temperate grasses [40].

In the study reported herein, we identified *MIP* genes in the genomes of *P*. *virgatum*, *S*. *italica*, *S*. *bicolor* and *B*. *distachyon*. We investigated the phylogeny, structural properties, *in silico* subcellular localization and expression profiles of MIPs in these plants. Based on structural analysis of the homology models and comparing the primary selectivity-related motifs, we further deciphered the non-aqua transport profiles (ammonia, antimony, arsenic, boron, CO_2_, H_2_O_2_, silicon and urea) and molecular determinants for substrate selectivity of the MIPs in the four grass plants and compared them with their counterparts in two grass plants such as rice (OsMIP) and maize (ZmMIP) and six non-grass plants such as soybean (GmMIP), poplar (PtMIP), cotton (GhMIP), *Arabidopsis thaliana* (AtMIP), *Selaginella moellendorffii* (SmMIP) and *Physcomitrella patens* (PpMIP).

## Materials and Methods

### Identification of *PvMIP*, *SiMIP*, *SbMIP* and *BdMIP* genes

The genomes of *P*. *virgatum* (JGI v1.1), *S*. *italica* (JGI v2.1), *S*. *bicolor* (v2.1) and *B*. *distachyon* (v1.2), available at Phytozome, were searched for MIPs using TBLASTN and BLASTp tools with the protein sequences of the complete set of 55 MIPs from *P*. *trichocarpa* and 22 MIPs from *P*. *patens* as queries. PvMIPs, SiMIPs, SbMIPs and BdMIPs were included until no more MIPs could be found from *P*. *virgatum*, *S*. *italica*, *S*. *bicolor* and *B*. *distachyon*, respectively. Every sequence from each plant was individually compared with functional annotations by browsing the Phytozome databases of *P*. *virgatum*, *S*. *italica*, *S*. *bicolor* and *B*. *distachyon* to indentify the maximum number of MIPs for further analyses. The genomic regions containing *MIP* genes were further used to determine the gene structure using the program GeneMark.hmm ES-3.0 [45] (http://exon.gatech.edu/GeneMark), a self-training based algorithm for prediction of genes from novel eukaryotic genomes, and *Arabidopsis* was chosen as a model organism in GeneMark for gene prediction in *P*. *virgatum*, *S*. *italica*, *S*. *bicolor* and *B*. *distachyon*. When short genes were found, their sequences with 1000 base flanking regions were subjected to Genetyx_SV_RC_version 7 to investigate their protein sequences.

### Phylogenetic and domain analysis of PvMIPs, SiMIPs, SbMIPs and BdMIPs

PvMIPs, SiMIPs, SbMIPs or BdMIPs were separately aligned with PtMIPs using the Clustal Omega program (http://www.ebi.ac.uk/Tools/msa/clustalo/) and a phylogenetic tree was constructed using Molecular Evolution Genetic Analysis (MEGA), version 5.0 [46]. The evolutionary history was inferred using the Neighbor-Joining method and the genetic distance was estimated by the p-distance method. To identify the total number of subfamilies present in PvMIPs, SiMIPs, SbMIPs and BdMIPs, phylogenetic analysis was also conducted with PpMIPs that have seven subfamilies [20], whereas PtMIPs have five subfamilies. The identified PvMIPs, SiMIPs, SbMIPs and BdMIPs were classified into different subfamilies and groups by their phylogenetic relationship with PtMIPs. To investigate the different subfamilies and groups, we further analyzed phylogeny separately with AtMIPs, ZmMIPs, OsMIPs and GmMIPs. PvMIPs, SiMIPs SbMIPs and BdMIPs were named according to the best similarities from the trees generated by phylogeny analysis. To construct the phylogenetic tree with the MIPs in the four grass plants, all of their MIPs were aligned as above. The TM α-helices were predicted by SOSUI (http://bp.nuap.nagoya-u.ac.jp/sosui/), TMpred (http://www.ch.embnet.org/software/TMPRED_form.html) and the tools of ExPASy (http://kr.expasy.org/tools/).

### Homology modeling

Homology models were constructed using the Molecular Operating Environment software (MOE 2009.10; Chemical Computing Group, Quebec, Canada). The sequence of each MIP homologue was aligned with the open conformation of spinach PIP, SoPIP2;1 (PDB, Protein Data Bank ID: 2B5F) [47] using the MOE software as described previously [36]. The alignment of the MIP homologue was based on both sequence and structural homology with the structure of SoPIP2;1. The 3D structure models were formed using the MOE homology program and the stereochemical quality of the templates and the models was assessed, as we described previously [36].

### Prediction of subcellular localization and computation of Ka/Ks value

The subcellular localizations of PvMIPs, SiMIPs, SbMIPs and BdMIPs were predicted *in silico* by using tools of WoLF PSORT (http://www.genscript.com/wolf-psort.html), TargetP (www.cbs.dtu.dk/Services/TargetP), Cello prediction system (http://cello.life.nctu.edu.tw/) and MultiLoc2 (www.abi.inf.uni-tuebingen.de/Services/MultiLoc2). Ka and Ks are the numbers of non-synonymous and synonymous substitutions per site, respectively on a protein-coding gene. The Ka/Ks values of the *PvMIPs*, *SiMIPs*, *SbMIPs and BdMIPs* were calculated using an online Ka/Ks calculation tool at http://services.cbu.uib.no/tools/kaks. A Ka/Ks value greater than one implies gene evolution under positive or Darwinian selection; less than one indicates purifying (stabilizing) selection and a Ka/Ks value of one suggests a lack of selection or possibly a combination of positive and purifying selections at different points within the gene that cancel each other out [18].

### Expression analysis

For expression analysis, a compendium of RNA-seq data for the plants in the Phytozome was used. In the Phytozome, *P*. *virgutam*, *S*. *bicolor* and *B*. *distachyon* were selected separately and the phytozome accession number of a specific *MIP* was entered to search the gene. Transcript level as FPKM (Fragments per Kilobase of Transcript per Million Mapped Reads) values of a *MIP* gene was achieved from the gene view link. The FPKM values for each *MIP* gene of *S*. *italica* was retrieved from the *InterMine* interface of Phytozome (https://phytozome.jgi.doe.gov/phytomine/template.do?name=One_Gene_Expression&scope=global) using phytozome accession number or identifier. The FPKM values of individual *MIP* gene in leaf, root and shoot under diverse conditions were retrieved and put into the Microsoft Excel. The heatmap was generated using conditional formatting based on the FPKM values. The FPKM values <1 were treated as no expression of the respective gene.

### Determination of pore diameter and pore lining residues

To analyze the MIP channels, the poreWalker server [48] (http://www.ebi.ac.uk/thornton-srv/software/PoreWalker/) was used. This is a fully automated method designed to detect and characterize transmembrane protein channels from their 3D structures. The 3D structure of a MIP in PDB format was uploaded to the server, which generated the specific pore characteristics, particularly the conformation and the regularity of the channel cavity, the corresponding pore lining residues and atoms, and the location of pore centers along the channel. From the PoreWalker outputs, the pore diameter profiles at different regions of a MIP channel were compiled. From the given pore diameter profile of a channel, continuous numerical data were constructed from the non-continuous numerical data through a customized statistical language R-script so that the precise pore diameter at a specific region particularly at the ar/R selectivity filter could be determined. The existing values of pore diameters generated by the PoreWalker were used as an input in the R-script to calculate the missing values of pore diameters to make a continuous pore diameter profile. Through the PoreWalker server, the pore lining residues, which are very important for the formation of a channel, were identified.

## Results

### Genome-wide identification of *PvMIP*, *SiMIP*, *SbMIP* and *BdMIP* genes

The whole genome shotgun sequence (WGS) of *P*. *virgatum*, *S*. *italica*, *S*. *bicolor* and *B*. *distachyon* available at Phytozome was searched for *PvMIP*, *SiMIP*, *SbMIP* and *BdMIP* genes using TBLASTN. The query PtMIP and PpMIP sequences from *P*. *trichocarpa* and *P*. *patens* resulted in 116, 51, 44 and 37 hits for *PvMIPs*, *SiMIPs*, *SbMIPs and BdMIPs*, respectively. We further analyzed the PvMIP, SiMIP, SbMIP and BdMIP sequences for domain identification. Out of 116 unique hits for *PvMIPs*, 48 were deemed to be pseudo *MIP* genes after manual inspection of their amino acid sequences, TM domains and homology models, and were discarded (S1 Table). Out of the 51 unique hits for *SiMIPs*, 9 were deemed to be pseudo *MIP* genes and were discarded (S1 Table). On the other hand, 6 and 9 unique hits for *SbMIPs* and *BdMIPs*, respectively were deemed to be pseudo *MIP* genes and discarded (S1 Table). We ultimately obtained 68, 42, 38 and 28 full-length PvMIP, SiMIP, SbMIP and BdMIP protein sequences from the WGS of *P*. *virgatum*, *S*. *italica*, *S*. *bicolor* and *B*. *distachyon*, respectively (Tables 1–4).

**Table 1:** *MIP* genes in *P*. *Virgatum*.

Gene Name	Phytozome accessions	Genomic Location	PPL(aa)	Maximum Identity with other *MIP* (%)^x^	PSCL^y^	Ka/Ks value
***PvPIP1;1***	Pavir.Gb01084.2	Chr07b: 13931659–13933701	288	XP_002454508(98)^a^	PLAS, CHLO	0.095
***PvPIP1;2***	Pavir.J11645.1	contig141014: 535–2653	288	AAO86706(97)^b^	PLAS, CHLO	0.380
***PvPIP1;3***	Pavir.Gb01084.3	Chr07b: 13931659–13933701	277	AAO86706(97)^b^	PLAS	0.669
***PvPIP1;4***	Pavir.Aa00868.1	Chr01a: 10299092–10302435	289	XP_004953388(99)^c^	PLAS, CHLO	0
***PvPIP1;5***	Pavir.J37677.1	contig69730: 133–3636	289	XP_004953388(99)^c^	PLAS, CHLO	0.116
***PvPIP1;6***	Pavir.Aa00075.1	Chr01a: 810607–812141	288	NP_001105131(98)^b^	PLAS, CHLO	0.052
***PvPIP1;7***	Pavir.Ab03380.1	Chr01b: 55703303–55704564	288	NP_001105131(99)^b^	PLAS, CHLO	0.023
***PvPIP2;1***	Pavir.Ab02356.1	Chr01b: 44317427–44320839	288	NP_001105026(98)^b^	PLAS, CHLO	0.307
***PvPIP2;2***	Pavir.Ab02356.2	Chr01b: 44317427–44320300	264	ACG33001(98)^b^	PLAS	0.569
***PvPIP2;3***	Pavir.Bb01320.1	Chr02b: 27409376–27413184	363	NP_001105024(98)^b^	PLAS	0.103
***PvPIP2;4***	Pavir.Ga01149.1	Chr07a: 14124713–14126981	266	XP_004976254(96)^c^	PLAS	0.463
***PvPIP2;5***	Pavir.Gb00671.1	Chr07b: 7857237–7859623	277	XP_004976254(99)^c^	PLAS	0.505
***PvPIP2;6***	Pavir.Ba02483.2	Chr02a: 37691323–37694961	290	XP_002461930(99)^a^	PLAS, CHLO	0.186
***PvPIP2;7***	Pavir.Bb01320.2	Chr02b: 27409376–27413188	290	XP_002461930(99)^c^	PLAS, CHLO	0.688
***PvPIP2;8***	Pavir.Bb01841.1	Chr02b: 46595867–46597386	286	XP_004956116(97)^c^	PLAS, CHLO	0.331
***PvPIP2;9***	Pavir.Ba02478.1	Chr02a: 37576388–37578287	286	XP_004956116(98)^c^	PLAS, CHLO	0.201
***PvPIP2;10***	Pavir.Ib04237.1	Chr09b: 67322496–67323750	276	XP_002489214(90)^a^	PLAS	0.111
***PvPIP2;11***	Pavir.Ia02751.1	Chr09a: 54199846–54200694	282	XP_002489214(89)^a^	PLAS	0.238
***PvPIP2;12***	Pavir.Ib03181.1	Chr09b: 51605207–51606895	294	XP_004986496(84)^c^	PLAS	0.476
***PvPIP2;13***	Pavir.Ba01199.1	Chr02a: 15220158–15221702	284	XP_004957505(85)^c^	PLAS	1.323
***PvPIP2;14***	Pavir.J11644.1	contig140997: 365–1545	287	XP_004957505(83)^c^	PLAS	0.327
***PvTIP1;1***	Pavir.Ia04869.1	Chr09a: 86386535–86388928	250	P50156(96)^d^	VACU	0
***PvTIP1;2***	Pavir.Ib00275.1	Chr09b: 2982020–2984273	250	P50156(96)^d^	PLAS	0.267
***PvTIP1;3***	Pavir.Ea04152.1	Chr05a: 63625246–63626300	252	XP_004971442(92)^c^	VACU	0
***PvTIP1;4***	Pavir.Ea04152.2	Chr05a: 63625246–63626533	252	XP_004971442(91)^c^	PLAS	0
***PvTIP2;1***	Pavir.Gb01125.1	Chr07b: 14244344–14245618	248	XP_004976439(98)^c^	PLAS	0.035
***PvTIP2;2***	Pavir.Ga01087.1	Chr07a: 12722976–12724266	248	XP_004976439(98)^c^	PLAS	0.076
***PvTIP2;3***	Pavir.J30578.1	contig357494: 1–1165	249	XP_004953349(98)^c^	PLAS	0.203
***PvTIP2;4***	Pavir.Da01714.1	Chr04a: 37554270–37555738	248	XP_002438430(97)^a^	PLAS	0.212
***PvTIP2;5***	Pavir.Db01217.1	Chr04b: 23834192–23835703	248	XP_002438430(97)^a^	PLAS	0.063
***PvTIP3;1***	Pavir.Ia01749.1	Chr09a: 21354884–21356273	263	NP_001105032(95)^b^	MITO	0.271
***PvTIP3;2***	Pavir.Ib03520.1	Chr09b: 57398634–57399928	264	NP_001105032(95)^b^	MITO	0.033
***PvTIP3;3***	Pavir.Ga00845.1	Chr07a: 10052517–10054500	273	XP_002446824(88)^a^	CHLO	0.456
***PvTIP4;1***	Pavir.Ea00003.1	Chr05a: 159662–160973	250	XP_004967395(94)^c^	VACU	0.682
***PvTIP4;2***	Pavir.J30482.1	contig355910: 206–1208	256	XP_004967395(91)^c^	CYTO	0.102
***PvTIP4;3***	Pavir.J20433.1	contig222165: 1187–2025	239	XP_004967395(88)^c^	VACU	0.819
***PvTIP4;4***	Pavir.Eb00023.1	Chr05b: 514689–516054	259	XP_004967394(92)^c^	CYTO	0.521
***PvTIP4;5***	Pavir.Cb01832.1	Chr03b: 43764796–43767586	347	XP_004960662(93)^c^	CHLO	0.154
***PvTIP4;6***	Pavir.Ca00461.1	Chr03a: 5397927–5400491	318	XP_004960662(91)^c^	CYTO	0.172
***PvTIP5;1***	Pavir.Gb01126.1	Chr07b: 14245888–14247100	270	XP_004978166(82)^c^	CHLO	0.187
***PvTIP5;2***	Pavir.Ga01088.1	Chr07a: 12724501–12725804	266	XP_004978166(78)^c^	CHLO	0.522
***PvNIP1;1***	Pavir.Cb01700.1	Chr03b: 42769884–42772044	280	XP_004960601(95)^c^	PLAS	0.289
***PvNIP1;2***	Pavir.J36379.1	contig59709: 2228–4657	277	XP_004960601(93)^c^	PLAS	0.288
***PvNIP1;3***	Pavir.Eb00236.3	Chr05b: 3774233–3776780	290	XP_002454982 (89)^a^	PLAS	0.137
***PvNIP1;4***	Pavir.Ea00222.1	Chr05a: 2686340–2692046	287	XP_002454982(89)^a^	PLAS	0.113
***PvNIP1;5***	Pavir.Ab01231.1	Chr01b: 18627382–18630325	280	XP_004951368(93)^c^	PLAS	0.436
***PvNIP1;6***	Pavir.Db00851.1	Chr04b: 11796860–11798059	322	XP_004967095(73)^c^	PLAS	0.328
***PvNIP1;7***	Pavir.Da00802.1	Chr04a: 12785576–12787025	287	XP_004967095(83)^c^	PLAS	0.327
***PvNIP2;1***	Pavir.Ab02995.1	Chr01b: 52353467–52357364	296	XP_004953867(97)^c^	E.R	0.116
***PvNIP2;2***	Pavir.Aa00406.1	Chr01a: 4613561–4619572	313	XP_004953867(76)^c^	CHLO	0.082
***PvNIP2;3***	Pavir.Db01588.1	Chr04b: 35916639–35920941	295	XP_004965042(97)^c^	PLAS	0.423
***PvNIP2;4***	Pavir.Da01156.1	Chr04a: 22506078–22510755	296	XP_004965042(97)^c^	PLAS	0.049
***PvNIP3;1***	Pavir.J11993.1	contig143579: 44–1260	286	XP_004974441(80)^c^	VACU	0.460
***PvNIP3;2***	Pavir.J04994.1	contig07346: 8718–11701	292	XP_004974441(81)^c^	PLAS	0.255
***PvNIP3;3***	Pavir.Fb00252.1	Chr06b: 4491634–4492686	288	XP_004974441(84)^c^	CHLO	0.672
***PvNIP3;4***	Pavir.Fa01950.2	Chr06a: 45025793–45028094	330	XP_004974441(82)^c^	CYTO	0.153
***PvNIP3;5***	Pavir.Fa01948.1	Chr06a: 45006164–45007342	298	XP_004974438(87)^c^	CYTO	0.598
***PvNIP3;6***	Pavir.Fa01949.1	Chr06a: 45022689–45023983	278	XP_004974438(93)^c^	PLAS	0.560
***PvNIP3;7***	Pavir.J17719.1	contig194795: 1290–2237	291	XP_004974439(81)^c^	PLAS	0.315
***PvNIP3;8***	Pavir.J35034.1	contig50657: 4142–5117	295	XP_004974439(80)^c^	CYTO	0.398
***PvNIP3;9***	Pavir.Ib03684.1	Chr09b: 59774269–59780622	301	XP_004982621(98)^c^	PLAS	0.594
***PvNIP3;10***	Pavir.Ia01421.1	Chr09a: 15383290–15385609	281	XP_002464380(87)^a^	CHLO	0.561
***PvNIP4;1***	Pavir.Ea00764.2	Chr05a: 10523338–10525337	310	XP_004971599(86)^c^	PLAS	1.429
***PvNIP4;2***	Pavir.Ea00764.3	Chr05a: 10523338–10525337	308	XP_004971599(85)^c^	PLAS	1.309
***PvSIP1;1***	Pavir.J16825.1	contig18611: 427–3959	243	XP_004962139(95)^c^	PLAS	0.107
***PvSIP1;2***	Pavir.J10110.1	contig12910: 934–4304	241	XP_004962139(95)^c^	PLAS	0.060
***PvSIP2;1***	Pavir.Ia03463.1	Chr09a: 68891317–68893364	242	XP_004984561(97)^c^	NUCL	0.135
***PvSIP2;2***	Pavir.J37350.1	contig67361: 886–3091	242	XP_004984561(97)^c^	PLAS	0.164

Where, Ka and Ks are numbers of non-synonymous and synonymous substitutions per site, respectively. PPL: polypeptide length, aa: amino acid, PSCL: predicted subcellular localization, PLAS: plasma membrane. VACU: vacuolar membrane, CYTO: cytosol, ER: endoplasmic reticulum, MITO: mitochondrion, NUCL: Nucleous and CHLO: chloroplast.

^x^A gene that shows the highest identity with *MIP* in other plants by BLASTp. Parenthesis indicates the percentage of identity at the amino acid level.

^a^*Sorghum bicolor*

^b^*Zea mays*

^c^*Setaria italica* and

^d^*Oryza sativa Japonica Group*

^y^The same abbreviations have been used in Tables 1–4.

**Table 2:** *MIP* genes in *S*. *italica*.

Gene Name	Accession No.		Genomic location	PPL(aa)	Maximum Identity with other *MIP* (%)^x^	PSCL^y^	Ka/Ks value
	Phytozome	NCBI					
*SiPIP1;1*	Seita.1G264900	XP_004953388	scaffold_1:33821741..33825147	289	XP_002454508(99) ^a^	PLAS, CHLO	0
*SiPIP1;2*	Seita.7G196700	XP_004976483	scaffold_7:26986155..26988583	288	XP_002446929 (96)^a^	PLAS, CHLO	0.003
*SiPIP1;3*	Seita.1G372300	AET81042	scaffold_1:41718834..41720554	288	NP_001105131(97)^b^	PLAS, CHLO CHLO	0.077
*SiPIP1;4*	Seita.4G089800	XP_004964964	scaffold_4:7477219..7478601	299	XP_002438067(90)^a^	PLAS	0.433
*SiPIP2;1*	Seita.2G123300	XP_004956116	scaffold_2:13956110..13957549	286	XP_002461936(97)^a^	PLAS, CHLO	0.111
*SiPIP2;2*	Seita.2G123200	XP_004956115	scaffold_2:13928862..13930357	286	XP_002461936 (96)^a^	PLAS, CHLO	0.061
*SiPIP2;3*	Seita.7G170200	XP_004976254	scaffold_7:25196250..25198921	290	NP_001105616(96)^b^	PLAS, CHLO	0.396
*SiPIP2;4*	Seita.1G241900	XP_004953172	scaffold_1:31952120..31955237	288	NP_001105026(96)^b^	PLAS, CHLO	0.155
*SiPIP2;5*	Seita.2G123000	XP_004956113	scaffold_2:13905407..13908718	289	NP_001105024(97)^b^	PLAS, CHLO	0.161
*SiPIP2;6*	Seita.9G219400	XP_004986496	scaffold_9:16160701..16162490	294	NP_001105024(74)^b^	PLAS	0.443
*SiPIP2;7*	Seita.9G268100	-	scaffold_9:22654024..22655606	284	AFW68878(89)^b^	PLAS	0.647
*SiPIP2;8*	Seita.2G291500	XP_004957505	scaffold_2:38725943..38727530	285	ADW85675(84)^e^	PLAS	0.628
*SiTIP1;1*	Seita.5G469800	-	scaffold_5:47173769..47175771	268	NP_001045562(90)^d^	CYTO	0.444
*SiTIP1;2*	Seita.9G541300	XP_004985722	scaffold_9:56280555..56282371	249	P50156(94)^d^	VACU	0.350
*SiTIP2;1*	Seita.5G452400	XP_004971257	scaffold_5:46290359..46291626	243	NP_001047632(90)^d^	PLAS	0.906
*SiTIP2;2*	Seita.1G259900	XP_004953349	scaffold_1:33381748..33382889	249	NP_001047632(94)^a^	PLAS	0.154
*SiTIP2;3*	Seita.7G189600	XP_004976439	scaffold_7:26563113..26564278	248	DAA36542(98)^b^	PLAS	0.810
*SiTIP2;4*	Seita.4G160700	-	scaffold_4:24091200..24092502	252	XP_002438430(96)^a^	CYTO	0.141
*SiTIP2;5*	Seita.7G175600	XP_004965462	scaffold_4: 24091083–24092571	248	XP_002438430(98)^a^	CYTO	1.013
*SiTIP3;1*	Seita.9G571600	XP_004986028	scaffold_9:58297296..58298733	257	NP_001146930(80)^a^	CHLO	0.557
*SiTIP3;2*	Seita.9G208400	XP_004982756	scaffold_9:14971858..14973146	262	NP_001105032(95)^b^	MITO	0.284
*SiTIP4;1*	Seita.5G007300	XP_004967394	scaffold_5:510443..513569	246	NP_001105035(88)^b^	PLAS	0.324
*SiTIP4;2*	Seita.5G007400	XP_004967392	scaffold_5:517674..519546	246	DAA53302(84)^b^	CYTO	0.225
*SiTIP4;3*	Seita.5G007500	XP_004967395	scaffold_5:526125..527419	250	XP_002457071(88)^a^	VACU	0.487
*SiTIP4;4*	Seita.3G082100	-	scaffold_3:5238397..5241760	377	NP_001105034(86)^b^	CHLO	0.625
*SiTIP5;1*	Seita.1G259800	XP_004953348	scaffold_1:33380410..33381506	259	AAF90122(71)^e^	CHLO	0.785
*SiTIP5;2*	Seita.7G189500	-	scaffold_7:26561818..26562859	259	EMT13969(77)^f^	CHLO	1.152
*SiNIP1;1*	Seita.1G025100	XP_004951368	scaffold_1:2256093..2258861	278	NP_001105721(93)^a^	PLAS	0.420
*SiNIP1;2*	Seita.3G073300	XP_004960601	scaffold_3:4665483..4668824	281	XP_002440774(92)^a^	PLAS	0.393
*SiNIP1;3*	Seita.4G180100	XP_004967095	scaffold_4:29058134..29059299	286	AFW86958(75)^b^	CYTO	0.728
*SiNIP2;1*	Seita.6G063400	-	scaffold_4: 8475698–8479630	282	XP_002438105(90)^a^	PLAS	0.083
*SiNIP2;2*	Seita.4G098700	XP_004965042	scaffold_4:8475700..8479629	297	XP_002438105(95)^a^	PLAS	0.060
*SiNIP2;3*	Seita.1G318800	XP_004953867	scaffold_1:37930981..37934840	341	NP_001105637(93)^b^	PLAS	0.271
*SiNIP3;1*	Seita.6G062200	XP_004974438	scaffold_6:5185326..5186805	296	XP_002443852(90)^a^	VACU	0.297
*SiNIP3;2*	Seita.6G062300	XP_004972846	scaffold_6:5190608..5192100	286	XP_002445047(71)^a^	VACU	0.211
*SiNIP3;3*	Seita.6G063300	XP_004974441	scaffold_6:5255676..5256858	277	NP_001150784(75)^b^	CYTO	0.419
*SiNIP3;4*	Seita.6G062400	XP_004974439	scaffold_6:5192809..5193684	291	XP_002445042(69)^a^	CYTO	0.654
*SiNIP3;5*	Seita.9G193500	XP_004982621	scaffold_9:13788219..13792953	299	XP_002464380(98)^a^	PLAS	0.949
*SiNIP4;1*	Seita.5G076000	XP_004971599	scaffold_5:6518742..6520453	298	ACL53915.1 (79)^b^	PLAS	0.845
*SiSIP1;1*	Seita.3G248900	XP_004962139	scaffold_3:21344768..21347980	243	XP_002441068(92)^a^	PLAS	0.719
*SiSIP1;2*	Seita.8G085300	XP_004979029	scaffold_8:10243492..10260237	251	XP_002449310.1(89)^a^	PLAS	0.721
*SiSIP2;1*	Seita.9G422800	XP_004984561	scaffold_9:47875350..47877444	252	NP_001105640(93)^b^	NUCL	0.541

^x^A gene that shows the highest identity with *MIP* in other plants by BLASTp. Parenthesis indicates the percentage of identity at the amino acid level.

^a^*Sorghum bicolor*

^b^*Zea mays*

^d^*Oryza sativa Japonica Group*

^e^*Hordeum vulgare* and

^f^*Aegilops tauschii*

^y^The same abbreviations have been used in Tables 1–4.

**Table 3:** *MIP* genes in *S*. *biocolor*.

Gene Name	Accession No.		CL	Genomic location	PPL (aa)	Maximum identity with other *MIP* (%)^x^	PSCL^y^	Ka/Ks value
	Phytozome	NCBI						
***SbPIP1;1***	Sobic.006G176700.1	XP_002446929	6	53192023..53194107	288	AAO86706(98)^b^	PLAS, CHLO	0.102
***SbPIP1;2***	Sobic.004G288700.1	XP_002454508	4	63023013..63027206	289	ACF84511(99)^b^	PLAS, CHLO	0.039
***SbPIP1;3***	Sobic.004G351200.1	XP_002453072	4	67981023..67983212	290	NP_001105131(96)^b^	PLAS, CHLO	0.156
***SbPIP1;4***	Sobic.010G087900.1	XP_002438067	10	7521029..7522397	296	NP_001105023(94)^b^	PLAS	0.415
***SbPIP2;1***	Sobic.002G125700.2	XP_002461936	2	16980280..16982926	286	NP_001105027(98)^b^	PLAS, CHLO	0.050
***SbPIP2;2***	Sobic.002G125300.1	XP_002461933	2	16906986..16908387	286	NP_001105027 (97)^b^	PLAS, CHLO	0.048
***SbPIP2;3***	Sobic.002G125000.1	XP_002461931	2	16883369..16884816	296	NP_001105027(96)^b^	PLAS, CHLO	0.080
***SbPIP2;4***	Sobic.002G125200.1	XP_002461932	2	16897836..16899264	286	NP_001105027(96)^b^	PLAS, CHLO	0.135
***SbPIP2;5***	Sobic.004G222000.1	XP_002452483	4	57220820..57224296	289	NP_001105026(98)^b^	PLAS, CHLO	0.156
***SbPIP2;6***	Sobic.006G150100.1	XP_002446796	6	51145123..51147729	292	NP_001105616(95)^b^	PLAS, CHLO	0.134
***SbPIP2;7***	Sobic.002G124700.1	XP_002461930	2	16844700..16848362	290	NP_001105024(99)^b^	PLAS, CHLO	0.260
***SbPIP2;8***	Sobic.K007000.1	XP_002489214	U	2606152..2607000	282	AFW68878(94)^b^	PLAS	0.325
***SbPIP2;9***	Sobic.002G281000.2	-	2	66275305..66276988	289	XP_004957505(84) ^c^	PLAS, CHLO	0.456
***SbTIP1;1***	Sobic.001G505100.1	XP_002465859	1	77324938..77327995	250	NP_001104896(94)^b^	PLAS	0.376
***SbTIP1;2***	Sobic.003G445300.2	XP_002459183	3	74316138..74319335	258	ACF78734(91)^b^	CYTO	0.362
***SbTIP2;1***	Sobic.004G295100.1	XP_002452808	4	Sobic.004G295100.1	249	NP_001105030(90)^b^	PLAS	0.144
***SbTIP2;2***	Sobic.006G170600.1	XP_002448289	6	52722392..52723580	249	XP_004976439(96)^c^	PLAS	0.130
***SbTIP2;3***	Sobic.010G146100.1	XP_002438430	10	41392271..41394011	248	EAZ00793 (96) ^d^	CYTO	0.652
***SbTIP3;1***	Sobic.001G208500.1	XP_002467022	1	19088973..19090440	266	NP_001105032(94)^b^	MITO	0.361
***SbTIP3;2***	Sobic.006G155300.1	XP_002446824	6	51467369..51469051	268	DAA36836(89)^b^	PLAS	0.852
***SbTIP3;3***	Sobic.001G535900.2	XP_002468661	1	79929261..79930715	271	NP_001146930(88)^b^	PLAS	0.510
***SbTIP4;1***	Sobic.003G007200.1	XP_002457071	3	622245..623367	252	ACG39579(95)^b^	CYTO	0.591
***SbTIP4;2***	Sobic.009G085900.1	XP_002439483	9	14570383..14573259	314	ACG46456(92)^b^	VACU	0.567
***SbTIP4;3***	Sobic.003G006600.1	XP_002457068	3	572753..574763	318	DAA53302(88)^b^	VACU	0.550
***SbNIP1;1***	Sobic.003G026400.1	XP_002454982	3	2231972..2234369	271	AFW77428(77)^b^	CYTO	0.393
***SbNIP1;2***	Sobic.009G075900.1	XP_002440774	9	9905084..9909435	283	NP_001151947(92)^b^	PLAS	0.253
***SbNIP1;3***	Sobic.004G102200.1	XP_002453573	4	9450179..9453286	287	NP_001105721(94)^b^	CYSK	0.453
***SbNIP1;4***	Sobic.010G164100.1	-	10	48401814..48403745	291	XP_004967095(80)^c^	CYTO	0.453
***SbNIP2;1***	Sobic.004G238100.1	XP_002454286	4	58614722..58618581	297	NP_001105637(97)^b^	PLAS	0.343
***SbNIP2;2***	Sobic.010G092600.1	XP_002438105	10	8195416..8200323	295	NP_001105020(98)^b^	PLAS	0.512
***SbNIP3;1***	Sobic.007G039600.1	XP_002443852	7	3826797..3828613	297	Q7EYH7(77) ^d^	VACU	0.777
***SbNIP3;2***	Sobic.007G039500.1	XP_002445047	7	3812660..3815360	289	AFW61239 (77)^b^	VACU	0.343
***SbNIP3;3***	Sobic.007G038500.1	XP_002445042	7	3735702..3736780	297	AFW57375(70) ^b^	CYTO	0.512
***SbNIP3;4***	Sobic.001G195800.1	XP_002464380	1	17588588..17593923	301	ACN36318(95) ^b^	PLAS	1.053
***SbNIP4;1***	Sobic.003G098100.1	XP_002455311	3	8668414..8671066	289	ACL53915(85)^b^	PLAS	0.844
***SbSIP1;1***	Sobic.005G091600.1	XP_002449310	5	13565974..13569467	246	NP_001105514(92)^b^	PLAS	0.324
***SbSIP1;2***	Sobic.009G131500.1	XP_002441068	9	48499291..48503602	243	NP_001105028(96)^b^	PLAS	0.319
***SbSIP2;1***	Sobic.001G389900.1	XP_002465351	1	67642857..67645670	249	NP_001105640(94)^b^	CHLO	1.058

Where, CL: chromosome location, U: Unknown chromosomal location

^x^A gene that shows the highest identity with *MIP* in other plants by BLASTp. Parenthesis indicates the percentage of identity at the amino acid level.

^b^*Zea mays*

^c^*Setaria italica* and

^d^*Oryza sativa Japonica Group*

^y^The same abbreviations have been used in Tables 1–4.

**Table 4:** *MIP* genes in *B. distachyon*.

Gene Name	Accession No.		CL	Genomic Location	PPL(aa)	Maximum identity with other *MIP* (%)^x^	PSCL^y^	Ka/Ks value
	Phytozome	NCBI						
***BdPIP1;1***	Bradi5g18170.1	XP_003580312	5	21376355..21380359	288	AFV92901(97)^g^	PLAS, CHLO	0.203
***BdPIP1;2***	Bradi3g56020.1	XP_003570439	3	55807156..55808872	289	ABJ98535(96)^h^	PLAS, CHLO	0.086
***BdPIP2;1***	Bradi3g49360.1	XP_003575410	3	50482001..50485770	288	BAE02729(94)^e^	PLAS, CHLO	0.101
***BdPIP2;2***	Bradi5g15970.1	XP_003580150	5	19545026..19547614	287	BAF33069(93)^e^	PLAS, CHLO	0.099
***BdPIP2;3***	Bradi1g28760.1	XP_003563177	1	24115585..24118617	290	BAG06231(95)^e^	PLAS, CHLO	0.207
***BdPIP2;4***	Bradi1g28780.1	XP_003563179	1	24143877..24145345	289	NP_001105027(92)^a^	PLAS, CHLO	0.351
***BdPIP2;5***	Bradi4g36601.1	XP_003578538	4	41709704..41711325	290	ADW85675(89)^e^	PLAS	0.376
***BdPIP2;6***	Bradi4g36610.1	XP_003576780	4	41713192..41714682	297	ADW85675(79)^e^	PLAS	1.118
***BdPIP2;7***	Bradi1g00552.1	-	1	440975..442208	290	EMT26209(73)^f^	PLAS	0.369
***BdPIP2;8***	Bradi3g18460.1	XP_003571557	3	16901458..16902737	295	BAJ92749(76)^e^	PLAS	0.340
***BdTIP1;1***	Bradi1g75290.1	XP_003558815	1	72464538..72466271	250	CAA56553(92)^e^	VACU	0.264
***BdTIP1;2***	Bradi2g62520.1	XP_003565186	2	58924353..58925772	252	EMT32480(94)^f^	CYTO	0.473
***BdTIP2;1***	Bradi3g50690.1	XP_003570028	3	51583379..51584778	249	BAI66435(96)^e^	PLAS	0.116
***BdTIP2;2***	Bradi5g17690.1	XP_003580281	5	21052804..21053722	248	AAF90121(94)^e^	CHLO	0.157
***BdTIP3;1***	Bradi3g29780.1	XP_003574110	3	31567966..31569631	265	BAI66441(93)^e^	MITO	0.375
***BdTIP3;2***	Bradi5g16370.1	XP_003580181	5	19889474..19890848	262	BAK04817(82)^e^	CHLO	0.534
***BdTIP4;1***	Bradi2g07830.1	XP_003565529	2	6185272..6187133	252	EMT15368(91)^f^	CYTO	0.455
***BdTIP4;2***	Bradi2g31800.2	XP_003568717	2	31480793..31482685	252	BAI66438(91)^e^	VACU	0.448
***BdTIP4;3***	Bradi2g07810.1	XP_003566010	2	6166394..6167158	254	ACG39579(78)^a^	CHLO	0.512
***BdTIP5;1***	Bradi5g17680.1	XP_003581502	5	21051238..21052610	263	AAF90122(89)^e^	CHLO	1.016
***BdNIP1;1***	Bradi3g08930.1	XP_003571857	3	7053864..7055984	280	BAI66443(93)^e^	PLAS	0.229
***BdNIP1;2***	Bradi2g32890.1	XP_003568755	2	32572036..32574264	282	BAI66444(86)^e^	PLAS	0.315
***BdNIP1;3***	Bradi1g38160.1	XP_003560673	1	34458353..34459897	282	EMT31551(78)^f^	PLAS	0.521
***BdNIP2;1***	Bradi3g59390.1	-	3	58343770..58347486	296	BAH24163(88)^e^	E.R	0.421
***BdNIP2;2***	Bradi1g45200.1	XP_003564051	1	43568241..43572469	302	BAH84977(97)^e^	E.R	0.327
***BdNIP3;1***	Bradi3g30540.1	XP_003574178	3	32426723..32431422	301	EAY79189(89)^i^	PLAS	0.760
***BdNIP4;1***	Bradi2g01095.1	XP_003565246	2	672850..675385	285	BAK04446(69)^e^	PLAS	0.803
***BdSIP1;1***	Bradi4g26870.1	XP_003577906	4	31780346..31782559	246	BAJ86223(88)^e^	CHLO	1.629

^x^A gene that shows the highest identity with *MIP* in other plants by BLASTp. Parenthesis indicates the percentage of identity at the amino acid level.

^a^*Sorghum bicolor*

^e^*Hordeum vulgare*

^f^*Aegilops tauschii*

^**g**^*Lolium perenne*

^**h**^*Stipa baicalensis* and

^**i**^*Triticum urartu.*

^y^The same abbreviations have been used in Tables 1–4.

The Ka/Ks value was >1 for *PvPIP2;13*, *PvNIP3;10*, *PvNIP4;1*, *SiPIP1;3*, *SiTIP2;5*, *SiTIP5;2*, *SbNIP3;4*, *SbSIP2;1*, *BdPIP2;6*, *BdTIP5;1 and BdSIP1;*1 (Tables 1–4), indicating their positive or Darwinian selection. The remaining *MIP*s showed Ka/Ks values <1, demonstrating their purifying selection.

### Nomenclature and predicted subcellular localization of PvMIPs, SiMIPs, SbMIPs and BdMIPs

**Fig 1:**
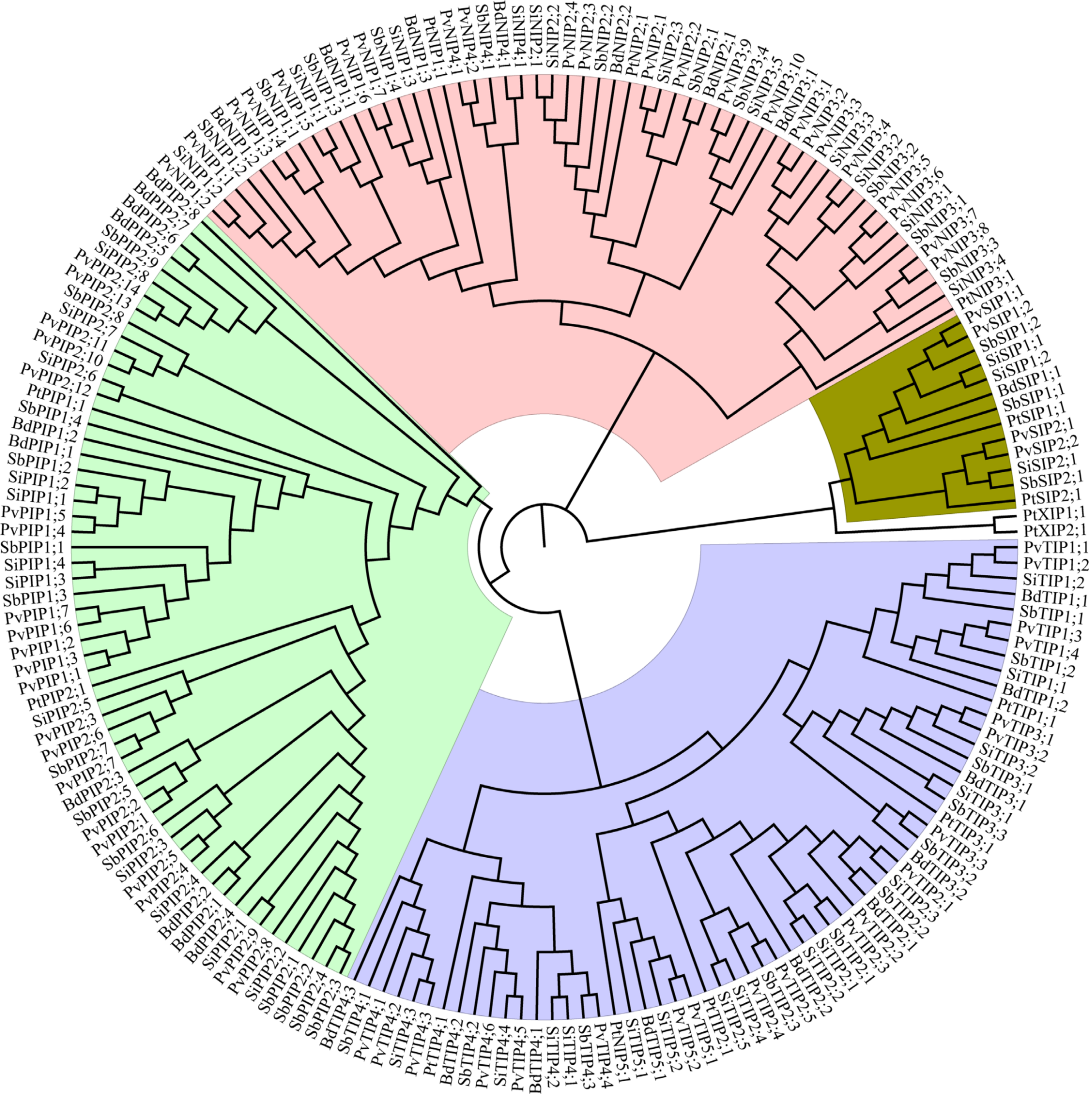
Evolutionary relationship of MIPs in the four grass plants. Phylogenetic analysis of all MIPs from the four grass plants is shown along with MIPs from poplar. The deduced amino acid sequences of MIPs were aligned using the Clustal Omega computer program and a phylogenetic tree was constructed using MEGA. The evolutionary history was inferred using the Bootstrap Neighbor-Joining (1000 replicates) method and the genetic distance was estimated by the p-distance method. PIPs, TIPs, NIPs and SIPs from the four plants clustered with the corresponding PtMIP subfamilies. Each MIP subfamily is shown with a specific background color to distinguish them from others.

The phylogenetic analysis showed that PvMIPs, SiMIPs, SbMIPs and BdMIPs were divided into four subfamilies. PIPs, TIPs, NIPs and SIPs of PvMIPs, SiMIPs SbMIPs and BdMIPs clustered with those subfamilies in the respective plant (Fig 1). However, no XIP was found. Sequences belonging to hybrid intrinsic proteins (HIPs) and a novel plant MIP (GIP, GlpF-like intrinsic protein) homologous to bacterial glycerol channel reported in the nonvascular moss *P*. *patens* [20] were not found. Fig 1 shows that most of the PIPs clustered either to PIP1s or PIP2s. However, some of the PIPs formed distinct clades from PIP1s and PIP2s. In contrast to PIP1s or PIP2s, they had no N- and C-terminal characteristic lengths [12], and in comparison with reference PIPs, they had the characteristic FPs (discussed later). The phylogenetic analysis with all PIPs from the 12 plants showed that these PIPs clustered with OsPIP2;7 and OsPIP2;8 (S1 Fig). Moreover, their percentage of identity at amino acid level with OsPIP2;7 and OsPIP2;8 (~65% to 80%) was higher than that with PpPIP3;1 (~54%). We therefore named these PIPs as PIP2s. The PvTIPs, SiTIPs and BdTIPs had five subgroups (TIP1 to TIP5) similar to TIPs in *Arabidopsis*, maize, poplar, rice and soybean. However, SbTIPs had four subgroups (SbTIP1 to SbTIP4). Four subgroups of NIPs were found in *P*. *virgatum*, S. *bicolor* and *B*. *distachyon*. Nevertheless, NIPs in *S*. *italica* had three subgroups. Although *Arabidopsis* and soybean have seven NIP subgroups [13,18], poplar, rice and maize have three to four NIP subgroups [11,12,17]. Similar to *Arabidopsis*, rice, maize, poplar and soybean, *P*. *virgatum*, *S*. *italica* and *S*. *bicolor* had two SIPs subgroups. However, *B*. *distachyon* had only one SIP of SIP1 subgroup.

PvPIPs, SiPIPs, SbPIPs and BdPIPs were predicted to be localized in the PM or both in the PM and chloroplast (Tables 1–4). However, the predicted subcellular loclization of TIPs was diversed including vacuole, PM, mitochondria, chloroplast and cytosol. Most of the NIPs were predicted to be localizd in the PM. However, some of the NIPs were predicted to be localized in any of the endoplasmic reticulum, choroplast, vacuole or cytosol. The predicted subcellular localization of SIPs was either in the PM or in the chloroplast. However, 1 PvSIP and 1 SiSIP were predicted to be localized in the nucleus. The amino acid lengths of PvMIP, SiMIP, SbMIP and BdMIP homologues with their maximum sequence identity with MIP in other plants are tabulated in Tables 1–4.

### Gene structure of *MIP*s in the four grass plants

**Fig 2:**
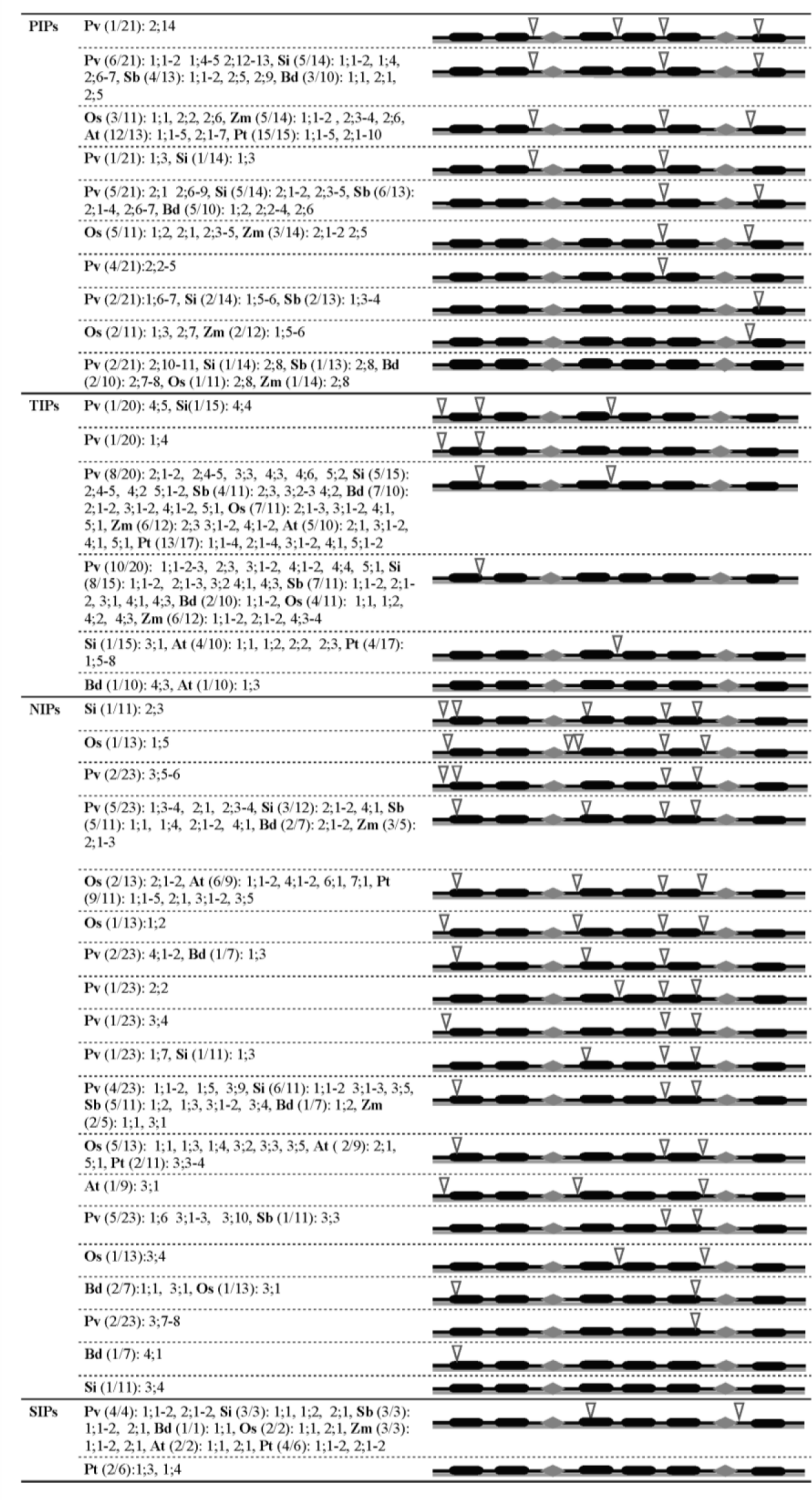
Gene structure of MIPs from grass plants, *P*. *trichocarpa* and *A*. *thaliana*. **Exon-intron organizations of *MIP* genes from grass plants are depicted for the PIP, TIP, NIP and SIP subfamilies.** The exon-intron pattern observed in the majority of *MIP*s within a subfamily is shown in gray background. In the parenthesis, the number of *MIP*s having that pattern is indicated for each plant species. For example, Pv (6/21) indicates that 6 out of 21 *PvPIP*s have the same gene structure. The members of homologue(s) are mentioned after the parenthesis. The six TM regions are shown in black bars and the loops B and E are shown in diamond shapes. The intron positions are indicated by inverted triangles.

All of the full-length *MIP* sequences found in *P*. *virgatum*, *S*. *italica*, *S*. *bicolor* and *B*. *distachyon* were analyzed for introns and exons. The introns in the *MIP*s of these plants were compared to *OsMIP*s and *ZmMIP*s of the two other grass plants as well as *AtMIP*s and *PtMIP*s of two non-grass plants (Fig 2). The number of introns varied from zero to five. However, apart from some disparities, the number and positions of introns were conserved within the subfamilies of *MIP*s in the grass plants. Nevertheless, major differences were observed when subfamilies from monocots were compared to those from dicots [11].

A comparison of members of the PIP subfamily revealed that among the grass plants only *PvPIP2;14* had four introns. Although the majority of *AtPIP*s and *PtPIP*s had three introns, only ~30% of *PIP*s in the six grass plants had three introns (Fig 2). The majority of *PIP*s in the grass plants had two introns because they lost one intron between helices H2 and H3; only PvPIP1;3 and SiPIP1;3 lost one intron between helices H5 and H6. Two *PIP* genes from each of *P*. *virgatum*, *S*. *italica*, *S*. *bicolor*, *O*. *sativa* and *Z*. *mays* had a single intron in the distal end of Loop E. The *P*. *virgatum* further had four *PIP* genes that carried a single intron between helices H4 and H5. Nonetheless, this intron position is conserved in all *PIP*s having more than one intron. Conversely, *B*. *distachyon* had no single intron bearing *PIP* gene. At least one *PIP* gene from each of *S*. *italica*, *S*. *bicolor*, *O*. *sativa* and *Z*. *mays* and two *PIP* genes from each of *P*. *virgatum* and *B*. *distachyon* had no intron. Members of the TIP subfamily showed the most stable gene structure in comparison with members of other subfamilies. The majority of *TIP*s in the grass plants including *Arabidopsis* and poplar had either two or one introns. Despite *PvTIP1;4*, *TIP*s with two introns had intron position at the end of helices H1 and H3. The position of the intron in *TIP*s having a single intron was at the end of helix H1. Two *TIP*s, each from *P*. *virgatum* and *S*. *italica*, had three introns. Similar to *AtTIP1;3*, only *BdTIP4;3* had a gene structure without any intron.

The gene structures of members of NIP subfamily in grass plants diverged from their counterparts in *Arabidopsis* and poplar (Fig 2). The majority of *NIP*s had four or three introns with highly variable introns organization. Similar to *OsNIP1;5*, the *SiNIP2;3* had five introns, which was the highest intron number among the MIPs. However, the intron positions in *SiNIP2;3* and *OsNIP1;5* were different. The *SiNIP3;4* possesed a unique gene structure without any intron. Similar to *AtSIP*s, all *SIP*s in the grass plants had two introns having highly conserved positions in helix H3 and loop E.

### Grouping of MIPs based on the ar/R selectivity filter and Froger's position

**Fig 3:**
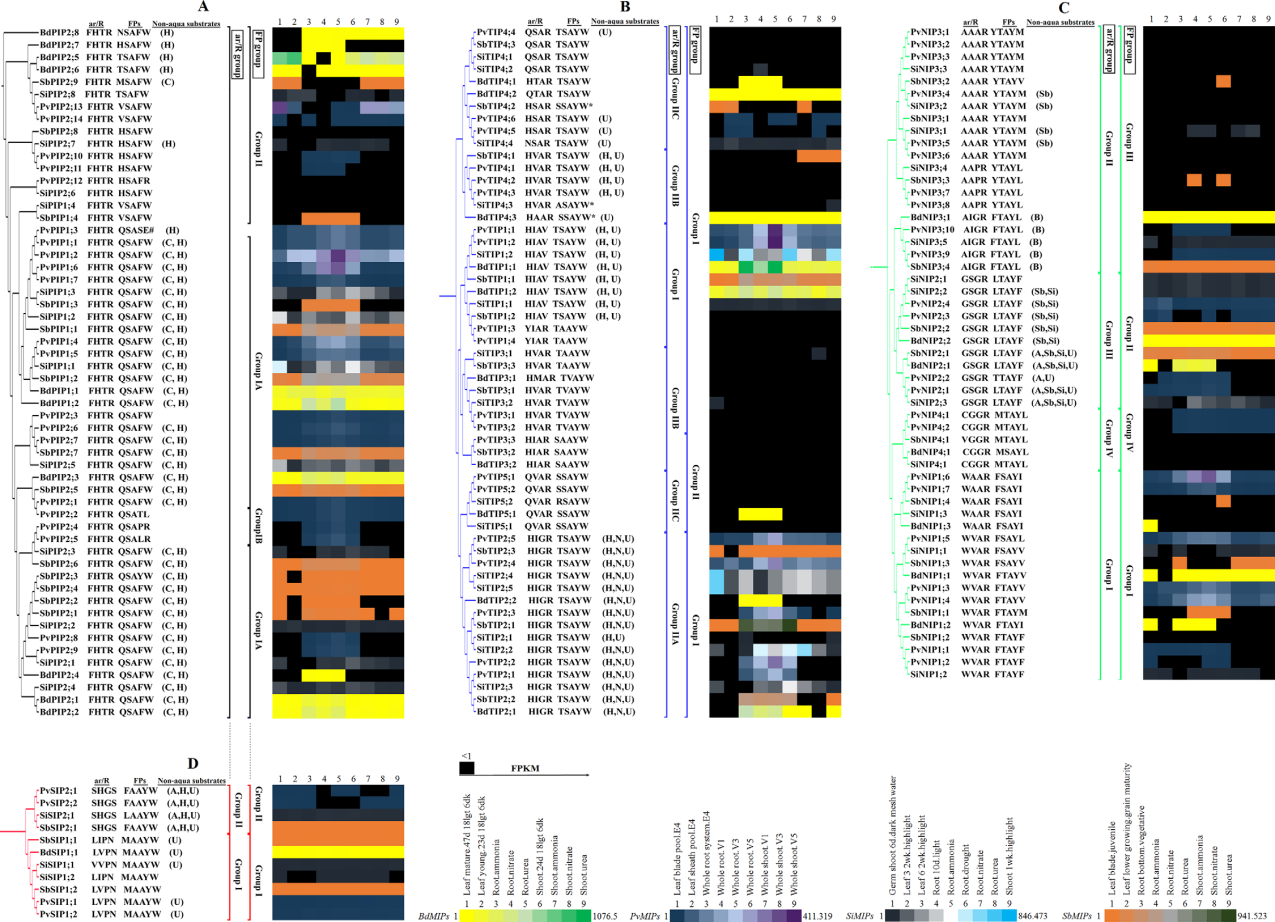
Grouping of MIPs based on the ar/R selectivity filter and FPs in the four grass plants and their expression profiles in different organs. The phylogenetic tree was generated as described in Fig 1. The residues in the ar/R selectivity filter and the FPs were selected from the 3D models as well as from the alignment shown in S2 and S3 Figs. The ar/R and FP groupings of PIPs (A), TIPs (B), NIPs (C), and SIPs (D), are indicated in the right side. # and * indicate the members of Group IB PIP and Group II TIP based on FPs, respectively. The non-aqua substrates predicted to be transported are mentioned. A, B, C, H, N, Sb, Si and U stand for arsenic, boron, CO_2_, H_2_O_2_, ammonia, antimony, silicon and urea, respectively. Expression heatmap in different organs are shown in the right side. Expression levels are given as the FPKM values.

To group the MIPs based on the ar/R selectivity filter and FPs, we constructed 3D models of all MIPs in *P*. *virgatum*, *S*. *italica*, *S*. *bicolor* and *B*. *distachyon*. The structure-based alignments and multiple sequence alignments of MIPs helped us to identify the four amino acid residues at the ar/R selectivity filter and the five residues in the FPs. The residues at the ar/R selectivity filter and in the FPs were considered to group MIPs and to compare these groups with those of the eight plants (Fig 3, S2 and S3 Figs). These groups were correlated with their expression and non-aqua transport profiles (discussed later).

The ar/R selectivity filters in all PIPs of the four grass plants contained residues F, H, T and R in H2, H5, LE1 and LE2, respectively (Fig 3A) identical to those found in *Arabidopsis*, maize, rice and *G*. *max*, and hence there was no group in PIPs based on this filter. Based on ar/R selectivity filters, all TIPs in *P*. *virgatum*, *S*. *italica*, *S*. *bicolor* and *B*. *distachyon* were grouped into two, Groups I and II, with different subgroups in Group II (Fig 3B). All members in TIP1 and TIP2 were in Group I and Group IIA, respectively, with the ar/R selectivity filter composed of HIAV and HIGR, correspondingly except PvTIP1;3 and PvTIP1;4 in which H in helix H2 was substituted by Y. All TIP3s and six members of TIP4 were in Group IIB with the tetrad composed of H, V/I/M, A and R. All members of TIP5 and most members of TIP4 in *P*. *virgatum*, *S*. *italica*, *S*. *bicolor* were sub-grouped to Group IIC having the residues Q/H/N, S/V/T, A and R. TIP Groups I, IIA and IIB in this study corresponded to those in *Arabidopsis* and *G*. *max*. However, the ar/R selectivity filter of TIP Group III, which was reported in *Arabidopsis*, *G*. *max* and poplar (S5 Fig; [11,18,30]), was not found in grass plants or cotton. Based on the ar/R selectivity filters, all NIPs were grouped into four (Fig 3C). All members of NIP1, NIP2, NIP3 and NIP4 were grouped to Groups I, III, II and IV, respectively. The tetrad of the ar/R selectivity filters in Group I (W, V/A, A and R) and Group II (A, A/I, A/P/G and R) were similar to those of Groups I and II, respectively in *Arabidopsis*, rice, maize, soybean, poplar and cotton. The tetrad of the ar/R filter in Group III (G, S, G and R) was conserved in the six glass plants as well as in soybean and poplar but was absent in *Arabidopsis*, cotton, *P*. *patens* and *S*. *moellendorffii* (Fig 3 and S6 Fig). The ar/R selectivity filter in NIPs of Group IV (C/V, G, G and R) was found only in grass plants but completely absent in other six plants. The SIPs were grouped into Group I and II based on the tetrad of the ar/R selectivity filter (Fig 3D). All SIP1s in the grass plants were clustered together with the ar/R filter composed of L/V, V/I, P and N which was fully conserved in some of the SIP1 members in other plants. All SIP2s were clustered into Group II with the conserved ar/R selectivity filter composed of S, H, G and S.

Based on the FPs, all PIPs from the four grass plants were clustered into two groups (Fig 3A). The P2-P5 positions were conserved in PIPs of both groups (Fig 3 and S3 Fig). While Gln was conserved in the P1 position in all members of Group I, the corresponding position in the homologues of Group II was substituted by H/V/T/M/N/E. The P3-P5 positions in all TIPs conserved the residues A, Y and W, respectively (Fig 3B). Based on the disparities in P1 and P2 positions, all TIPs could be divided into two groups. Despite three members of TIP3, all members of TIP1, TIP2, TIP3 and TIP4 were in Group I in which the P1 and P2 positions conserved T and S/V/A, respectively. All TIP5 members and a few members of TIP3 were in Group II in which the P1 and P2 positions conserved S and S/A, correspondingly. Similar FPs of Groups I and II TIPs were observed in rice, maize and other plants (S5 Fig). Based on the FPs, NIPs were clustered into four groups (Fig 3C). All NIP1 and NIP2 members were in Groups I and II, respectively, whereas all members of NIP3 and NIP4 clustered to Groups III and VI, individually. In all NIPs, P3 and P4 positions were conserved with A and Y, correspondingly. NIPs of rice and maize as well as other plants also followed this grouping (S6 Fig). Based on the FPs, all SIP1s and SIP2s clustered to Groups I and II, respectively, with the residues in P1-P5 positions correspondingly M, A, A, Y, W and F/L, A, A, Y, W (Fig 3D). However, the P2 position in other than grass plants was substituted by V.

### MIPs with unusual NPA motifs

Like their counterparts in other plants, all PIPs, TIPs, NIP1s and NIP2s in the four grass plants had dual conserved NPA motifs in loops B and E, respectively. In the NIPs with unusual NPA motifs, A of the NPA in Loop B was substituted by S and that in Loop E was substituted by V or I, as was found in poplar and other plants (Table 5). However, substitution of A with I in LE of PvNIP4;1–2 and SbNIP4;1 has not so far been reported although it is found in XIPs [11]. The NIPs with unusual NPA motifs in which A in loop B and that in loop E were substituted by S and V, respectively, had a characteristic Arg-rich C-termini (Table 5). In all SIPs in the grass plants, substitution of A by T (in SIP1s) or L (in SIP2s) in the NPA motif of Loop B was in agreement with other plants. The SIPs in all plants had the conserved NPA motif in Loop E with a unique characteristic Lys-rich C-termini (Table 5) which is a potential endoplasmic reticulum retention signal [1,49].

**Table 5:** NIPs and SIPs with unusual NPA motifs and the characteristic C-termini.

Plants	MIPs	NPA in LB*	NPA in LE*	C-terminal region
	NIPs			
*P*. *virgatum*	PvNIP3;9	NPS	NPV	-GETPRTQRSFRR
	PvNIP3;10	NPS	NPV	-GETPRAQRSFRR
	PvNIP4;1	NPA	NPI	-PHAIGAVASQQF
	PvNIP4;2	NPA	NPI	-PHAIGAVASQQF
*S*. *italica*	SiNIP3;5	NPS	NPV	-GETPRTQRSFRR
*S*. *bicolor*	SbNIP3;4	NPS	NPV	-GEAPRPQRSFRR
	SbNIP4;1	NPA	NPI	-RAVGSLASSPHY
*B*. *distachyon*	BdNIP3;1	NPS	NPV	-GEAPRPQRSFRR
	BdNIP4;1	NPA	NPV	-GRGGAAARSGSN
*O*. *setiva*	OsNIP3;1	NPS	NPV	-GETPRPQRSFRR
*Z*. *mays*	ZmNIP3;1	NPS	NPV	-GETPRTQRSFRR
*A*. *thaliana*	AtNIP1;2	NPA	NPG	-SFLKTVRNGSSR
	AtNIP5;1	NPS	NPV	-TDPPRPVRSFRR
	AtNIP6;1	NPA	NPV	-DEAPKERRSFRR
	AtNIP7;1	NPL	NPA	-SPVSPSVSSLLR
*P*. *trichocarpa*	PtNIP3;1	NPS	NPV	-NEKTSAARSFRR
	PtNIP3;2	NPS	NPV	-NEKTSATRSFRR
	PtNIP3;3	NPS	NPV	-ADPPRQVRSFRR
	PtNIP3;4	NPS	NPV	-TDPPRPVRSFRR
*G*. *max*	GmNIP5;1	NPS	NPV	-AEPPRQVRSFRR
	GmNIP6;2	NPA	NPV	-AKAKTSISSFRR
*G*. *hirsutum*	GhNIP6;1	NPA	NPV	-ILGSPCGCRTYT
*P*. *patens*	PpNIP3;1	NPA	NPV	-DPPRLPVRVFHR
	PpNIP6;1	NPA	NPM	-LAGTWTHTMLQI
*S*. *moellendorffii*	SmNIP3;2	NPA	NPI	-LGAGFYTLIRSS
	SmNIP6;2	NPS	NPA	-KPKKWGRNELLQ
	SmNIP5;4	NPA	NPC	-FKELERPKSFRR
	SmNIP7;2	NPS	NPA	-VLEGKEDSQNSM
	SIPs			
*P*. *virgatum*	PvSIP1;1	NPT	NPA	-LAPPPKPKAKKA
	PvSIP1;2	NPT	NPA	-LAPPPKPKAKKA
	PvSIP2;1	NPL	NPA	-TFLTKPKKIKEQ
	PvSIP2;2	NPL	NPA	-TFLTKPKKIKEQ
*S*. *italica*	SiSIP1;1	NPT	NPA	-LAPPPKPKAKKA
	SiSIP2;1	NPL	NPA	-EQEADENKTKKE
*S*. *bicolor*	SbSIP1;1	NPT	NPA	-LPPAPKPKTKKA
	SbSIP1;2	NPL	NPA	-LAPPPKPKAKKA
	SbSIP2;1	NPL	NPA	-EQEADENKTKKE
*B*. *distachyon*	BdSIP1;1	NPT	NPA	-PPPAPKPKAKKA
*O*. *sativa*	OsSIP1;1	NPT	NPA	-PPPAPKPKAKKA
	OsSIP2;1	NPL	NPA	-EEEADESKTKKE
*Z*. *mays*	ZmSIP1;1	NPT	NPA	-LPPAPKPKTKKA
	ZmSIP1;2	NPT	NPA	-LTPPPKPKAKKA
	ZmSIP2;1	NPL	NPA	-EQKVDENKIKKE
*A*. *thaliana*	AtSIP1;1	NPT	NPA	-PPRPQKKKQKKA
	AtSIP1;2	NPC	NPA	-APPLVQKKQKKA
	AtSIP2;1	NPL	NPA	-TEEQEKPKAKSE
*P*. *trichocarpa*	PtSIP1;1	NPT	NPA	-VFPPPAPKQKKT
	PtSIP1;2	NPT	NPA	-VFPPPAPKQKKA
	PtSIP2;1	NPL	NPA	-QDEKEKLKGKTE
	PtSIP2;2	NPL	NPA	-QDEKEKLKGKTD
*G*. *max*	GmSIP1;1	NPT	NPA	-PPAPRVVKQKKA
	GmSIP1;2	NPT	NPA	-VFPPRVVKQKKA
	GmSIP1;3	NPT	NPA	-PPPPPEVKQKKA
	GmSIP1;4	NPT	NPA	-PPSPPEVKQKKA
	GmSIP1;5	NPS	NPA	-SMFMPPIKQKKA
	GmSIP1;6	NPS	NPA	-SMFMPPIKQKKA
*G*. *hirsutum*	GhSIP1;2	NPT	NPA	-KKAKKTRKPKRA
	GhSIP1;3	NPT	NPA	-FSPSSSIKEKKA
*P*. *patens*	PpSIP1;1	NPT	NPA	-STGNAGDKMKAS
	PpSIP1;2	NPT	NPA	-LSENAAGKVKAS
*S*. *moellendorffii*	SmSIP1;2	NPT	NPA	-MFALGQNKEKTA

*LB and LE indicates loops B and E, respectively.

### Substrate-specific signature sequences or specificity-determining positions and non-aqua transport profiles of plant MIPs

The 3D models and the multiple sequence alignments of plant MIPs that have been shown experimentally to facilitate the transport of physiologically important non-aqua molecules such as ammonia, boron, CO_2_, H_2_O_2_, silicon and urea as well as toxic heavy metals arsenic and antimony [22,23,24,25,26,27,28,50,51,52,53,54,55] were analyzed for predicting substrate-specific signature sequences (SSSS) or specificity-determining positions (SDPs) in NPA regions, ar/R filter and FPs. The predicted SSSS or SDPs in these three constrictions in the experimentally proven MIPs are summarized in Table 6. All of the MIPs in each of the 12 plant genomes were subjected to ScanProsite tool (http://prosite.expasy.org/scanprosite/) to identify the SSSS or SDPs, and thereby the non-aqua transporters MIPs were predicted. Only the common homologues supported by all the characteristic SSSS or SDPs in the three constrictions (two NPA regions, ar/R selectivity filter and FPs) were listed as the transporter of the specific non-aqua molecule (Fig 3 and S4–S6 Figs).

**Table 6:** Substrate-specific signature sequences (SSSS) or specificity determining positions (SDPs) in MIPs transporting non-aqua substrates.

Substrate^a^	Sub-family	Signature sequences				References^c^
		Ar/R	NPA in Loop B	NPA in Loop E	FPs	
Ammonia (3.26 Å)	TIP	HI(G/A)R	SGGH(V/L)NPAVT	G(G/A)SMNPARSFG	TSAYW	[24]
	NIP	WVAR	SGGH(L/F)NPAVT	G(G/A)SMNPARSLG	FSAYL	
Antimonite (3.70 Å)	NIP	(G/A/T)(S/I/V/A)(G/A)R	SG(A/C)H(L/M)NP(S/A)(V/I/T)(T/S)	(G/S)(G/A)SMNP(V/A)R(T/S)L(G/A)	(L/F/Y/I)(T/S)AY(L/M/F)	[57]
Arsenic (4.00 Å)	NIP	(G/W/A)(V/S/I)(G/A)(R/V)	SGAH(L/M/I/V/)NP(A/S)(V/I)T	(G/S)(A/G)SMNP(A/V)R(T/S)(L/I)G	(L/F/Y)(T/S)AY(F/L/M)	[24]
	SIP*	SHGS	GGASYNPLT(I/V)	GG(I/V)MNPASAFA	(F/L)AAYW	
Boron (2.57 Å)	NIP	(A/G)(I/S)GR	SGAH(M/L/I)NP(A/S)(V/L)T	(G/S)(G/A)SMNP(A/V)R(S/T)LG	(F/I)TAY(F/L)	[24]
^b^CO_2_ (3.00 Å)	PIP	FHTR	SGGHINPAVT	GTGINPARSLG	(Q/M)SAFW	[24]
H_2_O_2_ (3.20 Å)	PIP	FHTR	SGGH(I/L/V/)NPAVT	GT(G/S)INPARS(L/F)G	(Q/F)SAFW	[24]
	TIP	HI(A/G)(R/V)	SGGH(V/L/I/)NPAVT	G(A/G)SMNPA(R/V)SFG	TSAYW	
	NIP	WVAR	SGAH(F/L/I/V)NPAVT	G(A/G)SMNPARSLG	FSAY(I/L)	
	SIP*	SHGS	GGASYNPLT(I/V)	GG(I/V)MNPASAFA	(F/L)AAYW	
^b^Silicon (4.38 Å)	NIP	GSGR	SGAHMNPA(V/L)T	GGSMNPARTL(G/A)	(L/I)TAYF	[69]
Urea (2.62 Å)	TIP	(H/G/N)(I/V)(A/G)(R/V/C)	SGGH(V/I/L/M)NPAVT	G(A/G)SMNPA(R/V/C)SFG	T(S/A)AYW	[24]
	NIP	(G/A)(S/I)AR	SGAH (M/ V/I/L/)NPAVT	(G/S)(A/G)SMNP(A/V)R(T/S)LG	(L/F/M/V/I)TAY(F/L)	
	SIP*	(L/V/I/A)(V/I/F/M/T)P(NF/I)	G(G/S)(V/A)(S/T)(F/W)NP(S/C/T/A)(T/A/G/D)(S/T/N/L/V/I/F)	(G/R)P(S/A)MNPA(N/F/I)A(F/Y)	(M/I)AAYW	

^a^ The diameter of the molecule is shown in the parenthesis.

^b^ SSSS or SDPs in two NPA regions, ar/R selectivity filter and FPs were determined by analyzing the MIPs that have been shown experimentally to transport CO_2_ and silicon [24] which synchronized with the report of Hove and Bhave [23].

^c^ The SSSS or SDPs were determined in this study by analyzing the experimental MIP homologues mentioned in the references within the parenthesis.

*SSSS and SDPs were not based on the experimental SIPs. SIPs that were predicted as arsenic, H_2_O_2_ and urea transporter based on the FPs of experimental PIPs, TIPs and NIPs, were used to predict the SSSS or SDPs in NPA regions, ar/R selectivity filter and FPs.

Our analysis showed that the predicted ammonia transporter MIPs were distributed to TIPs (TIP2s and TIP4s) (Fig 3 and S5 Fig), which was in agreement with experimental evidence [24]. This result indicated that ammonia transport through TIPs might be a conserved and ancient feature in higher plants since early branched plants such as *P*. *patens* and *S*. *moellendorffii* have no ammonia transporter. At least 5 MIPs from the four grass plants and 12 MIPs of the other plants were predicted to transport boron and were distributed only to members of NIP3, NIP5 and NIP6 except OsNIP2;1 (Fig 3 and S6 Fig). Boron transport in plants could be an ancestral feature as each of the 12 plants except *S*. *moellendorffii* had at least one NIP homologue predicted to be boron transporter. Our data showed that 36 PIPs in the four grass plants and 55 PIPs in the other 8 plants were predicted to be CO_2_ transporters with the highest and lowest numbers in cotton and *S*. *moellendorffii*, respectively (Fig 3 and S4 Fig). Despite AtPIP1;2 in *Arabidopsis*, no homologue in these 12 plants has experimental evidence, hence it would be interesting to test the CO_2_ permeability of these predicted PIPs in higher and lower plants. However, the plant MIPs especially in *Arabidopsis*, barley and tobacco, which have been experimentally proven to transport CO_2,_ are dispersed to PIPs [51,52,56]. Including a total of 72 MIPs in the four species, more than 139 MIP homologues in the 12 plants were predicted to facilitate the transport of H_2_O_2_ (Fig 3 and S4 and S5 Figs). These MIPs were mostly of PIPs and TIPs; the members of PIPs were of group I based on FPs (Fig 3 and S4 Fig, [24]). However, a few NIPs of group I from rice, poplar and *Arabidopsis*, and two HIPs each from *P*. *patens* and *S*. *moellendorffii* were predicted to be H_2_O_2_ transporters (S6 Fig). Data showed that all of the six grass plants had more than one silicon transporter and all were members of NIP2s (Fig 3 and S6 Fig). Furthermore, except PtNIP2;1, no silicon transporter was predicted in the other 5 plants. This result indicated that silicon transport might not be an ancestral characteristic and may be inherited based on the plant species. Each of the 12 plants had multiple urea transporters that were distributed to TIPs and NIPs (Fig 3 and S5 and S6 Figs). This result indicated that urea transport might be an ancestral characteristic of plants.

Phytotoxic antimony and arsenic transported through MIPs in the form of antimonite and arsenite, respectively can enter the food chain [25,57]. Our analysis predicted that the antimony and arsenic transporters were distributed only among the NIPs (either Group II or III NIPs based on the ar/R filter) in all grass plants including other higher plants (Fig 3 and S6 Fig). The antimony and arsenic transporter MIPs so far reported based on wet lab experiments are NIPs [25,57]. Therefore, antimony and arsenic transport through NIPs is a conserved and prehistoric characteristic. It was predicted that 24 MIPs from the 12 plants were arsenic transporters; of them 9 homologous were from the four grass plants (Fig 3 and S6 Fig), and among the six grass plants, *P*. *virgatum*, *O*. *sativa* had the highest number of arsenic transporters. However, A few PIP homologues in rice have been reported to have arsenic permeability [58]. Therefore, SSSS or SDPs prediction based on only a few PIP homologues might not be significant, and hence, PIPs were not considered in the analysis to predict arsenic transport.

Very few studies have examined the functions of SIPs. However, at least one of the two AtSIPs showed water channel activity when they were expressed in yeast [59]. Our analysis based on the SSSS or SDPs in the NPA regions, ar/R filter and FPs determined from the experimental PIPs, TIPs and NIPs did not detect their non-aqua transport. However, based on only the FPs, almost all SIP1s were predicted as urea transporters and SIP2s in the grass plants were predicted as transporters of arsenic and H_2_O_2_ in addition to urea (Fig 3D).

### MIPs predicted with multi, dual and single molecule transport activity

We defined a multichannel MIP when one MIP homologue was predicted to facilitate the transport of three or more than three non-aqua substrates. The total number of such MIPs in the four grass and in the other 6 higher plants was 18 and 37, respectively (Fig 3 and S5 and S6 Figs). However, this types of multichannel MIPs were not predicted in the lower plants, *P*. *patens* and *S*. *moellendorffii*. This result indicated that the multichannel MIPs were members of TIP2s and NIP2s. The 12 plants had a total of 136 MIP homologues that were predicted to transport two non-aqua substrates; 54 homologous were predicted in the four grass species (Fig 3 and S4–S6 Figs). A total of 78 MIP homologues in the 12 plants were predicted to transport only one non-aqua substrate.

### Expression of *MIP* genes in roots, shoots and leaves

The FPKM values obtained from the Phytozome could be assigned to 176 *MIP* genes of the four species. A heatmap showing their transcript levels in roots, shoots and leaves of the four plants was generated (Fig 3A–3D). The percentage of *MIP* genes in *P*. *virgatum*, *S*. *italica*, *S*. *bicolor* and *B*. *distachyon* expressed in at least one organ analyzed was 70, 76, 75 and 89, respectively, and that of *MIP*s in those plants expressed in all organs analyzed was 47, 59, 50 and 34, respectively. Among the *MIP*s, *PvTIP1;2* (FPKM = 411.5), *SiTIP1;1* (FPKM = 846.5), *SbTIP2;1* (FPKM = 941.5) and *BdTIP1;1* (FPKM = 1076) showed the highest expression in roots and these TIP homologues were ubiquitously expressed in all organs analyzed.

## Discussion

We identified and characterized a total of 176 MIP homologues from the genomes of four grass plants, *P*. *virgatum*, *S*. *italica*, *S*. *bicolor* and *B*. *distachyon* to predict and compare their structural properties and non-aqua transport functions to those in other two grass plants, rice and maize, as well as at least six non-grass plants comprising higher and lower plants. The genomes of all twelve plants included a total of 487 full-length MIP homologues. Therefore, this study provides a comparative particulars in context of their genome-wide number of homologues, subclasses or groups, non-aqua transport profile and structure-function relationships or non-aqua transport selectivity.

### The genome of *P*. *virgatum* has the largest number of MIP homologues

Although the number of MIP homologues varies from plant to plant, dicot plants comparatively have more homologues than monocot plants. Before our report, the highest known number of 66 full-length MIP homologues was shown in the genome of the dicot species *G*. *max* [18]. However, in the present study, we identified 68 full-length MIP homologues in a monocot species, *P*. *virgatum* (Table 1). This is the largest number of MIP homologues in a plant genome reported to date. It can be speculated that the polyploidy nature of *P*. *virgatum* resulted in duplication of these genes along the genome [33,42]. The large numbers of MIPs reflects wide diversity in substrate specificity, subcellular localization, transcriptional and post-translational regulation.

### Grass plants have the least number of MIP subfamilies

Similar to *Arabidopsis* [13], MIPs of grass plants comprise only four subfamilies, namely PIPs, TIPs, NIPs and SIPs (Fig 1), whereas MIPs of other higher plants with dicotyledon such as poplar, soybean, tomato and cotton have one more subfamily, XIPs [11,16,18,19]. The early-branched land plants, *P*. *patens* or mosses, possesses additional MIP subfamilies adding up to seven including GIPs and HIPs [20]. The PtMIPs and PpMIPs were chosen as queries so that XIPs, GIPs or HIPs could be detected if they were encoded in the genomes of grass plants. Occurrence of gene duplication as well as horizontal gene transfer during evolution is an important consideration for diversification of MIPs [33]. HIPs and GIPs might have been lost between the ancestor of early-branched vascular and seed plants and XIPs might have been lost between the ancestor and grass plants including *Arabidopsis*. Interestingly, although all higher plants have both SIP1s and SIP2s, *B*. *distachyon* possesses only SIP1 homologue as was found in lower plants, *P*. *patens* and *S*. *moellendorffii* [20,60]. This indicated that either SIP2s were present in the early-branched land plants but were subsequently lost in *B*. *distachyon*. It might be because of rapid divergence of SIP2s from SIP1 in *B*. *distachyon* as was suggested for *P*. *patens* and *S*. *moellendorffii*.

### Sub-cellular localizations and expression of plant MIPs are likely to be connected to their transport profiles

The sub-cellular localizations of plant MIPs are diversified, which might be connected to their functions. It was speculated that the same PIP localized in the PM and chloroplast might be responsible for transporting water and CO_2_, respectively [51,56]. Dual or multiple localizations might be coherent with the dual or multi channel activities of MIPs (Tables 1–4, Fig 3 and S4–S6 Figs). We guess that the PIPs predicted to transport CO_2_ are localized in the chloroplast in addition to PM. However, the score for localization in the chloroplast is lower than that in the PM. This is also applicable to the AtPIP1;2 in *Arabidopsis*, HvPIP2;1 in barley and NtAQP1 in *Nicotina tabaccum* (data not shown), which were shown experimentally to localize in PM and chloroplast and to transport CO_2_ [51,52,56]. Again PIP1 is localized in the PM when it is coexpressed with PIP2; if it is expressed alone, then it remains in the ER [61,62]. TIPs and NIPs exhibit multiple sub-cellular localizations and high functional diversity with transport of water, glycerol, H_2_O_2_, NH_3_, urea or metalloids such as arsenic, antimony, boron and silicon (Tables 1–4, Fig 3 and S5 and S6 Figs; [55,63]). The multiple sub-cellular localizations and diversified transport activities of MIPs are associated with osmoregulation and transcellular water transport, cell elongation, cell signaling, detoxification of excess urea, NH_3_ and H_2_O_2_ [3,27,36,55,64].

Data in the present study revealed that out of the 36 CO_2_ transporter PIPs, 32 were expressed in the leaves (Fig 3A). All predicted arsenic, silicon, boron, ammonia and H_2_O_2_ transporters were expressed in the roots. Nevertheless, most of these MIPs were also expressed in shoots and leaves. Similarly, almost all MIPs predicted to transport other non-aqua substrates such as antimony and urea were also ubiquitously expressed in the three organs roots, shoots and leaves. Interestingly, most of the unexpressed MIPs were not predicted to have non-aqua transport activity (Fig 3A–3D). These results indicate that the predicted non-aqua transport profiles of MIPs have a close relation with their expression. Again higher level of expression of some PIPs, TIPs and NIPs suggest that they have central physiological role in regulating water homeostasis, cell growth and cell expansion [4,36]. Therefore, the prediction of sub-cellular localization and expression profiles of MIPs in this study may be a nice direction for wet lab experiments to validate the relationship among the multiple sub-cellular localizations, expression and functional diversity.

### Non-aqua transport selectivity profile might be MIP group-specific

The non-aqua transport activities are mostly related to the phylogenetic framework of MIPs (Fig 3 and S4–S6 Figs). Group IA PIPs (based on FPs) from every plant were predicted to transport dual substrate CO_2_ and H_2_O_2_ (Fig 3A and S4 Fig). Because all the PIPs conserve the NPA motifs and the ar/R selectivity filter, their non-aqua transport selectivity profiles might be rendered by FPs. Group I PIPs usually differ from Group II PIPs by P1 position among the pore lining and their neighboring residues (S7 Fig**)**. The variety of hydogen bonding interaction of Gln and the substituted amino acid residue at P1 position (S8 Fig) might be a reason for the different conformation and thereby transport selectivity between group I and group II PIPs. The NH_2_ of polar Gln at the P1 position of group I PIPs may further influence the permeate molecules. Mutagenesis studies might be interesting to validate this hypothesis. However, the pore diameter and the transport profile might be regulated by post translational modification [5,6,47] and/or by heteromerization through physical interaction [62,65].

Since all the TIPs conserve the NPA motifs and also the FPs except some disparities (Fig 3B and S5 Fig), their non-aqua transport selectivity profiles might be rendered by the ar/R selectivity filter. The substitution of the Arg in the LE2 position by the smaller Val present in group I TIPs results in wider pore diameter (data not shown). We thus support other reports [36,66,67] that the wider pore apertures in group I TIPs might have facilitated the transport of larger non-aqua susbstrates such as urea and H_2_O_2_. However, ammonia transporters clustered only to group IIA in grass plants and also to group IIB in other plants (Fig 3B and S5 Fig), most of which had smaller pore diameter than the diameter of the ammonia molecule (data not shown). This indicates that pore diameter alone is not a determinant for selectivity of all non-aqua solutes. The regulatory events, biochemical properties of the filters and elsewhere or SDPs [23] might have effects on the transport selectivity profiles of TIPs. The TIP2s and TIP4s that have been predicted to be ammonia transporters have two motifs, G-L-x-y-G-G and P-x-H in loops B and C, respectively (S2 Fig and S9 Fig). The hydrophobic pore-linning conserved Leu, Pro and basic His with imidazole ring in these motifs might have imparted a more hydrophobic channel above the ar/R selectivity filter. The greater hydrophobicity of the channel might have aided the transport of ammonia [68]. Further studies such as mutagenesis are required to test the relevance of these motifs to ammonia transport.

The divergent NPA motifs, ar/R filter and FPs individually and/or collectively may play important roles in the substrate transport selectivity profiles of NIPs which were particularly predicted for metalloids such as arsenic, antimony, silicon and boron transporters in addition to H_2_O_2_ and urea (Fig 3C and S8 Fig). Conserved NPA motifs in silicon transporter and silicon non-transporter NIPs might have a limited role in the selectivity for silicon and also urea [37,69]. The ar/R filter in silicon transporter NIP2s characterized by the conserved G-S-G-R made the constriction wider [70]. The wider pore diameter of NIP2s might be one of the reasons to facilitate the transport of the bulkiest silicon molecule as well as urea, antimony and arsenic (Fig 3C and S6 Fig). In addition to NPA motifs, ar/R filter and FPs, the pore-lining highly conserved His in F/L-x-H-F-P motif in loop B may also influence the transport selectivity of metalloids in NIPs (S10 Fig). Hydrophobic Leu and Phe in the first position of this motif would be one of the determinants for boron and other metals such as arsenic, silicon and antimony, respectively. Interestingly, all of the predicted boron transporters conserved two unusual NPA motifs (NPS and NPV in Loops B and E, respectively) and Arg-enrich (R-x-x-R-S-F-R-R) C-termini (Table 5) that may play roles respectively in transport selectivity profiles and structural stabilization of the tetramers [49,71]. Furthermore, the highly conserved pore-lining SGGVTVP motifs in loop C of boron transporter NIPs might have important roles in the transport selectivity profile (data not shown).

The substitutions of corresponding positions of E14, H66, I187 and F200 of GlpF were focused to affect the width of the pore and the hydrophilic-hydrophobic pattern inside the channel in SIPs [72]. However, most of the substitutions were found to be SIP group-specific in the present study (Fig 3D, S11 Fig and S2 Table). Thus, the substrate selectivity profiles of SIP1s and SIP2s, notably both the width of the pore and the interior properties of the channels, are likely to differ. Comparison of the primary sequences of SIPs with GlpF and AQP1 suggests that SIPs are likely to transport solutes which are noble, hydrophobic and large in size [72]. It is usually supposed that MIPs with unusual NPA motifs may not transport water. However, water transport activity has been demonstrated in two AtSIP1s but not in AtSIP2;1 and the latter is supposed to have non-aqua transport activity [62]. Expression profiles of SIPs further indicated to have their transport activity. Nevertheless, wet-lab experiments are necessary to determine the intracellular localization, expression pattern and transport activities of SIPs.

## Conclusions

Analysis of genome sequences in four monocot grass plants revealed a new highest number of MIP homologues in *P*. *virgatum* without the recently discovered XIP subfamily in the grass plants. Further sequence and homology models analysis indicated that the signatures for substrate selectivity are group-specific, and like the ar/R selectivity filter, FPs can be an important basis for phylogenetic and functional groupings of MIP subfamilies. While the amino acid residue at the P1 position of FPs is one of the critical molecular determinants of the transport selectivity profiles of PIPs, residues at the ar/R filter and FPs are critical for substrate selectivity in TIPs and NIPs. Besides, the ar/R filter and FPs appear to work in coordination with pore-lining residues, particularly in loops B and C. Comparison of the predicted transport profiles with the expression profiles of MIPs in the four grass plants elucidateed a close correlation. The signature sequences or residues identified in the present study are important for predicting the transport profiles of uncharacterized MIPs. Prediction of the transport profiles and substrate selectivity of MIPs in the present study will provide an inroad to develop genetically modified plants that are tolerant to toxicity of heavy metals such as arsenic and antimony or deficiency of microelements and nutritionally better or healthier. However, the computational analysis-aided prediction for transport profiles, substrate selectivity and subcellular localization based on the critical primary sequence motifs and tertiary structural models of MIPs need to be validated by wet lab experiments.

## Supporting Information

S1 Fig Phylogenetic relationships of all PIPs from the 12 plants. The description of figure legend is as for Fig 1.
(TIF)

S2 Fig Homology models (green) of PvPIP2;1, PvTIP2;1, SiNIP3;5 and SiSIP1;1 superimposed with the models (red) of OsPIP2;1 (A), OsTIP2;1 (B), OsNIP3;1 (C) and OsSIP1;1 (D), respectively. A and D, the top views into the pore of PvPIP2;1 and SiSIP1;1, respectively, and B and C, the side views of PvTIP2;1 and SiNIP3;5, correspondingly. The 3D models of MIPs of the four grass plants were first constructed separately on the basis of the experimental structure of spinach PIP, SoPIP2;1(PDB ID:2B5F). Each of the 3D models of MIPs of the four grass plants was then superimposed on the MIP of other plants (only the representatives are shown). The residues that form the NPA box, ar/R filter and the FPs are shown as sticks. The residues of NPA, ar/R and FPs in PvPIP2;1, PvTIP2;1, SiNIP3;5 and SiSIP1;1 are shown in blue, green and yellow, respectively and those in OsMIPs are shown in black, red and pink, correspondingly and labeled. The TM α-helices and the loops to which they belong are indicated. The center of the pore is indicated as a black ball (A and D) and the path of the channel is indicated as the chain of red balls (B and C). The conserved pore-lining Leu in loop B and P-x-H in loop C found in predicted ammonia transporters TIP2s and TIP4s (B) and L-x-H-F-P in loop B and SGGVTVP found in predicted boron transporters NIP3s (C) are magenta; the same residues in the corresponding positions in OsTIP2;1 (B) and OsNIP3;1 (C) are cyan. The regions of NPA and ar/R selectivity filter and the conserved pore-lining residues in loops B and C in ammonia and boron transporters are boxed (B and C) and indicated by open arrows. The hydrogen bonding interaction between Pro and Val in loop C is shown by black dots.
(TIF)

S3 Fig NPA motifs, tetrad of ar/R filter and FPs in the MIPs of four grass plants. The amino acid sequences were aligned using the Clustal Omega sequence alignment program. From the multiple alignment, only structurally significant regions containing the NPA motifs, tetrad residues of ar/R filter and FPs are shown. The two conserved NPA motifs are bold, the residues at H2, H5, LE1, and LE2 of the ar/R filter are bold and underlined, FPs (P1-P5) are italic and underlined, conserved residues are shaded with grey.
(PDF)

S4 Fig Grouping of PIPs based on the FPs in *Arabidopsis* (At), rice (Os), maize (Zm), poplar (Pt), soybean (Gm), cotton (Gh) and moss (Pp). The description of the figure legend is as for Fig 3. Here, ^#^ and * indicate the members of group I and group II, respectively.
(TIF)

S5 Fig Grouping of TIPs based on the ar/R selectivity filter and FPs in *Arabidopsis*, rice, maize, poplar, soybean, cotton and moss. The description of the figure legend is as for Fig 3. Here, ^Ϯ^ and * indicate the members of group IIB of ar/R filter and group I of FPs, respectively.
(TIF)

S6 Fig Grouping of NIPs based on the ar/R selectivity filter and FPs in *Arabidopsis*, rice, maize, poplar, soybean, cotton and moss. The description of the figure legend is as for Fig 3. Here, * and ^#^ indicates the members of group I of FPs and Group IV of ar/R filter, respectively.
(TIF)

S7 Fig **Multiple sequence alignment of groups I (A) and II (B) PIPs of the twelve plants.** The amino acid sequences were aligned using the Clustal Omega program. The transmembrane helices and the dual NPA motifs are shown as gray and yellow, respectively. The residue (Q) at P1 position is shown as cyan. The pore-lining residues are indicated by arrows above the alignment and the conserved residues are indicated by stars (*) at the bottom of the alignment.
(PDF)

S8 Fig Intramolecular hydrogen-bonding interaction of the amino acid residue at the P1 position (A and B) and its possible role in pore conformation (C and D) in PIPs of Groups I and II. The Gln (Q) in P1 position of a Group I PIP is shown in magenta and its hydrogen bonding interactions with at least five amino acid residues are shown as black dashes (A). The hydrogen-bonding interaction of a substituted amino acid residue (magenta) at the corresponding position in a Group II PIP is shown as black dashes (B). The pore conformation (indicated by an open arrow) in the ar/R selectivity filter region (space-filling residues) of the same 3D models in (A) and (B) are shown in (C) and (D), respectively.
(TIF)

S9 Fig **Multiple sequence alignment of ammonia transporter TIP2s and TIP4s (A) and ammonia non-transporters (B) of the twelve plants. The c**onserved pore lining hydrophobic Leu in loop B and P-x-H in loop C are shown in the blue boxes. The description of the figure legend is as for Fig S9.
(PDF)

S10 Fig **Multiple sequence alignment of silicon transporter (A) and silicon non-transporter (B) NIPs of the twelve plants. The c**onserved pore lining F/L-x-H-F-P motif in loop B is shown in the blue boxes. The description of the figure legend is as for S9 Fig.
(PDF)

S11 Fig Multiple sequence alignment of SIPs with GlpF and AQP1. The amino acid sequences were aligned using the Clustal Omega sequence alignment program. Two NPA motifs, the residues at H2, H5, LE1, and LE2 of the ar/R filter and FPs (P1-P5) are yellow, green and cyan, respectively. The SIP group-specific residues corresponding to structurally important residues in GlpF shown by Fu et al. (2000) are in open boxes. The group-specific residues at TM5, LE and TM6, which may also have structural and/or functional roles, are shown in blue boxes. The star (*) at the bottom of the alignment indicates the conserved residues.
(PDF)

S1 Table MIPs discarded from the four grass plants. (PDF)

S2 Table Structurally important SIP group-specific amino acids and the role of residues in the corresponding positions in the structure of GlpF and AQP1 (or both). (PDF)

## Abbreviations

AQPs: Aquaporins
ar/R: Aromatic/arginine
FPs: Froger’s positions
FPKM: Fragments per Kilobase of Transcript per Million Mapped Reads
GIPs: GlpF-like intrinsic proteins
H2: Helix 2
H5: Helix 5
HIPs: Hybrid intrinsic proteins
MEGA: Molecular evolution genetic analysis
MIPs: Major intrinsic proteins
MOE: Molecular operating environment software
NIPs: Nodulin-26-like intrinsic proteins
PDB: Protein data bank
PIPs: Plasma membrane intrinsic proteins
PM: Plasma membrane
SDPs: Specificity-determining positions
SIPs: Small basic intrinsic proteins
SSSS: Substrate-specific signature sequences
TIPs: Tonoplast intrinsic proteins
TM: Transmembrane
WGS: Whole genome shotgun sequence
XIPs: Uncharacterised X intrinsic proteins

## References

[1] Gomes D, Agasse A, Thiébaud P, Delrot S, Gerós H, Chaumont F (2009) Aquaporins are multifunctional water and solute transporters highly divergent in living organisms. Biochimica et Biophysica Acta-Biomembranes 1788: 1213–1228. 10.1016/j.bbamem.2009.03.009 19327343

[2] Maurel C, Verdoucq L, Luu DT, Santoni V (2008) Plant aquaporins: membrane channels with multiple integrated functions. Annual Review Plant Biology 59: 595–624. 10.1146/annurev.arplant.59.032607.092734 18444909

[3] Muto Y, Segami S, Hayashi H, Sakurai J, Murai-Hatano M, Hattori Y et al (2011) Vacuolar proton pumps and aquaporins involved in rapid internode elongation of deepwater rice. Bioscience, Biotechnology, and Biochemistry 75: 114–122. 21228479 10.1271/bbb.100615

[4] Azad AK, Hanawa R, Ishikawa T, Sawa Y, Shibata H (2013) Expression profiles of aquaporin homologues and petal movement during petal development in *Tulipa gesneriana*. Physiologia Plantarum 148: 397–407. 10.1111/j.1399-3054.2012.01717.x 23088645

[5] Azad AK, Katsuhara M, Sawa Y, Ishikawa T, Shibata H (2008) Characterization of four plasma membrane aquaporins in tulip petals: a putative homolog is regulated by phosphorylation. Plant and Cell Physiology 49: 1196–1208. 10.1093/pcp/pcn095 18567892

[6] Azad AK, Sawa Y, Ishikawa T, Shibata H (2004) Phosphorylation of plasma membrane aquaporin regulates temperature-dependent opening of tulip petals. Plant and Cell Physiology 45: 608–617. 15169943 10.1093/pcp/pch069

[7] Uehlein N, Kaldenhoff R (2008) Aquaporins and plant leaf movements. Annals of Botany 101: 1–4. 18024416 10.1093/aob/mcm278 PMC2701841

[8] Gao Z, He X, Zhao B, Zhou C, Liang Y, Ge R et al (2010) Overexpressing a putative aquaporin gene from wheat, *TaNIP*, enhances salt tolerance in transgenic *Arabidopsis*. Plant and Cell Physiology 51: 767–775. 10.1093/pcp/pcq036 20360019

[9] Peng Y, Lin W, Cai W, Arora R (2007) Overexpression of a *Panax ginseng* tonoplast aquaporin alters salt tolerance, drought tolerance and cold acclimation ability in transgenic *Arabidopsis* plants. Planta 226: 729–740. 17443343 10.1007/s00425-007-0520-4

[10] Jeyaseelan K, Sepramaniam S, Armugam A, Wintour EM (2006) Aquaporins: a promising target for drug development. Expert Opinion on Therapeutic Targets 10: 889–909 17105375 10.1517/14728222.10.6.889

[11] Gupta AB, Sankararamakrishnan R (2009) Genome-wide analysis of major intrinsic proteins in the tree plant *Populus trichocarpa*: characterization of XIP subfamily of aquaporins from evolutionary perspective. BMC Plant Biology 9: 134 10.1186/1471-2229-9-134 19930558 PMC2789079

[12] Chaumont F, Barrieu F, Wojcik E, Chrispeels MJ, Jung R (2001) Aquaporins constitute a large and highly divergent protein family in maize. Plant Physiology 125: 1206–1215. 11244102 10.1104/pp.125.3.1206 PMC65601

[13] Johanson U, Karlsson M, Johansson I, Gustavsson S, Sjövall S, Fraysse L et al (2001) The complete set of genes encoding major intrinsic proteins in *Arabidopsis* provides a framework for a new nomenclature for major intrinsic proteins in plants. Plant Physiology 126: 1358–1369. 11500536 10.1104/pp.126.4.1358 PMC117137

[14] Bansal A, Sankararamakrishnan R (2007) Homology modeling of major intrinsic proteins in rice, maize and *Arabidopsis*: comparative analysis of transmembrane helix association and aromatic/arginine selectivity filters. BMC Structural Biology 7: 27 17445256 10.1186/1472-6807-7-27 PMC1866351

[15] Park W, Scheffler BE, Bauer PJ, Campbell BT (2010) Identification of the family of aquaporin genes and their expression in upland cotton (*Gossypium hirsutum* L.). BMC Plant Biology 10: 142 10.1186/1471-2229-10-142 20626869 PMC3095289

[16] Reuscher S, Akiyama M, Mori C, Aoki K, Shibata D, Shiratake K (2013) Genome-wide identification and expression analysis of aquaporins in tomato. PloS One 8: e79052 10.1371/journal.pone.0079052 24260152 PMC3834038

[17] Sakurai J, Ishikawa F, Yamaguchi T, Uemura M, Maeshima M (2005) Identification of 33 rice aquaporin genes and analysis of their expression and function. Plant and Cell Physiology 46: 1568–1577. 16033806 10.1093/pcp/pci172

[18] Zhang DY, Ali Z, Wang CB, Xu L, Yi JX, Xu ZL et al (2013) Genome-wide sequence characterization and expression analysis of major intrinsic proteins in soybean (*Glycine max* L.). PloS One 8: e56312 10.1371/journal.pone.0056312 23437113 PMC3577755

[19] Venkatesh J, Yu J-W, Park SW (2013) Genome-wide analysis and expression profiling of the *Solanum tuberosum* aquaporins. Plant Physiology and Biochemistry 73: 392–404. 10.1016/j.plaphy.2013.10.025 24215931

[20] Danielson JÅ, Johanson U (2008) Unexpected complexity of the aquaporin gene family in the moss *Physcomitrella patens*. BMC Plant Biology 8: 45 10.1186/1471-2229-8-45 18430224 PMC2386804

[21] Bienert GP, Bienert MD, Jahn TP, Boutry M, Chaumont F (2011) Solanaceae XIPs are plasma membrane aquaporins that facilitate the transport of many uncharged substrates. The Plant Journal 66: 306–317. 10.1111/j.1365-313X.2011.04496.x 21241387

[22] Hachez C, Chaumont F (2010) Aquaporins: a family of highly regulated multifunctional channels MIPs and their Role in the Exchange of Metalloids: Springer pp. 1–17. 10.1007/978-1-4419-6315-4_1 20666220

[23] Hove RM, Bhave M (2011) Plant aquaporins with non-aqua functions: deciphering the signature sequences. Plant Molecular Biology 75: 413–430. 10.1007/s11103-011-9737-5 21308399

[24] Di Giorgio JP, Soto G, Alleva K, Jozefkowicz C, Amodeo G, Muschietti JP et al (2014) Prediction of Aquaporin Function by Integrating Evolutionary and Functional Analyses. The Journal of Membrane Biology 247: 107–125. 10.1007/s00232-013-9618-8 24292667

[25] Mukhopadhyay R, Bhattacharjee H, Rosen BP (2014) Aquaglyceroporins: Generalized metalloid channels. Biochimica et Biophysica Acta-General Subjects 1840: 1583–1591. 10.1016/j.bbagen.2013.11.021 PMC3960311 24291688

[26] Takano J, Wada M, Ludewig U, Schaaf G, Von Wirén N, Fujiwara T (2006) The Arabidopsis major intrinsic protein NIP5; 1 is essential for efficient boron uptake and plant development under boron limitation. The Plant Cell 18: 1498–1509. 16679457 10.1105/tpc.106.041640 PMC1475503

[27] Bienert GP, Chaumont F (2014) Aquaporin-facilitated transmembrane diffusion of hydrogen peroxide. Biochimica et Biophysica Acta- General Subjects 1840: 1596–1604. 10.1016/j.bbagen.2013.09.017 24060746

[28] Ma JF, Yamaji N (2006) Silicon uptake and accumulation in higher plants. Trends in Plant Science 11: 392–397. 16839801 10.1016/j.tplants.2006.06.007

[29] Wang W-H, Köhler B, Cao F-Q, Liu L-H (2008) Molecular and physiological aspects of urea transport in higher plants. Plant Science 175: 467–477.

[30] Wallace IS, Roberts DM (2004) Homology modeling of representative subfamilies of *Arabidopsis* major intrinsic proteins. Classification based on the aromatic/arginine selectivity filter. Plant Physiology 135: 1059–1068. 15181215 10.1104/pp.103.033415 PMC514140

[31] Tajkhorshid E, Nollert P, Jensen MØ, Miercke LJ, O'Connell J, Stroud RM et al (2002) Control of the selectivity of the aquaporin water channel family by global orientational tuning. Science 296: 525–530. 11964478 10.1126/science.1067778

[32] Heymann JB, Engel A (2000) Structural clues in the sequences of the aquaporins. Journal of Molecular Biology 295: 1039–1053. 10656809 10.1006/jmbi.1999.3413

[33] Zardoya R (2005) Phylogeny and evolution of the major intrinsic protein family. Biology of the Cell 97: 397–414. 15850454 10.1042/BC20040134

[34] Fu D, Libson A, Miercke LJ, Weitzman C, Nollert P, Krucinski J et al (2000) Structure of a glycerol-conducting channel and the basis for its selectivity. Science 290: 481–486. 11039922 10.1126/science.290.5491.481

[35] Sui H, Han B-G, Lee JK, Walian P, Jap BK (2001) Structural basis of water-specific transport through the AQP1 water channel. Nature 414: 872–878. 11780053 10.1038/414872a

[36] Azad AK, Yoshikawa N, Ishikawa T, Sawa Y, Shibata H (2012) Substitution of a single amino acid residue in the aromatic/arginine selectivity filter alters the transport profiles of tonoplast aquaporin homologs. Biochimica et Biophysica Acta -Biomembranes 1818: 1–11. 10.1016/j.bbamem.2011.09.014 21963407

[37] Wallace IS, Roberts DM (2005) Distinct transport selectivity of two structural subclasses of the nodulin-like intrinsic protein family of plant aquaglyceroporin channels. Biochemistry 44: 16826–16834. 16363796 10.1021/bi0511888

[38] Froger A, Thomas D, Delamarche C, Tallur B (1998) Prediction of functional residues in water channels and related proteins. Protein Science 7: 1458–1468. 9655351 10.1002/pro.5560070623 PMC2144022

[39] Somerville C (2006) The billion-ton biofuels vision. Science 312: 1277 16741078 10.1126/science.1130034

[40] Vogel JP, Garvin DF, Mockler TC, Schmutz J, Rokhsar D, Bevan MW et al (2010) Genome sequencing and analysis of the model grass *Brachypodium distachyon*. Nature 463: 763–768. 10.1038/nature08747 20148030

[41] Aimar D, Calafat M, Andrade A, Carassay L, Abdala G, Molas ML (2011) Drought tolerance and stress hormones: From model organisms to forage crops. Plants and Environment: 137–164.

[42] Young HA, Lanzatella CL, Sarath G, Tobias CM (2011) Chloroplast genome variation in upland and lowland switchgrass. PloS One 6: e23980 10.1371/journal.pone.0023980 21887356 PMC3161095

[43] Zhang G, Liu X, Quan Z, Cheng S, Xu X, Pan S et al (2012) Genome sequence of foxtail millet (*Setaria italica*) provides insights into grass evolution and biofuel potential. Nature Biotechnology 30: 549–554. 10.1038/nbt.2195 22580950

[44] Paterson AH, Bowers JE, Bruggmann R, Dubchak I, Grimwood J, Gundlach H et al (2009) The *Sorghum bicolor* genome and the diversification of grasses. Nature 457: 551–556. 10.1038/nature07723 19189423

[45] Lomsadze A, Ter-Hovhannisyan V, Chernoff YO, Borodovsky M (2005) Gene identification in novel eukaryotic genomes by self-training algorithm. Nucleic Acids Research 33: 6494–6506. 16314312 10.1093/nar/gki937 PMC1298918

[46] Tamura K, Peterson D, Peterson N, Stecher G, Nei M, Kumar S (2011) MEGA5: molecular evolutionary genetics analysis using maximum likelihood, evolutionary distance, and maximum parsimony methods. Molecular Biology and Evolution 28: 2731–2739. 10.1093/molbev/msr121 21546353 PMC3203626

[47] Tornroth-Horsefield S, Wang Y, Hedfalk K, Johanson U, Karlsson M, Tajkhorshid E et al (2006) Structural mechanism of plant aquaporin gating. Nature 439: 688–694. 16340961 10.1038/nature04316

[48] Pellegrini-Calace M, Maiwald T, Thornton JM (2009) PoreWalker: a novel tool for the identification and characterization of channels in transmembrane proteins from their three-dimensional structure. PLoS Computational Biology 5: e1000440 10.1371/journal.pcbi.1000440 19609355 PMC2704872

[49] Ishibashi K (2006) Aquaporin subfamily with unusual NPA boxes. Biochimica et Biophysica Acta -Biomembranes 1758: 989–993. 10.1016/j.bbamem.2006.02.024 16579962

[50] Tanaka M, Wallace IS, Takano J, Roberts DM, Fujiwara T (2008) NIP6;1 is a boric acid channel for preferential transport of boron to growing shoot tissues in *Arabidopsis*. The Plant Cell 20: 2860–2875. 10.1105/tpc.108.058628 18952773 PMC2590723

[51] Heckwolf M, Pater D, Hanson DT, Kaldenhoff R (2011) The *Arabidopsis thaliana* aquaporin AtPIP1; 2 is a physiologically relevant CO_2_ transport facilitator. The Plant Journal 67: 795–804. 10.1111/j.1365-313X.2011.04634.x 21564354

[52] Mori IC, Rhee J, Shibasaka M, Sasano S, Kaneko T, Horie T et al (2014) CO_2_ Transport by PIP2 Aquaporins of Barley. Plant and Cell Physiology 55: 251–257. 10.1093/pcp/pcu003 24406630 PMC3913445

[53] Dynowski M, Schaaf G, Loque D, Moran O, Ludewig U (2008) Plant plasma membrane water channels conduct the signalling molecule H_2_O_2_. Biochemistry Journal 414: 53–61. 10.1042/BJ20080287 18462192

[54] Yang H, Menz J, Häussermann I, Benz M, Fujiwara T, Ludewig U (2015) High and low affinity urea root uptake: involvement of NIP5;1. Plant and Cell Physiology 56: 1588 10.1093/pcp/pcv067 25957355

[55] Soto G, Fox R, Ayub N, Alleva K, Guaimas F, Erijman EJ et al (2010) TIP5;1 is an aquaporin specifically targeted to pollen mitochondria and is probably involved in nitrogen remobilization in *Arabidopsis thaliana*. The Plant Journal 64: 1038–1047. 10.1111/j.1365-313X.2010.04395.x 21143683

[56] Uehlein N, Otto B, Hanson DT, Fischer M, McDowell N, Kaldenhoff R (2008) Function of *Nicotiana tabacum* aquaporins as chloroplast gas pores challenges the concept of membrane CO_2_ permeability. The Plant Cell 20: 648–657. 10.1105/tpc.107.054023 18349152 PMC2329941

[57] Bienert GP, Thorsen M, Schüssler MD, Nilsson HR, Wagner A, Tamás MJ et al (2008) A subgroup of plant aquaporins facilitate the bi-directional diffusion of As(OH)_3_ and Sb(OH)_3_ across membranes. BMC Biology 6: 26 10.1186/1741-7007-6-26 18544156 PMC2442057

[58] Mosa KA, Kumar K, Chhikara S, Mcdermott J, Liu Z, Musante C et al (2012) Members of rice plasma membrane intrinsic proteins subfamily are involved in arsenite permeability and tolerance in plants. Transgenic Research 21: 1265–1277. 10.1007/s11248-012-9600-8 22350764

[59] Ishikawa F, Suga S, Uemura T, Sato MH, Maeshima M (2005) Novel type aquaporin SIPs are mainly localized to the ER membrane and show cell-specific expression in *Arabidopsis thaliana*. FEBS Letters 579: 5814–5820. 16223486 10.1016/j.febslet.2005.09.076

[60] Anderberg HI, Kjellbom P, Johanson U (2012) Annotation of *Selaginella moellendorffii* major intrinsic proteins and the evolution of the protein family in terrestrial plants. Frontiers in Plant Science 3: 33 10.3389/fpls.2012.00033 22639644 PMC3355642

[61] Jozefkowicz C, Rosi P, Sigaut L, Soto G, Pietrasanta LI, Amodeo G et al (2013) Loop A is critical for the functional interaction of two *Beta vulgaris* PIP aquaporins. PloS One 8: e57993 10.1371/journal.pone.0057993 23483963 PMC3587573

[62] Fetter K, Van Wilder V, Moshelion M, Chaumont F (2004) Interactions between plasma membrane aquaporins modulate their water channel activity. The Plant Cell 16: 215–228. 14671024 10.1105/tpc.017194 PMC301406

[63] Gattolin S, Sorieul M, Frigerio L (2010) Mapping of tonoplast intrinsic proteins in maturing and germinating *Arabidopsis* seeds reveals dual localization of embryonic TIPs to the tonoplast and plasma membrane. Molecular Plant 4: 180–9. 10.1093/mp/ssq051 20833734

[64] Wudick MM, Luu DT, Maurel C (2009) A look inside: localization patterns and functions of intracellular plant aquaporins. New Phytologist 184: 289–302. 10.1111/j.1469-8137.2009.02985.x 19674338

[65] Yaneff A, Sigaut L, Marquez M, Alleva K, Pietrasanta LI, Amodeo G (2014) Heteromerization of PIP aquaporins affects their intrinsic permeability. Proceedings of the National Academy of Sciences 111: 231–236. 10.1073/pnas.1316537111 PMC3890845 24367080

[66] Beitz E, Wu B, Holm LM, Schultz JE, Zeuthen T (2006) Point mutations in the aromatic/arginine region in aquaporin 1 allow passage of urea, glycerol, ammonia, and protons. Proceedings of the National Academy of Sciences 103: 269–274. 10.1073/pnas.0507225103 PMC1326162 16407156

[67] Soto G, Alleva K, Mazzella MA, Amodeo G, Muschietti JP (2008) *AtTIP1;3* and *AtTIP5;1*, the only highly expressed *Arabidopsis* pollen-specific aquaporins, transport water and urea. FEBS Letters 582: 4077–4082. 10.1016/j.febslet.2008.11.002 19022253

[68] Jahn TP, Møller AL, Zeuthen T, Holm LM, Klærke DA, Mohsin B et al (2004) Aquaporin homologues in plants and mammals transport ammonia. FEBS Letters 574: 31–36. 15358535 10.1016/j.febslet.2004.08.004

[69] Mitani N, Yamaji N, Ma JF (2008) Characterization of substrate specificity of a rice silicon transporter, Lsi1. Pflügers Archiv-European Journal of Physiology 456: 679–686. 10.1007/s00424-007-0408-y 18214526

[70] Rougé P, Barre A (2008) A molecular modeling approach defines a new group of Nodulin 26-like aquaporins in plants. Biochemical and Biophysical Research Communications 367: 60–66. 18155659 10.1016/j.bbrc.2007.12.079

[71] Worth CL, Blundell TL (2010) On the evolutionary conservation of hydrogen bonds made by buried polar amino acids: the hidden joists, braces and trusses of protein architecture. BMC Evolutionary Biology 10: 161 10.1186/1471-2148-10-161 20513243 PMC2892493

[72] Johanson U, Gustavsson S (2002) A new subfamily of major intrinsic proteins in plants. Molecular Biology and Evolution 19: 456–461. 11919287 10.1093/oxfordjournals.molbev.a004101

