# Supplementary figures and images for: Genome-Wide Characterization of Major Intrinsic Proteins in Four Grass Plants and Their Non-Aqua Transport Selectivity Profiles with Comparative Perspective
Source: PLoS One. 2016 Jun 21;11(6):e0157735. doi: 10.1371/journal.pone.0157735 (PMC4915720; doi:10.1371/journal.pone.0157735)

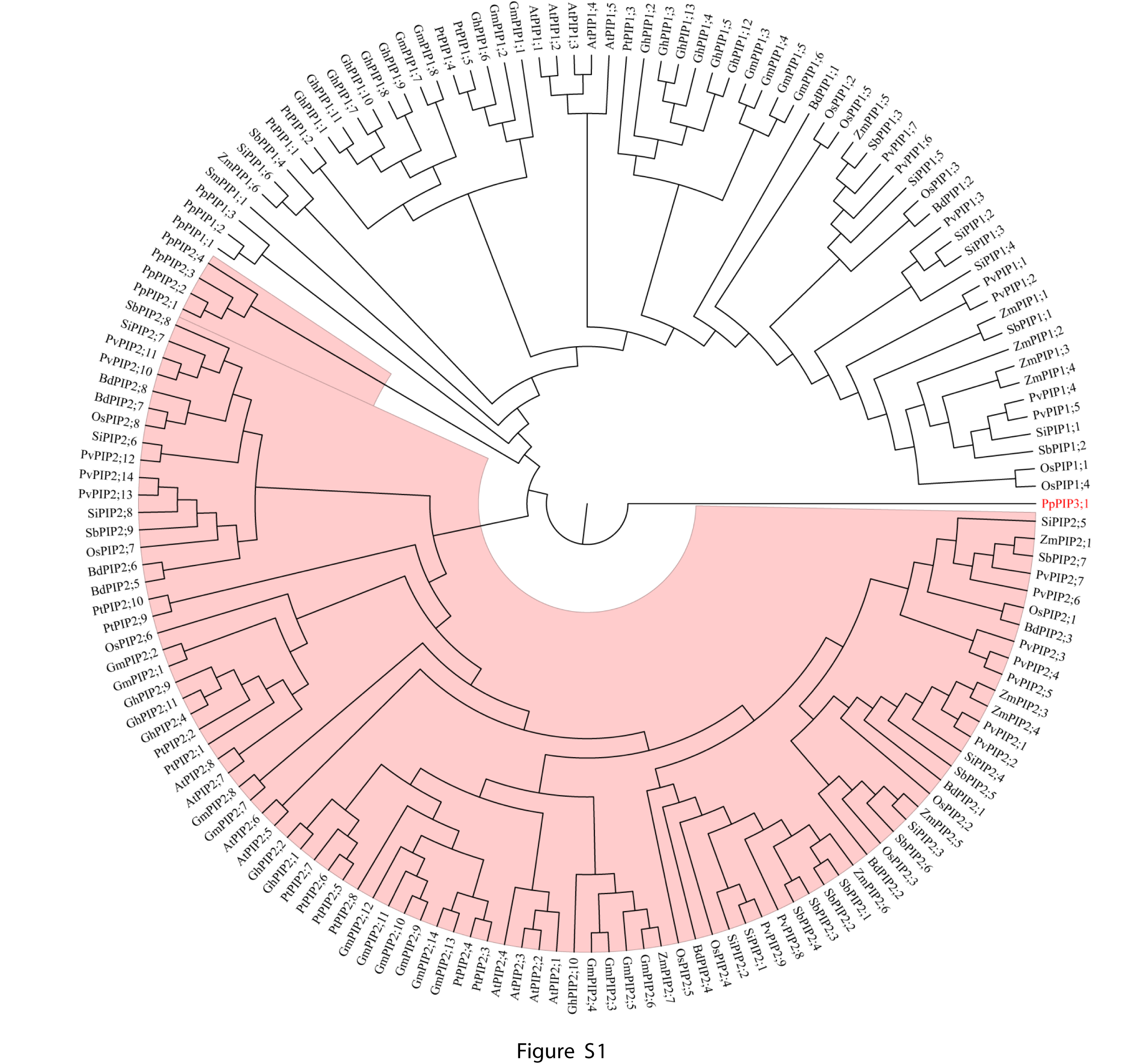

Supplement: S1 Fig — The description of figure legend is as for Fig 1. (TIF) [file pone.0157735.s001.tif]

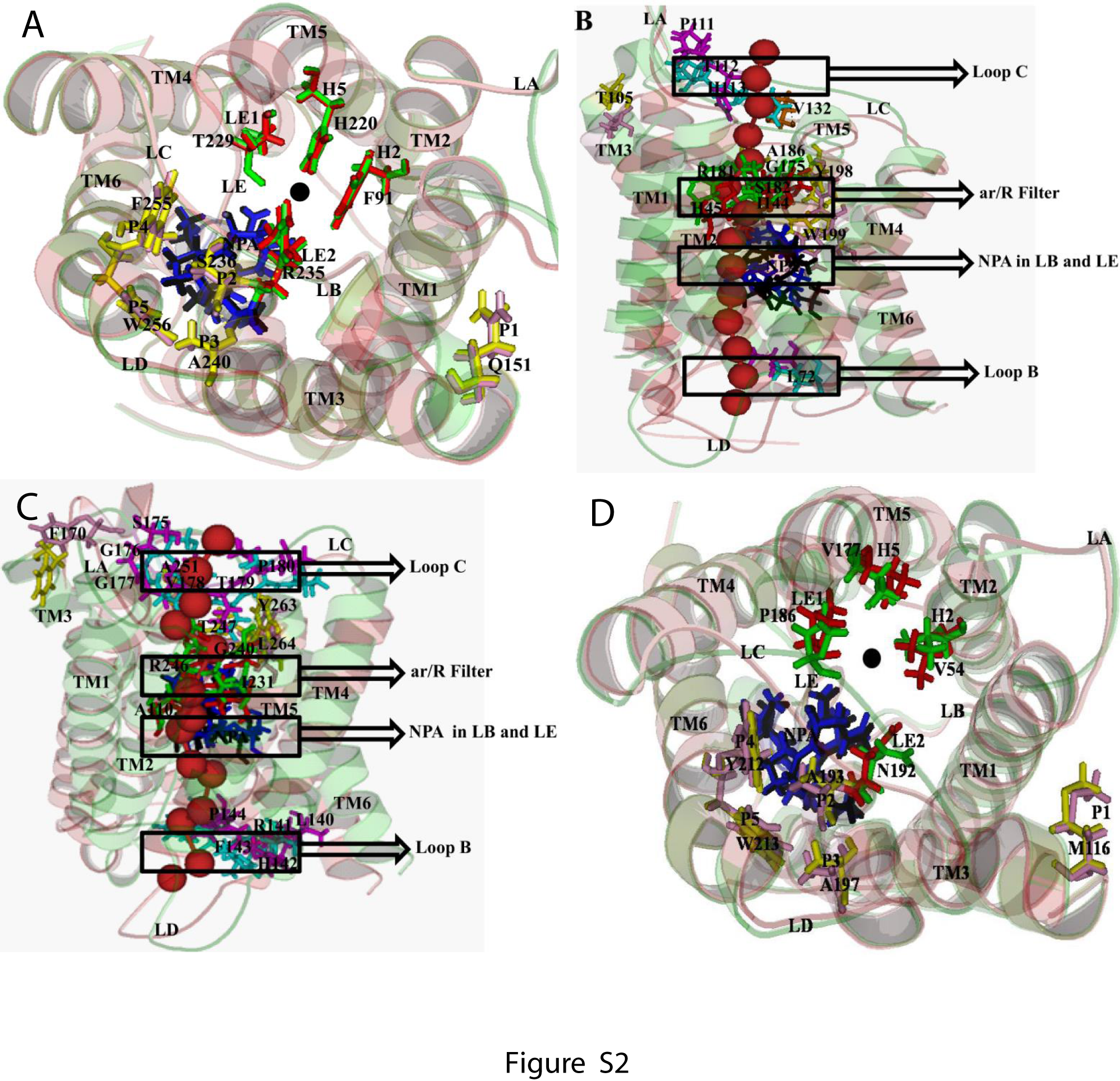

Supplement: S2 Fig — Homology models (green) of PvPIP2;1, PvTIP2;1, SiNIP3;5 and SiSIP1;1 superimposed with the models (red) of OsPIP2;1 (A), OsTIP2;1 (B), OsNIP3;1 (C) and OsSIP1;1 (D), respectively. A and D, the top views into the pore of PvPIP2;1 and SiSIP1;1, respectively, and B and C, the side views of PvTIP2;1 and SiNIP3;5, correspondingly. The 3D models of MIPs of the four grass plants were first constructed separately on the basis of the experimental structure of spinach PIP, SoPIP2;1(PDB ID:2B5F). Each of the 3D models of MIPs of the four grass plants was then superimposed on the MIP of other plants (only the representatives are shown). The residues that form the NPA box, ar/R filter and the FPs are shown as sticks. The residues of NPA, ar/R and FPs in PvPIP2;1, PvTIP2;1, SiNIP3;5 and SiSIP1;1 are shown in blue, green and yellow, respectively and those in OsMIPs are shown in black, red and pink, correspondingly and labeled. The TM α-helices and the loops to which they belong are indicated. The center of the pore is indicated as a black ball (A and D) and the path of the channel is indicated as the chain of red balls (B and C). The conserved pore-lining Leu in loop B and P-x-H in loop C found in predicted ammonia transporters TIP2s and TIP4s (B) and L-x-H-F-P in loop B and SGGVTVP found in predicted boron transporters NIP3s (C) are magenta; the same residues in the corresponding positions in OsTIP2;1 (B) and OsNIP3;1 (C) are cyan. The regions of NPA and ar/R selectivity filter and the conserved pore-lining residues in loops B and C in ammonia and boron transporters are boxed (B and C) and indicated by open arrows. The hydrogen bonding interaction between Pro and Val in loop C is shown by black dots. (TIF) [file pone.0157735.s002.tif]

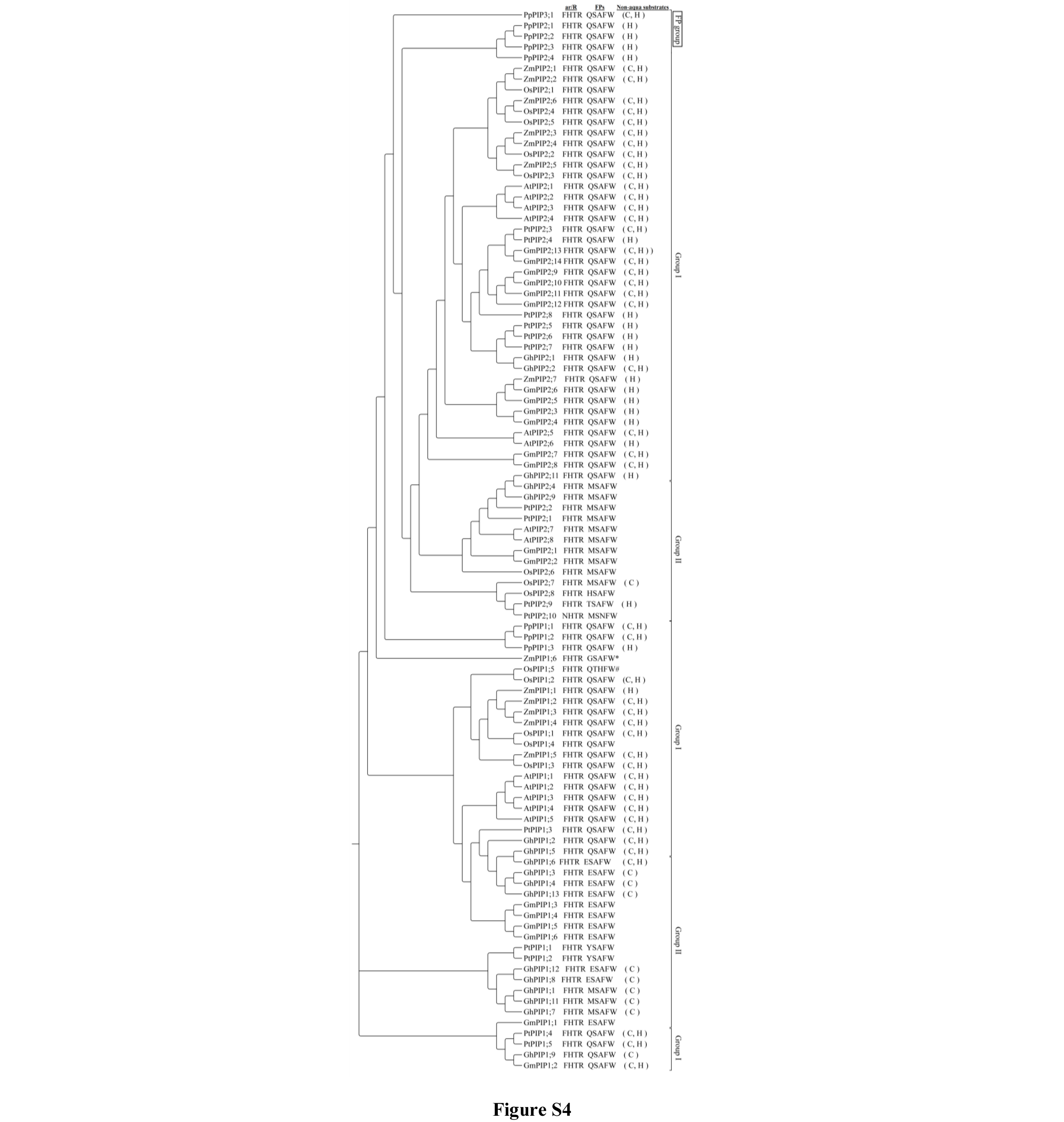

Supplement: S4 Fig — The description of the figure legend is as for Fig 3. Here, # and * indicate the members of group I and group II, respectively. (TIF) [file pone.0157735.s004.tif]

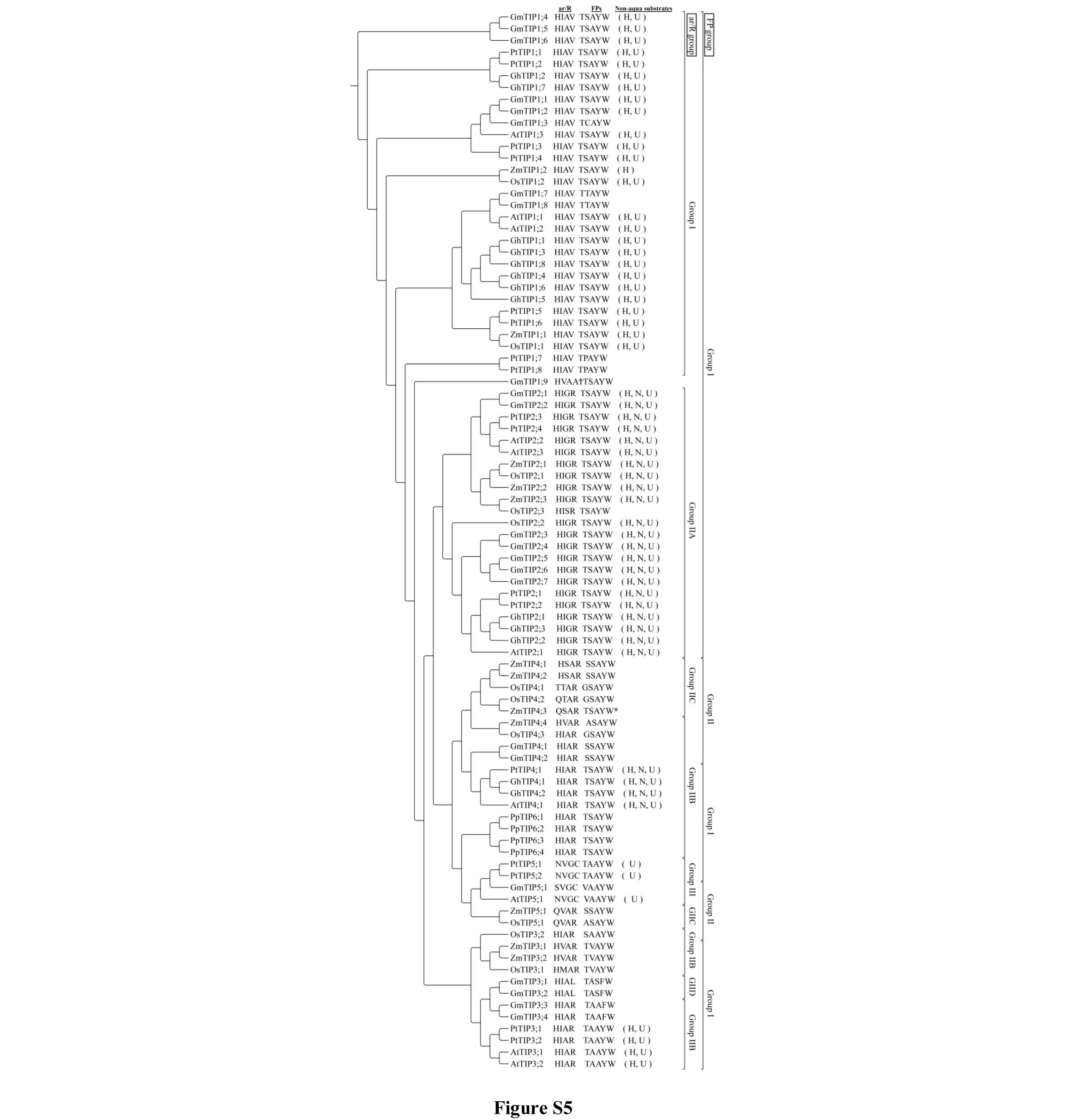

Supplement: S5 Fig — The description of the figure legend is as for Fig 3. Here, Ϯ and * indicate the members of group IIB of ar/R filter and group I of FPs, respectively. (TIF) [file pone.0157735.s005.tif]

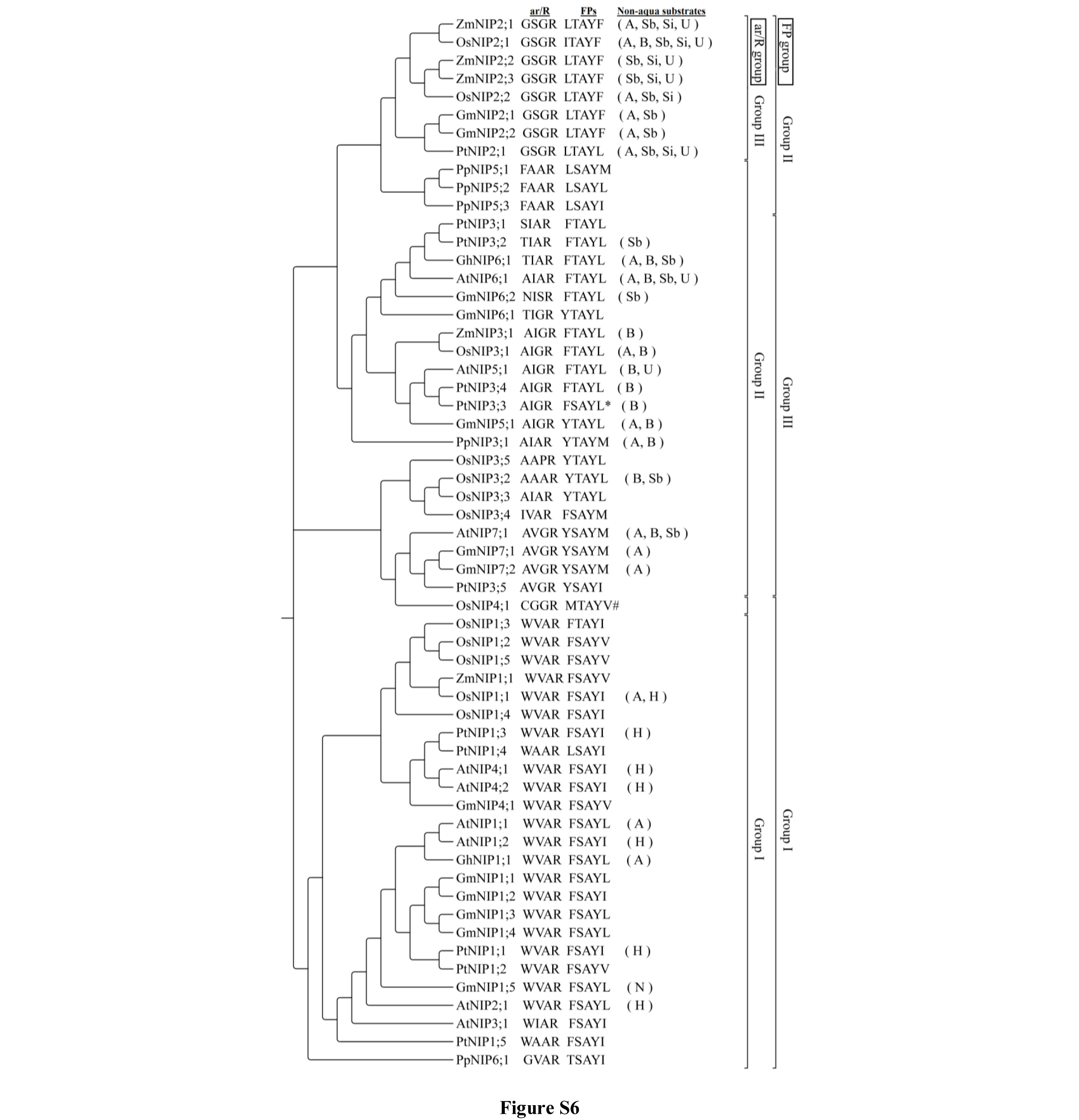

Supplement: S6 Fig — The description of the figure legend is as for Fig 3. Here, * and # indicates the members of group I of FPs and Group IV of ar/R filter, respectively. (TIF) [file pone.0157735.s006.tif]

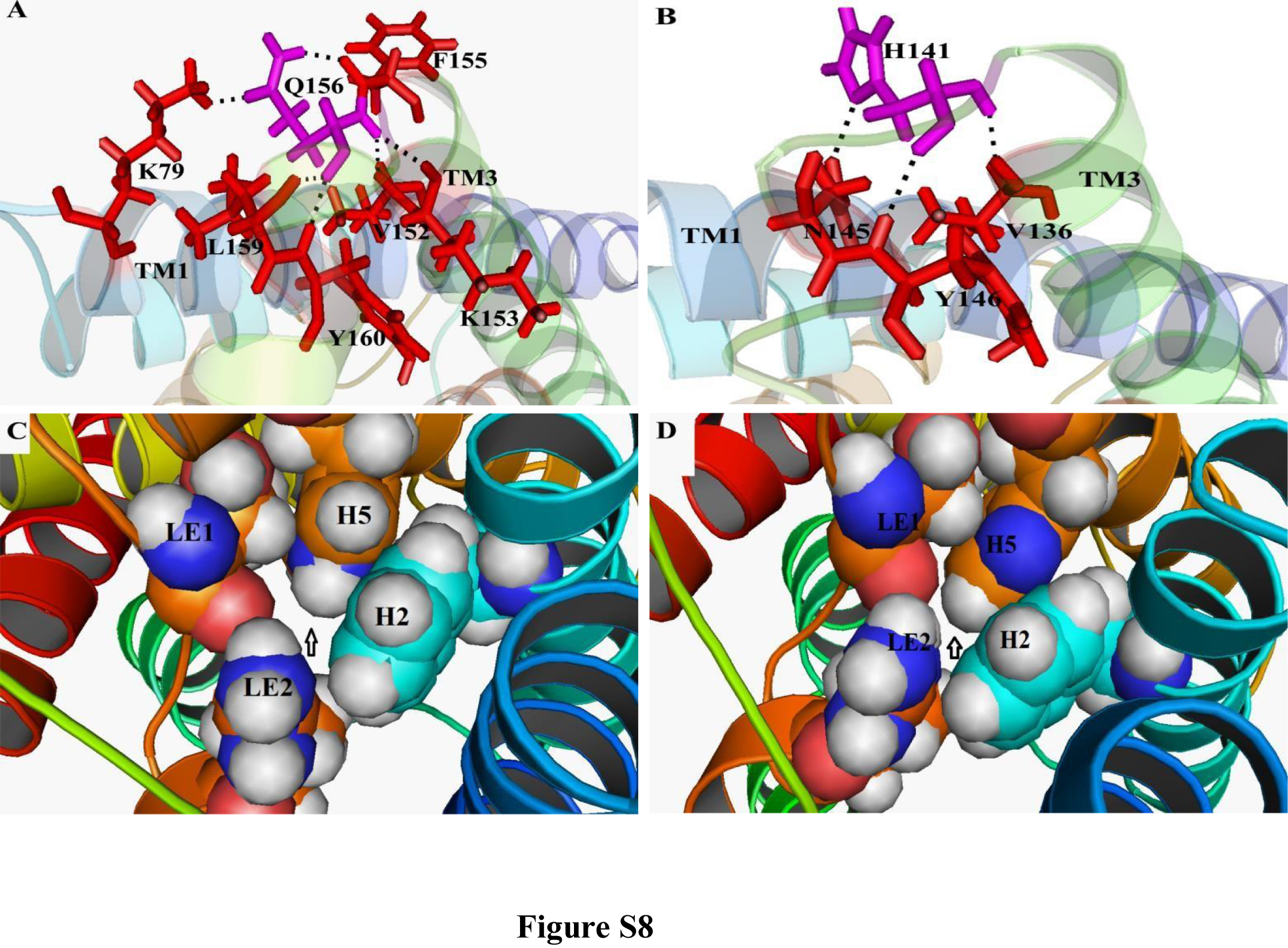

Supplement: S8 Fig — Intramolecular hydrogen-bonding interaction of the amino acid residue at the P1 position (A and B) and its possible role in pore conformation (C and D) in PIPs of Groups I and II. The Gln (Q) in P1 position of a Group I PIP is shown in magenta and its hydrogen bonding interactions with at least five amino acid residues are shown as black dashes (A). The hydrogen-bonding interaction of a substituted amino acid residue (magenta) at the corresponding position in a Group II PIP is shown as black dashes (B). The pore conformation (indicated by an open arrow) in the ar/R selectivity filter region (space-filling residues) of the same 3D models in (A) and (B) are shown in (C) and (D), respectively. (TIF) [file pone.0157735.s008.tif]
