# Supplementary material for: Genome-Wide Characterization of Major Intrinsic Proteins in Four Grass Plants and Their Non-Aqua Transport Selectivity Profiles with Comparative Perspective
Source: PLoS One. 2016 Jun 21;11(6):e0157735. doi: 10.1371/journal.pone.0157735 (PMC4915720; doi:10.1371/journal.pone.0157735)
Supplement: S3 Fig — The amino acid sequences were aligned using the Clustal Omega sequence alignment program. From the multiple alignment, only structurally significant regions containing the NPA motifs, tetrad residues of ar/R filter and FPs are shown. The two conserved NPA motifs are bold, the residues at H2, H5, LE1, and LE2 of the ar/R filter are bold and underlined, FPs (P1-P5) are italic and underlined, conserved residues are shaded with grey. (PDF) [file pone.0157735.s003.pdf]

```
                 H2                       P1        H5       LE1  LE2P2   P3           P4P5
PvPIP1;1    AWSFGGM..INPAV..AGVVKGFQQGL..FLVHLATIPITGTGINPARSLGAAIIYNRDHAW.IFWVGP
PvPIP1;2    AWSFGGM..INPAV..AGVVKGFQKGL..FLVHLATIPITGTGINPARSLGAAIIYNRDHAW.IFWVGP
PvPIP1;3    AWSFGGM..INPAV..AGVVKGFQQGL..FLVHLATIPITGTGINPARSLGAAIIYNRDHAW.VSEVD-
PvPIP1;4    AWSFGGM..INPAV..AGVVKGFQQGL..FLVHLATIPITGTGINPARSLGAAIIYNREHAW.IFWVGP
PvPIP1;5    AWSFGGM..INPAV..AGVVKGFQQGL..FLVHLATIPITGTGINPARSLGAAIIYNREHAW.IFWVGP
PvPIP1;6    AWSFGGM..INPAV..AGVVKGFQQGL..FLVHLATIPITGTGINPARSLGAAIIYNRSHAW.IFWVGP
PvPIP1;7    AWSFGGM..INPAV..AGVVKGFQQGL..FLVHLATIPITGTGINPARSLGAAIIYNRSHAW.IFWVGP
PvPIP2;1    AWAFGGM..INPAV..VGLVKGFQSAY..FMVHLATIPITGTGINPARSLGAAVIYNKDKAW.IFWVGP
PvPIP2;2    AWAFGGM..INPAV..VGLVKGFQSAY..FMVHLATIPITGTGINPARSLGAAVIYNKDKAW.Q---VR
PvPIP2;3    AWAFGGM..INPAV..VGLVKAFQSAY..FMVHLATIPVTGTGINPARSLGAAVIYNKDKPW.PTCIGA
PvPIP2;4    AWAFGGM..INPAV..VGLVKGFQSAY..FMVHLATIPITGTGINPARSFGAAVIYNEDKAW.Q---VR
PvPIP2;5    AWAFGGM..INPAV..VGLVKGFQSAY..FMVHLATIPITGTGINPARSFGAAVIYNNDKAW.H---VR
PvPIP2;6    AWAFGGM..INPAV..VGLVKAFQSAY..FMVHLATIPVTGTGINPARSLGAAVIYNKDKPW.IFWVGP
PvPIP2;7    AWAFGGM..INPAV..VGLVKAFQSAY..FMVHLATIPVTGTGINPARSLGAAVIYNKDKPW.IFWVGP
PvPIP2;8    AWAFGGM..INPAV..VALVKGFQSGF..FMVHLATIPITGTGINPARSLGAAVVYNNSKAW.IFWVGP
PvPIP2;9    AWAFGGM..INPAV..VALVKGFQSGF..FMVHLATIPVTGTGINPARSLGAAVVYNNSKAW.IFWVGP
PvPIP2;10   AWSFGGM..VNPAV..AGLVRAFHGGA..FMVHLATIPVTGTGINPARSLGPAVVYNQRKAW.VFWVGP
PvPIP2;11   AWSFGGM..VNPAV..AGLVRAFHGGP..FMVHLATIPVTGTGINPARSLGPAVVYNQRKAW.VFWVGP
PvPIP2;12   AWAFGGM..INPAG..AGLARAVHSSP..FVVHLATIPITGTGINPARSFGAAVVYNQARAW.IFRVGP
PvPIP2;13   SWSFGAT..INPAV..AGMVKGIVGGD..FVVHLATIPITGTGINPARSLGAAVMYN--KAW.IFWIGP
PvPIP2;14   AWSFGAT..INPAV..AGIVKGIVGGD..FVVHLATIPITGTGINPARSLGAAVVYD--MAW.IFWIGP

SiPIP1;1    AWSFGGM..INPAV..AGVVKGFQQG-..FLVHLATIPITGTGINPARSLGAAIIYNREHAW.IFWVGP
SiPIP1;2    AWSFGGM..INPAV..AGVVKGFQQG-..FLVHLATIPITGTGINPARSLGAAVIYNRRQAW.IFWVGP
SiPIP1;3    AWSFGGM..INPAV..AGVVKGFQQS-..FLVHLATIPITGTGINPARSLGAAIVYNRSQAW.IFWVGP
SiPIP1;4    AWAFGGM..INPAV..AGVVKALVGGA..FLVHLATIPITGTGINPARSLGAAIIYDRPHGW.IFWVGP
SiPIP2;1    AWAFGGM..INPAV..VALVKGFQS-G..FMVHLATIPITGTGINPARSLGAAVVYNNNKAW.IFWVGP
SiPIP2;2    AWAFGGM..INPAV..VALVKGFQS-G..FMVHLATIPITGTGINPARSLGAAVVYNNNKAW.IFWVGP
SiPIP2;3    AWAFGGM..INPAV..VGLVKGFQS-A..FMVHLATIPITGTGINPARSLGAAVIYNNDKAW.IFWVGP
SiPIP2;4    AWAFGGM..INPAV..VGLVKGFQS-A..FMVHLATIPITGTGINPARSLGAAVIYNNDKAW.IFWVGP
SiPIP2;5    AWAFGGM..INPAV..VGLVKAFQS-A..FMVHLATIPVTGTGINPARSLGAAVIYNKDKPW.IFWVGP
SiPIP2;6    AWAFGGM..INPAV..AGLVRAIHSPD..FVVHLATIPITGTGINPARSFGAAVVYNQARAW.IFWVGP
SiPIP2;7    AWSFGGM..VNPAV..AGLVRAFHGTS..FMVHLATIPVTGTGINPARSLGPAVVYNQRKAW.MFWVGP
SiPIP2;8    AWSFGAT..INPAV..VAIVKGITG-D..FVVHLATIPITGTGINPARSLGAAVIFG--EAW.IFWVGP

SbPIP1;1    AWSFGGM..INPAV..AGVVKGFQ-QG..FLVHLATIPITGTGINPARSLGAAVVYNQNHAW.IFWVGP
SbPIP1;2    AWSFGGM..INPAV..AGVVKGFQ-QG..FLVHLATIPITGTGINPARSLGAAIIYNRDHAW.IFWVGP
SbPIP1;3    AWSFGGM..INPAV..AGVVKGFQ-QT..FLVHLATIPITGTGINPARSLGAAIVYNRSNAW.IFWVGP
SbPIP1;4    AWAFGGM..INPAV..AGVVKALVGSA..FLVHLATIPITGTGINPARSLGAAIIYDSPHGW.IFWVGP
SbPIP2;1    AWAFGGM..INPAV..VALVKGFQSG-..FMVHLATIPITGTGINPARSLGAAVVYNNSKAW.IFWVGP
SbPIP2;2    AWAFGGM..INPAV..VALVKGFQSG-..FMVHLATIPITGTGINPARSLGAAVVYNNSKAW.IFWVGP
SbPIP2;3    AWAFGGM..INPAV..VALVKGFQSG-..FMVHLATIPITGTGINPARSLGAAVVYNNSKAW.IFWVGP
SbPIP2;4    AWAFGGM..INPAV..VALVKGFQSG-..FMVHLATIPITGTGINPARSLGAAVVYNNSKAW.IFWVGP
SbPIP2;5    AWSFGGM..INPAV..VGLVKGFQSA-..FMVHLATIPITGTGINPARSLGAAVIYNNDKAW.IFWVGP
SbPIP2;6    AWAFGGM..INPAV..VGLVKGFQSA-..FMVHLATIPITGTGINPARSLGAAVIYNNDKTW.IFWVGP
SbPIP2;7    AWAFGGM..INPAV..VGLVKAFQSA-..FMVHLATIPVTGTGINPARSLGAAVIYNKDKPW.IFWVGP
SbPIP2;8    AWSFGGM..INPAV..AGLVKAFHGAH..FMVHLATIPVTGTGINPARSLGPAVVYNQRKAW.IFWVGP
SbPIP2;9    AWSFGAT..INPAV..VGIVKGIMKV-..FVVHLATIPITGTGINPARSLGAAVVH---AAW.IFWVGP

BdPIP1;1    AWSFGGM..INPAV..AGVVKGFQS--..FLVHLATIPITGTGINPARSLGAAIIYNKSHNW.IFWVGP
BdPIP1;2    AWSFGGM..INPAV..AGVVKGFQT--..FLVHLATIPITGTGINPARSLGAAIIYNRKQAW.IFWVGP
BdPIP2;1    AWAFGGM..INPAV..VGLVKGFQS--..FMVHLATIPITGTGINPARSLGAAVIYNTDKAW.IFWVGP
BdPIP2;2    AWAFGGM..INPAV..VGLVKGFQS--..FMVHLATIPITGTGINPARSLGAAVIYNNDKAW.IFWVGP
BdPIP2;3    AWAFGGM..INPAV..VGLVKAFQS--..FMVHLATIPITGTGINPARSLGAAVIFNKDKAW.IFWVGP
BdPIP2;4    AWAFGGM..INPAV..VGLVKGFQR--..FMVHLATIPITGTGINPARSLGAAVVYNNDKAW.IFWVGP
BdPIP2;5    AWSFGAT..INPAV..AGLVKGIT--G..FVVHLATIPISGTGINPARSLGAAVMYNQHKAW.IFWVGP
BdPIP2;6    AWSFGAT..MNPAV..AAIVKAITRHA..FVVHLATIPVTGTGINPARSLGAAVMYNQHIAW.IFWVGP
BdPIP2;7    AWSFGGT..INPAV..AAIARSVHG--..FMVHLATIPITGTGINPARSLGAAVAYNGDKAW.IFWVGP
BdPIP2;8    AWAFGGS..INPAV..AGLVRTVNG--..FVVHLATIPVTGTSINPARSFGPAVVYNGEKAW.IFWVGP
```

```
                H2                    P1      H5      LE1    LE2P2 P3         P4P5
PvTIP1;1    AVAHAFA..VNPAV..CLLLRFSTGGL..GANILVGGAFDGASMNPAVSFGPALVSWSWTHQWVYWVGP
PvTIP1;2    AVAHAFA..VNPAV..CFLLRFSTGGL..GANILVGGAFDGASMNPAVSFGPALVSWSWTHQWVYWVGP
PvTIP1;3    SLAYALA..ANPAV..CLLLKIATGGA..GANILAGGAFDGAAMNPAVAFGPAVVTGVWENHWVYWLGS
PvTIP1;4    SLAYALA..ANPAV..CLLLKIATGGA..GANILAGGAFDGAAMNPAVAFGPAVVTGVWENHWVYWLGS
PvTIP2;1    AIAHAFA..LNPAV..CLLLQFVTHGK..GANILAAGPFSGGSMNPARSFGPAVAAGNFAGNWVYWVGP
PvTIP2;2    AIAHAFA..LNPAV..CLLLKFVTHGK..GANILAAGPFSGGSMNPARSFGPAVAAGNFAGNWVYWVGP
PvTIP2;3    AIAHALA..LNPAV..CLLLKFVTHGK..GANILAAGPFSGGSMNPARSFGPAVAAGNFAGNWVYWVGP
PvTIP2;4    AVCHGFG..VNPAV..AVLVQFST-GV..GANILVAGPFSGGSMNPARSFGPAVASGDFTNIWIYWVGP
PvTIP2;5    AVCHGFG..VNPAV..AVLVQFST-GV..GANILVAGPFSGGSMNPARSFGPAVASGDFTNIWIYWVGP
PvTIP3;1    ALAHALA..VNPAV..TLLLRLATGGA..GANVLAGGPFDGAGMNPARVFGPALVGWRWRHHWVYWLGP
PvTIP3;2    ALAHALA..VNPAV..TLLLRLATGGA..GANVLAGGPFDGAGMNPARVFGPALVGWRWRHHWVYWLGP
PvTIP3;3    AIAHALA..VNPAV..ALLLTLVSGGT..GANILAGGPFDGAAMNPARAFGPALVGWSWRHHWVYWVGP
PvTIP4;1    ALAHTLV..INPAV..CLLLAFLTGGA..GANVLAGGPFSGASMNPARSFGPALAAGVWADHWVYWVGP
PvTIP4;2    ALAHTLV..INPAV..CLLLAFLTGGA..GANVLAGGPFSGASMNPARSFGPALAAGVWADHWVYWVGP
PvTIP4;3    LLAHTLV..INPAV..CLLLAFLTGGA..GANVLAGGPFSGASMNPARSFGPALAAGVWADHWVYWVGP
PvTIP4;4    ALGQALV..VNPAV..CVLLRWLTGGQ..GANSIAGAALSGASMNPARSFGPAVATGVWTHHWVYWVGP
PvTIP4;5    AIAHALA..LNPAV..CILLRYLTGGM..GANSLAGGNFTGASMNPARSFGPALATGDWTHHWVYWLGP
PvTIP4;6    AIAHALA..LNPAV..CILLRYLTGGL..GANSLAGGNFTGASMNPARSFGPALATGDWTHHWVYWLGP
PvTIP5;1    AVAQAFG..ANPAV..CLVLHYISAGQ..GACVLSAGSLTGASMNPARSFGPAVVSGDYKNQAVYWAGP
PvTIP5;2    AVAQAFG..ANPAV..CLVLHYISAGQ..GACVLAAGSLTGASMNPARSFGPAVVSGDYKNQAVYWAGP

SiTIP1;1    ALAHALA..VNPAV..CLLLKIATGGE..GANILAGGAFDGASMNPAVSFGPAVVS.VWENHWVYWLGP
SiTIP1;2    AIAHAFA..VNPAV..CFLLRFSTGGL..GANILVGGAFDGASMNPAVSFGPALVS.SWGYQWVYWVGP
SiTIP2;1    AFTHALA..LNPAV..CLLLKFVTHGK..GANILAAGPFSGGSVNPARSFDPAVAA.NFAGNWVYWVGP
SiTIP2;2    AIAHALA..LNPAV..CLLLKFVTHGK..GANILAAGPFSGGSMNPARSFGPAVAA.NFAGNWVYWVGP
SiTIP2;3    AIAHAFA..LNPAV..CLLLKFVTHGQ..GANILAAGPFSGGSMNPARSFGPAVAA.NFAGNWVYWVGP
SiTIP2;4    AVCHGFG..VNPAV..AVLVQYSTGVV..GANILVAGPFSGGSMNPARSFGPAVAS.DFTNIWIYWVGP
SiTIP2;5    AVCHGFG..VNPAV..AVLVQYSTGVA..GANILVAGPFSGGSMNPARSFGPAVAS.DFTNIWIYWVGP
SiTIP3;1    ALAHALA..VNPAI..ALLLRLATGG-..GANVLACGALDGAVMNPARAFGPAIVG.RWSNHWVYWAGP
SiTIP3;2    ALAHALA..VNPAI..SLLLRLATGG-..GANVLAGGPFDGAGMNPARVFGPALVG.RWRHHWVYWLGP
SiTIP4;1    ALGQALV..VNPAV..CILLRWLTGG-..GANSIAGAVLSGASMNPARSFGPAVAT.VWTHHWVYWVGP
SiTIP4;2    ALGQALA..VNPAV..CVLLRWLTGGL..GANSIAGAVLSGASMNPARSFGPALAT.VWTHHWVYWVGP
SiTIP4;3    ALAHTLV..INPAV..CLLLAFLAGG-..GANVLAGGPFSGASMNPARSFGPALAA.VWADHWVYWVGP
SiTIP4;4    AITNALA..LNPAV..CILLRYLTGG-..GANSLAGGNFTGASMNPARSFGPALAT.DWTHHWVYWLGP
SiTIP5;1    AVAQAFA..ANPAV..CLVVHILSAGQ..GACALATGSLTGASMNPARSFGPAVVS.DFRNQAVYWAGP
SiTIP5;2    AVAQAFG..ANPAV..CL---------..GALVLSAGPLTGASMNPARSFGPAVVS.NYKNQAVYWAGP

SbTIP1;1    AVAHAFA..VNPAV..CFLLRFSTG....GANILVGGAFTGASMNPAVSFGPALVS.EWGYQWVYWVGP
SbTIP1;2    SLAHALA..VNPAV..CILLKMATG....GANILAGGAFDGASMNPAVSFGPAVVS.VWENHWVYWLGP
SbTIP2;1    AIAHALA..LNPAV..CLLLKFVTH....GANILAAGPFSGGSMNPARSFGPAVAA.NFAGNWVYWVGP
SbTIP2;2    AIAHAFA..LNPAV..CLLLKYVTH....GANILAAGPFSGGSMNPARSFGPAVAA.NFAGNWVYWVGP
SbTIP2;3    AVCHGFG..VNPAV..AVLVQYST-....GANILVAGPFSGGSMNPARSFGPAVAS.DFTNIWIYWVGP
SbTIP3;1    ALAHALA..VNPAV..TLLLRLATG....GANVLAGGPFDGAGMNPARVFGPALVG.RWRHHWVYWLGP
SbTIP3;2    AIAHALA..VNPAV..SLLLTLVSG....GANILAGGPFDGAAMNPARAFGPALVG.SWRHHWVYWVGP
SbTIP3;3    ALAHALA..VNPAV..ALVLRLATG....GANVLACGALEGAVMNPARAFGPAVVG.RWGNHWVYWVGP
SbTIP4;1    ALAHTLV..INPAV..CLLLAFLTAGG..GANVLAGGPFSGASMNPARSFGPALVA.VWADHWVYWVGP
SbTIP4;2    AIAHALA..LNPAV..CILLRYLS--...GANSLAGGNFSGASMNPARSFGPALAS.VWTNHWIYWIGP
SbTIP4;3    ALGQALV..VNPAV..CFLLRWLT--G..GANSIAGAVLSGASMNPARSFGPAVAT.VWTHHWVYWVGP

BdTIP1;1    AIAHAFA..VNPAV..CFLLRFSTG-..VGANILVGGAFDGASMNPAVSFGPALVS.EWGYQWVYWVGP
BdTIP1;2    ALAHAFA..VNPAV..CLLLQISTG-..VGANILAGGAFDGASMNPAVSFGPAVVS.VWENHWVYWLGP
BdTIP2;1    AIAHAFA..LNPAV..CFLLKFVTH-..VGANILAAGPFSGGSMNPARSFGPAVAA.NFAGNWVYWVGP
BdTIP2;2    AIAHAFA..LNPAV..CLLLQFVTH-..VGANILAAGPFSGGSMNPARSFGPAVAA.DFSGHWVYWVGP
BdTIP3;1    ALAHALA..VNPAI..SLLLRLTTG-..LGANMLAGGPFDGAGMNPARVFGPALVG.RWGHHWVYWVGP
BdTIP3;2    AVAHALA..VNPAV..ALLLSLVSG-..LGANILTGGPFDGAAMNPARAFGPALVG.NWSHHWVYWVGP
BdTIP4;1    ALAHALV..MNPAV..CLLLRALTG-..VGANTIAGGALTGASMNPARSFGPALAS.DWANHWVYWVGP
BdTIP4;2    ALAQALA..LNPAV..CILLRYLTG-..VGANTLAGGNFSGASMNPARSFGPALAT.VWTNHWVYWVGP
BdTIP4;3    ASAHAMA..INPAV..CLLLSFLSGS..VGANALAGGPFSGASMNPARSFGPALVT.EWAGHWVYWVGP
BdTIP5;1    AVAQSFG..VNPAV..CLVLHYFSA-..AGACVLAAGSLTGASMNPARSFGPAVVS.DFKNQAVYWVGP
```

```
                 H2                   P1       H5        LE1  LE2P2 P3         P4P5
PvNIP1;1    CIVWGLA..LNPAV..SLTLRLLFG--..LLNVLFAGPISGASMNPARTLGPAIVAGRYAGIWVYFAGP
PvNIP1;2    CIVWGLA..LNPAV..SLTLRLLFG--..LLNVLFAGPISGASMNPARTLGPAIVAGRYAGIWVYFAGP
PvNIP1;3    CAVWGLV..FNPAV..SLTLRVVFGG-..LLNVLFAGPITGASMNPARTLGPAIVAGRYRSVWVYVVGP
PvNIP1;4    CAVWGLV..FNPAV..SLTLRVVFGG-..LLNVLFAGPITGASMNPARTLGPAIVAGRYRSIWVYVVGP
PvNIP1;5    AIVWGLA..FNPAV..AGTLRLMFG--..LLNVLIAGPVSGASMNPARSVGPALVSGQYRSIWVYLVGP
PvNIP1;6    AVVWGMT..INPAV..SLMLRLMFG--..MLNALFAGPVSGASMNPARSIGPALVGSKYRALWVYIFGP
PvNIP1;7    AVVWGMT..INPAV..SLMLRLMFG--..MLNALFAGPVSGASMNPARSIGPALVGSKYTALWVYIFGP
PvNIP2;1    SVAGGLI..MNPAV..SFVLKAVL---..CITSIFAGAVSGGSMNPARTLGPALASNLYTGLWIYFLGP
PvNIP2;2    SVAGGLI..MNPAV..-----------..CITSIFAGAVSGGSMNPARTLGPALASNLYTGLWIYFLGP
PvNIP2;3    SVAGGLI..MNPAV..AFVLKAVL---..CITSIFAGPVSGGSMNPARTLAPAVASNVYTGLWIYFLGP
PvNIP2;4    SVAGGLI..MNPAV..AFVLKAVL---..CITSIFAGPVSGGSMNPARTLAPAVASNVYTGLWIYFLGP
PvNIP3;1    AAAAGLA..MNPAV..SFAAKALYGVG..MMNALVFAKLTGASMNPARTLGPAIATGTYTKIWVYMVAP
PvNIP3;2    AAAAGLA..MNPAV..SFTARALY--G..TRNALVFAKWTGASMNPARTLGAAIATGTYTKIWVYMVAP
PvNIP3;3    AAAAGLA..INPAV..SFAAKALY--G..TMNALVFARSTGASMNPARTLGPAIATGTYTKIWVYMVAP
PvNIP3;4    AVAAGSA..LNPAV..SFAARAVY---..MMGALISAETTGASMNPARTLGPAIAAGTYTKIWIYMVAP
PvNIP3;5    ATSAGLA..LNPAV..SFSVKGIY---..MMNALVAGPSTGASMNPARTLGPAVATGRYTQIWIYMVAT
PvNIP3;6    ATSAGLA..LNPAV..SFSVKGIY---..MMNALVAGPSTGASMNPARTIGPAVATGRYTQIWIYMVAT
PvNIP3;7    AASAGLA..LNPAV..TFLAKGMY---..TMNALVGGPSTGPSMNPARTIGAALATGKYKDIWVYLVAP
PvNIP3;8    AASAGLA..LNPAV..AFLAKGMY---..TMNALVGGPSTGPSMNPARTIGAALATGKYKDIWVYLVAP
PvNIP3;9    AACAGLA..LNPSL..SFALKGVF---..TLNILVAGTTTGGSMNPVRTLGPAVAAGNYRQLWIYLLAP
PvNIP3;10   AACAGLA..LNPSL..SFALKGVF---..TLNILVAGPTTGGSMNPVRTLGPAVAAGNYRQLWIYLLAP
PvNIP4;1    PMVCLVV..FNPAV..CLAVNGVMR--..GTLGLVIGPVSGGSMNPIRTLGPAIVLGRYTSVWIYLVAP
PvNIP4;2    PMVCLVV..FNPAV..CLAVNGVMR--..GTLGLVIGPVSGGSMNPIRTLGPAIVLGRYTSVWIYLVAP

SiNIP1;1    AIVWGLA..FNPAV..SGTLRLMFG--..LLNVLIAGPVSGASMNPARSVGPALVSGQYRSIWVYVVGP
SiNIP1;2    CIVWGLA..LNPAV..SLTLRLLFG--..LLNVLFAGPISGASMNPARTLGPAIVAGRYAGIWVYFAGP
SiNIP1;3    AVVWGMT..INPAV..SLLLRQMFG--..MLNALFAGPVSGASMNPVRSIGPALVGGKYTGLWVYIFGP
SiNIP2;1    SVAGGLI..MNPAV..AFVLKAVL---..-------GPVSGGSMNPARTLAPAVASNVYTGLWIYFLGP
SiNIP2;2    SVAGGLI..MNPAV..AFVLKAVL---..CITSIFAGPVSGGSMNPARTLAPAVASNVYTGLWIYFLGP
SiNIP2;3    SVAGGLI..MNPAV..SFVLKAVL---..CITSIFAGAVSGGSMNPARTLGPALASNLYTGLWIYFLGP
SiNIP3;1    ATSAGLA..LNPAV..SFSVKGIYH-P..MMNALVAGPSTGASMNPARTLGPAIATGRYTQIWIYMVAT
SiNIP3;2    AAVAGSA..LNPAV..SFAAKAVYNNP..MMSALISGESTGASMNPARTLGPAIAAGTYTKIWVYMVAP
SiNIP3;3    AAAAGMA..MNPAV..SFAAKALYD-P..VMSALVSGKWTGASMNPARTLGPAIATGTYAKIWVYMVAP
SiNIP3;4    AAAAGLA..LNPAV..TFLAKGMYR-P..TMNALVGASWTGPSMNPARTIGAAVATGKYKDIWVYLVAP
SiNIP3;5    AACAGLA..LNPSL..SFALKGVFHPF..TLNILVAGPTTGGSMNPVRTLGPAVAAGNYRQLWIYLLAP
SbNIP4;1    CLVCGLT..FNPAV..CVAANGVMK--..GTLGLVIGPVSGGSMNPIRTLGPAIVL..YTSVWIYLVAP

SbNIP1;1    CAVWGLV..FNPAV..SLTLRVVFGGA..LLNVLVAGPITGASMNPARTLGPAIVA..YRSIWVYMVGP
SbNIP1;2    CIVWGLA..LNPAV..SLTLRLLFG--..LLNVLFAGPVSGASMNPARTLGPAIVV..YAGIWVYFAGP
SbNIP1;3    AIVWGLA..FNPAV..SGTLRLMFG--..LLNVLIAGPVSGASMNPARSVGPALVS..YRSIWVYVVGP
SbNIP1;4    ALVWGMT..INPAV..SLVLRLMFGGE..ILNALFAGPVSGASMNPARSIGPALVG..YTSLWVYIFGP
SbNIP2;1    SVAGGLI..MNPAV..SFVLKAVLH--..CITSIFAGAVSGGSMNPARTLGPALAS..YTGLWIYFLGP
SbNIP2;2    SVAGGLI..MNPAV..AFVLKAVLH--..CITSIFAGPVSGGSMNPARTLAPAVAS..FTGLWIYFLGP
SbNIP3;1    ATSAGLA..LNPAV..SFTVKGIYHPV..MMNALVAGPSTEASMNPARTLGPAIAT..YTQIWIYMVAT
SbNIP3;2    AATAGMA..LNPAV..SFVAKALYDPV..MMSALISGESTGASMNPARTLGTAIAT..YTKIWIYVVAP
SbNIP3;3    AASAGLA..LNPAV..TFLAKAMYRPA..SVHAVLCRPSTGPSMNPARTIAAALAT..YKDIWVYLLAP
SbNIP3;4    AACAGLA..LNPSL..SFALKGVFHPF..TLNILVAGPTTGGSMNPVRTLGPAVAA..YRQLWIYLLAP
SbNIP4;1    CLVVALT..FNPAV..CVAANGVMK--..GTLGLVIGPVSGGSMNPIRTLGPAIVL..YTSVWIYLVAP

BdNIP1;1    AIVWGLA..FNPAV...GTLRLMFGGR..LLNVLIAG..SGASMNPARTVGPALVG..YRSIWVYVVGP
BdNIP1;2    CITWGLA..FNPAV...LTLRLLFG--..LLNVLFAG..SGASMNPARTLGPAMVA..YKGIWVYIVGP
BdNIP1;3    TMVWGLA..MNPAV...VVLRLMFGGR..TLNALFSG..TGASMNPARSIGPALVG..YTSLWVYILGP
BdNIP2;1    SVAGGLI..MNPAV...FVLKAVLHP-..CITSIFAG..SGGSMNPARTLGPALAS..YTGLWLYFLGP
BdNIP2;2    SLVGGLI..MNPAV...FVLRAVLHP-..CITSIFAG..SGGSMNPARTLAPAVAS..YSGLWIYFLGP
BdNIP3;1    AACAGLA..LNPSL...FALKGVFHP-..TLNILIAG..TGGSMNPVRTLGPAVAA..YRQLWIYLVAP
BdNIP4;1    PMVCLVV..LNPAV...LAVNALMRPR..GTLGLVIG..SGGSMNPVRSLGPAIVM..YESVWIYLVAP
```

```
              H2                  P1       H5      LE1  LE2P2 P3          P4P5
PvSIP1;1   VSLLSVL..FNPTGV......ELMPA....CLVLSGAAYTGPSMNPANAFGWAYVN..WEQFYVYWICP
PvSIP1;2   VSLLSAL..FNPTGV......ELMPA....CLVLSGAAYTGPSMNPANAFGWAYVN..WEQFYVYWICP
SiSIP1;1   VSLVSVL..FNPTGI......ELMPA....CLVLSGAAYTGPSMNPANAFGWAYVN..WEQFYVYWISP
SiSIP1;2   VSLLSVL..FNPTGI......ELMPA....CLILSGAAYTGPSMNPANAFGWAYVN..WEQFYVYWISP
SbSIP1;1   ASLLAVL..FNPTDF......ELMPA....SLIVAGAEYTGPSMNPANAFGWAYVN..WEQLYVYWICP
BdSIP1;1   VSLLSLL..FNPTAL......ELMPA....SLVLAGAAYTGPSMNPANAFGWAYVN..WEQLYVYWICP
PvSIP2;1   VSLSIVY..YNPLTI......IQFTF....TIHILSSDITGGIMNPASAFAWAYAR..FDHLLVYWLAP
PvSIP2;2   VSLSIVY..YNPLTI......IQFTF....TIHILSSDITGGIMNPASAFAWAYAR..FDHLLVYWLAP
SiSIP2;1   VSLSLVY..YNPLTV......IQFTF....TIHILSSDITGGIMNPASAFAWAYAR..FDHLLVYWLAP
SbSIP2;1   VSLSIVY..YNPLTV......IQLTF....TIHILSSDITGGIMNPASAFAWAYAR..FDHLLVYWLAP
```

Figure S3
