# Supplementary material for: Genome-Wide Characterization of Major Intrinsic Proteins in Four Grass Plants and Their Non-Aqua Transport Selectivity Profiles with Comparative Perspective
Source: PLoS One. 2016 Jun 21;11(6):e0157735. doi: 10.1371/journal.pone.0157735 (PMC4915720; doi:10.1371/journal.pone.0157735)
Supplement: S7 Fig — Multiple sequence alignment of groups I (A) and II (B) PIPs of the twelve plants. The amino acid sequences were aligned using the Clustal Omega program. The transmembrane helices and the dual NPA motifs are shown as gray and yellow, respectively. The residue (Q) at P1 position is shown as cyan. The pore-lining residues are indicated by arrows above the alignment and the conserved residues are indicated by stars (*) at the bottom of the alignment. (PDF) [file pone.0157735.s007.pdf]

Figure S7 (A)

```
PtPIP2;3      --------------------------------MAKDMEVAEAGS--------FSAKDYHD 20
PtPIP2;4      --------------------------------MAKDTEVAEAGS--------FSAKDYQD 20
GmPIP2;13     -----------------------MSVFWQEGGMAKDVEVAERGS--------FSGKDYQD 29
GmPIP2;14     --------------------------------MAKDVEVAERGS--------FSGKDYQD 20
GmPIP2;9      --------------------------------MAK--HDVEGGS--------FSAKDYHD 18
GmPIP2;10     --------------------------------MAK--HDVEGGS--------FSAKDYHD 18
GmPIP2;11     --------------------------------MAK--HDVEGGS--------FAAKDYHD 18
GmPIP2;12     --------------------------------MAK--HDVEGGS--------FSAKDYHD 18
PtPIP2;8      --------------------------------MAKDIEVAEHG---------ETVKDYQD 19
PtPIP2;5      --------------------------------MGK--DIEVGGE--------FSAKDYHD 18
PtPIP2;6      --------------------------------MGK--DVEVRGE--------FIAKDYHD 18
PtPIP2;7      --------------------------------MGK--DIEVGGE--------FSAKDYHD 18
GhPIP2;1      --------------------------------MAK--DVEVGGE--------FQAKDYHD 18
GhPIP2;2      --------------------------------MAK--DIEVGGE--------FQAKDYHD 18
ZmPIP2;7      --------------------------------MAKDVEQVTEQG-------EYSAKDYHD 21
GmPIP2;6      --------------------------------MAKDVEQVTEQG-------EYSAKDYHD 21
GmPIP2;5      --------------------------------MAKDVEQVTEQG-------EYSAKDYHD 21
GmPIP2;3      --------------------------------MAKDVEVQEQGG-------EYSAKDYHD 21
GmPIP2;4      --------------------------------MAKDVEVQEQGG-------EYSAKDYHD 21
GhPIP2;10     --------------------------------MTKDIETTAEQGGG----AEFSAKDYQD 24
ZmPIP2;1      --------------------------------MGKDDVIESGAGGG-----EFAAKDYTD 23
SbPIP2;7      --------------------------------MGKDDVIESGAGGG-----EFAAKDYTD 23
PvPIP2;6      --------------------------------MGKDDVIEGGAGGG-----EFAAKDYTD 23
PvPIP2;7      --------------------------------MGKDDVIESGAGGG-----EFAAKDYTD 23
ZmPIP2;2      --------------------------------MGKDDVVQSGAGGG-----EFAAKDYTD 23
SiPIP2;5      --------------------------------MGKDDVIESGAGGG-----EFAAKDYTD 23
OsPIP2;1      --------------------------------MGKDEVMESGGAAG-----EFAAKDYTD 23
BdPIP2;3      --------------------------------MAKDEVMESGGGHQ-----DFAAKDYTD 23
ZmPIP2;3      --------------------------------MAKQDIEASGPEAG-----EFSAKDYTD 23
ZmPIP2;4      --------------------------------MAK-DIEASGPEAG-----EFSAKDYTD 22
PvPIP2;1      --------------------------------MAK-DIEASGPEAG-----EFSAKDYTD 22
SiPIP2;4      --------------------------------MAK-DIEASGPEAG-----EFSAKDYSD 22
SbPIP2;5      --------------------------------MAK-DIEASGPEAG-----EFSAKDYTD 22
OsPIP2;2      --------------------------------MAK-DIEASAPEGG-----EFSAKDYTD 22
ZmPIP2;5      --------------------------------MAK-DIEAAAAHEG---------KDYSD 18
SbPIP2;6      --------------------------------MAK-DIEAAAAAHGG--GGEYTAKDYSD 25
SiPIP2;3      --------------------------------MAK-DIEAAAAPEG----GEYTAKDYSD 23
OsPIP2;3      --------------------------------MAK-DIEAAAAAEG----GEYMAKDYSD 23
BdPIP2;2      --------------------------------MAK-DIEAAPGEYA--------AKDYSD 19
BdPIP2;1      --------------------------------MAK-DIEASAPEGG-----EFSAKDYSD 22
PvPIP2;2      --------------------------------MAKD-IEASGPEAG-----EFSAKDYTD 22
```

```
PvPIP2;3    --------------------------------MGKDDVIESGAGGG-----EFAAKDYTD 23
PvPIP2;4    --------------------------------MAKDIEAAAA--GG-----EYTAKDYSD 21
PvPIP2;5    --------------------------------MAKDIEAAAAPEGG-----EYTAKDYSD 23
SbPIP2;1    --------------------------------MGK-EVDVSTLEA-----GGVR--DYAD 20
SbPIP2;2    --------------------------------MGK-EVDVSALEA-----GGVR--DYAD 20
ZmPIP2;6    --------------------------------MGK-EVDVSTLEA-----GGVRDRDYAD 22
SbPIP2;3    --------------------------------MAK-EVDVSALEA-----GGAR--DYID 20
SbPIP2;4    --------------------------------MAK-EVDVSTLEA-----GGAR--DYID 20
PvPIP2;8    --------------------------------MGK-EVDVSTLEA-----GGAR--DYAD 20
PvPIP2;9    --------------------------------MGK-EVDVSTLEA-----GGAR--DYAD 20
SiPIP2;1    --------------------------------MGK-EVDVSTLEA-----GGAR--DYAD 20
SiPIP2;2    --------------------------------MGK-EVDVSALEA-----GGAR--DYVD 20
OsPIP2;4    --------------------------------MGK-EVDVSTLEA-----GGAR--DYID 20
BdPIP2;4    --------------------------------MGK-EVDVASLEAG----GGAR--DYSD 21
OsPIP2;5    --------------------------------MGK-EADV---EA-----GGVR--DYED 17
AtPIP2;2    --------------------------------MAKDVEGP----------EGFQTRDYED 18
AtPIP2;3    --------------------------------MAKDVEGP----------DGFQTRDYED 18
AtPIP2;1    --------------------------------MAKDVEAVPG--------EGFQTRDYQD 20
AtPIP2;4    --------------------------------MAKDLDVNES--------GPPAARDYKD 20
GmPIP2;7    --------------------------------MAKDIETEVQSG--------LPHKDYHD 20
GmPIP2;8    --------------------------------MAKDLETEIQSG--------LPHKDYHD 20
AtPIP2;5    --------------------------------MTKEVVGDKRS---------FSGKDYQD 19
AtPIP2;6    --------------------------------MTKDELTEEES---------LSGKDYLD 19
PpPIP2;2    --------------------------------MAKD-VGVEPG---------FPSKDYTD 18
PpPIP2;3    --------------------------------MSKVPVGVEPG---------FPGKDYAD 19
PpPIP2;1    --------------------------------MAKD-AGTESG---------VPSKDYSD 18
PpPIP2;4    --------------------------------MEKIGICEEPK---------FRSKDYID 19
SmPIP2;1    --------------------------------MAKDASKESEAFV--------TAKDYEE 20
SmPIP3;1    --------------------------------MSKDLENGN------------AAKDFSE 16
PpPIP3;1    --------------------------------MPTDNEFR----------------DTHE 12
PpPIP1;1    ----------------MNQDKDDDIALGTNKYGDRSALGTHAPVP---------EKDYTE 35
PpPIP1;2    ----------------MQQDKDDDVALGANKYGTRSALGTHAPVP---------EKDYRE 35
PpPIP1;3    -----------------MADRGDDVAVGASRH-ERNPLGTSAQTR---------EKDYIE 33
SmPIP1;1    -----------------MEGNREDVHGVAKYPERE-LGTSAQA-----------EKDYVE 32
ZmPIP1;1    -----------------MEGKEEDVRLGANKFSERHAIGTAAQGTD--------DKDYKE 35
SbPIP1;1    -----------------MEGKEEDVRLGANKYSERQPIGTAAQGTD--------DKDYKE 35
ZmPIP1;3    -----------------MEGKEEDVRLGANKFSERQPIGTAAQGAGAGD----DDKDYKE 39
ZmPIP1;4    -----------------MEGKEEDVRLGANKFSERQPIGTAAQGAGAGD----DDKDYKE 39
SbPIP1;2    -----------------MEGKEEDVRLGANKFSERQPIGTAAQGAG-------DDKDYKE 36
ZmPIP1;2    -----------------MEGKEEDVRLGANKFSERQPIGTAAQGAA-------DDKDYKE 36
PvPIP1;1    -----------------MEGKEEDVRLGANKFSERQPIGTAAQGTD--------DKDYKE 35
PvPIP1;2    -----------------MEGKEEDVRLGANKFSERQPIGTAAQGTD--------DKDYKE 35
OsPIP1;1    -----------------MEGKEEDVRLGANRYSERQPIGTAAQGAG-------DDKDYKE 36
PvPIP1;4    -----------------MEGKEEDVRLGANKFSERQPIGTAAQGAG-------DDKDYKE 36
SiPIP1;1    -----------------MEGKEEDVRLGANKFSERQPIGTAAQGAG-------DDKDYKE 36
```

```
PvPIP1;5    -----------------MEGKEEDVRLGANKFSERQPIGTAAQGAG-------DDKDYKE 36
SiPIP1;4    -----------------MEGKEEDVRLGANKYSERQPIGTAAQGSD--------DKDYKE 35
SiPIP1;2    -----------------MEGKEEDVRLGANKFSERQPIGTAAQGAG-------DDKDYKE 36
SiPIP1;3    -----------------MEGKEEDVRLGANKYSERQPIGTAAQGS--------DDKDYKE 35
PvPIP1;3    -----------------MEGKEEDVRLGANKFSERQPIGTAAQGT--------DDKDYKE 35
OsPIP1;4    -----------------MEGKEEDLRLGANRFSERQPIGTAAQGAG-------DNKDYKE 36
OsPIP1;2    -----------------MEGKEEDVRLGANKFSERQPIGTAAQGSD--------DKDYKE 35
OsPIP1;5    -----------------MEGKEEDVRLGANKFSERQPIGTAAQGSD--------DKDYKE 35
BdPIP1;1    -----------------MEGKEEDVRLGANKFSERQPIGTAAQGSE--------DKDYKE 35
ZmPIP1;5    -----------------MEGKEEDVRLGANRYSERQPIGTAAQGTEE--------KDYKE 35
PvPIP1;7    -----------------MEGKEEDVRLGANRYSERQPIGTAAQGTEE--------KDYKE 35
PvPIP1;6    -----------------MEGKEEDVRLGANRYSERQPIGTAAQGSDD--------KDYKE 35
SiPIP1;5    -----------------MEGKEEDVRLGANRYSERQPIGTAAQGSDD--------KDYKE 35
SbPIP1;3    -----------------MEGKEEDVRLGANRYSERQPIGTAAQGTEEN------SKDYKE 37
OsPIP1;3    -----------------MEGKEEDVRLGANRYTERQPIGTAAQGAEE--------KDYRE 35
BdPIP1;2    -----------------MEGKEEDVRLGANRYSERQPIGTAAQGGDSE-------KDYKE 36
GhPIP1;2    -----------------MEGKEEDVKLGANKFSERQPIGTSAQTD----------KDYKE 33
GhPIP1;5    -----------------MEGKEEDVKLGANKFSERQPIGTSAQTD----------KDYKE 33
PtPIP1;3    -----------------MEGKEEDVKLGANKFSERQPIGTSAQTD----------KDYKE 33
AtPIP1;1    -----------------MEGKEEDVRVGANKFPERQPIGTSAQ-SD---------KDYKE 33
AtPIP1;2    -----------------MEGKEEDVRVGANKFPERQPIGTSAQ-SD---------KDYKE 33
AtPIP1;3    -----------------MEGKEEDVRVGANKFPERQPIGTSAQ-TD---------KDYKE 33
AtPIP1;4    -----------------MEGKEEDVRVGANKFPERQPIGTSAQSTD---------KDYKE 34
AtPIP1;5    -----------------MEGKEEDVNVGANKFPERQPIGTAAQTES---------KDYKE 34
PtPIP1;5    -----------------MEGREEDVRVGANKYGERQPIGTAAQAQDV--------KDYTD 35
GhPIP1;6    -----------------MEGQGEDVRLGANKYRERQPIGTAAQTQDT--------KDYKE 35
GmPIP1;2    LRLPHSHIHIEVKLALKMEGRDEDVRVGANRYGERQPIGTAAQTQDA--------KDYRE 52
PtPIP1;4    -----------------MEEGEEDVKVGANRYGEGQPIGTAAQTQHG--------KDYTE 35
                                                                  *

                            TM1

PtPIP2;3    PPPAPLFDAKELTKWSFYRALIAEFIATLLFLYITVLTVIGYKSQID--G--S--ADSCG 74
PtPIP2;4    PPPAPLIDAEELTKWSFYRALIAEFIATMLFLYITVLTVIGYKSQID--G--N--ADPCG 74
GmPIP2;13   PPPAPLIDAEELTKWSFYRALIAEFIATLLFLYITVLTVIGYNHQTD--LKEN--GEICG 85
GmPIP2;14   PPPAPLIDAEELTKWSFYRALIAEFIATLLFLYITVLTVIGYKHQTD--H-----ADACG 73
GmPIP2;9    PPPAPLIDAEELTQWSFYRALIAEFIATLLFLYITVLTVIGYKSQSD--VKAG--GDVCG 74
GmPIP2;10   PPPAPLIDAEELTQWSFYRALIAEFIATMLFLYITVLTVIGYKSQSD--VKAG--GDVCG 74
GmPIP2;11   PPPAPLIDAEELTQWSFYRALIAEFIATMLFLYITVLTVIGYKSQSD--VKAG--GDVCG 74
GmPIP2;12   PPPAPLIDAEELTQWSFYRALIAEFIATLLFLYITVLTVIGYKSQSD--VKAG--GDVCG 74
PtPIP2;8    PPPAPLIDAEELGQWSFYRALIAEFIATLLFLYVTVLTVIGYKSQTD--PDKG--LDACG 75
PtPIP2;5    PPPAPLIDAEELTQWSLYRAIIAEFIATLLFLYITVLTVIGYKSQTD--TTKN--SDACG 74
PtPIP2;6    PPPAPLIDAEELTQWSLYRAIIAEFIATLLFLYITVLTVIGYKSQTD--TTKN--SDACG 74
```

TM1

```
PtPIP2;7      PPPAPLIDAEEITQWSFYRAIIAEFVATLLFLYITVLTVIGYKSQTD--VNKN--GDECG 74
GhPIP2;1      PPPAPLVDAQELTQWSFYRAIIAEFIAALLFLYITVLTVIGYKSQVD--PDKG--GQDCD 74
GhPIP2;2      PPPAPLVDAQELAKWSFYRAVIAEFIATLLFLYITVLTVIGYKTQTD--PAKG--GEDCG 74
ZmPIP2;7      PPPAPLIDPDELTKWSLYRAAIAEFIATLLFLYITVLTIIGYKRQSD-TKIPG--NTECD 78
GmPIP2;6      PPPAPLIDPDELTKWSLYRAAIAEFIATLLFLYITVLTIIGYKRQSD-TKIPG--NTECD 78
GmPIP2;5      PPPAPLIDPDELTKWSLYRAAIAEFIATLLFLYITVLTIIGYKRQSD-TKIPG--NTECD 78
GmPIP2;3      PPPAPLFDPEELTQWSFYRALIAEFIATLLFLYVTVLTIIGYKRQTD-ATLGG---TECD 77
GmPIP2;4      PPPAPLFDPEELTQWSFYRALIAEFIATLLFLYVTVLTIIGYKRQTD-TTVGG---TDCD 77
GhPIP2;10     PPPAPLIDLEELTKWSLYRAAIAEFIATLLFLYVTVLTVIGYKVQTDPVKNTV--DPDCG 82
ZmPIP2;1      PPPAPLIDAAELGSWSLYRAVIAEFIATLLFLYITVATVIGYKHQTDASAS--GADAACG 81
SbPIP2;7      PPPAPLIDAAELGSWSLYRAVIAEFIATLLFLYITVATVIGYKHQTDATAS--GADAACG 81
PvPIP2;6      PPPAPLIDAAELGSWSLYRAMIAEFIATLLFLYITVATVIGYKHQTDAAAS--GADAACG 81
PvPIP2;7      PPPAPLVDAAELCSWSLYRAVIAEFIATLLFLYITVATVIGYKHQTDAAAS--GADAACG 81
ZmPIP2;2      PPPAPLVDAAELGSWSLYRAVIAEFIATLLFLYVTVATVIGYKHQTDASASGAGADAACG 83
SiPIP2;5      PPPAPLIDAAELGSWSLYRAVIAEFIATLLFLYITVLTVIGYKHQTDPNVA---GTDACG 80
OsPIP2;1      PPPAPLIDAAELGSWSLYRAVIAEFIATLLFLYITVATVIGYKHQTDASAS--GADAACG 81
BdPIP2;3      PPPAPLIDAAELASWSLYRAVIAEFIATLLFLYITVATVIGYKHQTDVSAS--GPDAACG 81
ZmPIP2;3      PPPAPLIDADELTKWSLYRAVIAEFIATLLFLYITVATVIGYKHQTDAAASGP--DAACG 81
ZmPIP2;4      PPPAPLIDAEELTQWSLYRAVIAEFIATLLFLYITVATVIGYKHQTDASASGP--DAACG 80
PvPIP2;1      PPPAPLIDAEELTKWSLYRAVIAEFIATLLFLYITVATVIGYKHQTDAAASGP--DAACG 80
SiPIP2;4      PPPAPLIDAEELTKWSLYRAAIAEFVATLLFLYITVATVIGYKHQTDAAASGP--DAACG 80
SbPIP2;5      PPPAPLIDAAELTQWSLYRAVIAEFIATLLFLYITVATVIGYKHQTDATASGP--DAACG 80
OsPIP2;2      PPPAPLIDVEELTKWSLYRAVIAEFIATLLFLYITVATVIGYKHQSDATVNTT--DAACS 80
ZmPIP2;5      PPPAPLVDAEELTKWSLYRAVIAEFVATLLFLYITVATVIGYKHQTDAAASGP--DAACG 76
SbPIP2;6      PPPAPLVDAEELTKWSLYRAVIAEFVATLLFLYITVATVIGYKHQTDASSSGP--DAACG 83
SiPIP2;3      PPPAPLIDAEELTKWSLYRAVIAEFVATLLFLYITVATVIGYKHQTDAAASGP--DAACG 81
OsPIP2-3      PPPAPLIDAEELTKWSLYRAVIAEFVATLLFLYITVATVIGYKHQSDPGANAA--DAACS 81
BdPIP2;2      PPPAPLFDAEELTKWSLYRAAIAEFVATLLFLYITVATVIGYKHQADASASGA--DAACG 77
BdPIP2;1      PPPAPIVDFEELTKWSLYRAAIAEFVATLLFLYITVATVIGYKHQTDVSASGP--DAACG 80
PvPIP2;2      PPPAPLIDAEELTKWSLYRAVIAEFIATLLFLYITVATVIGYKHQTDAAASGP--DAACG 80
PvPIP2;3      PPPAPLVDAAELCSWSLYRAVIAEFIATLLFLYITVATVIGYKHQTDAAASGA--DAACG 81
PvPIP2;4      PPPAPLIDAEELTKWSLYRAVIAEFVATLLFLYITVATVIGYKHQTDAAASGP--DAACG 79
PvPIP2;5      PPPAPLIDAEELTKWSLYRAVIAEFVATLLFLYITVATVIGYKHQTDATASGP--DAACG 81
SbPIP2;1      PPPAPLIDIDELGKWSLYRAVIAEFVATLLFLYITVATVIGYKHQTDATASGA--DAACG 78
SbPIP2;2      PPPVPLIDIDELGKWSLYRAVIAEFVATLLFLYITVATVIGYKHQTDATASGA--DAACG 78
ZmPIP2;6      PPPAPLIDIDELGKWSLYRAVIAEFVATLLFLYITVATVIGYKHQTDASASGP--DAACS 80
SbPIP2;3      PPPAPLVDIHELGKWSLYRAVIAEFVATLLFLYITVATVIGYKHQTDATASGA--DAACS 78
SbPIP2;4      PPPAPLVDIDELGKWSLYRAVIAEFVATLLFLYITVATVIGYKHQTDATASGA--DAACG 78
PvPIP2;8      PPPAPLVDIEELGKWSLYRAVIAEFVATLLFLYITVATVIGYKHQTDAAASGP--DAACG 78
PvPIP2;9      PPPAPLVDIDELCKWSLYRAVIAEFVATLLFLYITVATVIGYKHQTDASASGP--DAACG 78
SiPIP2;1      PPPAPLVDIDELGKWSLYRAVIAEFVATLLFLYITVATVIGYKHQTDASASGP--DAACG 78
SiPIP2;2      PPPAPLVDIDELGKWSLYRAVIAEFVATLLFLYITVATVIGYKHQTDASASGP--DAACG 78
OsPIP2;4      PPPAPLVDVDELGKWSLYRALIAEFVATLLFLYVTVATVIGYKHQTDAAVNGA--DAACG 78
BdPIP2;4      PPPAPLIDIDELGKWSLYRAVIAEFVATLLFLYITVATVIGYKHQTDASAPGAGADAACG 81
```

```
                TM1
OsPIP2;5   PPPAPLVDIDELGRWSLYRAVIAEFVATLLFLYVTVATVIGYKHQTDASASGD--DAACG 75
AtPIP2;2   PPPTPFFDADELTKWSLYRAVIAEFVATLLFLYITVLTVIGYKIQSDTKAG----GVDCG 74
AtPIP2;3   PPPTPFFDAEELTKWSLYRAVIAEFVATLLFLYVTVLTVIGYKIQSDTKAG----GVDCG 74
AtPIP2;1   PPPAPFIDGAELKKWSFYRAVIAEFVATLLFLYITVLTVIGYKIQSDTDAG----GVDCG 76
AtPIP2;4   PPPAPFFDMEELRKWPLYRAVIAEFVATLLFLYVSILTVIGYKAQTDATAG----GVDCG 76
GmPIP2;7   PPAAAFYDPAELRKWSFYRALIAEFVATLLFLYVTILTVIGYNHQTATGSP-----DLCN 75
GmPIP2;8   PPPAPFYDPAELRKWSFFRALIAEFVATLLFLYVTILTVIGYNHQTAT-AA-----EPCS 74
AtPIP2;5   PPPEPLFDATELGKWSFYRALIAEFIATLLFLYVTIMTVIGYKSQTDPALN----PDQCT 75
AtPIP2;6   PPPVKTFEVRELKKWSFYRAVIAEFIATLLFLYVTVLTVIGFKSQTDINAG----GGACA 75
PpPIP2;2   PPPAPLIDASEFGQWSFYRAVIAEFVATLLFLYITIATVIGAVRNAG-----------CD 67
PpPIP2;3   PPAAPLIDASEFGQWSFYRAIIAEFVATLLFLYITIATVIGASRNAG-----------CA 68
PpPIP2;1   PPPAPLIDAAEFGRWSFYRAIIAEFVATLLFLYITISTVIGASRNAG-----------CA 67
PpPIP2;4   PPAVPFVDASELRKWSFYRAIITEFISTLLFLYIAIGTVVGASRNAD-----------CA 68
SmPIP2;1   PPPARLVDPKEFGSWSFYRAGIAEFVATLLFLYITVQTVIGHSRNAAN----------CG 70
SmPIP3;1   PPPTPLIDLAELKSWSLYRACIVELVATLLFLYIGVTALIGHARAQAAAGGDS-----CG 71
PpPIP3;1   PPPAPILARDEFNEWSFYRAIIAEFIATLLFLYVSLTTLMGTTRIFG------------G 60
PpPIP1;1   PSVTPFFDGSEFRRWSFWRAGIAEFIATLLFLYITIQTVMGHKRS----------ADPCL 85
PpPIP1;2   PSVTPFFDGGELRLWSFWRAGIAEFFATLLFLYITIQTVMGHVRN----------TDPCL 85
PpPIP1;3   PASSPFIDPVELGRWSFWRAGIAEFFASFLFLYITVQTVMGHNR-----------GDACA 82
SmPIP1;1   PPPTRLIEPSEFSSWSFWRAGIAEFFATFLFLYITILTVVGNVD-----------RTSCL 81
ZmPIP1;1   PPPAPLFEPGELKSWSFYRPGIAEFVATFLFLYISILTVMGVSKS----------TSKCA 85
SbPIP1;1   PPPAPLFEPGELKSWSFYRAGIAEFVATFLFLYISILTVMGVSKS----------TSKCA 85
ZmPIP1;3   PPPAPLFEPGELKSWSFYRAGIAEFVATFLFLYITVLTVMGVSKS----------TSKCA 89
ZmPIP1;4   PPPAPLFEPGELKSWSFYRAGIAEFVATFLFLYITVLTVMGVSKS----------TSKCA 89
SbPIP1;2   PPPAPLFEPGELKSWSFYRAGIAEFVATFLFLYITILTVMGVSKS----------NSKCA 86
ZmPIP1;2   PPPAPLFEPGELKSWSFYRAGIAEFVATFLFLYITILTVMGVSKS----------TSKCA 86
PvPIP1;1   PPPAPLFEPGELKSWSFYRAGIAEFVATFLFLYISILTVMGVSKS----------QSKCA 85
PvPIP1;2   PPPAPLFEPGELKSWSFYRAGIAEFVATFLFLYISILTVMGVSNS----------QSKCA 85
OsPIP1;1   PPPAPLFEPGELKSWSFYRAGIAEFVATFLFLYITILTVMGVSKS----------SSKCA 86
PvPIP1;4   PPPAPLFEPGELKSWSFYRAGIAEFVATFLFLYITILTVMGVSKS----------NSKCA 86
SiPIP1;1   PPPAPLFEPGELKSWSFYRAGIAEFVATFLFLYITILTVMGVSKS----------NSKCA 86
PvPIP1;5   PPPAPLFEPGELKSWSFYRAGIAEFVATFLFLYITILTVMGVSKS----------NSKCA 86
SiPIP1;4   PPPAPLFEPGELKSWSFYRAGIAEFVATFLFLYISILTVMGVSKS----------QSKCA 85
SiPIP1;2   PPPAPLFEPGELKSWSFYRAGIAEFVATFLFLYITILTVMGVSKS----------NSKCA 86
SiPIP1;3   PPPAPLFEPGELKSWSFYRAGIAEFVATFLFLYISILTVMGVSKS----------QSKCA 85
PvPIP1;3   PPPAPLFEPGELKSWSFYRAGIAEFVATFLFLYISILTVMGVSKS----------QSKCA 85
OsPIP1;4   PPPAPLFEPGELKSWSFYRAGIADFVATFLFLYITILTVMGVSKS----------SSKCA 86
OsPIP1;2   PPPAPLFEPGELKSWSFYRAGIAEFMATFLFLYITVLTVMGVNNS----------TSKCA 85
OsPIP1;5   PPPAPLFEPGELKSWSFYRAGIAEFMATFLFLYITVLTVMGVNNS----------TSKCA 85
BdPIP1;1   PPPAPLFEPGELKSWSFYRAGIAEFMATFLFLYVTILTVMGVNNS----------PSKCA 85
ZmPIP1;5   PPPAPLFEAEELTSWSFYRAGIAEFVATFLFLYISILTVMGVSKS----------SSKCA 85
PvPIP1;7   PPPAPLFEAEELTSWSFYRAGIAEFVATFLFLYISILTVMGVSKS----------SSKCA 85
PvPIP1;6   PPPAPLFEAEELASWSFYRAGIAEFVATFLFLYISILTVMGVSKS----------SSKCA 85
SiPIP1;5   PPPAPLFEAEELTSWSFYRAGIAEFVATFLFLYISILTVMGVSKS----------PSKCA 85
```

```
                                 TM1
SbPIP1;3      PPAAPLFEAEELTSWSLYRAGIAEFVATFLFLYISILTVMGVSKS----------GSKCG 87
OsPIP1;3      PPAAPVFEVEELTSWSFYRAGIAEFVATFLFLYISILTVMGVNKS----------ASKCA 85
BdPIP1;2      PPPAPIFEAEELTSWSFYRAGIAEFIATFLFLYISVLTVMGVSSS----------SSKCG 86
GhPIP1;2      PPAAPFFEPGELKSWSFYRAGIAEFVATFLFLYITVLTVMGVSQS----------KTKCA 83
GhPIP1;5      PPAAPFFEPGELKSWSFYRAGIAEFVATFLFLYITVLTVMGVSQS----------KTKCA 83
PtPIP1;3      APPAPLFEPGELKSWSFYRAGIAEFIATFLFLYITVLTVMGVTKPG---------TSKCS 84
AtPIP1;1      PPPAPFFEPGELSSWSFWRAGIAEFIATFLFLYITVLTVMGVKRS----------PNMCA 83
AtPIP1;2      PPPAPLFEPGELASWSFWRAGIAEFIATFLFLYITVLTVMGVKRS----------PNMCA 83
AtPIP1;3      PPPAPFFEPGELSSWSFYRAGIAEFIATFLFLYITVLTVMGVKRA----------PNMCA 83
AtPIP1;4      PPPAPLFEPGELSSWSFYRAGIAEFIATFLFLYITVLTVMGVKRA----------PNMCA 84
AtPIP1;5      PPPAPFFEPGELKSWSFYRAGIAEFIATFLFLYVTVLTVMGVKRA----------PNMCA 84
PtPIP1;5      PPPAPLFEPGELSSWSFYRAGIAEFVATFLFLYITVLTVMGVAKS----------PTKCS 85
GhPIP1;6      PPAAPLFEPAELSSWSFYRAGIAEFVATFLFLYITVLTVMGVAKS----------STKCS 85
GmPIP1;2      APPAPLFEPRELTSWSFYRAGIAEFVATFLFLYVTVLTVMGVAKS----------PSKCS 102
PtPIP1;4      PPPAPLYQPGEWLSWSFYRAGIAEFVATFLFLYITVLTVMGVARS----------STKCS 85
              ..         *   *.::*. *.::.:::****: : :::*

                   TM2                                  TM3
PtPIP2;3      GVGILGIAWAFGGMIFVLVYCTAGISGGHINPAVTFGLFLARKVSLIRAVMYMVAQCLGA 134
PtPIP2;4      GVGILGIAWAFGGMIFVLVYCTAGISGGHINPAVTFGLFLARKVSLIRAVMYMVAQCAGA 134
GmPIP2;13     GVGILGIAWAFGGMIFILVYCTAGISGGHINPAVTFGLFLARKVSLIRAIMYMVAQCLGA 145
GmPIP2;14     GVGILGIAWAFGGMIFILVYCTAGISGGHINPAVTFGLFLARKVSLIRAIMYMVAQCLGA 133
GmPIP2;9      GVGILGIAWAFGGMIFILVYCTAGISGGHINPAVTFGLFLARKVSLIRAIMYMVAQCLGA 134
GmPIP2;10     GVGILGIAWAFGGMIFILVYCTAGISGGHINPAVTFGLFLARKVSLIRAIMYMVAQCLGA 134
GmPIP2;11     GVGILGIAWAFGGMIFILVYCTAGISGGHINPAVTFGLFLARKVSLIRAIMYMVAQCLGA 134
GmPIP2;12     GVGILGIAWAFGGMIFILVYCTAGISGGHINPAVTFGLFLARKVSLIRAIMYMVAQCLGA 134
PtPIP2;8      GVGILGIAWAFGGMIFVLVYCTAGISGGHINPAVTFGLFLARKVSLIRAVLYMVAQCLGA 135
PtPIP2;5      GVGILGIAWAFGGMIFVLVYCTAGISGGHINPAVTFGLFLARKVSLVRAVLYMVAQCLGA 134
PtPIP2;6      GVGILGIAWAFGGMIFVLVYCTAGISGGHINPAVTFGLFLARKVSLVRAVLYMVAQCLGA 134
PtPIP2;7      GVGILGIAWAFGGMIFILVYCTAGISGGHINPAVTFGLFLARKVSLVRAILYMVAQCLGA 134
GhPIP2;1      GVGILGIAWAFGGMIFILVYCTAGISGGHINPAVTFGLFLARKVSLVRAILYMAAQCLGA 134
GhPIP2;2      GVGILGIAWAFGGMIFILVYCTAGISGGHINPAVTFGLFLARKVSLVRAIFYMGAQCLGA 134
ZmPIP2;7      GVGILGIAWAFGGMIFILVYCTAGISGGHINPAVTFGLFLGRKVSLVRALLYMIAQCAGA 138
GmPIP2;6      GVGILGIAWAFGGMIFILVYCTAGISGGHINPAVTFGLFLGRKVSLVRALLYMIAQCAGA 138
GmPIP2;5      GVGILGIAWAFGGMIFILVYCTAGISGGHINPAVTFGLFLGRKVSLVRALLYMIAQCAGA 138
GmPIP2;3      GVGILGIAWAFGGMIFILVYCTAGISGGHINPAVTFGLFLGRKVSLIRALLYMVAQCAGA 137
GmPIP2;4      GVGILGIAWAFGGMIFILVYCTAGISGGHINPAVTFGLFLGRKVSLIRALLYMVAQCAGA 137
GhPIP2;10     GVGILGIAWAFGGMIFILVYCTAGISGGHINPAVTFGLFVGRKVSLIRALLYMVAQCLGA 142
ZmPIP2;1      GVGVLGIAWAFGGMIFVLVYCTAGISGGHINPAVTFGLFLARKVSLVRALLYIVAQCLGA 141
SbPIP2;7      GVGVLGIAWAFGGMIFVLVYCTAGISGGHINPAVTFGLFLARKVSLVRALLYIVAQCLGA 141
PvPIP2;6      GVGVLGIAWAFGGMIFVLVYCTAGISGGHINPAVTFGLFLARKVSLVRALLYMVAQCLGA 141
PvPIP2;7      GVGVLGIAWAFGGMIFVLVYCTAGISGGHINPAVTFGLFLARKVSLVRALLYIVAQCLGA 141
```

```
                TM2                                     TM3
ZmPIP2;2   GVGVLGIAWAFGGMIFVLVYCTAGISGGHINPAVTFGLFLARKVSLVRALLYMVAQCLGA 143
SiPIP2;5   GVGILGIAWAFGGMIFVLVYCTAGISGGHINPAVTFGLFLARKVSLVRALLYIVAQCLGA 140
OsPIP2;1   GVGVLGIAWAFGGMIFILVYCTAGISGGHINPAVTFGLFLARKVSLVRAILYIVAQCLGA 141
BdPIP2;3   GVGILGIAWAFGGMIFVLVYCTAGISGGHINPAVTFGLFLARKVSLVRALLYMIAQCLGA 141
ZmPIP2;3   GVGILGIAWAFGGMIFILVYCTAGISGGHINPAVTFGLFLARKVSLVRALLYIIAQCLGA 141
ZmPIP2;4   GVGILGIAWAFGGMIFILVYCTAGISGGHINPAVTFGLFLARKVSLVRALLYIIAQCLGA 140
PvPIP2;1   GVGVLGIAWAFGGMIFILVYCTAGISGGHINPAVTFGLFLARKVSLVRALLYIVAQCLGA 140
SiPIP2;4   GVGILGIAWAFGGMIFILVYCTAGISGGHINPAVTFGLFLARKVSLVRAVLYIIAQCLGA 140
SbPIP2;5   GVGILGIAWSFGGMIFILVYCTAGISGGHINPAVTFGLFLARKVSLVRALLYIIAQCLGA 140
OsPIP2;2   GVGILGIAWAFGGMIFILVYCTAGISGGHINPAVTFGLFLARKVSLIRAVLYIIAQCLGA 140
ZmPIP2;5   GVG-LGIAWAFGGMIFILVYCTAGVSGGHINPAVTFGLFLARKVSLVRALLYIVAQCLGA 135
SbPIP2;6   GVGILGIAWAFGGMIFILVYCTAGVSGGHINPAVTFGLFLARKVSLVRALLYIVAQCLGA 143
SiPIP2;3   GVGILGIAWAFGGMIFILVYCTAGVSGGHINPAVTLGLFLARKVSLVRALLYIVAQCLGA 141
OsPIP2;3   GVGILGIAWAFGGMIFILVYCTAGVSGGHINPAVTFGLFLARKVSLVRAVLYIVAQSLGA 141
BdPIP2;2   GVGILGIAWAFGGMIFVLVYCTAGVSGGHINPAVTFGLFLARKVSLVRAVLYMVAQCLGA 137
BdPIP2;1   GVGILGIAWAFGGMIFVLVYCTAGVSGGHINPAVTFGLFLARKVSLVRAVLYIVAQCLGA 140
PvPIP2;2   GVGVLGIAWAFGGMIFILVYCTAGISGGHINPAVTFGLFLARKVSLVRALLYIVAQCLGA 140
PvPIP2;3   GVGVLGIAWAFGGMIFVLVYCTAGISGGHINPAVTFGLFLARKVSLVRALLYIVAQCLGA 141
PvPIP2;4   GVGVLGIAWAFGGMIFILVYCTAGVSGGHINPAVTLGLFLARKVSLVRALLYMVAQCLGA 139
PvPIP2;5   GVGILGIAWAFGGMIFILVYCTAGVSGGHINPAVTLGLFLARKVSLVRALLYIVAQCLGA 141
SbPIP2;1   GVGILGIAWAFGGMIFILVYCTAGISGGHINPAVTFGLFLARKVSLVRAILYMAAQSLGA 138
SbPIP2;2   GVGILGIAWAFGGMIFILVYCTAGISGGHINPAVTFGLFLARKVSLVRALLYMAAQSLGA 138
ZmPIP2;6   GVGILGIAWAFGGMIFILVYCTAGISGGHINPAVTFGLFLARKVSLVRALLYMAAQSLGA 140
SbPIP2;3   GVGILGIAWAFGGMIFILVYCTAGISGGHINPAVTFGLFLARKVSLVRALLYMAAQSLGA 138
SbPIP2;4   GVGILGIAWAFGGMIFILVYCTAGISGGHINPAVTFGLFLARKVSLVRALLYMAAQSLGA 138
PvPIP2;8   GVGILGIAWAFGGMIFILVYCTAGISGGHINPAVTFGLFLARKVSLVRAILYMAAQSLGA 138
PvPIP2;9   GVGILGIAWAFGGMIFILVYCTAGISGGHINPAVTFGLFLARKVSLVRAVLYMAAQCLGA 138
SiPIP2;1   GVGILGIAWAFGGMIFILVYCTAGISGGHINPAVTFGLFLARKVSLVRAILYMAAQCLGA 138
SiPIP2;2   GVGILGIAWAFGGMIFILVYCTAGISGGHINPAVTFGLFLARKVSLVRAVLYMAAQCLGA 138
OsPIP2;4   GVGVLGIAWAFGGMIFILVYCTAGVSGGHINPAVTLGLFLARKVSLVRALLYMAAQCLGA 138
BdPIP2;4   GVGILGIAWAFGGMIFVLVYCTAGVSGGHINPAVTFGLFLARKVSLVRALLYMVAQCLGA 141
OsPIP2;5   GVGVLGIAWAFGGMIFILVYCTAGISGGHINPAVTFGLFLARKVSLVRAILYIVAQCLGA 135
AtPIP2;2   GVGILGIAWAFGGMIFILVYCTAGISGGHINPAVTFGLFLARKVSLIRAVLYMVAQCLGA 134
AtPIP2;3   GVGILGIAWAFGGMIFILVYCTAGISGGHINPAVTFGLFLARKVSLIRAVLYMVAQCLGA 134
AtPIP2;1   GVGILGIAWAFGGMIFILVYCTAGISGGHINPAVTFGLFLARKVSLPRALLYIIAQCLGA 136
AtPIP2;4   GVGILGIAWAFGGMIFVLVYCTAGISGGHINPAVTVGLFLARKVSLVRTVLYIVAQCLGA 136
GmPIP2;7   GVGVLGIAWAFGGMIFVLVYCTAGISGGHINPAVTFGLFLARKVSLIRAVGYMVAQVLGA 135
GmPIP2;8   GVGVLGIAWAFGGMIFVLVYCTAGISGGHINPAVTFGLFLARKVSLTRAVGYMVAQVLGA 134
AtPIP2;5   GVGVLGIAWAFGGMIFILVYCTAGISGGHINPAVTFGLLLARKVTLVRAVMYMVAQCLGA 135
AtPIP2;6   SVGLLGISWAFGGMIFILVYCTAGISGGHINPAVTFGLFLASKVSLVRAVSYMVAQCLGA 135
PpPIP2;2   GVGLLGIAWAFGGMIFVLVYCTAGISGGHINPAVTFGLLLARKISLPRALAYMIAQCLGA 127
PpPIP2;3   GVGTLGIAWAFGGMIFVLVYCTAGISGGHINPAVTFGLLLARKISLPRALAYMIAQCLGA 128
PpPIP2;1   GVGLLGIAWAFGGMIFVLVYCTAGVSGGHINPAVTFGLLMARKISLPRALTYMIAQCLGA 127
PpPIP2;4   GVGILGIAWAFGGMIFVLVYCTAGISGGHINPAVTFGLLLARKISLTRSLAYMVAQCLGA 128
```

	TM2 ... TM3	
SmPIP2;1	GVGLLGIAWAFGGMIFVLVYCTAGISGGHINPAVTFGLFVARKVSLPRAIFYMIMQCLGA	130
SmPIP3;1	GIGLLGVAWVFGGMIFVLVYCTAGVSGGHLNPAVTFGMFLARKVSIPRALLYVASQVAGA	131
PpPIP3;1	SVGLIETAWAFGGMIFILVYCTAGISGGHINPAVTFGLFLAQQVTLPRASAYIVAQCLGA	120
PpPIP1;1	GVGIQGIAWAFGGMIFALVYCTAGISGGHINPAVTFGLFLARKVSLNRALFYMIMQCLGA	145
PpPIP1;2	GVGIQGIAWAFGGMIFALVYCTAGISGGHINPAVTFGLFLARKVSLNRALYYMIMQCLGA	145
PpPIP1;3	GVGIQGIAWAFGGMIFTLVYCTAGISGGHINPAVTFGLFLARKVTFPRTVLYIVCQCLGA	142
SmPIP1;1	GVGIQGIAWAFGGMIFALVYCTAGISGGHINPAVTFGLFLARKVSLPRTLLYMVAQCLGA	141
ZmPIP1;1	TVGIQGIAWSFGGMILALVYCTAGISG-HINPAVTFGLFLARKLSLTRAVFYIIMQCLGA	144
SbPIP1;1	TVGIQGIAWSFGGMIFALVYCTAGISGGHINPAVTFGLFLARKLSLTRAVFYIIMQCLGA	145
ZmPIP1;3	TVGIQGIAWSFGGMIFALVYCTAGISGGHINPAVTFGLFLARKLSLTRAIFYIIMQCLGA	149
ZmPIP1;4	TVGIQGIAWSFGGMIFALVYCTAGISGGHINPAVTFGLFLARKLSLTRAIFYIIMQCLGA	149
SbPIP1;2	TVGIQGIAWSFGGMIFALVYCTAGISGGHINPAVTFGLFLARKLSLTRAIFYIIMQCLGA	146
ZmPIP1;2	TVGIQGIAWSFGGMIFALVYCTAGISGGHINPAVTFGLFLARKLSLTRALFYIIMQCLGA	146
PvPIP1;1	TVGIQGIAWSFGGMIFALVYCTAGISGGHINPAVTFGLFLARKLSLTRAIFYMVMQCLGA	145
PvPIP1;2	TVGIQGIAWSFGGMIFALVYCTAGISGGHINPAVTFGLFLARKLSLTRAIFYIVMQCLGA	145
OsPIP1;1	TVGIQGIAWSFGGMIFALVYCTAGISGGHINPAVTFGLFLARKLSLTRAIFYIVMQCLGA	146
PvPIP1;4	TVGIQGIAWSFGGMIFALVYCTAGISGGHINPAVTFGLFLARKLSLTRAIFYIIMQCLGA	146
SiPIP1;1	TVGIQGIAWSFGGMIFALVYCTAGISGGHINPAVTFGLFLARKLSLTRAIFYIIMQCLGA	146
PvPIP1;5	TVGIQGIAWSFGGMIFALVYCTAGISGGHINPAVTFGLFLARKLSLTRAVFYIIMQCLGA	146
SiPIP1;4	TVGIQGIAWSFGGMIFALVYCTAGISGGHINPAVTFGLFLARKLSLTRAVFYMIMQCLGA	145
SiPIP1;2	TVGIQGIAWSFGGMIFALVYCTAGISGGHINPAVTFGLFLARKLSLTRAIFYIIMQCLGA	146
SiPIP1;3	TVGIQGIAWSFGGMIFALVYCTAGISGGHINPAVTFGLFLARKLSLTRAVFYMIMQCLGA	145
PvPIP1;3	TVGIQGIAWSFGGMIFALVYCTAGISGGHINPAVTFGLFLARKLSLTRAIFYMVMQCLGA	145
OsPIP1;4	TVGIQGIPWSFGSMIFALVYCTAGISGRHINPAVTFGLFLAKNVSLSRAIFYILMQCLGA	146
OsPIP1;2	TVGIQGIAWSFGGMIFALVYCTAGISGGHINPAVTFGLFLARKLSLTRALFYMVMQCLGA	145
OsPIP1;5	TVGIQGIAWSFGGMIFALVYCTAGISGGHINPAVTFGLFLARNLSLTRALFYMVMQCLGA	145
BdPIP1;1	TVGIQGIAWSFGGMIFALVYCTAGISGGHINPAVTFGLFLARKLSLTRAVFYIVMQCLGA	145
ZmPIP1;5	TVGIQGIAWSFGGMIFALVYCTAGISGGHINPAVTFGLFLARKLSLTRALFYMVMQCLGA	145
PvPIP1;7	TVGIQGIAWSFGGMIFALVYCTAGISGGHINPAVTFGLFLARKLSLTRALFYMVMQCLGA	145
PvPIP1;6	TVGIQGIAWSFGGMIFALVYCTAGISGGHINPAVTFGLFLARKLSLTRALFYMVMQCLGA	145
SiPIP1;5	TVGIQGIAWSFGGMIFALVYCTAGISGGHINPAVTFGLFLARKLSLTRALFYMVMQCLGA	145
SbPIP1;3	TVGIQGIAWSFGGMIFALVYCTAGISGGHINPAVTFGLLLARKLSLTRALLYMVMQCLGA	147
OsPIP1;3	TVGIQGIAWSFGGMIFALVYCTAGISGGHINPAVTFGLFLARKLSLTRAVFYMAMQCLGA	145
BdPIP1;2	TVGIQGIAWSFGGMIFVLVYCTAGISGGHINPAVTFGLFLARKLSLTRAVFYMVMQCLGA	146
GhPIP1;2	TVGIQGIAWAFGGMIFALVYCTAGISGGHINPAVTFGLFLARKLSLTRALFYMIMQCLGA	143
GhPIP1;5	TVGIQGIAWAFGGMIFALVYCTAGISGGHINPAVTFGLFLARKLSLTRALFYMIMQCLGA	143
PtPIP1;3	TVGIQGIAWAFGGMIFALVYCTAGISGGHINPAVTFGLFLARKLSLTRAVFYIIMQCLGA	144
AtPIP1;1	SVGIQGIAWAFGGMIFALVYCTAGISGGHINPAVTFGLFLARKLSLTRALYYIVMQCLGA	143
AtPIP1;2	SVGIQGIAWAFGGMIFALVYCTAGISGGHINPAVTFGLFLARKLSLTRAVYYIVMQCLGA	143
AtPIP1;3	SVGIQGIAWAFGGMIFALVYCTAGISGGHINPAVTFGLFLARKLSLTRAVFYIVMQCLGA	143
AtPIP1;4	SVGIQGIAWAFGGMIFALVYCTAGISGGHINPAVTFGLFLARKLSLTRAVFYMIMQCLGA	144
AtPIP1;5	SVGIQGIAWAFGGMIFALVYCTAGISGGHINPAVTFGLFLARKLSLTRALFYIVMQCLGA	144
PtPIP1;5	TVGIQGIAWAFGGMIFALVYCTAGISGGHINPAVTFGLLLARKLSLTRAVFYMLMQCLGA	145
GhPIP1;6	TVGIQGIAWAFGGMIFALVYCTAGISGGHINPAVTFGLFLARKLSLIRAVFYMIMQCLGA	145

```
GmPIP1;2      TVGVQGIAWSFGGMIFALVYCTAGISGGHINPAVTFGLFLARKLSLTRTVFYMIMQCLGA 162
PtPIP1;4      TVGIQGIAWAFGGMIFVLVYCTAGISGGHINPAVTFGLLLARKLTLTRAVFYMIMQCLGA 145
               :*    .* **.**: *******:** *:*****.*:::. :::: *:  *:  *  **

              TM3                                  TM4
PtPIP2;3      ICGVGLVKAFQK-SYYKKYGG-GANTLAD--GFSTGTGLGAEIIGTFVLVYTVFSATDPK 190
PtPIP2;4      ICGVGLVKAFQK-SYYTKYNG-GANVLAD--GYSTGTGLGAEIIGTFVLVYTVFSATDPK 190
GmPIP2;13     ICGVGLVKAFQK-SYFNKYGG-GANSLAA--GYSTGTGLGAEIIGTFVLVYTVFSATDPK 201
GmPIP2;14     ICGVGLVKAFQK-SYFNKYGG-GANSLAD--GYSTGTGLGAEIIGTFVLVYTVFSATDPK 189
GmPIP2;9      ICGVGLVKAFQK-AYYNRYGG-GANELSE--GYSTGVGLGAEIIGTFVLVYTVFSATDPK 190
GmPIP2;10     ICGVGLVKAFQK-AYYNRYGG-GANELSE--GYSTGVGLGAEIIGTFVLVYTVFSATDPK 190
GmPIP2;11     ICGVGLVKAFQK-AYYNRYGG-GANELSE--GYSTGVGLGAEIIGTFVLVYTVFSATDPK 190
GmPIP2;12     MCGVGLVKAFQK-AYYNRYGG-GANELSE--GYSTGVGLGAEIIGTFVLVYTVFSATDPK 190
PtPIP2;8      ICGCGLVKAFQK-SYYNHYGG-GANELQE--GYNKGTGLGAEIIGTFVLVYTVFSATDPK 191
PtPIP2;5      ICGCGLVKAFQK-SYYTKYGG-GVNELAT--GFSKGTGLGAEIIGTFVLVYTVFSATDPK 190
PtPIP2;6      ICGCGLVKAFQK-SYYTKYGG-GANELAT--GFSKGTGLGAEIIGTFVLVYTVFSATDPK 190
PtPIP2;7      ICGCGLVKAFQK-SYYTNYGG-GANGLAN--GYSKGTGLGAEIIGTFVLVYTVFSATDPK 190
GhPIP2;1      ICGCGLVKAFQK-SYYNKYGG-GANSLAD--GYSTGTGLAAEIIGTFVLVYTVFSATDPK 190
GhPIP2;2      ICGCGLVKAFQN-SYYNQYGG-GANSLAE--GYSTGTGLAAEIIGTFVLVYTVFSATDPK 190
ZmPIP2;7      ICGAGLAKGFQK-SFYNRYGG-GVNTVSD--GYNKGTALGAEIIGTFVLVYTVFSATDPK 194
GmPIP2;6      ICGAGLAKGFQK-SFYNRYGG-GVNTVSD--GYNKGTALGAEIIGTFVLVYTVFSATDPK 194
GmPIP2;5      ICGAGLAKGFQK-SYYNRYGG-GVNTVSD--GYNKGTALGAEIIGTFVLVYTVFSATDPK 194
GmPIP2;3      ICGTGLAKGFQK-SYYNRYGG-GANSVAD--GYNNGTALGAEIIGTFVLVYTVFSATDPK 193
GmPIP2;4      ICGTGLAKGFQK-AYYNRYGG-GANSVAD--GYNNGTALGAEIIGTFVLVYTVFSATDPK 193
GhPIP2;10     ICGCGLVKAFQK-TYYNSYGG-GANELQD--GFNKGTGLGAEIIGTFVLVYTVFSATDPK 198
ZmPIP2;1      ICGVGLVKAFQS-AYFDRYGG-GANSLAS--GYSRGTGLGAEIIGTFVLVYTVFSATDPK 197
SbPIP2;7      ICGVGLVKAFQS-AYFDRYGG-GANSLAS--GYSRGTGLGAEIIGTFVLVYTVFSATDPK 197
PvPIP2;6      ICGVGLVKAFQS-AYFDRYGG-GANSLAS--GYSRGTGLGAEIIGTFVLVYTVFSATDPK 197
PvPIP2;7      ICGVGLVKAFQS-AYFNRYGG-GANSLAS--GYSRGTGLGAEIIGTFVLVYTVFSATDPK 197
ZmPIP2;2      VCGVGLVKAFQS-AYFDRYGG-GANSLAS--GYSRGAGLGAEIVGTFVLVYTVFSATDPK 199
SiPIP2;5      ICGVGLVKAFQS-AYFDRYGG-GANSLAS--GYSRGTGLGAEIIGTFVLVYTVFSATDPK 196
OsPIP2;1      ICGVGLVKAFQS-AYFNRYGG-GANTLAA--GYSKGTGLAAEIIGTFVLVYTVFSATDPK 197
BdPIP2;3      MCGVGLVKAFQS-AYFVRYGG-GANTLAA--GYSKGTGLAAEIIGTFVLVYTVFSATDPK 197
ZmPIP2;3      ICGVGLVKGFQS-AYYVRYGG-GANELSD--GYSKGTGLAAEIIGTFVLVYTVFSATDPK 197
ZmPIP2;4      ICGVGLVKGFQS-AYYVRYGG-GANELSD--GYSKGTGLAAEIIGTFVLVYTVFSATDPK 196
PvPIP2;1      ICGVGLVKGFQS-AYFVRYGG-GANELSD--GYSKGTGLAAEIIGTFVLVYTVFSATDPK 196
SiPIP2;4      ICGVGLVKGFQS-AYFVRYGG-GANELSA--GYSKGTGLAAEIIGTFVLVYTVFSATDPK 196
SbPIP2;5      ICGVGLVKGFQS-AYYVRYGG-GANELSD--GYSKGTGLAAEIIGTFVLVYTVFSATDPK 196
OsPIP2;2      ICGVGLVKGFQS-SYYARYGG-GANELSD--GYSKGTGLGAEIIGTFVLVYTVFSATDPK 196
ZmPIP2;5      ICGVGLVKGFQS-AFYVRYGG-GANELSA--GYSKGTGLAAEIIGTFVLVYTVFSATDPK 191
SbPIP2;6      ICGVGLVKGFQS-AFYVRYGG-GANGLSD--GYSKGTGLAAEIIGTFVLVYTVFSATDPK 199
```

```
                    TM3                                    TM4
SiPIP2;3   ICGVGLVKGFQS-AYYVRYGG-GANELSD--GYSKGTGLAAEIIGTFVLVYTVFSATDPK 197
OsPIP2;3   ICGVGLVKGFQS-AFYVRYGG-GANELSD--GYSKGTGLAAEIIGTFVLVYTVFSATDPK 197
BdPIP2;2   ICGVGLVKGFQS-AYFVRYGG-GANGLSA--GYSKGTGLAAEIIGTFVLVYTVFSATDPK 193
BdPIP2;1   ICGVGLVKGFQS-AYYVRYGG-GANELSA--GYSKGTGLAAEIIGTFVLVYTVFSATDPK 196
PvPIP2;2   ICGVGLVKGFQS-AYFVRYGG-GANELSD--GYSKGTGLAAEIIGTFVLVYTVFSATDPK 196
PvPIP2;3   ICGVGLVKAFQS-AYFNRYGG-GANSLAS--GYSRGTGLGAEIIGTFVLVYTVFSATDPK 197
PvPIP2;4   ICGVGLVKGFQS-AYFVRYGG-GANELSD--GYSKGTGLAAEIIGTFVLVYTVFSATDPK 195
PvPIP2;5   ICGVGLVKGFQS-AYFVRYGG-GANELSD--GYSKGTGLAAEIIGTFVLVYTVFSATDPK 197
SbPIP2;1   ICGVALVKGFQS-GFYARYGG-GANEVSP--GYSTGTGLAAEIIGTFVLVYTVFSATDPK 194
SbPIP2;2   ICGVALVKGFQS-GFYARYGG-GANEVSP--GYSTGTGLAAEIIGTFVLVYTVFSATDPK 194
ZmPIP2;6   ICGVALVKGFQS-GFYARYGG-GANEVSA--GYSTGTGLAAEIIGTFVLVYTVFSATDPK 196
SbPIP2;3   ICGVALVKGFQS-GFYARYGG-GANEVSP--GYSTGTGLAAEIIGTFVLVYTVFSATDPK 194
SbPIP2;4   ICGVALVKGFQS-GFYTRYGG-GANEVSP--GYSTGTGLAAEIIGTFVLVYTVFSATDPK 194
PvPIP2;8   ICGVALVKGFQS-GFYKRYGG-GANEVGA--GYSTGTGLAAEIIGTFVLVYTVFSATDPK 194
PvPIP2;9   ICGVALVKGFQS-GFYARYGG-GANEVSA--GYSTGTGLAAEIVGTFVLVYTVFSATDPK 194
SiPIP2;1   ICGVALVKGFQS-GFYVRYGG-GANEVST--GYSTGTGLAAEIVGTFVLVYTVFSATDPK 194
SiPIP2;2   ICGVALVKGFQS-GFYARYGG-GANEVSA--GYSTGTGLAAEIIGTFVLVYTVFSATDPK 194
OsPIP2;4   ICGVALVKGFQS-SLYDRYGG-GANELAA--GYSTGTGLAAEIIGTFVLVYTVFSATDPK 194
BdPIP2;4   ICGVGLVKGFQR-DFYARYGG-GANGVSA--GYSMGTGLAAEIIGTFVLVYTVFSATDSK 197
OsPIP2;5   VCGVALVKGFQS-SFYDRYGG-GANELAA--GYSKGTGLAAEIIGTFVLVYTVFSATDPK 191
AtPIP2;2   ICGVGFVKAFQS-SYYDRYGG-GANSLAD--GYNTGTGLAAEIIGTFVLVYTVFSATDPK 190
AtPIP2;3   ICGVGFVKAFQS-SHYVNYGG-GANFLAD--GYNTGTGLAAEIIGTFVLVYTVFSATDPK 190
AtPIP2;1   ICGVGFVKAFQS-SYYTRYGG-GANSLAD--GYSTGTGLAAEIIGTFVLVYTVFSATDPK 192
AtPIP2;4   ICGCGFVKAFQS-SYYTRYGG-GANELAD--GYNKGTGLGAEIIGTFVLVYTVFSATDPK 192
GmPIP2;7   ISGVGLVKALQK-SYYNRYNG-GVNMLAD--GYSKGTGLGAEIIGTFILVYTVFSATDPK 191
GmPIP2;8   ISGVGLVKALQK-SYYNRYKG-GVNMLAD--GYSKGTGLGAEIIGTFILVYTVFSATDPK 190
AtPIP2;5   ICGVALVKAFQS-AYFTRYGG-GANGLSD--GYSIGTGVAAEIIGTFVLVYTVFSATDPK 191
AtPIP2;6   TCGVGLVKVFQS-TYYNRYGG-GANMLSD--GYNVGVGVGAEIIGTFVLVYTVFSATDPK 191
PpPIP2;2   ICGAGLVKGFQT-AFYMRYGG-GANSVAA--GYSIGTGLAAEIIGTFVLVYTVFSATDPK 183
PpPIP2;3   ICGAGLVKGFQQ-SFYMTYGG-GANAVNA--GYGIGTGLAAEIIGTFVLVYTVFSATDPK 184
PpPIP2;1   ICGAGLAKGFQT-AFYMRYGG-GANSVAL--GYSTGTGLAAEIIGTFVLVYTVFSATDPK 183
PpPIP2;4   ICGAGLVKEFQH-SFYMDHGG-GANAVAP--GYSTGTGLAAEIIGTFVLMFTVFSATDPK 184
SmPIP2;1   IVGCGLAKGFQK-SFYVQQGG-GANSVARARGYSTGTGLGAEIIGTFVLVYTVFSATDPK 188
SmPIP3;1   IFGAGLAKGFQA-SFYNGNMG-GATFIQN--GYTKAEGLGAEIIGTFVLAYTVFSATDPK 187
PpPIP3;1   IVGAAIARGVQEGGEYRSFASNAVNGVQP--GYNIGQALAAEIMGTFVLLYTVLSATDPT 178
PpPIP1;1   MCGAEIVKGFQPN-FYQEQGG-GSNSVAH--GYTKGDGLGAEIVGTFVLVYTVFSATDAK 201
PpPIP1;2   MAGAGIVKGFQPD-FYQAQGG-GANAVNH--GYTKGDGLGAEIVGTFVLVYTVFSATDAK 201
PpPIP1;3   ICGAGAVKGFQPD-FYQSVGG-GANTVAH--GYTKGDGLGAEIVGTFVLVYTVFSATDAK 198
SmPIP1;1   ICGAGVVKGFQKA-KFNAAGG-GANYVHH--GYTIGDGLGAEIVGTFVLVYTVFSATDAK 197
ZmPIP1;1   ICGRGVVKGFQQG-LYMGNGG-RRNVVAP--GYTKGDGLGAEIVGTFILVYTVFSATDAK 200
SbPIP1;1   ICGAGVVKGFQQG-LYMGNGG-GANVVAP--GYTKGDGLGAEIVGTFVLVYTVFSATDAK 201
ZmPIP1;3   ICGAGVVKGFQQG-LYMGNGG-GANVVAP--GYTKGDGLGAEIVGTFILVYTVFSATDAK 205
ZmPIP1;4   ICGAGVVKGFQQG-LYMGNGG-GANVVAP--GYTKGDGLGAEIVGTFILVYTVFSATDAK 205
SbPIP1;2   ICGAGVVKGFQQG-LYMGNGG-GANVVAP--GYTKGDGLGAEIVGTFILVYTVFSATDAK 202
```

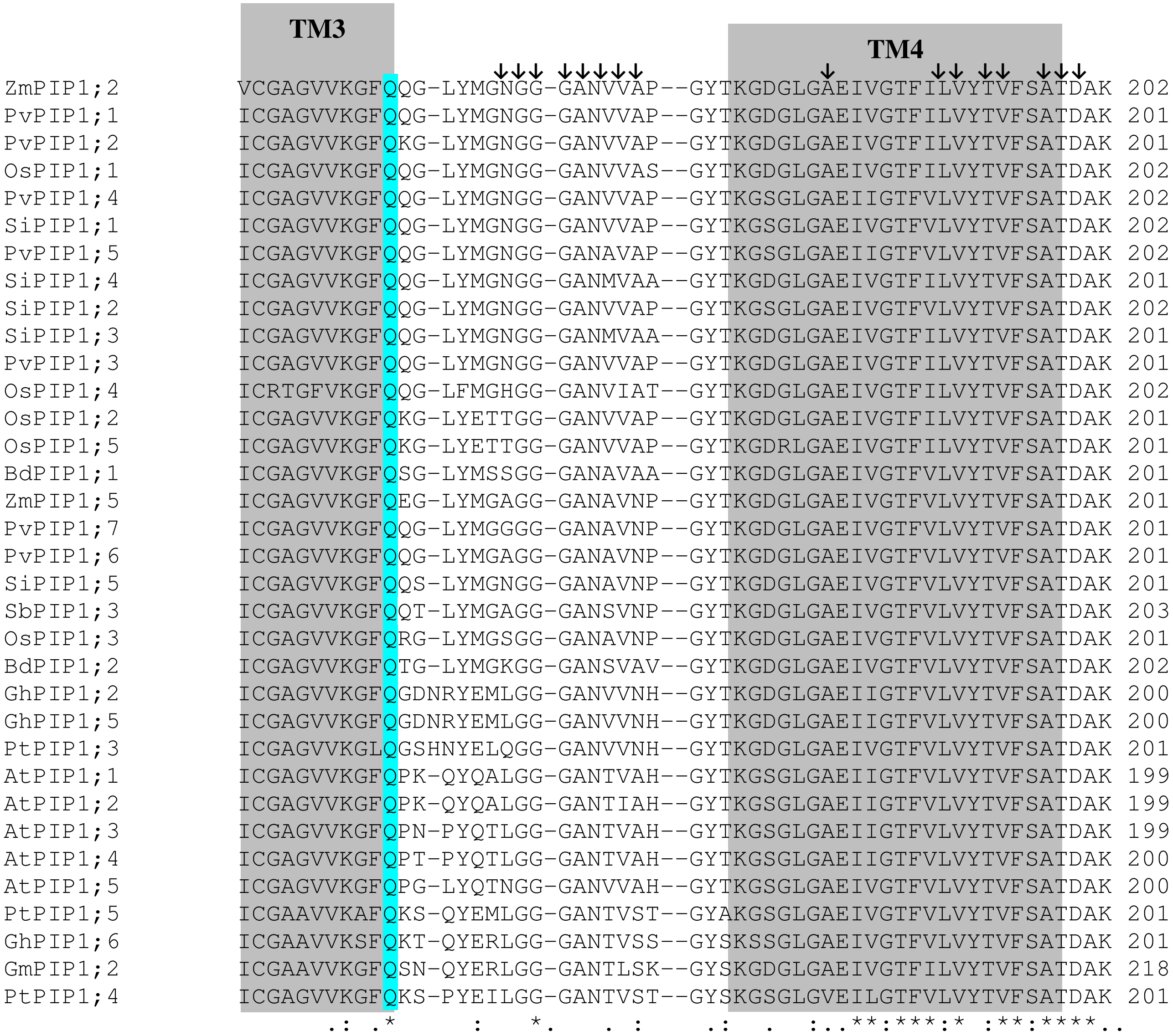
TM3
TM4
ZmPIP1;2 VCGAGVVKGFQQG-LYMGNGG-GANVVAP--GYTKGDGLGAEIVGTFILVYTVFSATDAK 202
PvPIP1;1 ICGAGVVKGFQQG-LYMGNGG-GANVVAP--GYTKGDGLGAEIVGTFILVYTVFSATDAK 201
PvPIP1;2 ICGAGVVKGFQKG-LYMGNGG-GANVVAP--GYTKGDGLGAEIVGTFILVYTVFSATDAK 201
OsPIP1;1 ICGAGVVKGFQQG-LYMGNGG-GANVVAS--GYTKGDGLGAEIVGTFILVYTVFSATDAK 202
PvPIP1;4 ICGAGVVKGFQQG-LYMGNGG-GANVVAP--GYTKGSGLGAEIIGTFVLVYTVFSATDAK 202
SiPIP1;1 ICGAGVVKGFQQG-LYMGNGG-GANVVAP--GYTKGSGLGAEIVGTFVLVYTVFSATDAK 202
PvPIP1;5 ICGAGVVKGFQQG-LYMGNGG-GANAVAP--GYTKGSGLGAEIIGTFVLVYTVFSATDAK 202
SiPIP1;4 ICGAGVVKGFQQG-LYMGNGG-GANMVAA--GYTKGDGLGAEIVGTFILVYTVFSATDAK 201
SiPIP1;2 ICGAGVVKGFQQG-LYMGNGG-GANVVAP--GYTKGSGLGAEIVGTFVLVYTVFSATDAK 202
SiPIP1;3 ICGAGVVKGFQQG-LYMGNGG-GANMVAA--GYTKGDGLGAEIVGTFILVYTVFSATDAK 201
PvPIP1;3 ICGAGVVKGFQQG-LYMGNGG-GANVVAP--GYTKGDGLGAEIVGTFILVYTVFSATDAK 201
OsPIP1;4 ICRTGFVKGFQQG-LFMGHGG-GANVIAT--GYTKGDGLGAEIVGTFILVYTVFSATDAK 202
OsPIP1;2 ICGAGVVKGFQKG-LYETTGG-GANVVAP--GYTKGDGLGAEIVGTFILVYTVFSATDAK 201
OsPIP1;5 ICGAGVVKGFQKG-LYETTGG-GANVVAP--GYTKGDRLGAEIVGTFILVYTVFSATDAK 201
BdPIP1;1 ICGAGVVKGFQSG-LYMSSGG-GANAVAA--GYTKGDGLGAEIVGTFVLVYTVFSATDAK 201
ZmPIP1;5 ICGAGVVKGFQEG-LYMGAGG-GANAVNP--GYTKGDGLGAEIVGTFVLVYTVFSATDAK 201
PvPIP1;7 ICGAGVVKGFQQG-LYMGGGG-GANAVNP--GYTKGDGLGAEIVGTFVLVYTVFSATDAK 201
PvPIP1;6 ICGAGVVKGFQQG-LYMGAGG-GANAVNP--GYTKGDGLGAEIVGTFVLVYTVFSATDAK 201
SiPIP1;5 ICGAGVVKGFQQS-LYMGNGG-GANAVNP--GYTKGDGLGAEIVGTFVLVYTVFSATDAK 201
SbPIP1;3 ICGAGVVKGFQQT-LYMGAGG-GANSVNP--GYTKGDGLGAEIVGTFVLVYTVFSATDAK 203
OsPIP1;3 ICGAGVVKGFQRG-LYMGSGG-GANAVNP--GYTKGDGLGAEIVGTFVLVYTVFSATDAK 201
BdPIP1;2 ICGAGVVKGFQTG-LYMGKGG-GANSVAV--GYTKGDGLGAEIVGTFVLVYTVFSATDAK 202
GhPIP1;2 ICGAGVVKGFQGDNRYEMLGG-GANVVNH--GYTKGDGLGAEIIGTFVLVYTVFSATDAK 200
GhPIP1;5 ICGAGVVKGFQGDNRYEMLGG-GANVVNH--GYTKGDGLGAEIIGTFVLVYTVFSATDAK 200
PtPIP1;3 ICGAGVVKGLQGSHNYELQGG-GANVVNH--GYTKGDGLGAEIVGTFVLVYTVFSATDAK 201
AtPIP1;1 ICGAGVVKGFQPK-QYQALGG-GANTVAH--GYTKGSGLGAEIIGTFVLVYTVFSATDAK 199
AtPIP1;2 ICGAGVVKGFQPK-QYQALGG-GANTIAH--GYTKGSGLGAEIIGTFVLVYTVFSATDAK 199
AtPIP1;3 ICGAGVVKGFQPN-PYQTLGG-GANTVAH--GYTKGSGLGAEIIGTFVLVYTVFSATDAK 199
AtPIP1;4 ICGAGVVKGFQPT-PYQTLGG-GANTVAH--GYTKGSGLGAEIIGTFVLVYTVFSATDAK 200
AtPIP1;5 ICGAGVVKGFQPG-LYQTNGG-GANVVAH--GYTKGSGLGAEIVGTFVLVYTVFSATDAK 200
PtPIP1;5 ICGAAVVKAFQKS-QYEMLGG-GANTVST--GYAKGSGLGAEIVGTFVLVYTVFSATDAK 201
GhPIP1;6 ICGAAVVKSFQKT-QYERLGG-GANTVSS--GYSKSSGLGAEIVGTFVLVYTVFSATDAK 201
GmPIP1;2 ICGAAVVKGFQSN-QYERLGG-GANTLSK--GYSKGDGLGAEIVGTFILVYTVFSATDAK 218
PtPIP1;4 ICGAGVVKGFQKS-PYEILGG-GANTVST--GYSKGSGLGVEILGTFVLVYTVFSATDAK 201
.: .* : *. . : .: . :..**:***:* :**:****..

```
                      TM5
PtPIP2;3    RSARDSHVP--------VLAPLPIGFAVFMVHLATIPITGTGINPARSLGAAVIYN---Q 239
PtPIP2;4    RSARDSHVP--------VLAPLPIGFAVFMVHLATIPITGTGINPARSFGAAVIYN---N 239
GmPIP2;13   RNARDSHVP--------VLAPLPIGFAVFMVHLATIPVTGTGINPARSLGAAVIYN---Q 250
GmPIP2;14   RNARDSHVP--------VLAPLPIGFAVFMVHLATIPVTGTGINPARSLGAAVIYN---Q 238
GmPIP2;9    RNARDSHVP--------VLAPLPIGFAVFMVHLATIPVTGTGINPARSLGAAVMYN---Q 239
GmPIP2;10   RNARDSHVP--------VLAPLPIGFAVFMVHLATIPVTGTGINPARSLGAAVMYN---Q 239
GmPIP2;11   RNARDSHVP--------VLAPLPIGFAVFMVHLATIPVTGTGINPARSLGAAVMYN---Q 239
GmPIP2;12   RNARDSHVP--------VLAPLPIGFAVFMVHLATIPVTGTGINPARSFGAAVMYN---Q 239
PtPIP2;8    RNARDSHVP--------VLAPLPIGFAVFMVHLATIPITGTGINPARSFGAAVIFN---Q 240
PtPIP2;5    RNARDSHVP--------VLAPLPIGFAVFMVHLATIPITGTGINPARSFGAAVIYN---E 239
PtPIP2;6    RNARDSHVP--------VLAPLPIGFAVFMVHLATIPITGTGINPARSFGAAVIYN---K 239
PtPIP2;7    RNARDSHVP--------VLAPLPIGFAVFMVHLATIPITGTGINPARSFGAAVIFN---K 239
GhPIP2;1    RNARDSHVP--------VLAPLPIGFAVFMVHLATIPITGTGINPARSFGAAVMFN---Q 239
GhPIP2;2    RNARDSHIPV-------VLAPLPIGFAVFMVHLATIPITGTGINPARSLGAAVIFN---Q 240
ZmPIP2;7    RNARDSHVP--------VLAPLPIGFAVFMVHLATIPVTGTGINPARSFGPAVIFN---N 243
GmPIP2;6    RNARDSHVP--------VLAPLPIGFAVFMVHLATIPVTGTGINPARSFGPAVIFN---N 243
GmPIP2;5    RSARDSHVP--------VLAPLPIGFAVFMVHLATIPVTGTGINPARSFGPAVIFN---N 243
GmPIP2;3    RNARDSHVP--------VLAPLPIGFAVFMVHLATIPITGTGINPARSFGAAVIYN---K 242
GmPIP2;4    RNARDSHVP--------VLAPLPIGFAVFMVHLATIPITGTGINPARSFGAAVIYN---E 242
GhPIP2;10   RSARDSHVP--------VLAPLPIGFAVFMVHLATIPITGTGINPARSFGAAVIYN---Q 247
ZmPIP2;1    RNARDSHVP--------VLAPLPIGFAVFMVHLATIPVTGTGINPARSLGAAVIYN---K 246
SbPIP2;7    RNARDSHVP--------VLAPLPIGFAVFMVHLATIPVTGTGINPARSLGAAVIYN---K 246
PvPIP2;6    RNARDSHVP--------VLAPLPIGFAVFMVHLATIPVTGTGINPARSLGAAVIYN---K 246
PvPIP2;7    RNARDSHVP--------VLAPLPIGFAVFMVHLATIPVTGTGINPARSLGAAVIYN---K 246
ZmPIP2;2    RNARDSHVP--------VLAPLPIGFAVFMVHLATIPVTGTGINPARSLGAAVVYN---K 248
SiPIP2;5    RNARDSHVP--------VLAPLPIGFAVFMVHLATIPVTGTGINPARSLGAAVIYN---K 245
OsPIP2-1    RNARDSHVP--------VLAPLPIGFAVFMVHLATIPITGTGINPARSIGAAVIFN---N 246
BdPIP2;3    RSARDSHVP--------VLAPLPIGFAVFMVHLATIPITGTGINPARSLGAAVIFN---K 246
ZmPIP2;3    RSARDSHVP--------VLAPLPIGFAVFMVHLATIPITGTGINPARSLGAAVIYN---K 246
ZmPIP2;4    RSARDSHVP--------VLAPLPIGFAVFMVHLATIPITGTGINPARSLGAAVIYN---K 245
PvPIP2;1    RNARDSHVP--------VLAPLPIGFAVFMVHLATIPITGTGINPARSLGAAVIYN---K 245
SiPIP2;4    RSARDSHVP--------VLAPLPIGFAVFMVHLATIPITGTGINPARSLGAAVIYN---N 245
SbPIP2;5    RNARDSHVP--------VLAPLPIGFAVFMVHLATIPITGTGINPARSLGAAVIYN---N 245
OsPIP2;2    RNARDSHIP--------VLAPLPIGFAVFMVHLATIPITGTGINPARSLGTAVIYN---K 245
ZmPIP2;5    RNARDSHVP--------VLAPLPIGFAVFMVHLATIPITGTGINPARSLGAAVIYN---N 240
SbPIP2;6    RNARDSHVP--------VLAPLPIGFAVFMVHLATIPITGTGINPARSLGAAVIYN---N 248
SiPIP2;3    RNARDSHVP--------VLAPLPIGFAVFMVHLATIPITGTGINPARSLGAAVIYN---N 246
OsPIP2;3    RNARDSHVP--------VLAPLPIGFAVFMVHLATIPITGTGINPARSLGAAVIYN---Q 246
BdPIP2;2    RSARDSHVP--------VLAPLPIGFAVFMVHLATIPITGTGINPARSLGAAVIYN---N 242
```

TM5

```
BdPIP2;1    RNARDSHIP--------VLAPLPIGFAVFMVHLATIPITGTGINPARSLGAAVIYN---T 245
PvPIP2;2    RNARDSHVP--------VLAPLPIGFAVFMVHLATIPITGTGINPARSLGAAVIYN---K 245
PvPIP2;3    RNARDSHVP--------VLAPLPIGFAVFMVHLATIPVTGTGINPARSLGAAVIYN---K 246
PvPIP2;4    RSARDSHVP--------VLAPLPIGFAVFMVHLATIPITGTGINPARSFGAAVIYN---E 244
PvPIP2;5    RNARDSHVP--------VLAPLPIGFAVFMVHLATIPITGTGINPARSFGAAVIYN---N 246
SbPIP2;1    RNARDSHVP--------VLAPLPIGFAVFMVHLATIPITGTGINPARSLGAAVVYN---N 243
SbPIP2;2    RNARDSHVP--------VLAPLPIGFAVFMVHLATIPITGTGINPARSLGAAVVYN---N 243
ZmPIP2;6    RNARDSHVP--------VLAPLPIGFAVFMVHLATIPITGTGINPARSLGAAVVYN---N 245
SbPIP2;3    RNARDSHVP--------VLAPLPIGFAVFMVHLATIPITGTGINPARSLGAAVVYN---N 243
SbPIP2;4    RNARDSHVP--------VLAPLPIGFAVFMVHLATIPITGTGINPARSLGAAVVYN---N 243
PvPIP2;8    RNARDSHVP--------VLAPLPIGFAVFMVHLATIPITGTGINPARSLGAAVVYN---N 243
PvPIP2;9    RNARDSHVP--------VLAPLPIGFAVFMVHLATIPVTGTGINPARSLGAAVVYN---N 243
SiPIP2;1    RNARDSHIP--------VLAPLPIGFAVFMVHLATIPITGTGINPARSLGAAVVYN---N 243
SiPIP2;2    RNARDSHVP--------VLAPLPIGFAVFMVHLATIPITGTGINPARSLGAAVVYN---N 243
OsPIP2;4    RNARDSHVP--------VLAPLPIGFAVFMVHLATIPITGTGINPARSLGVAVVYN---N 243
BdPIP2;4    RNARDSHVP--------VLAPLPIGFAVFMVHLATIPITGTGINPARSLGAAVVYN---N 246
OsPIP2;5    RNARDSHVP--------VLAPLPIGFAVFMVHLATIPVTGTGINPARSLGAAVVYN---N 240
AtPIP2;2    RNARDSHVP--------VLAPLPIGFAVFMVHLATIPITGTGINPARSFGAAVIYN---K 239
AtPIP2;3    RNARDSHVP--------VLAPLPIGFAVFMVHLATIPITGTGINPARSFGAAVIFN---K 239
AtPIP2;1    RSARDSHVP--------VLAPLPIGFAVFMVHLATIPITGTGINPARSFGAAVIYN---K 241
AtPIP2;4    RNARDSHVP--------VLAPLPIGFAVFMVHLATIPITGTGINPARSFGAAVIYN---N 241
GmPIP2;7    RVARDSHVP--------VLAPLPIGFAVFIVHLATIPITGTGINPARSLGPAVIFN---N 240
GmPIP2;8    RVARDSHVP--------VLAPLPIGFAVFMVHLATIPITGTGINPARSLGPAVIFN---N 239
AtPIP2;5    RSARDSHVP--------VLAPLPIGFAVFIVHLATIPITGTGINPARSLGAAIIYN---K 240
AtPIP2;6    RNARDSHIP--------VLAPLPIGFSVFMVHLATIPITGTGINPARSFGAAVIYN---N 240
PpPIP2;2    RNARDSHVP--------VLAPLPIGFAVFMVHLATIPITGTGINPARSFGAAVIYN---R 232
PpPIP2;3    RNARDSHVP--------VLAPLPIGFAVFMVHLATIPITGTGINPARSFGAAVIYN---R 233
PpPIP2;1    RNARDSHVP--------VLAPLPIGFAVFMVHLATIPITGTGINPARSFGAAVIYN---R 232
PpPIP2;4    RKARDSHVP--------VLAPLPIGFAVFVVHCATIPITGTGINPARSFGAAVIFN---R 233
SmPIP2;1    RVARDSHVP--------VLAPLPIGFAVFMVHLATIPITGTGINPARSFGAAVIFN---K 237
SmPIP3;1    RVARDSHVP--------VLAPLPIGFAVFMVHLALLPVTGSSVNPARSFATAVIYN---N 236
PpPIP3;1    RKARDSHVP--------VLAPLPIGFAIFVVHLATIPITGTGINPARSLGAAV------- 223
PpPIP1;1    RNARDSHVP--------VLAPLPIGFAVFLVHLATIPITGTGINPARSLGAAVVFNK--Q 251
PpPIP1;2    RSARDSHVP--------VLAPLPIGFAVFLVHLATIPITGTGINPARSLGAATIYNT--Q 251
PpPIP1;3    RNARDSHVP--------LLAPLPIGFAVFLVHLATIPITGTSINPARSLGAAVIWN---R 247
SmPIP1;1    RSARDSHVP--------LLAPLPIGFAVFLVHLATIPITGTGINPARSLGSAVIYN---G 246
ZmPIP1;1    RRARDSHVP--------ILAPLPIGFAVFLVHLATMGITGTGINPARSLGAAVIYN---Q 249
SbPIP1;1    RNARDSHVP--------ILAPLPIGFAVFLVHLATIPITGTGINPARSLGAAVVYN---Q 250
ZmPIP1;3    RNARDSHVP--------ILAPLPIGFAVFLVHLATIPITGTGINPARSLGAAIIYN---R 254
ZmPIP1;4    RNARDSHVP--------ILAPLPIGFAVFLVHLATIPITGTGINPARSLGAAIIYN---R 254
```

TM5

```
SbPIP1;2     RNARDSHVP--------ILAPLPIGFAVFLVHLATIPITGTGINPARSLGAAIIYN---R 251
ZmPIP1;2     RNARDSHVP--------ILAPLPIGFAVFLVHLATIPITGTGINPARSLGAAIIYN---R 251
PvPIP1;1     RNARDSHVP--------ILAPLPIGFAVFLVHLATIPITGTGINPARSLGAAIIYN---R 250
PvPIP1;2     RNARDSHVP--------ILAPLPIGFAVFLVHLATIPITGTGINPARSLGAAIIYN---R 250
OsPIP1;1     RNARDSHVP--------ILAPLPIGFAVFLVHLATIPITGTGINPARSLGAAIIYN---K 251
PvPIP1;4     RNARDSHVP--------ILAPLPIGFAVFLVHLATIPITGTGINPARSLGAAIIYN---R 251
SiPIP1;1     RNARDSHVP--------ILAPLPIGFAVFLVHLATIPITGTGINPARSLGAAIIYN---R 251
PvPIP1;5     RNARDSHVP--------ILAPLPIGFAVFLVHLATIPITGTGINPARSLGAAIIYN---R 251
SiPIP1;4     RNARDSHVP--------ILAPLPIGFAVFLVHLATIPITGTGINPARSLGAAVIYN---R 250
SiPIP1;2     RNARDSHVP--------ILAPLPIGFAVFLVHLATIPITGTGINPARSLGAAIIYN---R 251
SiPIP1;3     RNARDSHVP--------ILAPLPIGFAVFLVHLATIPITGTGINPARSLGAAVIYN---R 250
PvPIP1;3     RNARDSHVP--------ILAPLPIGFAVFLVHLATIPITGTGINPARSLGAAIIYN---R 250
OsPIP1;4     RNARDSHVP--------ILAPLPIGFAVFLVHLATIPITGTGINPARSLGAAIIYN---K 251
OsPIP1;2     RNARDSHVP--------ILAPLPIGFAVFLVHLATIPITGTGINPARSLGAAIIYN---R 250
OsPIP1;5     KNARDSHVP--------ILAPLPIGFAVFLVHLATIPITGTGINPRRTWGCHHLQQGHAW 253
BdPIP1;1     RNARDSHVP--------ILAPLPIGFAVFLVHLATIPITGTGINPARSLGAAIIYN---K 250
ZmPIP1;5     RSARDSHVP--------ILAPLPIGFAVFLVHLATIPITGTGINPARSLGAAIVYN---R 250
PvPIP1;7     RSARDSHVP--------ILAPLPIGFAVFLVHLATIPITGTGINPARSLGAAIIYN---R 250
PvPIP1;6     RSARDSHVP--------ILAPLPIGFAVFLVHLATIPITGTGINPARSLGAAIIYN---R 250
SiPIP1;5     RSARDSHVP--------ILAPLPIGFAVFLVHLATIPITGTGINPARSLGAAIVYN---R 250
SbPIP1;3     RSARDSHVP--------ILAPLPIGFAVFLVHLATIPITGTGINPARSLGAAIVYN---R 252
OsPIP1;3     RNARDSHVP--------ILAPLPIGFAVFLVHLATIPITGTGINPARSLGAAIVYN---R 250
BdPIP1;2     RSARDSHVP--------ILAPLPIGFAVFLVHLATIPITGTGINPARSLGAAIIYN---R 251
GhPIP1;2     RNARDSHVP--------ILAPLPIGFAVFLVHLATIPITGTGINPARSLGAAIIYN---K 249
GhPIP1;5     RNARDSHVP--------ILAPLPIGFAVFLVHLATIPITGTGINPARSLGAAIIYN---K 249
PtPIP1;3     RNARDSHVP--------ILAPLPIGFAVFLVHLATIPITGTGINPARSLGAAIIFN---K 250
AtPIP1;1     RNARDSHVP--------ILAPLPIGFAVFLVHLATIPITGTGINPARSLGAAIIYN---K 248
AtPIP1;2     RNARDSHVP--------ILAPLPIGFAVFLVHLATIPITGTGINPARSLGAAIIFN---K 248
AtPIP1;3     RSARDSHVP--------ILAPLPIGFAVFLVHLATIPITGTGINPARSLGAAIIYN---K 248
AtPIP1;4     RSARDSHVPVWTPLLVPILAPLPIGFAVFLVHLATIPITGTGINPARSLGAAIIYN---K 257
AtPIP1;5     RSARDSHVP--------ILAPLPIGFAVFLVHLATIPITGTGINPARSLGAAIIYN---K 249
PtPIP1;5     RNARDSHVP--------ILAPLPIGFAVFLVHLATIPITGTGINPARSLGAALIYN---K 250
GhPIP1;6     RNARDSHVP--------ILAPLPIGFAVFLVHLATIPVTGTGINPARSLGAALIYN---K 250
GmPIP1;2     RNARDSHVP--------ILAPLPIGFAVFLVHLATIPITGTGINPARSLGAALVYN---K 267
PtPIP1;4     RSARDSHVP--------VLAPLPIGFAVFLVHLATIPITGTGINPARSLGAALIYN---K 250
             : *****:*        :********::.:** * : :**:.:** *: .
```

```
                  TM6
PtPIP2;3    DKAWDGHWIFW-VGP----FAGAAIAAFYHQFILRA-GAVKALGSFRSAQRF-------- 285
PtPIP2;4    KKAWHDQWIFW-AGP----FIGAAIAAFYHQFILRA-GAIKALGSFRSNPNV-------- 285
GmPIP2;13   DKPWDDHWIFW-VGP----FIGAAIAAFYHQFILRA-GAAKALGSFRSNPHN-------- 296
GmPIP2;14   DKPWDDHWIFW-VGP----FIGAAIAAFYHQFILRA-GAAKALGSFRSNPHN-------- 284
GmPIP2;9    QKAWDDHWIFW-VGP----FIGAAIAAFYHQFILRA-GAAKALGSFRSNPTI-------- 285
GmPIP2;10   QKAWDDHWIFW-VGP----FIGAAIAAFYHQFILRA-GAAKALGSFRSNPAI-------- 285
GmPIP2;11   QKAWDDHWIFW-VGP----FIGAAIAAFYHQFILRA-SAAKALGSFRSNPTI-------- 285
GmPIP2;12   KKAWDDQWIFW-VGP----FIGAAIAAFYHQFILRA-SAAKAVGSFRSNPTI-------- 285
PtPIP2;8    SKAWDDHWLFW-VGP----FIGAAIAAFYHQFILRA-AAIKALGSFRSNA---------- 284
PtPIP2;5    DKAWDDHWIFW-VGP----FIGAAIAALYHQYVLRA-AAVKALGSFRSSSNI-------- 285
PtPIP2;6    DKAWDDHWIFW-VGP----FIGAAIAALYHQYVLRA-AAVKALGSFRSSSNI-------- 285
PtPIP2;7    EKAWDDHWIFW-VGP----FIGAAIAALYHQFILRA-AAVKSLGSFRSSPNI-------- 285
GhPIP2;1    DKPWDDHWIFW-VGP----FIGAAIAAIYHQYILRA-GAAKALGSFRSSSAM-------- 285
GhPIP2;2    DKIWDDHWIFW-VGP----FIGAAIAAIYHQFILRA-SGAKALGSFRSSFAM-------- 286
ZmPIP2;7    DKAWDDQWIYW-VGP----FVGAAVAAIYHQYILRG-SAIKALGSFRSNA---------- 287
GmPIP2;6    DKAWDDQWIYW-VGP----FVGAAVAAIYHQYILRG-SAIKALGSFRSNA---------- 287
GmPIP2;5    DKAWDDQWIYW-VGP----FVGAAVAAFYHQYILRA-AAIKALGSFRSNT---------- 287
GmPIP2;3    DKIWDDQWIFW-VGP----IVGAAVAAFYHQYILRA-AAIKALGSFRSNA---------- 286
GmPIP2;4    DKIWDDQWIFW-VGP----IVGAAVAAFYHQYILRA-AAIKALGSFRSNA---------- 286
GhPIP2;10   EKSLAMINGYSGLDP----SLGAFVAAF-------------------------------- 271
ZmPIP2;1    DKPWDDHWIFW-VGP----LVGAAIAAFYHQYILRA-GAIKALGSFRSNA---------- 290
SbPIP2;7    DKPWDDHWIFW-VGP----FVGAAIAAFYHQYILRA-GAIKALGSFRSNA---------- 290
PvPIP2;6    DKPWDDHWIFW-VGP----FVGAAIAAFYHQYILRA-GAIKALGSFRSNA---------- 290
PvPIP2;7    DKPWDDHWIFW-VGP----FVGAAIAAFYHQYILRA-GAIKALGSFRSNA---------- 290
ZmPIP2;2    DKPWDDHWIFW-VGP----LLGAAIAAFYHQYILRA-GAIKALGSFRSNA---------- 292
SiPIP2;5    DKPWDDHWIFW-VGP----FAGAAIAAFYHQYILRA-GAIKALGSFRSNA---------- 289
OsPIP2;1    EKAWHNHWIFW-VGP----FVGAAIAAFYHQYILRA-GAIKALGSFRSNA---------- 290
BdPIP2;3    DKAWDDQWIFW-VGP----MVGAAIAAFYHQYILRA-GAIKALGSFRSNA---------- 290
ZmPIP2;3    DKAWDDQWIFW-VGP----LIGAAIAAAYHQYVLRA-SATKLG-SYRSNA---------- 289
ZmPIP2;4    DKAWDDQWIFW-VGP----LIGAAIAAAYHQYVLRA-SATKLG-SYRSNA---------- 288
PvPIP2;1    DKAWDDQWIFW-VGP----LIGAAIAAAYHQYVLRA-SAAKLG-SYRSNA---------- 288
SiPIP2;4    DKAWDDQWIFW-VGP----LIGAAIAAAYHQYVLRA-SAAKLG-SFRSNA---------- 288
SbPIP2;5    DKAWDDQWIFW-VGP----LIGAAIAAAYHQYVLRA-SASKLGSSYRSNA---------- 289
OsPIP2;2    DKAWDDQWIFW-VGP----LIGAAIAAAYHQYVLRA-SAAKLG-SYRSNA---------- 288
```

```
                                     TM6
ZmPIP2;5    DKAWDDHWIFW-VGP----FIGAAIAAAYHQYVLRA-SAAKLG-SSASFSR--------- 284
SbPIP2;6    DKTWDDHWIFW-VGP----FIGAAIAAAYHQYVLRA-SAAKLG-SSASFSR--------- 292
SiPIP2;3    DKAWDDHWIFW-VGP----FIGAAIAAAYHQYVLRA-SASKLG-SSASFSR--------- 290
OsPIP2;3    HKAWHDHWIFW-VGP----LIGAAIAAAYHQYVLRA-SAAKLG-SSSSFRG--------- 290
BdPIP2;2    DKAWDDQWIFW-VGP----FIGAAIAAAYHQYVLRA-SATKLG-SSASFGRN-------- 287
BdPIP2;1    DKAWDDQWIFW-VGP----LIGAAIAAAYHQYVLRA-SAAKLG-SYRSSS---------- 288
PvPIP2;2    DKAWDDQVR----------------TLAYWRSFQS------------------------ 264
PvPIP2;3    DKPWDDHVRSI-I------RSARIIHDASHLTSHSS-PTCIGALSSDLMVRFCHLLFFL- 297
PvPIP2;4    DKAWDDQVR----------------PRQLMTTLRP-SPR-------------------- 266
PvPIP2;5    DKAWDDHVR----------------LRQHLVLLLL-IVKALMASCQRN----------- 277
SbPIP2;1    SKAWSDQWIFW-VGP----FIGAAIAALYHQIVLRA-SARGYG-SFRSNA---------- 286
SbPIP2;2    SKAWSDQWIFW-VGP----FIGAAIAALYHQIVLRA-SARGHG-SFRSNA---------- 286
ZmPIP2;6    SKAWSDQWIFW-VGP----FIGAAIAALYHQIVLRA-SARGYG-SFRSNA---------- 288
SbPIP2;3    SKAWSDQWIFW-VGP----FIGAAIAALYHQIVLRA-SARGYG-SFRSNA---------- 286
SbPIP2;4    SKAWSDQWIFW-VGP----FIGAAIAALYHQIVLRA-SARGHG-SFRSNA---------- 286
PvPIP2;8    SKAWSDQWIFW-VGP----FIGAAIAALYHQIVLRA-SARGYG-SFRSNA---------- 286
PvPIP2;9    SKAWSDQWIFW-VGP----FIGAAIAALYHQIVLRA-SARGYG-SFRSNA---------- 286
SiPIP2;1    NKAWSDQWIFW-VGP----FIGAAIAALYHQIVLRA-SARGYG-SFRSNA---------- 286
SiPIP2;2    NKAWSDQWIFW-VGP----FIGAAIAALYHQIVLRA-SARGYG-SFRSNS---------- 286
OsPIP2;4    NKAWSDQWIFW-VGP----FIGAAIAALYHQVILRA-SARGYG-SFRSNA---------- 286
BdPIP2;4    DKAWSDQWIFW-VGP----FIGAAIAALYHQTVLRA-SARGYG-SFRSNA---------- 289
OsPIP2;5    SKAWSDQWIFW-VGP----FIGAAIAALYHQIVLRA-SARGYG-SFRSNA---------- 283
AtPIP2;2    SKPWDDHWIFW-VGP----FIGAAIAAFYHQFVLRA-SGSKSLGSFRSAANV-------- 285
AtPIP2;3    SKPWDDHWIFW-VGP----FIGATIAAFYHQFVLRA-SGSKSLGSFRSAANV-------- 285
AtPIP2;1    SKPWDDHWIFW-VGP----FIGAAIAAFYHQFVLRA-SGSKSLGSFRSAANV-------- 287
AtPIP2;4    EKAWDDQWIFW-VGP----MIGAAAAAFYHQFILRA-AAIKALGSFGSFGSFRSFA---- 291
GmPIP2;7    EKAWDDQWIFW-VGP----FIGAAIAAFYHQSVLRA-QAAKALGSFRSSSNL-------- 286
GmPIP2;8    EKAWDDQWIFW-VGP----FIGAALAAFYHQSVLRA-QAAKALGSFRSSSNL-------- 285
AtPIP2;5    DKAWDHHWIFW-VGP----FAGAAIAAFYHQFVLRA-GAIKALGSFRSQPHV-------- 286
AtPIP2;6    QKAWDDQWIFW-VGP----FVGAAIAAFYHQFVLRA-GAMKAYGSVRSQLHELHA----- 289
PpPIP2;2    SKPWNDHWIYW-VGP----FLGAALAAAYHQYVLRA-GPFKSLGSFRSAPSHI------- 279
PpPIP2;3    SKPWDDHWIFW-VGP----FVGAALAAAYHQYVLRA-GPFKQLGSFRSAPSRV------- 280
PpPIP2;1    SKPWDDHWIFW-VGP----FLGAALAASYHQYILRA-APFKSLGSFRSAPSHV------- 279
PpPIP2;4    SKSWDDHWIFW-VGP----FLGAALAAAYHQYILRA-NPIKSMRSFGNGSNHT------- 280
SmPIP2;1    SVSWDDQWIFW-VGP----FIGAAAAAIYHQYVLRAGSALKALGSFRSNPPHQHHGHP-- 290
SmPIP3;1    SRVWNDQWVYW-VGP----LLGAALAAMYHQYILR------------------------- 266
PpPIP3;1    -------WIFW-VGP----IVGSTCAAIYYTYVLKA-----ASLRFRSLYE--------- 257
PpPIP1;1    NNAWADHWIFWIGP-----MLGAALAAAYHTLVIRA-------LPFRKRV---------- 289
PpPIP1;2    HNAWADHWIFWVGP-----FIGAALAAAYHTLVIRA-------LPFRKRV---------- 289
PpPIP1;3    DQAWNDHWIFWVGP-----ILGATLAAMYHTLVIRA-------IPFSANRA--------- 286
```

```
                              TM6
SmPIP1;1    DQAWDDHWIFWVGP-----LIGAALAAFYHQFVIRA-------IPFH------------- 281
ZmPIP1;1    HHAWADHWIFWVGP-----FIGAALAAIYHQVIIRA-------IPFKSRS---------- 287
SbPIP1;1    NHAWSDHWIFWVGP-----FIGAALAAIYHQVIIRA-------IPFKSRS---------- 288
ZmPIP1;3    DHAWSDHWIFWVGP-----FIGAALAAIYHQVIIRA-------IPFKSRS---------- 292
ZmPIP1;4    DHAWSDHWIFWVGP-----FIGAALAAIYHQVIIRA-------IPFKSRS---------- 292
SbPIP1;2    DHAWSDHWIFWVGP-----FIGAALAAIYHQVIIRA-------IPFKSRS---------- 289
ZmPIP1;2    DHAWNDHWIFWVGP-----FIGAALAAIYHQVIIRA-------IPFKSRS---------- 289
PvPIP1;1    DHAWDDHWIFWVGP-----FIGAALAAIYHQVIIRA-------IPFKSRS---------- 288
PvPIP1;2    DHAWNDHWIFWVGP-----FIGAALAAIYHQVIIRA-------IPFKSRS---------- 288
OsPIP1;1    DHAWNDHWIFWVGP-----FVGAALAAIYHQVIIRA-------IPFKSRS---------- 289
PvPIP1;4    EHAWSHHWIFWVGP-----FIGAALAAIYHQVIIRA-------IPFKSRS---------- 289
SiPIP1;1    EHAWSHHWIFWVGP-----FIGAALAAIYHQVIIRA-------IPFKSRS---------- 289
PvPIP1;5    EHAWSHHWIFWVGP-----FIGAALAAIYHQVIIRA-------IPFKSRS---------- 289
SiPIP1;4    RQAWDDHWIFWVGP-----FIGAALAAIYHQVVIRA-------IPFKSRS---------- 288
SiPIP1;2    EHAWSHHVMDLLGRPLHRRCPGCYLPPGDHQGHPVQ-------EQVLSCDGPARRDMPAR 304
SiPIP1;3    RQAWDDHVSSLTEN-------WCNLV---------------------------------- 269
PvPIP1;3    DHAWDDHVSEVDLQ--------SYLALFLFWLEVR------------------------- 277
OsPIP1;4    DHAWNDHWIFWVGP-----FVGAALAAIYHQVIIRA-------IPFKSRS---------- 289
OsPIP1;2    GHAWDDHWIFWVGP-----FIGAALAAIYHQVVIRA-------IPFKSRS---------- 288
OsPIP1;5    GHAGEDHWIFWVGP-----LMGGALVAIYHQGVIRA-------IPFKSRW---------- 291
BdPIP1;1    SHNWADHWIFWVGP-----FIGAALAAVYHQVVIRA-------IPFKTKS---------- 288
ZmPIP1;5    SHAWNDHWIFWVGP-----FIGAALAAIYHVVIIRA-------LPFKSRD---------- 288
PvPIP1;7    SHAWNDHWIFWVGP-----FIGAALAAIYHVVIIRA-------IPFKSRD---------- 288
PvPIP1;6    SHAWNDHWIFWVGP-----FIGAALAAIYHVVIIRA-------IPFKSRD---------- 288
SiPIP1;5    SQAWNDHWIFWVGP-----FIGAALAAIYHVVIIRA-------IPFKSRD---------- 288
SbPIP1;3    SNAWNDHWIFWVGP-----FIGAALAAIYHVVIIRA-------IPFKSRD---------- 290
OsPIP1;3    AHAWHDHWIFWVGP-----FIGAALAAIYHVVVIRA-------IPFKSRD---------- 288
BdPIP1;2    KQAWDDHWIFWVGP-----FIGAALAAIYHVVVIRA-------IPFKSRD---------- 289
GhPIP1;2    DHAWDDHWVFWVGP-----FIGAALAAVYHQIIIRA-------IPFKTRD---------- 287
GhPIP1;5    DHAWDDHWVFWG-------------WTLHWTCTCCS-------LPPDNP----------- 278
PtPIP1;3    DHAWDDHWIFWVGP-----FIGAALAAVYHQIVIRA-------IPFKSRA---------- 288
AtPIP1;1    DHSWDDHWVFWVGP-----FIGAALAALYHVVVIRA-------IPFKSRS---------- 286
AtPIP1;2    DNAWDDHWVFWVGP-----FIGAALAALYHVIVIRA-------IPFKSRS---------- 286
AtPIP1;3    DHAWDDHWIFWVGP-----FIGAALAALYHQLVIRA-------IPFKSRS---------- 286
AtPIP1;4    DHSWDDHWIFWVGP-----FIGAALAALYHQIVIRA-------IPFKSKS---------- 295
AtPIP1;5    DHAWDDHWIFWVGP-----FIGAALAALYHQIVIRA-------IPFKSKT---------- 287
PtPIP1;5    DQAWDDHWIFWVGP-----FIGAALASLYHQIVIRA-------IPFKSK----------- 287
GhPIP1;6    DQAWDDHWIFWVGP-----FIGAALAALYHQIVIRA-------IPFKSK----------- 287
GmPIP1;2    DQAWDNHWIFWVGP-----FIGAALAALYHQIVLRA-------IPFKSK----------- 304
PtPIP1;4    DKAWDDHWIFWVGP-----FIGAALASLYHQIVIRA-------IPFKSK----------- 287
```

Figure S7 (B)

```
                                                                      ↓  ↓↓
PvPIP2;10       --------------------------------METVGAKKDYKDPAPAPLVNAGELGKWS 28
PvPIP2;11       -------------------------MNSS-QSMETVGAKKDYKDPAPAPLVNAGELGKWS 34
SiPIP2;8        -------------------------MNPS-ESMEAAGGKKDYKDPAPAPLVNAGELGKWS 34
SbPIP2;8        -------------------------MDPCHQSIETAGAK-DYSDPPPAPLVNAGELGKWS 34
BdPIP2;7        ---------------MAPSVCT-----ACSNGNGNDNNGKDYLDPPPALLLDTAELTKWS 40
OsPIP2;8        ----------------------------MAAGSGSGSNPKDYQDPPPAPLVDTGELGKWS 32
BdPIP2;8        ---------------MTPDTDSNNKVAKPSAGDDTEIPSKDYLNPPPTPLFDGSELGKWS 45
PvPIP2;12       -------------MAADQEIIMQQ-QRE-EHGGGGESSRRDYTDPPPQPVVATSELRRWS 45
SiPIP2;6        -------------MAVGHEIVQQQRQRDPEHGGGGESSGKDYTDPPPQPVLTASELRRWS 47
GhPIP1;1        --------MEGKEEDVRLGANKFTERQPIGTAAQSQDDGKDYTEPPPAPFFEPGELTSWS 52
GhPIP1;11       --------MEGKEEDVRLGANKFTERQPIGTAAQTQDDGKDYSEPPPAPFFEPGELTSWS 52
GhPIP1;7        --------MEGKEEDVRLGANKFSERQPIGTAAQSQDD-KDYTEPPPAPLFEPSELTSWS 51
GmPIP1;7        --------MEGKEQDVSLGANKFPERQPIGTAAQSQDDGKDYQEPAPAPLVDPTEFTSWS 52
GmPIP1;8        --------MEGKEEDVSLGANKFSERQPIGTAAQSQDDGKDYTEPPPAPLFEPSELTSWS 52
PtPIP1;1        --------MEGKEEDVRLGANKFNERQPLGTAAQSQDD-KDYKEPPPAPLFEPSELTSWS 51
PtPIP1;2        --------MEGKEEDVRLGANRFNERQPIGTAAQSLDD-KDYKEPPPAPLFEPGELTSWS 51
GmPIP1;1        --------MEGKEEDVRVGANRYGERQPIGTAAQA----KDYREPPSAPLFEPGELSSWS 48
GmPIP1;3        --------ME-REEDVKVGAQKFSERQALGTGAKS---DKDYKEAPPAPLFEPGELKSWS 48
GmPIP1;4        --------ME-REEDVKVGAQKFSERQALGTGAQG---DKDYKEAPPAPLFEPGELKSWS 48
GmPIP1;5        --------MESKEEDVRVGATKFSERQPIGTAAQG---DKDYKEPPPAPLFEPGELKSWS 49
GmPIP1;6        --------MESKEEDVNVGANKFSERQPIGTAAQGGG-DKDYKEAPPAPLFEPGELKSWS 51
GhPIP1;3        --------MEGKEEDVKLGANKFSERQPIGTSAQT---DKDYKEPPPAPLFEPGELKSWS 49
GhPIP1;13       --------MEGKEEDVKLGANKFSERQPIGTSAQT---DKDYKEPPPAPLFEPGELKSWS 49
GhPIP1;4        --------MEGKEEDVKLGANKFSERQPIGTSAQT---DKDYKEPPPAPLFEPGELKSWS 49
GhPIP1;12       --------MEGKEEDVKLGANKFSERQPIGTSAQT---DKDYKEPPPAPLFEPGELKSWS 49
SiPIP1;6        MAGGKLQDRFQDDEDVRVGVDRFPERHPIGATAADDLG-RDYTEPPPAPLFDAAELSSWS 59
ZmPIP1;6        MAGGTLQDRS-EEEDVRVGVDRFPERQPIG-TAADDLG-RDYSEPPAAPLFEASELSSWS 57
SbPIP1;4        MAGG---NRS-QDEDVRVGVDRFPERQPIGTTAADDLGGRDYSEPPAAPLFESSELSSWS 56
GhPIP2;4        ---------------MSKEVSEE-GQGR-----------KDYVDPPPAPLIDMAELKSWS 33
GhPIP2;11       ---------------MSKEVSEE-GQGR-----------KDYVDPPPAPLIDMAELKSWS 33
GhPIP2;9        ---------------MSKEVSEE-GQGR-----------KDYVDPPPAPLIDMAELKSWS 33
PtPIP2;2        ---------------MSKEVSEV-GQTHG----------KDYVDPPPAPLLDLGELKLWS 34
PtPIP2;1        ---------------MSKDVIEE-GQTHT----------KDYVDPPPAPLFDVGELKLWS 34
AtPIP2;7        --------MSKEVSEEGKTH-----------------HGKDYVDPPPAPLLDMGELKSWS 35
AtPIP2;8        --------MSKEVSEEGR-------------------HGKDYVDPPPAPLLDMAELKLWS 33
GmPIP2;1        ---------------MSKEVSQ----QR-----------KDYVDPPPAPLIDLAEIKLWS 30
GmPIP2;2        ---------------MSKEVSQE-GLQR-----------KDYVDPPPAPLFDLAEIKLWS 33
OsPIP2;6        ---------------MSKEVSEEPEHVRP----------KDYTDPPPAPLFDVGELRLWS 35
PtPIP2;9        ---------------MSTG---------G----------KDYRDPPPAPLLDMEELKQWS 26
                                    ↓↓↓
PtPIP2;10       ---------------MSSEERNI-ERQHG----------RDYHDPPPAPLLDMGELKQWS 34
PvPIP2;13       ---------------MPKE------DVSIEAAAAE---KAPYWDPPPAPVLDTSELQKWS 36
PvPIP2;14       ---------------MPKEDVSIEEDVSIEAAAAE---KAPYWDPPPAPVLDTSELGKWS 42
```

```
SiPIP2;7     ---------------MPIEDVS---IETTEAAGPQ---KVPYWDPPPAPLLETSELMKWS 39
SbPIP2;9     ---------------MSKD------DVTAEAEAAA---KAPYWDPPPAPLLDTSELKKWS 36
OsPIP2;7     --------------MASKEEVA--VETVEGGAAAA---KAPYWDPPPAPLLDTSELGKWS 41
BdPIP2;5     ---------------MSKEEVA---AADTSEQPIT---KAPYWDPPPAPLLDTSELSRWS 39
BdPIP2;6     -------------MPNTKEEVA---AAKLAKQATL---TAPYWDPP-AELLDRSELSRWS 40
                                                    * :..   ..   *:  **

                   TM1                                  TM2
PvPIP2;10    LYRAVIAEFVATLLFVYVALATVIGHKRQDEAQPCGGVGV--LGIAWSFGGMIFVLVYCT 86
PvPIP2;11    LYRAVIAEFVATLLFVYVALATVIGHKRQDEAQTCGGVGA--LGIAWSFGGMIFVLVYCT 92
SiPIP2;8     LYRAVITEFVATLLFVYVTLATVIGHKRQSESQPCGGAGV--LGIAWSFGGMIFVLVYCT 92
SbPIP2;8     LYRAAIAEFVATLLFVYVTLATVIGHKRQAESQPCGSVGV--LGIAWSFGGMIFVLVYCT 92
BdPIP2;7     LYRAAIAEFTATLLFVYIAIATVIGHNHQSSTACSSGAGI--LGIAWSFGGTIFVLVYST 98
OsPIP2;8     LYRAAIAEFTATLLLVCISVSTVIGEKRQSG---EGGAGV--LGIAWAFGGLIFVLVYCT 87
BdPIP2;8     LYRAVIAEFTATLLFVYVTVATVIGHKRQQQQPDTAGAGVGLLGIAWAFGGSIAVLVYCT 105
PvPIP2;12    LYRAAIAEFVATLLFLYAAVATVIGHKRQTESDASGCGGVGVLGIAWAFGGMIFLLVYCT 105
SiPIP2;6     LYRAAIAEFVATLLFLYLTVATVIGYKRQAESDASGCGGVGVLGIAWAFGGMIFLLVYCA 107
GhPIP1;1     FYRAGIAEFVATFLFLYITILTVMGVVKEK--TKCPTVGI--QGIAWAFGGMIFALVYCT 108
GhPIP1;11    FYRAGIAEFVATFLFLYITILTVMGVVKEK--TKCPTVGI--QGIAWAFGGMIFALVYCT 108
GhPIP1;7     FYRAGIAEFVATFLFLYISVLTVMGVLKDK--TKCTTVGI--QGIAWAFGGMIFALVYCT 107
GmPIP1;7     FYRAGIAEFVATFLFLYITVLTVMGVAGAK--SKCSTVGI--QGIAWAFGGMIFALVYCT 108
GmPIP1;8     FYRAGIAEFVATFLFLYITILTVMGVNRSS--SKCATVGI--QGIAWAFGGMIFALVYCT 108
PtPIP1;1     FYRAGIAEFMATFLFLYITVLTVMGVFKDT--TKCTTVGI--QGIAWAFGGMIFALVYCT 107
PtPIP1;2     FYRAGIAEFMATFLFLYITVLTVMGVVKDQ--TKCTTVGI--QGIAWAFGGMIFALVYCT 107
GmPIP1;1     FYRAGIAEFVATFLFLYITVLTVMGVFKSK--SKCSTVGI--QGIAWAFGGMIFALVYST 104
GmPIP1;3     FYRAGIAEFVATFLFLYITVLTVMGVNRAP--NKCSSVGI--QGIAWAFGGMIFALVYCT 104
GmPIP1;4     FYRAGIAEFVATFLFLYITVLTVMGVNRAP--NKCSSVGI--QGIAWAFGGMIFALVDCT 104
GmPIP1;5     FYRAGIAEFVATFLFLYITILTVMGVNRSP--SKCASVGI--QGIAWAFGGMIFALVYCT 105
GmPIP1;6     FYRAGIAEFVATFLFLYITILTVMGVNRSP--SKCASVGI--QGIAWAFGGMIFALVYCT 107
GhPIP1;3     FYRAGIAEFVATFLFLYITVLTVMGVSQSK--TKCTTVGI--QGIAWAFGGMIFALVYCT 105
GhPIP1;13    FYRAGIAEFVATFLFLYITVLTVMGVSQSK--TKCTTVGI--QGIAWAFGGMIFALVYCT 105
GhPIP1;4     FYRAGIAEFVATFLFLYITVLTVMGVSQSK--TKCTTVGI--QGIAWAFGGMIFALVYCT 105
GhPIP1;12    FYRAGIAEFVATFLFLYITVLTVMGVSQSK--TKCTTVGI--QGIAWAFGGMIFALVYCT 105
SiPIP1;6     FYRAGIAEFVATFLFLYVTVLAVMGVSNSP--SKCGTVGV--QGIAWAFGGMIFALVYCT 115
ZmPIP1;6     FYRAGIAEFVATFLFLYVTVLTVMGVSKSP--SKCGTVGI--QGIAWAFGGMIFALVYCT 113
SbPIP1;4     FYRAGIAEFVATFLFLYVTVLTVMGVSKSP--SKCGTVGI--QGIAWAFGGMIFALVYCT 112
GhPIP2;4     FYRA--------LLFLYVTVATVIGHKKQQ--DACDGVGL--LGIAWAFGGMIFILVYCT 81
GhPIP2;11    FYRALIAEFIATLLFLYVTVATVIGHKKQQ--DACDGVGL--LGIAWAFGGMIFILVYCT 89
GhPIP2;9     FYRALIAEFIATLLFLYVTVATVIGHKKQQ--DACDGVGL--LGIAWAFGGMIFILVYCT 89
PtPIP2;2     FYRALIAEFIATLLFLYVTVATVIGHKSNK--DPCDGVGL--LGIAWAFGGMIFILVYCT 90
PtPIP2;1     FFRALIAEFIATLLFLYVTVATVIGHKKNQ--DACGGVGL--LGIAWAFGGMIFILVYCT 90
AtPIP2;7     FYRALIAEFIATLLFLYVTVATVIGHKKQT--GP-CDGVGL-LGIAWAFGGMIFVLVYCT 90
AtPIP2;8     FYRAIIAEFIATLLFLYVTVATVIGHKNQT--GP-CGGVGL-LGIAWAFGGMIFVLVYCT 88
GmPIP2;1     FYRALIAEFIATLLFLYVTVATVIGHKKQT--GPCDGVGL--LGIAWAFGGMIFVLVYCT 86
GmPIP2;2     FYRALIAEFIASLLFLYVTVATIIGHKKQT--GPCDGVGL--LGIAWSFGGMIFVLVYCT 89
```

```
                           TM1                                  TM2
                      ↓  ↓↓  ↓↓   ↓  ↓  ↓             ↓             ↓ ↓↓
OsPIP2;6       FYRALIAEFIATLLFLYITVATVIGYKVQSSADQCGGVGT--LGIAWAFGGMIFILVYCT 93
PtPIP2;9       FYRALIAEFVATFLFLYIGVGTVVGYKG-VHNNLCDGAGY--LGVAWAFGGMIFVLVYCT 83
PtPIP2;10      FYRAAIAEFIATFLFLFFSVSTVVNYKEPNYTDQCSRVGH--LGIAWANGGMIFVLVYCT 92
PvPIP2;13      LYRALIAEFVATLIFLYVSIATVIGYKAQS-NNLQTCTGVGFLGVSWSFGATIFILVYCT 95
PvPIP2;14      LYRALIAEFVATLIFLYVSIATVIGYKAQS-NNLQTCTGVGFLGVAWSFGATIFILVYCT 101
SiPIP2;7       LYRALIAEFVATLIFLYVSIATVIGYKDQS-KALA-CNGVGFLGVAWSFGATIFILVYCI 97
SbPIP2;9       LYRALIAEFMATLIFLYVSIATVIGYKNQS-KAES-CTGVGALGIAWSFGATIFILVYCT 94
OsPIP2;7       LYRALIAEFMATLIFLYVSIATVIGYKNQR-ATVDACTGVGYLGVAWSFGATIFVLVYCT 100
BdPIP2;5       LYRAAIAEFTATFIFLYVSIATVIGYKSQA-AAET-CTGVGYLGVAWSFGATIFVLVYCT 97
BdPIP2;6       LYRAVIAEFMATLIFLYISIATVIGYKSQA-AAQA-CTGVGYLGVAWSFGATIFVLVHCT 98
               ::**        ::::   : ::::.            *    *::*: *. *  ** .

                                                   TM3
               ↓ ↓↓ ↓↓↓↓  ↓↓↓ ↓  ↓↓↓↓  ↓    ↓   ↓   ↓                 ↓  ↓↓
PvPIP2;10      AGISGGHVNPAVTFALLLARKVSLVRAVLYVVAQCLGAICGAGLVRAFHG--GANYLRFG 144
PvPIP2;11      AGVSGGHVNPAVTFGLLLALKVSLVRAVLYVVAQCLGAICGAGLVRAFHG--GPNYLRYG 150
SiPIP2;8       AGVSGGHVNPAVTFGLLLARKVSLVRAALYIVAQCLGAICGAGLVRAFHG--TSSYLRHG 150
SbPIP2;8       AGISGGHINPAVTFGLLLARKVSLVRAALYVVAQCLGAMCGAGLVKAFHG--AHWYLHYG 150
BdPIP2;7       AGISGGHINPAVTFALLLARKLTLLRAVFYIVSQCVGAIFGAAIARSVHG--RARYLLHG 156
OsPIP2;8       AGISGGHMNPAVTFAMVLARRVSLPRAALYTMAQCVGAVCGAGLARAMHG--GGQYARHG 145
BdPIP2;8       AGISGGHINPAVTFGLLLARKVSLPRAGLYMLAQCLGAICGAGLVRTVNG--GDAYLKHG 163
PvPIP2;12      AGTSGGPINPAGTFALLLARKVSLPRAALYVAAQCFGAVCGAGLARAVHSSPPGAFARLG 165
SiPIP2;6       AGISGGHINPAVTLALLQARKVSVPRAALYVAAQCLGAVCGAGLVRAIHS--PDAFVRLG 165
GhPIP1;1       AGISGGHINPAVTFGLFLARKLSLTRAIFYMVMQCLGAICGAGVVKGFMG--KTQYGMLG 166
GhPIP1;11      AGISGGHINPAVTFGLFLARKLSLTRAIFYMVMQCLGAICGAGVVKGFMG--KTQYGMLG 166
GhPIP1;7       AGISGGHINPAVTFGLFLGRKLSLTRAIFYMVMQCLGAICGAGVVKGFMG--KTRYGALG 165
GmPIP1;7       AGISGGHINPAVTFGLFLARKLSLPRAIFYIVMQCLGAICGAGVVKGFEG--KTKYGALN 166
GmPIP1;8       AGISGGHINPAVTFGLFLARKLSLTRALFYMVMQVLGAIVGAGVVKGFEG--KTFYGQHN 166
PtPIP1;1       AGISGGHINPAVTFGLFLARKLSLTRAVFYMLMQCLGAICGAGVVKGFYG--KKNYELLN 165
PtPIP1;2       AGISGGHINPAVTFGLFLARKLSLTRAVFYMVMQCLGAICGAGVVKGFYG--KTNYELHN 165
GmPIP1;1       AGISGGHINPAVTFGLFLARKLSLTRAIFYIIMQCLGAICGAGVVKGFEP---HLYERLG 161
GmPIP1;3       AGISGGHINPAVTFGLFLARKLSLTRAVFYIVMQCLGAICGAGVVKGFEG--NARYELFK 162
GmPIP1;4       AGISGGHINPAVTFGLFLARKLSLTRALFYIVMQCLGAICGAGVVKGFEG--NARYELFK 162
GmPIP1;5       AGISGGHINPAVTFGLFLARKLSLTRALFYIIMQCLGAICGAGVVKGFEG--NARYEMFK 163
GmPIP1;6       AGISGGHINPAVTFGLFLARKLSLTRALFYIIMQCLGAICGAGVVKGFEG--NANYELFK 165
GhPIP1;3       AGISGGHINPAVTFGLLLARKLSLTRAVFYMIMQCLGAICGAGVVKGFEG--DSRYEMLG 163
GhPIP1;13      AGISGGHINPAVTFGLLLARKLSLTRAVFYMIMQCLGAICGAGVVKGFEG--DSRYEMLG 163
GhPIP1;4       AGISGGHINPAVTFGLLLARKLSLTRAVFYMIMQCLGAICGAGVVKGFEG--DSRYEMLG 163
GhPIP1;12      AGISGGHINPAVTFGLLLARKLSLTRAVFYMIMQCLGAICGAGVVKGFEG--DSRYEMLG 163
SiPIP1;6       AGVSGGHINPAVTFGLLLARKLSLPRAGYYAVMQCLGAACGAGVVKALVG--GALYEAAG 173
ZmPIP1;6       AGVSGGHINPAVTFGLLLARKLSLTRAVYYVVMQCLGAVCGAGVVKAFG---SALYESAG 170
SbPIP1;4       AGVSGGHINPAVTFGLLLARKLSLARAVYYVVMQCLGAVCGAGVVKALVG--SALYQSAG 170
GhPIP2;4       AGISGGHINPAVTFGLFLARKVSFIRAVAYMVSQCLGAICGVGLVKAFMK--HPYNSLG- 138
GhPIP2;11      AGISGGHINPAVTFGLFLARKVSFIRAVAYMVSQCLGAICGVGLVKAFMK--HPYNSLG- 146
GhPIP2;9       AGISGGHINPAVTFGLFLARKVSLIRAVAYMVSQCLGAICGVGLVKAFMK--HPYNSLG- 146
PtPIP2;2       AGISGGHINPAVTFGLFLARKVSLIRAVAYMVAQCLGAICGVGLVKAFMK--KNYNSLG- 147
```

TM3

```
PtPIP2;1    AGISGGHINPAVTFGLLLARKVSLIRAVGYMVAQCLGAVCGVGLVKAFMK--PYYNSLG- 147
AtPIP2;7    AGISGGHINPAVTFGLFLARKVSLVRALGYMIAQCLGAICGVGFVKAFMK--TPYNTLG 148
AtPIP2;8    AGISGGHINPAVTFGLFLARKVSLPRAVAYMVAQCLGAICGVGLVKAFMM--TPYKRLG 146
GmPIP2;1    AGISGGHINPAVTFGLFLARKVSLIRALFYMVAQCLGAICGVGLVKAFMK--HSYNSLG- 143
GmPIP2;2    AGISGGHINPAVTFGLFLARKVSLIRAVFYMVAQCLGAICGVGLVKAFMK--HSYNSLG- 146
OsPIP2-6    AGISGGHINPAVTFGLLLARKVSVIRAVMYIVAQCLGGIVGVGIVKGIMK--HQYNANG- 150
PtPIP2;9    AGISGGHINPAVTFGLFVARKVSLIRAVAYMMAQCLGAMLGVWMVMILTG--IHYDQAG- 140
PtPIP2;10   SGISGGHLNPAVTFGMLVARKMSLIRAAAYMLAQCLGAILGHLFVFLFMY--ADEQQSSV 150
PvPIP2;13   GGVSGGHINPAVTFGLFVGRKLSLVRTVLYIVAQCLGAICGAGMVKGIVG--GDLYDTLG 153
PvPIP2;14   GGVSGGHINPAVTFGLFVGRKLSLVRTVLYIVAQCLGAICGAGIVKGIVG--GDIYDAYG 159
SiPIP2;7    GGISGGHINPAVTFGLFVGRKLSLLRTVLYIVAQCLGAICGVAIVKGITG---DQYSLLG 154
SbPIP2;9    GGISGGHINPAVTFGLFVGRKLSLVRTLLYIVAQCLGAICGVGIVKGIMK---VPYNSLG 151
OsPIP2;7    GGVSGGHINPAVTLGLFFGRKLSLVRTVLYVVAQCLGAIAGAGIVKGIMK---RPYDALG 157
BdPIP2;5    GGVSGGHINPAVTFGLLVGRKLSLVRAVLYIVAQCLGAICGAGLVKGIT--G-RSYETLG 154
BdPIP2;6    GGLSGGHMNPAVTFGMLVGRKLSLVRASMYIAAQCLGAICGAAIVKAITRHA-HSYEAFG 157
            .* *** :*** *:.:. . :::. *:  *   * .*.  *  ..  .
```

TM4

```
PvPIP2;10   GGANELS--------AGYSKGAGLAAEIVGTFVLVYTVFSATDPKRKVRDTHVPVLAPLP 196
PvPIP2;11   GGANELS--------AGYSKGAGLAAEIVGTFVLVYTVFSATDPKRKVRDTHVPVLAPLP 202
SiPIP2;8    GGANELA--------AGYSKGAGLAAEIVGTFVLVYTVFSATDPKRKVRDTHVPVLAPLP 202
SbPIP2;8    GGANELS--------AGYSKGAGLAAEIVGTFVLVYTVFSATDPKRKVRDSHVPVLAPLP 202
BdPIP2;7    GGANELA--------PGFSAAAGLGAEVVGTFVLVYIVFSATDPKRKARDCHVPVLAPLP 208
OsPIP2;8    GGANELA--------AGYSAGAGVVAEMVGTFVLVYTVFSATDPKRKARDSHVPVLAPLP 197
BdPIP2;8    GGANEVA--------DGYSNAAGFVAEVVGTFVLVYTVFSATDPKRMARDSHISVLAPLL 215
PvPIP2;12   GGANVVG--------NGYSRGTGLAAEAVGTFVLVYTVFSATDAKRSARDSHIPMLAPRP 217
SiPIP2;6    GGANAVG--------DGYGRGTGLAAEVVGTFVLVYTVFSATDAKRNARDSHIPVLAPLP 217
GhPIP1;1    GGANSVA--------HGYTKGDGLGAEIVGTFVLVYTVFSATDAKRSARDSHVPILAPLP 218
GhPIP1;11   GGANSVA--------HGYTKGDGLGAEIVGTFVLVYTVFSATDAKRSARDSHVPILAPLP 218
GhPIP1;7    GGANSVN--------HGYTKGDGLGAEIVGTFVLVYTVFSATDAKRSARDSHVPILAPLP 217
GmPIP1;7    GGANFVA--------PGYTKGDGLGAEIVGTFILVYTVFSATDAKRSARDSHVPILAPLP 218
GmPIP1;8    GGANFVA--------PGYTKGDGLGAEIVGTFILVYTVFSATDAKRSARDSHVPILAPLP 218
PtPIP1;1    GGANMVS--------PGYTKGDGLGAEIVGTFVLVYTVFSATDAKRSARDSHVPILAPLP 217
PtPIP1;2    GGANMVA--------HGYTKGDGLGAEIVGTFILVYTVFSATDAKRSARDSHVPILAPLP 217
GmPIP1;1    GGANTIA--------KGYTNSAGLGAEIVGTFVLVYTVFSATDAKRNARDSHVPILAPLP 213
GmPIP1;3    GGANFVS--------HGYTKGDGLGAEIVGTFILVYTVFSATDAKRNARDSHVPILAPLP 214
GmPIP1;4    GGANFVS--------HGYTKGDGLGAEIVGTFILVYTVFSATDAKRNARDSHVPILAPLP 214
GmPIP1;5    GGANFVN--------SGYTKGDGLGAEIVGTFVLVYTVFSATDAKRNARDSHVPILAPLP 215
GmPIP1;6    GGANFVN--------SGYTKGDGLGAEIVGTFVLVYTVFSATDAKRNARDSHVPILAPLP 217
GhPIP1;3    GGANVVN--------HGYTKGDGLGAEIIGTFVLVYTVFSATDAKRNARDSHVPILAPLP 215
GhPIP1;13   GGANVVN--------HGYTKGDGLGAEIIGTFVLVYTVFSATDAKRNARDSHVPILAPLP 215
GhPIP1;4    GGANVVN--------HGYTKGDGLGAEIVGTFILVYTVFSATDAKRNARDSHVPILAPLP 215
GhPIP1;12   GGANVVN--------HGYTKGDGLGAEIVGTFILVYTVFSATDAKRNARDSHVPILAPLP 215
SiPIP1;6    GGANAVN--------PGYTKGDGLGAEIVGTFVLVYTVFSATDAKRSARDSHVPVLAPLP 225
ZmPIP1;6    GGANAVS--------PGYTKGDGLGAEVVGTFVLVYTVFSATDAKRTARDSHVPALAPLP 222
```

TM4

```
SbPIP1;4     GGANAVA--------PGYTKGDGLGAEIVGTFVLVYTVFSATDAKRSARDSHVPVLAPLP 222
GhPIP2;4     GGANTVA--------SGYNKGTALGAEIIGTFVLVYTVFSATDPKRSARDSHVPVLAPLP 190
GhPIP2;11    GGANTVA--------SGYNKGTALGAEIIGTFVLVYTVFSATDPKRSARDSHVPVLAPLP 198
GhPIP2;9     GGANTVA--------SGYNNGTALGAEIIGTFVLVYTVFSATDPKRSARDSHVPVLAPLP 198
PtPIP2;2     GGANTVA--------MGYNTGTALGAEIIGTFVLVYTVFSATDPKRSARDSHVPVLAPLP 199
PtPIP2;1     GGANMVA--------PGYSTGTAVGAEIIGTFVLVYTVFSATDPKRSARDSHIPVLAPLP 199
AtPIP2;7     -GGANTVA-------DGYSKGTALGAEIIGTFVLVYTVFSATDPKRSARDSHIPVLAPLP 199
AtPIP2;8     -GGANTVA-------DGYSTGTALGAEIIGTFVLVYTVFSATDPKRSARDSHVPVLAPLP 197
GmPIP2;1     GGANSVS--------AGYNKGSALGAEIIGTFVLVYTVFSATDPKRSARDSHIPVLAPLP 195
GmPIP2;2     GGANSVS--------AGYNKGSALGAEIIGTFVLVYTVFSATDPKRSARDSHVPVLAPLP 198
OsPIP2;6     GGANMVA--------SGYSTGTALGAEIIGTFVLVYTVFSATDPKRNARDSHVPVLAPLP 202
PtPIP2;9     GAVNVVA--------PGYSKGTALGAEIIGTFVLVYTVLAATDPKRMARDSHVPVLAPLP 192
PtPIP2;10    GVVNVVS--------RNYSKGAGLGAEFIGTFVLVYTVFSATDPKRNARDSHVPVLAPLP 202
PvPIP2;13    GGANAVG--------SGFSVAAALGAEIAGTFVLVYTVFSATDPKRTARDSFIPVLVPLP 205
PvPIP2;14    GGAT-----------RGFSVAAALGAEIAGTFVLVYTVFSATDPKRTARDSFIPVLLPLP 208
SiPIP2;7     GGANSVA--------DGFSVVAGLGAEIMGTFVLVYTVFSATDPKRTARDSFIPVLVPLP 206
SbPIP2;9     GGANAVATGPSDGVLTGYSVGSALAAEIVGTFILVYTVFSATDPKRTARDSFIPVLVPLP 211
OsPIP2;7     GGANTVS--------DGYSAAGALGAEIVGTFILVYTVFSATDPKRTARDSFIPVLVPLP 209
BdPIP2;5     GGANSVS--------DGFSVGAAFGAEIAGTFVLVYTVLSATDPKRTARDSFIPVLVPLP 206
BdPIP2;6     GGANTVA--------DGFTVGGAIGAEIVGTFVLVYTVFSSTDPKRTARDSFVPVLQPLP 209
             *  ..            .:    .. **   ***:***  *:::**.**  .**  .:.  * *
```

TM5 **TM6**

```
PvPIP2;10    IGFAVFMVHLATIPVTGTGINPARSLGPAVVY--N-QRKAWEDHWVFWVGPLIGAAAAMV 253
PvPIP2;11    IGFAVFMVHLATIPVTGTGINPARSLGPAVVY--N-QRKAWEDHWIFWVGPLIGAAAAMV 259
SiPIP2;8     IGFAVFMVHLATIPITGTGINPARSLGPAVVY--N-QRKAWEDHWMFWVGPLIGSAAAMV 259
SbPIP2;8     IGFAVFMVHLATIPVTGTGINPARSLGPAVVY--N-QRKAWEDQWIFWVGPLIGAAAAMI 259
BdPIP2;7     IGFAVFMVHLATIPITGTGINPARSLGAAVAY--N-GDKAWSEHWIFWVGPLLGAALAMV 265
OsPIP2;8     IGLAVLVVHLATIPITGTGINPARSLGPALVLGLG-TTKAWSHLWIFWVGPFAGAAAAMI 256
BdPIP2;8     IGFSVFVVHLATIPVTGTSINPARSFGPAVVY--N-GEKAWADLWIFWVGPFTGAAVAMV 272
PvPIP2;12    IGFAVFVVHLATIPITGTGINPARSFGAAVVY--N-QARAWHDQWIFRVGPLIGAAIATL 274
SiPIP2;6     IGFAVFVVHLATIPITGTGINPARSFGAAVVY--N-QARAWQDQWIFWVGPLTGAAMATL 274
GhPIP1;1     IGFAVFLVHLATIPITGTGINPARSLGAAIIFN---KDKGWDDHWIFWVGPFIGAALAAL 275
GhPIP1;11    IGFAVFLVHLATIPITGTGINPARSLGAAIIFN---KDKGWDDHWIFWVGPFIGAALAAL 275
GhPIP1;7     IGFAVFLVHLATIPITGTGINPARSLGAAIIFN---KDKGWGGHWIFWVGPFIGAALAAL 274
GmPIP1;7     IGFAVFLVHLATIPITGTGINPARSLGAAIIFN---KDLGWDEHWIFWVGPFIGAALAAL 275
GmPIP1;8     IGFAVFLVHLATIPITGTGINPARSLGAAIIFN---KDLGWDDHWIFWVGPFVGAALAAL 275
PtPIP1;1     IGFAVFLVHLATIPITGTGINPARSLGAAIIFN---KDKAWDDHWIFWVGPFIGAALAAL 274
PtPIP1;2     IGFAVFLVHLATIPITGTGINPARSLGAAIIFN---KDSAWDDHWIFWVGPFIGAALAAL 274
GmPIP1;1     IGFAVFLVHLATIPVTGTGINPARSLGAAIIFN---KDQAWDDHWIFWVGPFIGAALAAL 270
GmPIP1;3     IGFAVFLVHLATIPITGTGINPARSLGAAIIYN---RDHAWDDHWIFWVGPFIGAALAAV 271
GmPIP1;4     IGFAVFLVHLATIPITGTGINPARSLGAAIIYN---RDHAWDDHWIFWVGPFIGAALAAL 271
GmPIP1;5     IGFAVFLVHLATIPITGTGINPARSLGAAIIYN---RDHAWDDQWIFWVGPFIGAALAAV 272
GmPIP1;6     IGFAVFLVHLATIPITGTGINPARSLGAAIIYN---RDHAWDDQWIFWVGPFIGAALAAV 274
```

```
                              TM5                                          TM6

GhPIP1;3        IGFAVFLVHLATIPITGTGINPARSLGAAIIYN---KDHAWDDHWIFWVGPFIGAALAAI 272
GhPIP1;13       IGFAVFLVHLATIPITGTGINPARSLGAAIIYN---KDHAWDDHWIFWLGPFIGP----- 267
GhPIP1;4        IGFAVFFVHLATIPITGTGINPARSLGAAIIYN---KDHAWDDHWIFWVGPFIGAALAAI 272
GhPIP1;12       IGFAVFLVHLATIPITGTGINPARSLGAAIIYN---KDHAWDDHWIFWVGPLHWT----- 267
SiPIP1;6        IGLAVFLVHLATIPITGTGINPARSLGAAIIYD---RPHGWHGHWIFWVGPFTGAALAAV 282
ZmPIP1;6        IGFAVFLVHLATIPITGTGINPARSLGAAIIYD---NPHGWHGHWIFWVGPFAGAALAAV 279
SbPIP1;4        IGFAVFLVHLATIPITGTGINPARSLGAAIIYD---SPHGWHGHWIFWVGPFAGAALAAV 279
GhPIP2;4        IGFAVFMVHLATIPITGTGINPARSFGAAVIYN---NDKAWDEHWIFWVGPFVGALAAAI 247
GhPIP2;11       IGFAVFMVHLATIPITGTGINPARSFGAAVIYN---NDKAWDEHWIFWVGPFVGALAAAI 255
GhPIP2;9        IGFAVFMVHLATIPITGTGINPARSFGAAVIYN---NDKAWDDHWIFWVGPFVGALAAAI 255
PtPIP2;2        IGFAVFMVHLATIPITGTGINPARSFGAAVIFN---NEKAWDDHWIFWVGPFVGALAAAA 256
PtPIP2;1        IGFAVFMVHLATIPITGTGINPARSFGAAVIIN---DKKAWDDHWIFWVGPFVGALAAAA 256
AtPIP2;7        IGFAVFMVHLATIPITGTGINPARSFGAAVIYN---NEKAWDDQWIFWVGPFLGALAAA- 256
AtPIP2;8        IGFAVFMVHLATIPITGTGINPARSFGAAVIYN---NEKAWDDHWIFWVGPFVGALAAA- 254
GmPIP2;1        IGFAVFMVHLATIPITGTGINPARSFGAAVIYN---NGKVWDDHWIFWVGPFVGALAAAA 252
GmPIP2;2        IGFAVFMVHLATIPITGTGINPARSLGAAVIYN---NGKVWDEHWIFWVGPLVGALAAAA 255
OsPIP2;6        IGFAVFMVHLATIPITGTGINPARSIGAAVIYN---QKKAWDDHWIFWAGPFIGALAAAA 259
PtPIP2;9        IGFAVFVVHLALIPITGTGINPARSLGAAVVKN---AKEIWDDHWIFWVGPFVGALAAAV 249
PtPIP2;10       IGFAVFVVHLATIPITGTGINPARSLATNLLHR--STAEAMDDLWIFWVGPFLGALAAAV 260
PvPIP2;13       IGFAVFVVHLATIPITGTGINPARSLGAAVMYN-----KAWSYHWIFWIGPAIGATAAAL 260
PvPIP2;14       IGFAVFVVHLATIPITGTGINPARSLGAAVVYD-----MAWKNHWIFWIGPAIGATAAAM 263
SiPIP2;7        IGFAVFVVHLATIPITGTGINPARSLGAAVIFG-----EAWKNHWIFWVGPLIGATAAAL 261
SbPIP2;9        IGFAVFVVHLATIPITGTGINPARSLGAAVVH------AAWKDHWIFWVGPLIGATVAAL 265
OsPIP2;7        IGFAVFVVHLATIPITGTGINPARSLGAAVLYN---QHAAWKDHWIFWVGPVIGAFLAAA 266
BdPIP2;5        IGFAVFVVHLATIPISGTGINPARSLGAAVMYN---QHKAWKDHWIFWVGPLLGATVAAF 263
BdPIP2;6        IGFAVFVVHLATIPVTGTGINPARSLGAAVMYN---QHIAWKHHWIFWVGPFVGATVAAL 266
                **::*:.**** **::**.******:.. :            *:*   **    .

PvPIP2;10       YHQLVLRAGAAKAFASWRNSNHI-------- 276
PvPIP2;11       YHQLVLRAGAAKAFVSWRNSNHI-------- 282
SiPIP2;8        YHQLVLRAGAAKAFASWRNNNHTGI------ 284
SbPIP2;8        YHQLVLRAGAAKALASLRNNYHI-------- 282
BdPIP2;7        YHEFVLRGKAMASFRRSNYTAAAAY------ 290
OsPIP2;8        YHHYILRGAAAKAFASSSYRSPHF------- 280
BdPIP2;8        YHQFVLRNTVSVFFHRSDYAAAV-------- 295
PvPIP2;12       YHEHVLRASALKALGSFKAG----------- 294
SiPIP2;6        YHEHVLRASAIKALGSFKAG----------- 294
GhPIP1;1        YHVVVIRAIPFKSK----------------- 289
GhPIP1;11       YHVVVIRAIPFKSK----------------- 289
GhPIP1;7        YHVVVIRAIPFKSK----------------- 288
GmPIP1;7        YHQVVIRAIPFKSK----------------- 289
GmPIP1;8        YHQVVIRAIPFKSS----------------- 289
PtPIP1;1        YHQVVIRAIPFKK------------------ 287
PtPIP1;2        YHQVVIRAIPFKK------------------ 287
GmPIP1;1        YHQIVIRAIPFSSK----------------- 284
```

```
GmPIP1;3        YHQIVIRAIPFKTRG---------------- 286
GmPIP1;4        YHQIVIRAIPFKTRG---------------- 286
GmPIP1;5        YHQIVIRAIPFKTRA---------------- 287
GmPIP1;6        YHQIVIRAIPFKTRA---------------- 289
GhPIP1;3        YHQIIIRAIPFKTRA---------------- 287
GhPIP1;13       -------------------------------
GhPIP1;4        YHQIIIRGIPFKTRA---------------- 287
GhPIP1;12       -------------------------------
SiPIP1;6        YHQVVIRAIPFKSSAHY-------------- 299
ZmPIP1;6        YHQVVLRAIPFKSSAHY-------------- 296
SbPIP1;4        YHQVVIRAIPFKSSAHY-------------- 296
GhPIP2;4        YHQYILRATAIKALGSFR------SNPTN-- 270
GhPIP2;11       YHQYILRAAAIKALGSFR------SNPTN-- 278
GhPIP2;9        YHQYILRAAAIKALGSFR------SNPTN-- 278
PtPIP2;2        YHQYILRAAAIKALGSFR------SNPAN-- 279
PtPIP2;1        YHQYILRAGAIKALGSFR------SHPTN-- 279
AtPIP2;7        AYHQYILRASAIKALGSFR------SNATN--280
AtPIP2;8        AYHQYILRAAAIKALASFR------SNPTN--278
GmPIP2;1        YHQYILRAAAIKALGSFR------SNPTN-- 275
GmPIP2;2        YHQYILRAGAIKALGSFR------SNPTN-- 278
OsPIP2;6        YHQYILRAAAIKALGSFR------SNPSN-- 282
PtPIP2;9        YHQYILGSGAAKALASFR------SNPTS-- 272
PtPIP2;10       YHKYVLRAGAVKTLKSFRALGSFGSQPPV-- 289
PvPIP2;13       YHKLVLRGEAVKALGSFRSTSATV------- 284
PvPIP2;14       YHKLVLRGEAVKALCSFRSTSATV------- 287
SiPIP2;7        YHKLVLRGEAAKALGSFRSTSATV------- 285
SbPIP2;9        YHKLVLRGEAVKALGSFRSTSATV------- 289
OsPIP2;7        YHKLVLRGEAAKALSSFRSTSVTA------- 290
BdPIP2;5        YHRFVLRGEAAKALGSFRSTGAATART---- 290
BdPIP2;6        YHQCVLRGKAAKALDSFRGTGAATAPQDAGT 297
```
