# Supplementary material for: Genome-Wide Characterization of Major Intrinsic Proteins in Four Grass Plants and Their Non-Aqua Transport Selectivity Profiles with Comparative Perspective
Source: PLoS One. 2016 Jun 21;11(6):e0157735. doi: 10.1371/journal.pone.0157735 (PMC4915720; doi:10.1371/journal.pone.0157735)
Supplement: S9 Fig — Multiple sequence alignment of ammonia transporter TIP2s and TIP4s (A) and ammonia non-transporters (B) of the twelve plants. The conserved pore lining hydrophobic Leu in loop B and P-x-H in loop C are shown in the blue boxes. The description of the figure legend is as for Fig S9. (PDF) [file pone.0157735.s009.pdf]

Figure S9 (A)

```
                                                 TM1
                                     ↓    ↓↓  ↓       ↓  ↓
GhTIP4;1  ------------------MPK-IALGTSQEAAQPDCIKALVVEFITTFLF 31
GhTIP4;2  ------------------MPK-IALGTSQEAAQPDCIKALVVEFITTFLF 31
PtTIP4;1  ------------------MTK-IALGSRHEAAQPDCLKALVVEFVTTFLF 31
AtTIP4;1  ------------------MKK-IELGHHSEAAKPDCIKALIVEFITTFLF 31
PvTIP2;4  ------------------MSGNIAFGRFDDSFSAASLKAYIAEFISTLVF 32
PvTIP2;5  ------------------MSGNIAFGRFDDSFSAASIKAYIAEFISTLVF 32
SiTIP2;4  ------------------MSGNIAFGRFDDSFSAASLKAYVAEFISTLVF 32
SiTIP2;5  ------------------MSGNIAFGRFDDSFSAASLKAYVAEFISTLVF 32
SbTIP2;3  ------------------MSGNIAFGRFDDSFSAASLKAYVAEFISTLVF 32
OsTIP2;2  ------------------MSGNIAFGRFDDSFSAASLKAYVAEFISTLVF 32
PtTIP2;1  ------------------MAG-IAFGRFDDSFSLGSFKAYLAEFISTLLF 31
PtTIP2;2  ------------------MAR-IAFGRFNDSFSLGSLKAYLAEFISTLLF 31
GhTIP2;1  ------------------MAG-IAFGRFDDSFSLGTVKAYLAEFISTLVF 31
GhTIP2;2  ----------------------SARGRFDDSFSLGTVKAYLAEFISTLVF 28
GhTIP2;3  ------------------MAG-IAFGRFDDSFSLGSVKAYLAEFISTLVF 31
GmTIP2;6  ------------------MAG-IAFGSFNDSFSLASIKAYIAEFISTLLF 31
GmTIP2;5  ------------------MAG-IAFGSFNDSVSFASIKAYIAEFISTLLF 31
GmTIP2;7  ------------------MAG-IAFGNFNDSVSFASIKAYIAEFISTLLF 31
GmTIP2;4  ------------------MGG-IAFGRFDDSFSLTSIKAYIAEFHSTLLF 31
GmTIP2;3  ------------------MGG-IAFGRLDDSFSLTSIKAYIAEFHSTLLF 31
AtTIP2;1  ------------------MAG-VAFGSFDDSFSLASLRAYLAEFISTLLF 31
SiTIP2;3  ------------------MVK-LAFGSFGDSFSAASLKAYVAEFIATLLF 31
ZmTIP2;3  ------------------MVK-LAFGSFRDSLSAASLKAYVAEFIATLLF 31
PvTIP2;1  ------------------MVK-LAFGSLGDSFSAASLKAYVAEFIATLLF 31
PvTIP2;2  ------------------MVK-LAFGSLGDSFSAASLKAYVAEFIATLLF 31
SbTIP2;2  ------------------MVK-LAFGSLGDSFSAASLKSYVAEFIATLLF 31
BdTIP2;2  ------------------MVK-LAFGSLGDSFSVTSIRSYVAEFIATLLF 31
PvTIP2;3  ------------------MVK-LAFGGVGDSFSTTSIKAYVAEFIATLLF 31
SiTIP2;2  ------------------MVK-LAFGSVGDSFSTTSIKAYVAEFIATLLF 31
SbTIP2;1  ------------------MVK-LAFGSVGDSFSATSIKAYVSEFIATLLF 31
ZmTIP2;1  ------------------MVK-LAFGSVGDSFSATSIKAYVAEFIATLLF 31
ZmTIP2;2  ------------------MVK-LAFGSVGDSFSVTSIKAYVAEFIATLLF 31
OsTIP2;1  ------------------MVK-LAFGSLGDSFSATSVKAYVAEFIATLLF 31
BdTIP2;1  ------------------MVK-LAFGSCGDSFSATSIKSYVAEFIATLLF 31
AtTIP2;2  ------------------MVK-IEIGSVGDSFSVASLKAYLSEFIATLLF 31
AtTIP2;3  ------------------MVK-IEVGSVGDSFSVSSLKAYLSEFIATLLF 31
PtTIP2;3  ------------------MAK-IAFGSLGDSFSLASLKAYLSEFIATLLF 31
PtTIP2;4  ------------------MVK-IAFGSLGDSFSVGSLKAYLSEFIATLLF 31
GmTIP2;1  ------------------MVK-IALGTLDDSFSAASLKAYFAEFHATLIF 31
GmTIP2;2  ------------------MVK-ITLGTFDDSFGVASLKAYLAEFHATLIF 31
                                     .      .*  . *   * .*
```

```
                                 LA                    TM2
GhTIP4;1   VFVGVGAAMAADESGANALV---GLFAVAVAHALVVGVMISAG-HISGGH 77
GhTIP4;2   VFVGVGAAMAADESGANALV---GLFAVAVAHALVVGVMISAG-HISGGH 77
PtTIP4;1   VFAGVGSAMAADKLTGDALL---GLFVVAVAHAFVVAVMISAG-HISGGH 77
AtTIP4;1   VFAGVGSAMATDSLVGNTLV---GLFAVAVAHAFVVAVMISAG-HISGGH 77
PvTIP2;4   VFAGVGSAIAYTKLSGGAPLDPSGLISVAVCHGFGLFVAVAIGANISGGH 82
PvTIP2;5   VFAGVGSAIAYTKLSGGAPLDPSGLIAVAVCHGFGLFVAVAIGANISGGH 82
SiTIP2;4   VFAGVGSAIAYTKLSGGAPLDAAGLVAVAVCHGFGLFVAVAIGANISGGH 82
SiTIP2;5   VFAGVGSAIAYTKLSGGAPLDAAGLVAVAVCHGFGLFVAVAIGANISGGH 82
SbTIP2;3   VFAGVGSAIAYTKLTGGAPLDPAGLIAVAVCHGFGLFVAVAIGANISGGH 82
OsTIP2;2   VFAGVGSAIAYTKLTGGAPLDPAGLVAVAVCHGFGLFVAVAIGANISGGH 82
PtTIP2;1   VFAGVGSAMAYNKLTGDAALDPAGLVAIAVCHGFALFVAVSVGANISGGH 81
PtTIP2;2   VFAGVGSAMAYNKLTGDAALDPAGLVAIAVCHGFALFVAVAVGANISGGH 81
GhTIP2;1   VFAGVGSAIAYNKLTTDAALDPDGLVAIAVCHGFALFVAVAIGANISGGH 81
GhTIP2;2   VFAGVGSAIAYNKLTTDAALDPDGLVAIAVCHGFALFVAVAIGANISGGH 78
GhTIP2;3   VFAGVGSAIAYNKLTTDAALDPDGLVAIAVCHGFALFVAVAIGANISGGH 81
GmTIP2;6   VFAGVGSAIAYAKLTSDAALDPTGLVAVAICHGFALFVAVSVGANISGGH 81
GmTIP2;5   VFAGVGSAIAYAKLTSDAALDPTGLVAVAICHGFALFVAVSVGANISGGH 81
GmTIP2;7   VFAGVGSAIAYAKLTSDAALDPTGLVAVAICHGFALFVAVSVGANISGGH 81
GmTIP2;4   VFAGVGSAIAYGKLTSDAALDPAGLLAVAICHGFALFVAVSVGANISGGH 81
GmTIP2;3   VFAGVGSAIAYGKLTSDAALDPAGLLAVAICHGFALFVAVSVGANISGGH 81
AtTIP2;1   VFAGVGSAIAYAKLTSDAALDTPGLVAIAVCHGFALFVAVAIGANISGGH 81
SiTIP2;3   VFAGVGSAIAYSQLTKGGALDPAGLVAIAIAHAFALFVGVSMAANISGGH 81
ZmTIP2;3   VFAGVGSAIAYSQLTKGGALDPAGLVAIAIAHAFALFVGVSMAANISGGH 81
PvTIP2;1   VFAGVGSAIAYSQLTKGGALDPAGLVAIAIAHAFALFVGVSMAANISGGH 81
PvTIP2;2   VFAGVGSAIAYSQLTKGGALDPAGLVAIAIAHAFALFVGVSMAANISGGH 81
SbTIP2;2   VFAGVGSAIAYSQLTKGGALDPAGLVAIAIAHAFALFVGVSMAANVSGGH 81
BdTIP2;2   VFAGVGSAIAYGQLTKGGALDPSGLVAIAIAHAFALFVGVAMAANISGGH 81
PvTIP2;3   VFAGVGSAIAYGQLTHGGALDPAGLVAIAIAHALALFVGVSIAANISGGH 81
SiTIP2;2   VFAGVGSAIAYGQLSHGGALDASGLVAIAIAHALALFVGVSVAANISGGH 81
SbTIP2;1   VFAGVGSAIAFGQLTNDGALDPAGLVAIAIAHALALFVGVSIAANISGGH 81
ZmTIP2;1   VFAGVGSAIAYGQLTNGGALDPAGLVAIAIAHALALFVGVSVAANISGGH 81
ZmTIP2;2   VFAGVGSAIAFGQLTNGGALDPAGLVAIAVAHALALFVGVSVAANTSGGH 81
OsTIP2;1   VFAGVGSAIAYGQLTNGGALDPAGLVAIAIAHALALFVGVSVAANISGGH 81
BdTIP2;1   VFAGVGSAIAYGKLTDDGALDPAGLVAVAIAHAFALFVGVAIAANISGGH 81
AtTIP2;2   VFAGVGSALAFAKLTSDAALDPAGLVAVAVAHAFALFVGVSIAANISGGH 81
AtTIP2;3   VFAGVGSAVAFAKLTSDGALDPAGLVAIAIAHAFALFVGVSIAANISGGH 81
PtTIP2;3   VFAGVGSAIAYSKLTTDAALDPPGLVAVAVAHAFALFVGVSIAANISGGH 81
PtTIP2;4   VFAGVGSAIAYSKLTTDAALDPPGLVAVAVAHAFALFVGVSIAANISGGH 81
GmTIP2;1   VFAGVGSAIAYNELTKDAALDPTGLVAVAVAHAFALFVGVSVAANISGGH 81
GmTIP2;2   VFAGVGSAIAYNELTKDAALDPTGLVAVAVAHAFALFVGVSVAANISGGH 81
           **.* .: :.  .          *  :*:  .: : * :    * ****
```

```
                        LB                        TM3
GhTIP4;1    LNPAVTLGLLFGGHITVVRAILYWIDQLLASSAACILLKYLTGGLN---T 124
GhTIP4;2    LNPAVTLGLLFGGHITVVRAILYWIDQLLASSAACILLKYLTGGLN---T 124
PtTIP4;1    LNPAVTIGLLFGGHITVVRSILYWIDQLLASTAACFLLKYLTGGLA---T 124
AtTIP4;1    LNPAVTLGLLLGGHISVFRAFLYWIDQLLASSAACFLLSYLTGGMG---T 124
PvTIP2;4    VNPAVTFGLALGGQITILTGIFYWIAQLLGAIVGAVLVQFSTGVA----T 128
PvTIP2;5    VNPAVTFGLALGGQITILTGIFYWIAQLLGAIVGAVLVQFSTGVA----T 128
SiTIP2;4    VNPAVTFGLALGGQITILTGLFYWIAQLLGAIVGAVLVQYSTGVVRQQAT 132
SiTIP2;5    VNPAVTFGLALGGQITILTGLFYWIAQLLGAIVGAVLVQYSTGVA----T 128
SbTIP2;3    VNPAVTFGLALGGQITILTGIFYWIAQLLGAIVGAVLVQYSTGVA----T 128
OsTIP2;2    VNPAVTFGLALGGQITILTGVFYWIAQLLGAIVGAVLVQFCTGVA----T 128
PtTIP2;1    VNPAVTFGLALGGQITILTGIFYWIAQLLGSIVACYLLKVATGGLA---V 128
PtTIP2;2    VNPAVTLGLALGGQMTILTGIFYWIAQLLGSIVACYLLKVVTGGLA---V 128
GhTIP2;1    VNPAVTFGLALGGQITILTGIFYWIAQLLGSIVACFLLKAVTGGLT---V 128
GhTIP2;2    VNPAVTFGLALGGQITILTGIFYWIAQLLGSIVACFLLKAVTGGLT---V 125
GhTIP2;3    VNPAVTFGLALGGQITILTGIFYWIAQLLGSIVACFLLKAVTGGLT---V 128
GmTIP2;6    VNPAVTFGLALGGHITILTGLFYWIAQLLGSIVASLLLKFVTG-YD---T 127
GmTIP2;5    VNPAVTFGLALGGHITILTGLFYWIAQLLGSIVASLLLKFVTG-YD---T 127
GmTIP2;7    VNPAVTFGLALGGHITILTGLFYWIAQLLGSIVASLLLKFVTG-YD---T 127
GmTIP2;4    VNPAVTFGLALGGHITILTGFFYWIAQLLGSIVACFLLNYVTGGLP---T 128
GmTIP2;3    VNPAVTFGLALGGHITILTGFFYWIAQLLGSIVACFLLNYVTGGLP---T 128
AtTIP2;1    VNPAVTFGLAVGGQITVITGVFYWIAQLLGSTAACFLLKYVTGGLA---V 128
SiTIP2;3    LNPAVTFGLAVGGHITILTGIFYWVAQLLGASVACLLLKFVTHGQA---I 128
ZmTIP2;3    LNPAVTFGLAVGGHITILTGILYWVAQLLGASVACFLLQYVTHGQA---I 128
PvTIP2;1    LNPAVTFGLAVGGHITILTGIFYWVAQLLGASVACLLLQFVTHGKA---I 128
PvTIP2;2    LNPAVTFGLAIGGHITILTGIFYWVAQLLGASVACLLLKFVTHGKA---I 128
SbTIP2;2    LNPAVTFGLAVGGHITILTGIFYWVAQVLGASVACLLLKYVTHGQA---I 128
BdTIP2;2    LNPAVTFGLAVGGHITILTGIFYWVAQLLGASVACLLLQFVTHTQA---M 128
PvTIP2;3    LNPAVTFGLAVGGHITILTGLFYWVAQLLGASVACLLLKFVTHGKA---I 128
SiTIP2;2    LNPAVTFGLAVGGHITILTGLFYWVAQLLGASVACLLLKFVTHGKA---I 128
SbTIP2;1    LNPAVTFGLAVGGHITILTGIFYWVAQLLGASVACLLLKFVTHGKA---I 128
ZmTIP2;1    LNPAVTFGLAVGGHITILTGVFYWVAQLLGATVACLLLGFVTHGKA---I 128
ZmTIP2;2    LNPAVTFGLAVGGHITVLTGLFYWVAQLLGASVACLLLRFVTHGKA---I 128
OsTIP2;1    LNPAVTFGLAVGGHITILTGLFYWIAQLLGASIACLLLKFVTHGKA---I 128
BdTIP2;1    LNPAVTFGLAVGGNITILTGIFYWVAQLLGATVACFLLKFVTHGKA---I 128
AtTIP2;2    LNPAVTLGLAVGGNITVITGFFYWIAQCLGSIVACLLLVFVTNGES---V 128
AtTIP2;3    LNPAVTLGLAIGGNITLITGFFYWIAQCLGSIVACLLLVFVTNGKS---V 128
PtTIP2;3    LNPAVTFGLAIGGNITFLTGLLYWIAQCLGSIVACLLLKVVTSAEG---I 128
PtTIP2;4    LNPAVTFGLAIGGNITILTGLLYWIAQCLGSIAACLLLKFATSAES---I 128
GmTIP2;1    LNPAVTFGLAIGGNITLITGFLYWIAQLLGSIVACLLLNLIT-AKS---I 127
GmTIP2;2    LNPAVTFGLAIGGNITLITGFLYWIAQLLGSIVACLLLNFIT-AKS---I 127
            .*****:.*    .*...     * : * *.:  ..  :
```

```
          LC                TM4              LD
GhTIP4;1  PVHTLASGMGFLQGVIWEIILTFALLFTVYATIVDPKKGSIDGLGPMLTG 174
GhTIP4;2  PVHTLASGMGFLQGVIWEIILTFALLFTVYATIVDPKKGSIDGLGPMLTG 174
PtTIP4;1  PVHTLASGMDYLQGVVWEIVLTFSLLFTVYATIVDPKKGSIDGLGPMLTG 174
AtTIP4;1  PVHTLASGVSYTQGIIWEIILTFSLLFTVYATIVDPKKGSLDGFGPLLTG 174
PvTIP2;4  PTHGLS-GIGAFEGVVMEIIVTFGLVYTVYATAADPKKGSLGTIAPIAIG 177
PvTIP2;5  PTHGLS-GIGAFEGVVMEIIVTFGLVYTVYATAADPKKGSLGTIAPIAIG 177
SiTIP2;4  PTHGLS-GIGALEGVVMEIIVTFGLVYTVYATAADPKKGSLGTIAPIAIG 181
SiTIP2;5  PTHGLS-GIGALEGVVMEIIVTFGLVYTVYATAADPKKGSLGTIAPIAIG 177
SbTIP2;3  PTHGLS-GIGAFEGVVMEIIVTFGLVYTVYATAADPKKGSLGTIAPIAIG 177
OsTIP2;2  PTHGLS-GVGAFEGVVMEIIVTFGLVYTVYATAADPKKGSLGTIAPIAIG 177
PtTIP2;1  PIHSVAAGVGAIEGVVMEIIITFALVYTVYATAADPKKGSLGTIAPIAIG 178
PtTIP2;2  PIHSVAAGVGAIEGVVMEIIITFALVYTVYATAADPKKGSLGTIAPIAIG 178
GhTIP2;1  PIHGLGAGVGAIQGVVMEIIITFALVYTVYATAADPKKGSLGTIAPIAIG 178
GhTIP2;2  PIHGLGAGVGAIQGVVMEIIITFALVYTVYATAADPKKGSLGTIAPIAIG 175
GhTIP2;3  PIHGLGAGVGAIQGVVMEIIITFALVYTVYATAADPKKGSLGTIAPIAIG 178
GmTIP2;6  PIHSVAAGVGAGEGVVTEIIITFGLVYTVYATAADPKKGSLGTIAPIAIG 177
GmTIP2;5  PIHSVAAGIGAGEGVVTEIIITFGLVYTVYATAADPKKGSLGTIAPIAIG 177
GmTIP2;7  PIHSVAAGIGAGEGVVTEIIITFGLVYTVYATTADPKKGSLGTIAPIAIG 177
GmTIP2;4  PIHSVASGVGAVEGVVTEIIITFGLVYTVYATAADPKKGSLGIIAPIAIG 178
GmTIP2;3  PIHSVASGVGAVEGVVTEIIITFGLVYTVYATAADPKKGSLGTIAPIAIG 178
AtTIP2-1  PTHSVAAGLGSIEGVVMEIIITFALVYTVYATAADPKKGSLGTIAPLAIG 178
SiTIP2;3  PTHGVSG-ISEVEGVVMEIVITFALVYTVYATAADPKKGSLGTIAPMAIG 177
ZmTIP2;3  PTHGVSG-ISEIEGVVMEIVITFALVYTVYATAADPKKGSLGTIAPMAIG 177
PvTIP2;1  PTHGVSG-ISEIEGVVMEIVITFALVYTVYATAADPKKGSLGTIAPIAIG 177
PvTIP2;2  PTHGVSG-ISEIEGVVMEIVITFALVYTVYATAADPKKGSLGTIAPMAIG 177
SbTIP2;2  PTHGVSG-ISEIEGVVMEIVITFALVYTVYATAADPKKGSLGTIAPIAIG 177
BdTIP2;2  PRHAVAG-ISEMEGVVMEIVITFALVYTVYATAADPKKGSLGTIAPIAIG 177
PvTIP2;3  PTHGVAG-ISELEGVVFEIIITFALVYTVYATAADPKKGSLGTIAPIAIG 177
SiTIP2;2  PTHGVAG-ISELEGVVFEIIITFALVYTVYATAADPKKGSLGTIAPIAIG 177
SbTIP2;1  PTHGVSG-ISELEGVVFEIIITFALVYTVYATAADPKKGSLGTIAPIAIG 177
ZmTIP2;1  PTHAVAG-ISELEGVVFEVVITFALVYTVYATAADPKKGSLGTIAPIAIG 177
ZmTIP2;2  PTHGVSGGTTELEGVVFEIVITFALVYTVYATAADPKKGSLGTIAPIAIG 178
OsTIP2;1  PTHGVAG-ISELEGVVMEIVITFALVYTVYATAADPKKGSLGTIAPIAIG 177
BdTIP2;1  PTHGVAAGMNELEGVVMEIVITFALVYTVYATAADPKKGSLGTIAPIAIG 178
AtTIP2;2  PTHGVAAGLGAIEGVVMEIVVTFALVYTVYATAADPKKGSLGTIAPIAIG 178
AtTIP2;3  PTHGVSAGLGAVEGVVMEIVVTFALVYTVYATAADPKKGSLGTIAPIAIG 178
PtTIP2;3  PTHGVASGMSAIEGVVMEIVITFALVYTVYATAADPKKGSLGIIAPIAIG 178
PtTIP2;4  PTHGVASGMSAVEGVVMEIVITFALVYTVYATAADPKKGSIGIIAPIAIG 178
GmTIP2;1  PSHSPANGVNDLQAVVFEIVITFGLVYTVYATAVDPKKGSLGIIAPIAIG 177
GmTIP2;2  PSHAPATGVNDFQAVVFEIVITFGLVYTVYATAADPKKGSLGIIAPIAIG 177
          * *         :..: *.::** *::.: .. .* *  *:. :. :  *
```

```
                     TM5            LE                      TM6
GhTIP4;1    FVVGANILAGGAFSGASMNPARSFGPALVSWNWTDHWVYWVGPLIGGGLA 224
GhTIP4;2    FVVGANILAGGAFSGASMNPARSFGPALVSWNWTDHWVYWVGPLIGGGLA 224
PtTIP4;1    FVVGANILAGGAFSGASMNPARSFGPALVSWDWTDHWVYWVGPLIGGGLA 224
AtTIP4;1    FVVGANILAGGAFSGASMNPARSFGPALVSGNWTDHWVYWVGPLIGGGLA 224
PvTIP2;4    FIVGANILVAGPFSGGSMNPARSFGPAVASGDFTNIWIYWVGPLIGGGLA 227
PvTIP2;5    FIVGANILVAGPFSGGSMNPARSFGPAVASGDFTNIWIYWVGPLIGGGLA 227
SiTIP2;4    FIVGANILVAGPFSGGSMNPARSFGPAVASGDFTNIWIYWVGPLVGGGLA 231
SiTIP2;5    FIVGANILVAGPFSGGSMNPARSFGPAVASGDFTNIWIYWVGPLVGGGLA 227
SbTIP2;3    FIVGANILVAGPFSGGSMNPARSFGPAVASGDFTNIWIYWVGPLVGGGLA 227
OsTIP2;2    FIVGANILVAGPFSGGSMNPARSFGPAVASGDYTNIWIYWVGPLVGGGLA 227
PtTIP2;1    FIVGANILAAGPFSGGSMNPARSFGPAVASGDFHDNWIYWAGPLVGGGIA 228
PtTIP2;2    FIVGANILAAGPFSGGSMNPARSFGPAVASGDFHDNWIYWVGPLIGGGLA 228
GhTIP2;1    FIVGANILAAGPFSGGSMNPARSFGPAVASGDFNGIWIYWVGPLIGGGLS 228
GhTIP2;2    FIVGANILAAGPFSGGSMNPARSFGPAVASGDFNGIWIYWVGPLIGGGLS 225
GhTIP2;3    FIVGANILAAGPFSGGSMNPARSFGPAVASGDFNGIWIYWVGPLIGGGLA 228
GmTIP2;6    FIVGANILAAGPFSGGSMNPARSFGPAVVSGDFHDNWIYWVGPLIGGGLA 227
GmTIP2;5    FIVGANILAAGPFSGGSMNPARSFGPAVVSGDFHDNWIYWVGPLIGGGLA 227
GmTIP2;7    FIVGANILAAGPFSGGSMNPARSFGPAVVSGDFHDNWIYWVGTLIGGGLA 227
GmTIP2;4    FIVGANILAAGPFSGGSMNPARSFGPAVVSGDFHDNWIYWVGPLIGGGLA 228
GmTIP2;3    FIVGANILAAGPFSGGSMNPARSFGPAVVSGDFHDNWIYWVGPLIGGGLA 228
AtTIP2-1    LIVGANILAAGPFSGGSMNPARSFGPAVAAGDFSGHWVYWVGPLIGGGLA 228
SiTIP2;3    FIVGANILAAGPFSGGSMNPARSFGPAVAAGNFAGNWVYWVGPLIGGGLA 227
ZmTIP2;3    FIVGANILAAGPFSGGSMNPARSFGPAVAAGNFAGNWVYWVGPLVGGGLA 227
PvTIP2;1    FIVGANILAAGPFSGGSMNPARSFGPAVAAGNFAGNWVYWVGPLIGGGLA 227
PvTIP2;2    FIVGANILAAGPFSGGSMNPARSFGPAVAAGNFAGNWVYWVGPLIGGGLA 227
SbTIP2;2    FIVGANILAAGPFSGGSMNPARSFGPAVAAGNFAGNWVYWVGPLIGGGLA 227
BdTIP2;2    FIVGANILAAGPFSGGSMNPARSFGPAVAAGDFSGHWVYWVGPLIGGGLA 227
PvTIP2;3    FIVGANILAAGPFSGGSMNPARSFGPAVAAGNFAGNWVYWVGPLIGGGLA 227
SiTIP2;2    FIVGANILAAGPFSGGSMNPARSFGPAVAAGNFAGNWVYWVGPLIGGGLA 227
SbTIP2;1    FIVGANILAAGPFSGGSMNPARSFGPAVAAGNFAGNWVYWVGPLIGGGLA 227
ZmTIP2;1    FIVGANILAAGPFSGGSMNPARSFGPAVAAGDFAGNWVYWVGPLVGGGLA 227
ZmTIP2;2    FIVGANILAAGPFSGGSMNPARSFGPAVAAADFAGNWVYWVGPLIGGGLA 228
OsTIP2;1    FIVGANILAAGPFSGGSMNPARSFGPAVAAGNFAGNWVYWVGPLIGGGLA 227
BdTIP2;1    FIVGANILAAGPFSGGSMNPARSFGPAVAAGNFAGNWVYWVGPLIGGGLA 228
AtTIP2;2    FIVGANILAAGPFSGGSMNPARSFGPAVVSGDFSQIWIYWVGPLVGGALA 228
AtTIP2;3    FIVGANILAAGPFSGGSMNPARSFGPAVVSGDLSQIWIYWVGPLVGGALA 228
PtTIP2;3    FIVGANILAAGPFSGGSMNPARSFGPAVVSGDFSQNWIYWLGPLVGGGLA 228
PtTIP2;4    FIVGANILAAGPFSGGSMNPARSFGPAVVSGDFSQNWIYWLGPLIGGGLA 228
GmTIP2;1    FVVGANILAAGPFSGGSMNPARSFGPAVVSGDLAANWIYWVGPLIGGGLA 227
GmTIP2;2    FVVGANILAAGPFSGGSMNPARSFGPAVVSGDFAANWIYWVGPLIGGGLA 227
              :  *** .*..**.*******:***.. :    *:*  ..*:*.  .
```

	↓	
GhTIP4;1	GYIYENFFIVR-THVLLP-QNEAF-------	246
GhTIP4;2	GYIYENFFIVR-THVLLP-QNEAF-------	246
PtTIP4;1	GFIYENFFITR-SHRPLPSEEEPF-------	247
AtTIP4;1	GFIYENVLIDR-PHVPVADDEQPLLN-----	249
PvTIP2;4	GIVYRYIYMCG-DHAPVAGSDF---------	248
PvTIP2;5	GIVYRYIYMCG-DHAPVASSDF---------	248
SiTIP2;4	GIVYRYIYMCG-DHAPVASSDF---------	252
SiTIP2;5	GIVYRYIYMCG-DHAPVASSDF---------	248
SbTIP2;3	GIVYRYIYMCG-DHAPVASSGF---------	248
OsTIP2;2	GLVYRYVYMCG-DHAPVASSEF---------	248
PtTIP2;1	GLIYGNVFIT--DHTPLS-GDF---------	247
PtTIP2;2	GLIYGNLYIT--DHSPSS-YEF---------	247
GhTIP2;1	GLIYGNVFMNS-DHAPLS-NDF---------	248
GhTIP2;2	GLIYGNVFMNS-DHAPLV-NDF---------	245
GhTIP2;3	GLIYGNVFMNS-DHAPLS-NDF---------	248
GmTIP2;6	GLIYTYAFIPT-QHAPLA-TDF---------	247
GmTIP2;5	GLIYTYAFIPT-NHAPLA-TEF---------	247
GmTIP2;7	GLIYTYAFIM---------------------	237
GmTIP2;4	GLIYGNVFIRS-DHAPLS-SEF---------	248
GmTIP2;3	GLIYGNVFIRS-DHAPLS-SEF---------	248
AtTIP2;1	GLIYGNVFMGSSEHVPLASADF---------	250
SiTIP2;3	GLVYGDVFIA--SYQPVGQQE-YP-------	248
ZmTIP2;3	GLVYGDVFIA--SYQPVGQQE-YP-------	248
PvTIP2;1	GLVYGDVFIA--SYQPVGQQE-YP-------	248
PvTIP2;2	GLVYGDVFIA--SYQPVGQQE-YP-------	248
SbTIP2;2	GLVYGDVFIA--SYQPVGQQDQYP-------	249
BdTIP2;2	GLVYGDVFIA--SYQPVAQQE-YP-------	248
PvTIP2;3	GLIYGDVFIGG-SYQQVADQD-YA-------	249
SiTIP2;2	GLIYGDVFIGG-NYQQVADQD-YA-------	249
SbTIP2;1	GLIYGDVFIGG-NYQQVADQD-YA-------	249
ZmTIP2;1	GLVYGDVFIGG-SYQQVADQD-YA-------	249
ZmTIP2;2	GLVYGDVFIGG-SYQQVADQD-YA-------	250
OsTIP2;1	GLVYGDVFIG--SYQPVADQD-YA-------	248
BdTIP2;1	GLVYGDVFIA--SYQPVADQD-YA-------	249
AtTIP2;2	GLIYGDVFIG--SYAPAPTTESYP-------	250
AtTIP2;3	GLIYGDVFIG--SYEAVETREIRV-------	250
PtTIP2;3	GLVYGGIFIG--SYAPAPVSEDYA-------	250
PtTIP2;4	GLVYGDIFIG--SYTAAPVSEDYA-------	250
GmTIP2;1	GLIYGDVFIG--SYAPVPASETYP-------	249
GmTIP2;2	GLIYGDVFIG--SYAAVPASETYP-------	249
	. :*	

Figure S9 (B)

```
PpTIP6;1        ------------------------------------------------------------
PpTIP6;2        ------------------------------------------------------------
PpTIP6;3        ------------------------------------------------------------
PpTIP6;4        ------------------------------------------------------------
PvTIP5;1        ------------------------------------------------------------
PvTIP5;2        ------------------------------------------------------------
SiTIP5;2        ------------------------------------------------------------
BdTIP5;1        ------------------------------------------------------------
OsTIP5;1        ------------------------------------------------------------
ZmTIP5;1        ------------------------------------------------------------
SiTIP5;1        ------------------------------------------------------------
PtTIP5;1        ------------------------------------------------------------
PtTIP5;2        ------------------------------------------------------------
AtTIP5;1        ------------------------------------------------------------
PtTIP1;1        ---------------------------------------------------------MP- 2
PtTIP1;2        ---------------------------------------------------------MA- 2
GhTIP1;2        ---------------------------------------------------------MP- 2
GhTIP1;7        ---------------------------------------------------------MP- 2
PvTIP1;3        ---------------------------------------------------------MP- 2
PvTIP1;4        ------------------------------------------------------------
SiTIP1;1        ---------------------------------------------------------MP- 2
OsTIP1;2        ---------------------------------------------------------MP- 2
ZmTIP1;2        ---------------------------------------------------------MP- 2
SbTIP1;2        ---------------------------------------------------------MP- 2
BdTIP1;2        ---------------------------------------------------------MP- 2
PtTIP1;3        ---------------------------------------------------------MP- 2
PtTIP1;4        ---------------------------------------------------------MP- 2
AtTIP1;3        ---------------------------------------------------------MP- 2
AtTIP1;1        ---------------------------------------------------------MP- 2
AtTIP1;2        ---------------------------------------------------------MP- 2
GhTIP1;1        ---------------------------------------------------------MP- 2
GhTIP1;3        ---------------------------------------------------------MP- 2
GhTIP1;8        ---------------------------------------------------------MP- 2
GhTIP1;4        ---------------------------------------------------------MP- 2
GhTIP1;6        ---------------------------------------------------------GT- 2
GhTIP1;5        ---------------------------------------------------------MP- 2
PtTIP1;5        ---------------------------------------------------------MP- 2
PtTIP1;6        ---------------------------------------------------------MP- 2
PvTIP1;1        ---------------------------------------------------------MP- 2
PvTIP1;2        ---------------------------------------------------------MP- 2
SiTIP1;2        ---------------------------------------------------------MP- 2
OsTIP1;1        ---------------------------------------------------------MP- 2
SbTIP1;1        ---------------------------------------------------------MP- 2
ZmTIP1;1        ---------------------------------------------------------MP- 2
BdTIP1;1        ---------------------------------------------------------MP- 2
```

```
PtTIP1;7        ---------------------------------------------------------MPN 3
PtTIP1;8        ---------------------------------------------------------MRN 3
PvTIP3;1        -----------------------------------------------------MS--TGA 5
PvTIP3;2        -----------------------------------------------------MS--TGA 5
SiTIP3;2        -----------------------------------------------------MS--TGA 5
ZmTIP3;1        -----------------------------------------------------MS--TGV 5
SbTIP3;1        -----------------------------------------------------MS--TGA 5
ZmTIP3;2        -----------------------------------------------------MSTATGV 7
OsTIP3;1        -----------------------------------------------------MS-TAAA 6
BdTIP3;1        -----------------------------------------------------MSTAARS 7
SiTIP3;1        -----------------------------------------------------------M 1
SbTIP3;3        -----------------------------------------------------MN---MI 4
PvTIP3;3        -----------------------------------------------------MLPVPAL 7
SbTIP3;2        -----------------------------------------------------MLPG--R 5
BdTIP3;2        -----------------------------------------------------MLPT--R 5
OsTIP3;2        -----------------------------------------------------MLPG--R 5
AtTIP3;1        ----------------------------------------------------------MA 2
AtTIP3;2        ----------------------------------------------------------MA 2
PtTIP3;1        ------------------------------------------------------------
PtTIP3;2        ------------------------------------------------------------
SmTIP6;1        ------------------------------------------------------------
SmTIP7;1        ------------------------------------------------------------

                                           TM1
                                 ↓  ↓↓↓↓          ↓↓   ↓↓  ↓↓↓ ↓↓  ↓
PpTIP6;1        ----MKIAFGEAD----EASSPDAIKGAVAEFISLFLFVFIGVGSVMAYEK--------- 43
PpTIP6;2        ----MKVAFGEAD----EVSSPDALKGALAEFISLFLFVFIGVGSVMSYEK--------- 43
PpTIP6;3        ---MVKLAFGESD----EASSPDALKGALAEFISLFLFVFIGVGSVMSYEK--------- 44
PpTIP6;4        ---MVKVAFGEAN----EASSADALKGAFAEFLALFLFVFIGVGSVMSYEK--------- 44
PvTIP5;1        ---MASSSSTNLVAHLSRSVSAASLRSYCAEFISTFLFVFTAVGSAISARM--------- 48
PvTIP5;2        ---MASSSSSNLVAHLRRSVSPASFRSYCAEFISTFLFVFTAVGSAISARM--------- 48
SiTIP5;2        ---MASS----LLAKLKRCVSPPSLRSYFAEFISTFLFVFAAVGSAISARM--------- 44
BdTIP5;1        ---MASN----LRAHMKHCFSAASLRSYLAEFVSTFLFVFVAVGSAMSARM--------- 44
OsTIP5;1        ----MAN----ICANMKRCFSPPALRAYFAEFFSTFLFVFIAVGSTISARM--------- 43
ZmTIP5;1        ---MASN---NLLVDLKRCFSAPSLRSYLAEFISTFLFVFTAVGSAISARM--------- 45
SiTIP5;1        ---MASC----VVGCLQQCFSPSALRSYLAEFISTFLFVFATAGSAISARM--------- 44
PtTIP5;1        --KMASTS---LTARFKQSVTPASLRAYLAEFISTFFYVFAVVGSAMASRK--------- 46
PtTIP5;2        --KMAPTS---LTARFQQSVTPASLRAYLAEFISTFFYVFAVVGSAMASRK--------- 46
AtTIP5;1        MRRMIPTS---FSSKFQGVLSMNALRCYVSEFISTFFFVLAAVGSVMSSRK--------- 48
PtTIP1;1        ---ITSIAFG-SPA---EAGQPDALRAALAEFISMLIFVFAGEGSGMAFN---------- 45
PtTIP1;2        ---ITSIAFG-SPA---EVGQSDALKAALAEFISMLIFVFAGEGSGMAFN---------- 45
GhTIP1;2        ---ISRIAVG-SPA---EAGQADALKAALAEFISVLIFVFAGEGSGMAFN---------- 45
GhTIP1;7        ---ISRIAVG-SPA---EAGQADALKAALAEFISVLIFVFAGEGSGMAFN---------- 45
PvTIP1;3        ---VSRIAVG-APG---ELSHPDTAKAAVSEFISVLIFIFAGSGSGMAFS---------- 45
PvTIP1;4        ---MNRIAVG-APG---ELSHPDTAKAAVSEFISVLIFIFAGSGSGMAFS---------- 43
SiTIP1;1        ---VSRISVG-APG---ELSHPDTAKAAVAEFISMLIFVFAGSGSGMAFSKQSSFSSSSS 55
OsTIP1;2        ---VSRIAVG-APG---ELSHPDTAKAAVAEFISMLIFVFAGSGSGMAFS---------- 45
ZmTIP1;2        ---VSRIAVG-APG---ELSHPDTAKAAVAEFISTLIFVFAGSGSGMAFS---------- 45
```

```
                                          TM1
                           ↓ ↓↓  ↓↓          ↓↓   ↓↓   ↓↓↓ ↓↓  ↓
SbTIP1;2      ---VSRIAVG-APG---ELSHPDTAKAAVAEFISMLIFVFAGSGSGMAFS---------- 45
BdTIP1;2      ---VSRIAIG-SPG---ELSHPDTFKAAVAEFISMLIFVFAGSGSGMAFG---------- 45
PtTIP1;3      ---INRIAFG-TPR---EASHPDALRAALAEFISMLIFVFAGSGSGMAFN---------- 45
PtTIP1;4      ---INRIAVG-TPG---EASHPDSLRAALAEFISTLIFVFAGSGSGMAFN---------- 45
AtTIP1;3      ---INRIAIG-TPG---EASRPDAIRAAFAEFFSMVIFVFAGQGSGMAYG---------- 45
AtTIP1;1      ---IRNIAIG-RPD---EATRPDALKAALAEFISTLIFVVAGSGSGMAFN---------- 45
AtTIP1;2      ---TRNIAIGGVQE---EVYHPNALRAALAEFISTLIFVFAGSGSGIAFN---------- 46
GhTIP1;1      ---IRNIAVG-RPE---EATHPDALKAALAEFISTLIFVFAGSGSGMAFN---------- 45
GhTIP1;3      ---IRNIAVG-RPE---EATHPDALKAALAEFISTLIFVFAGSGSGMAFN---------- 45
GhTIP1;8      ---IRNIPVG-RPE---EATHPDALKAALAEFISTLIFVFAGSGSGMAFN---------- 45
GhTIP1;4      ---IRNIAVG-RPE---EATQPDALKAALAEFISTLIFVFAGSGSGMAFN---------- 45
GhTIP1;6      ---SRNIAVG-RPE---EATQPDALKAALAEFISTLIFVFAGSGSGMAFN---------- 45
GhTIP1;5      ---IRNIAVG-RPE---EATHPDALKAALAEFISTLIFVFAGSGSGMAFN---------- 45
PtTIP1;5      ---IRNIAVG-HYR---ETTQPDALKAALAEFISTLIFVFAGEGSGMAFS---------- 45
PtTIP1;6      ---IRNIAVG-HYH---EATQPDALRAALAEFISTLIFVFAGEGSGMAFA---------- 45
PvTIP1;1      ---ISRIAVG-SHQ---EVYHPGALKAAFAEFISTLIFVFAGQGSGMAFS---------- 45
PvTIP1;2      ---ITRIAVG-SHQ---EVYHPGALKAAFAEFISTLIFVFAGQGSGMAFS---------- 45
SiTIP1;2      ---INRIAVG-SHE---EVYHPGALKAAFAEFISTLIFVFAGQGSGMAFS---------- 45
OsTIP1;1      ---IRNIAVG-SHQ---EVYHPGALKAALAEFISTLIFVFAGQGSGMAFS---------- 45
SbTIP1;1      ---ISRIAVG-THH---EVYHPGALKAALAEFISTLIFVFAGQGSGMAFS---------- 45
ZmTIP1;1      ---INRIALG-SHQ---EVYHPGALKAAFAEFISTLIFVFAGQGSGMAFS---------- 45
BdTIP1;1      ---ISRVAVG-SHH---EVYQAGALKAAVAEFISTLIFVFAGQGSGMAFN---------- 45
PtTIP1;7      LIVIDRIAIG-TVA---ADFHPNAFKAALAEFISTLIFVFAGQGSTMAYN---------- 49
PtTIP1;8      FIIIERITIG-RVE---DDFHSNAFKAALAEFISTLIFVFAGQGSTMAYN---------- 49
PvTIP3;1      RPGRQ-FTVGRSE----DATHPDTIRAAISEFIATAIFVFAAEGSVLSLG---------- 50
PvTIP3;2      RPGRR-FTVGRSE----DATHPDTIRAAISEFIATAIFVFAAEGSVLSLG---------- 50
SiTIP3;2      RPGRR-FTVGRSE----DATHPDTIRAAISEFLATAIFVFAAEGSVLSLG---------- 50
ZmTIP3;1      RPGRR-FTVGRSE----DATHPDTIRAAISEFIATAIFVFAAEGSVLSLG---------- 50
SbTIP3;1      RPGRR-FTVGRSE----DATHPDTIRAAISEFIATAIFVFAAEGSVLSLG---------- 50
ZmTIP3;2      RAGRR-FTVGRSE----DATHPDTIRAAISEFIATAIFVFAAEGSVLSLG---------- 52
OsTIP3;1      RPGRR-FTVGRSE----DATHPDTIRAAISEFLATAIFVFAAEGSILSLG---------- 51
BdTIP3;1      TAGRRGFTVGRSE----DATHPDTIRAAISEFLATAIFVFAAEGSILSLG---------- 53
SiTIP3;1      RAGRRRFNVGRLA----TATDPGTLRDAAAELLATAVFVFAAEGATLSFG---------- 47
SbTIP3;3      RSVRRRFTVGHMA----TATDPATLRRAAAELLATAIFVFAAEGATLSLG---------- 50
PvTIP3;3      HPARRADAAGAG------PLLPVATRAAVAEFVATAIFVFAAEGSVYGLW---------- 51
SbTIP3;2      HPPRRSDTAGTR------PLLPDATRAAVAEFVATAMFVFAAEGSVYGLW---------- 49
BdTIP3;2      FPSRRADDAAGP-----EPLMPSTSRAVLSELVATAVFVFAAEGSLYGLW---------- 50
OsTIP3;2      HTPRRADAAAAAAM--EPLVPGATRAALSEFVATAVFVFAAEGSVYGLW---------- 53
AtTIP3;1      TSARRAYGFGRAD----EATHPDSIRATLAEFLSTFVFVFAAEGSILSLD---------- 48
AtTIP3;2      TSARRAYGFGRAD----EATHPDSIRATLAEFLSTFVFVFAGEGSILALD---------- 48
PtTIP3;1      --MPRRYAFGKAD----EATRPDAMRAALAELVSTFIFVFAGEGSILALD---------- 44
PtTIP3;2      --MPRRHAFGRAD----EATHPDSMRAALAEFVSTFVFVFAGEGSVLALD---------- 44
SmTIP6;1      ---MARLTVGGAD----EYRQPDAMKAALAEFIGMFLFVFAGVGSAMAFAK--------- 44
SmTIP7;1      --MLRSWSLGVRDP-----FSPTTLLGCAAEFIGTFLFVFLGCGSVVSSG---------- 43
                                                        .:
```

```
                            LA                  TM2                  LB
PpTIP6;1   -------IHVG------DLDAAGLLMIAIAHGLAIAVLVAATANISGGHVNPAVSLGLAL 90
PpTIP6;2   -------IHVG------DLEAGGLLIIAIAHGLAIAILVAATANISGGHVNPAVSLGLAL 90
PpTIP6;3   -------IHVG------DLEAGGLLMIAIAHGLAIAILVAATANISGGHVNPAVSLGLAL 91
PpTIP6;4   -------IHAG------DMDAAGLLVIAIAHGLAIAVLVSATANISGGHINPAVSLGLAL 91
PvTIP5;1   -------ATPK----GSAPDAASLVATAVAQAFGLFAAVLIAADVSGGHANPAVTFALAL 97
PvTIP5;2   -------ATPK----GSAPDAASLVATAVAQAFGLSAAVLIAADVSGGHANPAVTFALAL 97
SiTIP5;2   -------VTALDG--GTASDAASLVATAVAQAFGLFAAVLIAADVSGGHANPAVTFAFAI 95
BdTIP5;1   -------LTPD-----VTSDASSLVATAVAQSFGLFAAVFIAADVSGGHVNPAVTFALAI 92
OsTIP5;1   -------LTPD-----ETSDASSLMATAVAQAFGLFAAVFIAADVSGGHVNPAVTFAYAI 91
ZmTIP5;1   -------LTTP----DVTSSAGPLVATAVAQAFGLFAAVLIAADVSGGHVNPAVTFAYAI 94
SiTIP5;1   -------LTPD----STSSDASSLVATAVAQAFALFVAVFIAADASGGHANPAVTFAFAI 93
PtTIP5;1   -------LLPD-----AAAVPSSLVIVAIANAFALSSAVYIAANASGGHVNPAVTFGMAV 94
PtTIP5;2   -------LLPD-----AAADPSSLVIVAIANAFALSSAVYIAANASGGHVNPAVTFGMAV 94
AtTIP5;1   -------LMAG-----DVSGPFGVLIPAIANALALSSSVYISWNVSGGHVNPAVTFAMAV 96
PtTIP1;1   ------KLTDNG-----SSTPAGLVAASLAHAFALFVAVSVGANISGGHVNPAVTFGAFI 94
PtTIP1;2   ------KLTDDG-----SSTPAGLVAASLAHAFALFVAVSVGANISGGHVNPAVTFGAFL 94
GhTIP1;2   ------KLTDDG-----SSTPAGLVAAALAHALALFVAVSIGANISGGHVNPAVTFGAFV 94
GhTIP1;7   ------KLTDDD-----SSTPAGLVAAALAHALALFVAVSVGANISGGHVNPAVTFGAFV 94
PvTIP1;3   ------KLTAGG-----ATTPAGLIAASLAYALALIVSVSIGANISGGHANPAVTFGAFV 94
PvTIP1;4   ------KLTAGG-----ATTPAGLIAASLAYALALIVSVSIGANISGGHANPAVTFGAFV 92
SiTIP1;1   SSWCAGKLTAGG-----ATTPSGLIAAALAHALALFVAVAVGANISGGHVNPAVTFGAFV 110
OsTIP1;2   ------KLTDGG-----GTTPSGLIAASLAHALALFVAVAVGANISGGHVNPAVTFGAFV 94
ZmTIP1;2   ------KLTDGG-----AATPAGLIAASLAHALALFVAVSVGANISGGHVNPAVTFGAFV 94
SbTIP1;2   ------KLTTDGGA---SGTPCGLIAASLAHALALFVAVSVGANISGGHVNPAVTFGAFV 96
BdTIP1;2   ------KLTDGG-----ATTPSGLIAAALAHAFALFVAVSVGANISGGHVNPAVTFGAFI 94
PtTIP1;3   ------KLTDNA-----STTPSGLVAAALAHAFALFVAVSVGANISGGHVNPAVTFGALI 94
PtTIP1;4   ------KLTDSA-----STTPAGLVAAALAHAFALFVAVSVGANISGGHVNPAVTFGALI 94
AtTIP1;3   ------KLTGDG-----PATPAGLVAASLSHAFALFVAVSVGANVSGGHVNPAVTFGAFI 94
AtTIP1;1   ------KLTENG-----ATTPSGLVAAAVAHAFGLFVAVSVGANISGGHVNPAVTFGAFI 94
AtTIP1;2   ------KITDNG-----ATTPSGLVAAALAHAFGLFVAVSVGANISGGHVNPAVTFGVLL 95
GhTIP1;1   ------KLTDNG-----ATTPAGLVAAALAHGFGLFVAVSVGANISGGHVNPAVTFGAFV 94
GhTIP1;3   ------KLTDNG-----ATTPAGLVAAALAHGFGLFVAVSVGANISGGHVNPAVTFGAFV 94
GhTIP1;8   ------KLTDNG-----ATTPAGLVAAALAHGFGLFVAVSVGANISGGHVNPAVTFGAFV 94
GhTIP1;4   ------KLTDNG-----ATTPAGLVAASIAHGLGLFVAVSVGANISGGHVNPAVTFGAFV 94
GhTIP1;6   ------KLTDNG-----ATTPAGLVAASIAHGLGLFVAVSVGANISGGHVNPAVTFGAFV 94
GhTIP1;5   ------KLTDGG-----ATTPAGLVAASLAHGFALFVAVSVGANISGGHVNPAVTFGAFV 94
PtTIP1;5   ------KLTDGA-----SNTPAGLIAAAIAHAFALFVAVSVGANISGGHVNPAVTFGAFI 94
PtTIP1;6   ------KLTDGA-----ANTPAGLIAAAIAHAFALFVAVSVGANISGGHVNPAVTFGAFI 94
PvTIP1;1   ------KLSAGG-----ATTPAGLISAAVAHAFALFVAVSVGANISGGHVNPAVTFGAFV 94
PvTIP1;2   ------KLSAGG-----ATTPAGLISAAVAHAFALFVAVSVGANISGGHVNPAVTFGAFV 94
SiTIP1;2   ------KLSPGG-----S-TPTGLIAAAIAHAFALFVAVSVGANISGGHVNPAVTFGAFV 93
OsTIP1;1   ------KLTGGG-----ATTPAGLIAAAVAHAFALFVAVSVGANISGGHVNPAVTFGAFV 94
SbTIP1;1   ------KLTSGG-----ATTPSGLIAAAVAHAFALFVAVSVGANISGGHVNPAVTFGAFV 94
ZmTIP1;1   ------KLTGGG-----PTTPAGLIAAAVAHAFALFVAVSVGANISGGHVNPAVTFGAFV 94
BdTIP1;1   ------KLNAGG-----AATPAGLISAAIAHAFALFVAVSVGANISGGHVNPAVTFGAFL 94
PtTIP1;7   ------KLTSNA-----PTSPAGLIAVALAHAFGLFVAVATSANISGGHCNPAVTFGAFL 98
```

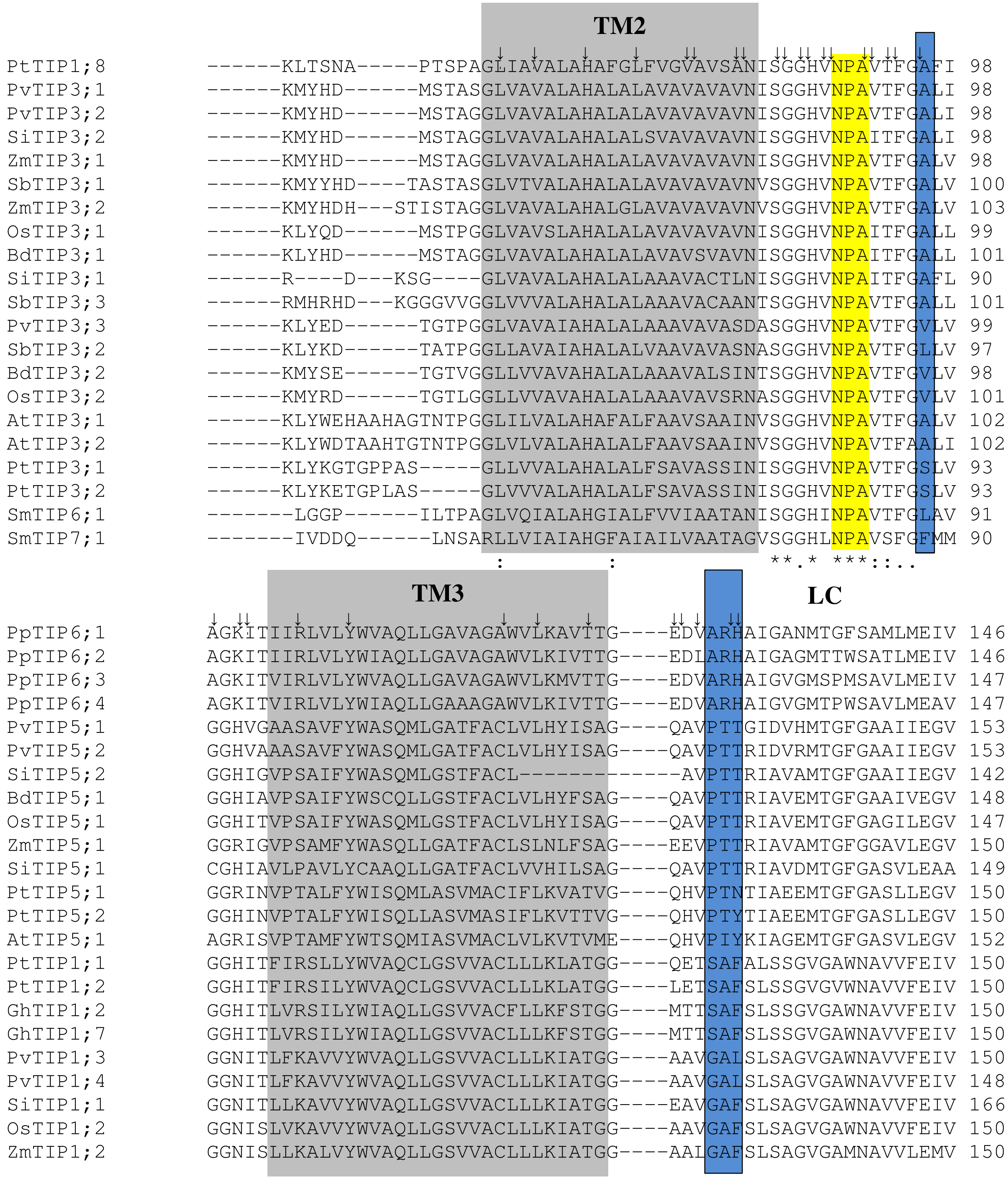
TM2
PtTIP1;8 ------KLTSNA-----PTSPAGLIAVALAHAFGLFVGVAVSANISGGHVNPAVTFGAFI 98
PvTIP3;1 ------KMYHD------MSTASGLVAVALAHALALAVAVAVAVNISGGHVNPAVTFGALI 98
PvTIP3;2 ------KMYHD------MSTAGGLVAVALAHALALAVAVAVAVNISGGHVNPAVTFGALI 98
SiTIP3;2 ------KMYHD------MSTAGGLVAVALAHALALSVAVAVAVNISGGHVNPAITFGALI 98
ZmTIP3;1 ------KMYHD------MSTAGGLVAVALAHALALAVAVAVAVNISGGHVNPAVTFGALV 98
SbTIP3;1 ------KMYYHD----TASTASGLVTVALAHALALAVAVAVAVNVSGGHVNPAVTFGALV 100
ZmTIP3;2 ------KMYHDH---STISTAGGLVAVALAHALGLAVAVAVAVNVSGGHVNPAVTFGALV 103
OsTIP3;1 ------KLYQD------MSTPGGLVAVSLAHALALAVAVAVAVNISGGHVNPAITFGALL 99
BdTIP3;1 ------KLYHD------MSTAGGLVAVALAHALALAVAVSVAVNISGGHVNPAITFGALL 101
SiTIP3;1 ------R----D---KSG----GLVAVALAHALALAAAVACTLNISGGHVNPAITFGAFL 90
SbTIP3;3 ------RMHRHD---KGGGVVGGLVVVALAHALALAAAVACAANTSGGHVNPAVTFGALL 101
PvTIP3;3 ------KLYED------TGTPGGLVAVAIAHALALAAAVAVASDASGGHVNPAVTFGVLV 99
SbTIP3;2 ------KLYKD------TATPGGLLAVAIAHALALVAAVAVASNASGGHVNPAVTFGLLV 97
BdTIP3;2 ------KMYSE------TGTVGGLLVVAVAHALALAAAVALSINTSGGHVNPAVTFGVLV 98
OsTIP3;2 ------KMYRD------TGTLGGLLVVAVAHALALAAAVAVSRNASGGHVNPAVTFGVLV 101
AtTIP3;1 ------KLYWEHAAHAGTNTPGGLILVALAHAFALFAAVSAAINVSGGHVNPAVTFGALV 102
AtTIP3;2 ------KLYWDTAAHTGTNTPGGLVLVALAHALALFAAVSAAINVSGGHVNPAVTFAALI 102
PtTIP3;1 ------KLYKGTGPPAS-----GLLVVALAHALALFSAVASSINISGGHVNPAVTFGSLV 93
PtTIP3;2 ------KLYKETGPLAS-----GLVVVALAHALALFSAVASSINISGGHVNPAVTFGSLV 93
SmTIP6;1 -------LGGP------ILTPAGLVQIALAHGIALFVVIAATANISGGHINPAVTFGLAV 91
SmTIP7;1 -------IVDDQ------LNSARLLVIAIAHGFAIAILVAATAGVSGGHLNPAVSFGFMM 90
: : **.* ***::..
TM3
LC
PpTIP6;1 AGKITIIRLVLYWVAQLLGAVAGAWVLKAVTTG----EDVARHAIGANMTGFSAMLMEIV 146
PpTIP6;2 AGKITIIRLVLYWIAQLLGAVAGAWVLKIVTTG----EDLARHAIGAGMTTWSATLMEIV 146
PpTIP6;3 AGKITVIRLVLYWVAQLLGAVAGAWVLKMVTTG----EDVARHAIGVGMSPMSAVLMEIV 147
PpTIP6;4 AGKITVIRLVLYWIAQLLGAAAGAWVLKIVTTG----EDVARHAIGVGMTPWSAVLMEAV 147
PvTIP5;1 GGHVGAASAVFYWASQMLGATFACLVLHYISAG----QAVPTTGIDVHMTGFGAAIIEGV 153
PvTIP5;2 GGHVAAASAVFYWASQMLGATFACLVLHYISAG----QAVPTTRIDVRMTGFGAAIIEGV 153
SiTIP5;2 GGHIGVPSAIFYWASQMLGSTFACL------------AVPTTRIAVAMTGFGAAIIEGV 142
BdTIP5;1 GGHIAVPSAIFYWSCQLLGSTFACLVLHYFSAG----QAVPTTRIAVEMTGFGAAIVEGV 148
OsTIP5;1 GGHITVPSAIFYWASQMLGSTFACLVLHYISAG----QAVPTTRIAVEMTGFGAGILEGV 147
ZmTIP5;1 GGRIGVPSAMFYWASQLLGATFACLSLNLFSAG----EEVPTTRIAVAMTGFGGAVLEGV 150
SiTIP5;1 CGHIAVLPAVLYCAAQLLGATFACLVVHILSAG----QAVPTTRIAVDMTGFGASVLEAA 149
PtTIP5;1 GGRINVPTALFYWISQMLASVMACIFLKVATVG----QHVPTNTIAEEMTGFGASLLEGV 150
PtTIP5;2 GGHINVPTALFYWISQLLASVMASIFLKVTTVG----QHVPTYTIAEEMTGFGASLLEGV 150
AtTIP5;1 AGRISVPTAMFYWTSQMIASVMACLVLKVTVME----QHVPIYKIAGEMTGFGASVLEGV 152
PtTIP1;1 GGHITFIRSLLYWVAQCLGSVVACLLLKLATGG----QETSAFALSSGVGAWNAVVFEIV 150
PtTIP1;2 GGHITFIRSILYWVAQCLGSVVACLLLKLATGG----LETSAFSLSSGVGVWNAVVFEIV 150
GhTIP1;2 GGHITLVRSILYWIAQLLGSVVACFLLKFSTGG----MTTSAFSLSSGVGAWNAVVFEIV 150
GhTIP1;7 GGHITLVRSILYWIAQLLGSVVACLLLKFSTGG----MTTSAFSLSSGVGAWNAVVFEIV 150
PvTIP1;3 GGNITLFKAVVYWVAQLLGSVVACLLLKIATGG----AAVGALSLSAGVGAWNAVVFEIV 150
PvTIP1;4 GGNITLFKAVVYWVAQLLGSVVACLLLKIATGG----AAVGALSLSAGVGAWNAVVFEIV 148
SiTIP1;1 GGNITLLKAVVYWVAQLLGSVVACLLLKIATGG----EAVGAFSLSAGVGAWNAVVFEIV 166
OsTIP1;2 GGNISLVKAVVYWVAQLLGSVVACLLLKIATGG----AAVGAFSLSAGVGAWNAVVFEIV 150
ZmTIP1;2 GGNISLLKALVYWVAQLLGSVVACLLLKIATGG----AALGAFSLSAGVGAMNAVVLEMV 150

```
                TM3
                ↓ ↓↓   ↓   ↓           ↓  ↓   ↓      ↓↓↓  ↓↓
SbTIP1;2        GGNMSLVKAVVYWVAQLLGSVVACILLKMATGG----AAVGGFSLSAGVGAWNAVVLEMV 152
BdTIP1;2        GGNISLLKAIVYWVAQLLGSTAACLLLQISTGG----ASVGAFSLSAGVGVWNAVVFEIV 150
PtTIP1;3        GGNITLLRSILYWIAQLLGSVVACLLLKFATGG----LETPAFGLSSGVGAWNALVFEIV 150
PtTIP1;4        GGNITLLRSILYWIAQLLGSVVACLLLKFSTGG----LETPAFGLSSGVGAWNAVVFEIV 150
AtTIP1;3        GGNITLLRAILYWIAQLLGAVVACLLLKVSTGG----METAAFSLSYGVTPWNAVVFEIV 150
AtTIP1;1        GGNITLLRGILYWIAQLLGSVVACLILKFATGG----LAVPAFGLSAGVGVLNAFVFEIV 150
AtTIP1;2        GGNITLLRGILYWIAQLLGSVAACFLLSFATGG----EPIPAFGLSAGVGSLNALVFEIV 151
GhTIP1;1        GGNITLLRGILYWIAQLLGSTVACLLLKFATGD----LAVPAFGLSSGVGVSNALVFEIV 150
GhTIP1;3        GGNITLLRGILYWIAQLLGSTVACLLLKFATGD----LAVPAFGLSSGVGVSNALVFEIV 150
GhTIP1;8        GGNITLLRGILYWIAQLLGSTVACLLLKFATGD----LAVPAFGLSSGVGVSNALVFEIV 150
GhTIP1;4        GGNISLLRGILYWIAQLLGSTVACLLLKFATSD----MTVPAFGLSSGVGVWNAVVFEIV 150
GhTIP1;6        GGNISLLRGILYWIAQLLGSTVACLLLKFATSD----MTVPAFGLSSGVGVWNAVVFEIV 150
GhTIP1;5        GGNITLLRGILYWIAQLLGSTVACLLLKFATSG----LGVPAFGLSSGVGALNAFVFEIV 150
PtTIP1;5        GGNITLFRGILYWIAQLLGSTVACLLLKFVTGG----LETSAFALSTGVGVWNAFVLEIV 150
PtTIP1;6        GGNITLLRGILYWIAQLLGSTVACLLLKFTTGG----LETSAFALSSGVGVWNAFVLEIV 150
PvTIP1;1        GGNITLFRGLLYWVAQLLGSTVACLLLRFSTGG----LATGTFGLT-GISVWEALVLEIV 149
PvTIP1;2        GGNITLFRGLLYWVAQLLGSTVACFLLRFSTGG----LATGTFGLT-GISVWEALVLEIV 149
SiTIP1;2        GGNITLFRGILYWIAQLLGSTVACFLLRFSTGG----LPTGTFGLT-GISVWEALVLEIV 148
OsTIP1;1        GGNITLFRGLLYWIAQLLGSTVACFLLRFSTGG----LATGTFGLT-GVSVWEALVLEIV 149
SbTIP1;1        GGNITLFRGLLYWVAQLLGSTVACFLLRFSTGG----LATGTFGLT-GVSVWEAVVMEIV 149
ZmTIP1;1        GGNITLFRGLLYWVAQLLGSTVACFLLRFSTGG----QATGTFGLT-GVSVWEALVLEIV 149
BdTIP1;1        GGNITLFRGLLYWVAQLLGSTAACFLLRFSTGG----LPTGTFGLT-GVSVWEALVLEIV 149
PtTIP1;7        GGNITLLRGILYWIAQLLGSTVACLLLKFTTHY----MTVSVFTLSPGVSVWNAFVFEIV 154
PtTIP1;8        GGNISLLRGILYWIAQLLGSTVACLLLKYTTHH----MTVSVFTLSPGVTVWNAFVFEIV 154
PvTIP3;1        GGRVSLIRAVFYWVAQLLGAVAATLLLRLATGG----ARPPGFALASGVGEWHAVLLEAA 154
PvTIP3;2        GGRVSLIRAVFYWVAQLLGAVAATLLLRLATGG----ARPPGFALASGVGEWHAVLLEAA 154
SiTIP3;2        GGRISLVRAVFYWVAQLLGAIAASLLLRLATGG----MRPPGFALASGVGDWHAVLLEAA 154
ZmTIP3;1        GGRVSLVRAVLYWVAQLLGAVAATLLLRLATGG----MRPPGFALASGVGDWHAVLLEAV 154
SbTIP3;1        GGRISLVRAVFYWVAQLLGAVAATLLLRLATGG----ARPPGFALASGVGDWHAVLLEAA 156
ZmTIP3;2        GGRVSLVRAVLYWAAQLLGAVAATLLLRLATGG----ARPPGFALASGVGDGHAVLLEAV 159
OsTIP3;1        GGRLSLIRALFYWLAQLLGAVVATLLLRLTTGG----MRPPGFALASGVGDWHAVLLEAT 155
BdTIP3;1        GGRITLVRALFYWVAQLLGAIVASLLLRLTTGG----MRPPGFSLAAGVRDWHAVLLEAV 157
SiTIP3;1        GGRICLVRSLVYWAAQLIGAVTAALLLRLATGG----VRLPEYALAGGVSGWHAVVLEAA 146
SbTIP3;3        AGRICLVRSLVYWAAQLLGAVAAALVLRLATGG----MHLPEYALAGCVSGWQAAVLEAA 157
PvTIP3;3        GRRISFGRAAIYWAAQLLGAVAAALLLTLVSGG----TRPVGLGLGRGVHERHALLLEAV 155
SbTIP3;2        GRRISFGRAAVYWLAQLLGAVVASLLLTLVSGG----TRPVGVGLVRGIHERHALLLEAV 153
BdTIP3;2        GRRISFARAVLYWVAQLLGSVLAALLLSLVSGG----ARPMGSALGHGIHERHALLLEVV 154
OsTIP3;2        GRRISFARAALYWAAQLLGAVLAVLLLRLASGG----MRPMGFTLGHRIHERHALLLEVV 157
AtTIP3;1        GGRVTAIRAIYYWIAQLLGAILACLLLRLTTNG----MRPVGFRLASGVGAVNGLVLEII 158
AtTIP3;2        GGRISVIRAIYYWVAQLIGAILACLLLRLATNG----LRPVGFHVASGVSELHGLLMEII 158
PtTIP3;1        GGRISVIRAVSYWVAQLLGSIFAALLLRLVTNG----MIPAGFHVQSEVGEVHGLLLEMA 149
PtTIP3;2        GGRISVIRAVYYWVAQLLGSIVAALLLRLVTNG----MRPVGFHVQSGVGEVHGLLMEMA 149
SmTIP6;1        GGHITIARGVLYWIAQLLGSVLAALVLKFTFLH----EAVPIHAVGAHESVISALVIEIV 147
SmTIP7;1        SGNMSIIKGLMYWISQLAGAVLGAGFYREFPSAIAG--HFGAHAVNSKFTVIEAFGLEAL 148
                  .:       :   *  .:  .                             .   *
```

```
                 TM4                 LD                    TM5
PpTIP6;1   LTFTLMFVVFATAVDP---------NKGTVGVIA------PLAIGFTVLAQIFVGAPFSG 191
PpTIP6;2   LTFTLVFVVFATAVDP---------KKGTVGVIA------PLAIGFTVLAQIFVGAPFSG 191
PpTIP6;3   LTFTLVFVVFATAVDP---------KKGTVGVIA------PLAIGFTVLAQIFVGAPFSG 192
PpTIP6;4   LTFTLVFVVFATAVDP---------KKGTVGVIA------PLAIGFTVLAQIFVGAPFSG 192
PvTIP5;1   LTFMLVYTVHVAGDLRAGGGGGGKRRGFAATALG------ALAVGLVTGACVLSAGSLTG 207
PvTIP5;2   LTFMVVYTVHVAG--------GGKRRGFAAAALG------ALAAGSLTGACVLAAGSLTG 199
SiTIP5;2   LTFMLVYTVHVAGDLR---AAAGGKRGFADTALG------ALAVGLVAGALVLSAGPLTG 193
BdTIP5;1   MTFMLVYAVHVAADPR----ACGRSRGLATTAMG------SLVVGLVAGACVLAAGSLTG 198
OsTIP5;1   LTFMVVYTVHVAGDPRG--GGFGGRKGPAATALG------ALVVGAVTGACVLAAGSLTG 199
ZmTIP5;1   LTFLLVYTVHVVGEREPRSRGGDGKREFAATALG------ALAVGLTQGAFVLAAGALTG 204
SiTIP5;1   ATFMVVYTVHAACDPRR--VRAGGGRSAAETATG------SLAIGLVTGACALATGSLTG 201
PtTIP5;1   MAFGLVYTVYAAGDPR---------RG-SLGAIG------PLAVGLTAGANVLAAGPFSG 194
PtTIP5;2   MTFGLVYTVYAAGDPR---------RS-SLGAIG------PLAVGLMAGANVLAAGPFSG 194
AtTIP5;1   LAFVLVYTVFTASDPR---------RG-LPLAVG------PIFIGFVAGANVLAAGPFSG 196
PtTIP1;1   MTFGLVYTVYATAVDP---------KKGDIGIIA------PIAIGFIVGANILAGGAFDG 195
PtTIP1;2   MTFGLVYTVYATAVDP---------KRGDIGIIA------PIAIGFIVGANILAGGAFDG 195
GhTIP1;2   MTFGLVYTVYATAVDP---------KKGNIGIIA------PIAIGFIVGANILAGGAFDG 195
GhTIP1;7   MTFGLVYTVYATAVDP---------KKGNIGIIA------PIAIGFIVGANILAGGAFDG 195
PvTIP1;3   MTFGLVYTVYATAVDP---------KKGDLGVIA------PIAIGFIVGANILAGGAFDG 195
PvTIP1;4   MTFGLVYTVYATAVDP---------KKGDLGVIA------PIAIGFIVGANILAGGAFDG 193
SiTIP1;1   MTFGLVYTVYATAVDP---------KKGDLGVIA------PIAIGFIVGANILAGGAFDG 211
OsTIP1;2   MTFGLVYTVYATAVDP---------KKGDLGVIA------PIAIGFIVGANILAGGAFDG 195
ZmTIP1;2   MTFGLVYTVYATAVDP---------KKGDLGVIA------PIAIGFIVGANILAGGAFDG 195
SbTIP1;2   MTFGLVYTVYATAVDP---------NKGDLGVIA------PIAIGFIVGANILAGGAFDG 197
BdTIP1;2   MTFGLVYTVYATAVDP---------KRGDLGVIA------PIAIGFIVGANILAGGAFDG 195
PtTIP1;3   MTFGLVYTVYATAVDP---------KKGNLGIIA------PIAIGFIVGANILAGGAFDG 195
PtTIP1;4   MTFGLVYTVYATAVDP---------KKGNLGIIA------PIAIGFIVGANILAGGAFDG 195
AtTIP1;3   MTFGLVYTVYATAVDP---------KKGDIGIIA------PLAIGLIVGANILVGGAFDG 195
AtTIP1;1   MTFGLVYTVYATAIDP---------KNGSLGTIA------PIAIGFIVGANILAGGAFSG 195
AtTIP1;2   MTFGLVYTVYATAVDP---------KNGSLGTIA------PIAIGFIVGANILAGGAFSG 196
GhTIP1;1   MTFGLVYTVYATAVDP---------KKGSLGTIA------PLAIGFIVGANILAGGAFDG 195
GhTIP1;3   MTFGLVYTVYATAVDP---------KKGSLGTIA------PLAIGFIVGANILAGGAFDG 195
GhTIP1;8   MTFGLVYTVYATAVDP---------KKGSLGTIA------PLAIGFIEGANILAGGAFDG 195
GhTIP1;4   MTFGLVYTVYATAIDP---------KKGDLGVIA------PLAIGFIVGANILAGGAFDG 195
GhTIP1;6   MTFGLVYTVYATAIDP---------KKGDLGVIA------PLAIGFIVGANILAGGAFDG 195
GhTIP1;5   MTFGLVYTVYATAVDP---------KRGNLGVIA------PLAIGLIVGANILAGGAFDG 195
PtTIP1;5   MTFGLVYTVYATAIDP---------KKGNLGIIA------PIAIGFIVGANILVGGAFDG 195
PtTIP1;6   MTFGLVYTVYATAVDP---------KKGNLGIIA------PIAIGFIVGANILAGGAFDG 195
PvTIP1;1   MTFGLVYTVYATAVDP---------KKGSLGTIA------PIAIGFIVGANILVGGAFDG 194
PvTIP1;2   MTFGLVYTVYATAVDP---------KKGSLGTIA------PIAIGFIVGANILVGGAFDG 194
SiTIP1;2   MTFGLVYTVYATAVDP---------KKGSLGTIA------PIAIGFIVGANILVGGAFDG 193
OsTIP1;1   MTFGLVYTVYATAVDP---------KKGSLGTIA------PIAIGFIVGANILVGGAFDG 194
SbTIP1;1   MTFGLVYTVYATAVDP---------KKGSLGTIA------PIAIGFIVGANILVGGAFTG 194
ZmTIP1;1   MTFGLVYTVYATAVDP---------KKGSLGTIA------PIAIGFIVGANILVGGAFDG 194
BdTIP1;1   MTFGLVYTVYATAVDP---------KKGSIGTIA------PLAIGFIVGANILVGGAFDG 194
PtTIP1;7   MTFALVYTVYATAIDA---------KKGDVGVIA------PLAIGFVLGANILAGGAFEG 199
PtTIP1;8   MTFALVYTVYATAIDP---------KKGDVGVIA------PLAIGFVLGANILVGGAFEG 199
```

```
                   TM4                                  TM5
PvTIP3;1     MTFGLMYAYYATVIDP----------KRGPVGTIA------PLAVGFLLGANVLAGGPFDG 199
PvTIP3;2     MTFGLMYAYYATVIDP----------KRGAVGTIA------PLAVGFLLGANVLAGGPFDG 199
SiTIP3;2     MTFGLMYAYYATVIDP----------KRGSVGTIG------PLAVGFLLGANVLAGGPFDG 199
ZmTIP3;1     MTFGLMYAYYATVIDP----------KRGHVGTIA------PLAVGFLLGANVLAGGPFDG 199
SbTIP3;1     MTFGLMYAYYATVVDP----------KRGHVGTIA------PLAVGFMLGANVLAGGPFDG 201
ZmTIP3;2     MTFGFVYAYYATVVDP----------KRGHLGTIA------PLAVGFLLGANVLAGGPFDG 204
OsTIP3;1     MTFGLMYAYYATVIDP----------KRGHVGTIA------PLAVGFLLGANMLAGGPFDG 200
BdTIP3;1     MTFGLMYAYYATVMDP----------KRGSVGTIG------PLAVGFLLGANMLAGGPFDG 202
SiTIP3;1     MAFGLMYAYCATAMEP---------RRGRAAGAVA------PLAVGLLAGANVLACGALDG 192
SbTIP3;3     MAFGLMHAYFVTVMDHHT---RRVRAGAGAGAVAA-----PLAVGLLAGANVLACGALEG 209
PvTIP3;3     MTFGLMYAVYATAVDH-----------RSRGGGGGVGAIAPLAIGFVLGANILAGGPFDG 205
SbTIP3;2     MTFGLMYAVYATAVDH-----------RGRSGATTTI-AIAPLAIGFVLGANILAGGPFDG 202
BdTIP3;2     MTFGLMYTVYATAVDR-----------NDGVG------AIAPVAIGFVLGANILTGGPFDG 198
OsTIP3;2     MTFGLVYTVYATAVDR-----------RSGGG------DIAPLAIGLVAGANILAGGPFDG 201
AtTIP3;1     LTFGLVYVVVYSTLIDP----------KRGSLGIIA------PLAIGLIVGANILVGGPFSG 203
AtTIP3;2     LTFALVYVVVYSTAIDP----------KRGSIGIIA------PLAIGLIVGANILVGGPFDG 203
PtTIP3;1     LTFGLVYTVYATAIDP----------KRGSLGIIA------PLAIGFVVGANILVGGPFDG 194
PtTIP3;2     LTFGVVYTVYATALDP----------KRGSLGIIA------PLAIGFIVGANILVGGPFDG 194
SmTIP6;1     TTFALIFTVYGTAVDH----------KRGVVGTIA------PIAIGFIVLANILAAGPFSG 192
SmTIP7;1     LTFVLVYVIFGTAVDK------------KGPSTIA------PLTIGMAVLVDHLVGVPVTG 191
               :*  .:  .                                .:   *     .   .        *

              LE                   TM6
PpTIP6;1     ASMNPGRSFGPAVVA-WDFKNHWVYWVGPLVGAALAALIYDGVFISPAPPA-GHQPVP-- 247
PpTIP6;2     ASMNPGRSFGPAVVA-WDFTNHWVYWVGPFIGAALAALIYDGVFMSPAAPE-GHQPVP-- 247
PpTIP6;3     ASMNPGRSFGPAVIA-MDFTNHWVYWVGPFIGAALAAVIYDGVFISPSPPA-GHQAIP-- 248
PpTIP6;4     ASMNPGRSFGPALVA-MDFTNHWVYWVGPFIGAALAALIYDGVFISPSPPP-GHHAIP-- 248
PvTIP5;1     ASMNPARSFGPAVVS-GDYKNQAVYWAGPMIGAAVAALAHQVLAC--PPDAAA-----GS 259
PvTIP5;2     ASMNPARSFGPAVVS-GDYKNQAVYWAGPMIGAAIAALAHQVLACG-PPDAAAS--ATGS 255
SiTIP5;2     ASMNPARSFGPAVVS-GNYKNQAVYWAGPMIGAAVAALAHQILAG--APDAAAA--GSSS 248
BdTIP5;1     ASMNPARSFGPAVVS-GDFKNQAVYWVGPMIGAAVAALVHQNLVFPSAPEPLP-----HE 252
OsTIP5;1     ASMNPARSFGPAVVS-GHYSNQAVYWAGPMVGAAVAALVHQALVFPTVPEPAPAPATNES 258
ZmTIP5;1     ASMNPARSFGPAVVS-GHFKNQAVYWAGPMVGAAVAALVYQIMACP-------------- 249
SiTIP5;1     ASMNPARSFGPAVVS-GDFRNQAVYWAGPMVGAALAAIVHQHVMYP------------AS 248
PtTIP5;1     GSMNPACAFGSAVIA-GRLKNQAVYWVGPLIGAAVAGLLYDNVVFP-----------TE- 241
PtTIP5;2     GSMNPACAFGSAVIA-GKFKNQAVYWVGPLIGASVAGLLYDNVVFP-----------TQ- 241
AtTIP5;1     GSMNPACAFGSAMVY-GSFKNQAVYWVGPLLGGATAALVYDNVVVP-----------VED 244
PtTIP1;1     ASMNPAVSFGPAVVS-WTWDSHWVYWLGPFVGSAIAAIVYEVIFINP--ST--HEQLP-- 248
PtTIP1;2     ASMNPAVSFGPAVVS-WTWDNHWVYWLGPFVGSAIAAIVYEVCFISP--TT--HEQLT-- 248
GhTIP1;2     ASMNPAVSFGPAVVS-WTWDNHWVYWLGPFIGSAIAAIVYEVFFIAP--ST--YEEVP-- 248
GhTIP1;7     ASMNPAVSFGPAVVS-WTWDNHWVYWLGPFIGSAIAAIVYEVFFIAP--ST--YEELP-- 248
PvTIP1;3     AAMNPAVAFGPAVVT-GVWENHWVYWLGSFVGAAIAALVYDIIFIGQ--RP--HDQLP-- 248
PvTIP1;4     AAMNPAVAFGPAVVT-GVWENHWVYWLGSFVGAAIAALVYDIIFIGQ--RP--HDQLP-- 246
SiTIP1;1     ASMNPAVSFGPAVVS-GVWENHWVYWLGPFVGAAIAALVYDIIFIGQ--RP--HDHLP-- 264
OsTIP1;2     ASMNPAVSFGPAVVT-GVWDNHWVYWLGPFVGAAIAALIYDIIFIGQ--RP--HDQLP-- 248
ZmTIP1;2     ASMNPAVSFGPAVVT-GVWENHWVYWVGPLAGAAIAALVYDIIFIGQ--RP--HQQLPT- 249
SbTIP1;2     ASMNPAVSFGPAVVS-GVWENHWVYWLGPFAGAAIAALVYDIIFIGQQQRPTHHHHLP-- 254
```

TM6

```
BdTIP1;2     ASMNPAVSFGPAVVS-GVWENHWVYWLGPFAGAAIAALVYDIVFIGQ--RP--HDQLP-- 248
PtTIP1;3     ASMNPAVSFGPAVVS-WTWTNHWVYWLGPFIGAAIAALVYDNIFIGS--GG--HEPLP-- 248
PtTIP1;4     ASMNPAVSFGPAVVS-WTWTNHWVYWLGPFIGAGIAALVYDNIFIGS--GG--HEPLP-- 248
AtTIP1;3     ASMNPAVSFGPAVVS-WIWTNHWVYWVGPFIGAAIAAIVYDTIFIGS--NG--HEPLP-- 248
AtTIP1;1     ASMNPAVAFGPAVVS-WTWTNHWVYWAGPLVGGGIAGLIYEVFFINT-----THEQLPT- 248
AtTIP1;2     ASMNPAVAFGPAVVS-WTWTNHWVYWAGPLIGGGLAGIIYDFVFIDEN----AHEQLPT- 250
GhTIP1;1     ASMNPAVSFGPALVS-WTWDNHWIYWVGPLIGGGLAGLIYEFIFISN-----THEQLPT- 248
GhTIP1;3     ASMNPAVSFGPALVS-WTWDNHWIYWVGPLIGGGLAGLIYEFIFISN-----THEQLPT- 248
GhTIP1;8     ASMNPAVSFGPALVS-WTWDNHWIYWVGPLIGGGLAGLIYEFIFISN-----THEQLPT- 248
GhTIP1;4     ASMNPAVSFGPALVS-WSWDNHWIYWLGPLIGGGLAGLIYEFAFINQ-----SHEQLPP- 248
GhTIP1;6     ASMNPAVSFGPALVS-WSWDNHWIYWLGPLIGGGLAGLIYEFPFINQ-----SHEQLPP- 248
GhTIP1;5     ASMNPAVSFGPALVS-WSWENHWVYWAGPLIGGGLAGLVYEFIFINQ-----THEQLP-- 247
PtTIP1;5     ASMNPAVSFGPALVS-WSWTNHWVYWAGPLVGGGLAGLIYELFFIGFG----THEQLP-- 248
PtTIP1;6     ASMNPAVSFGPALVS-WTWTNHWVYWAGPLIGGGLAGLIYEFFFIGFG----NHEQLP-- 248
PvTIP1;1     ASMNPAVSFGPALVS-WSWTHQWVYWVGPLIGGGLAGVIYEVLFISHT-----HEQLP-- 246
PvTIP1;2     ASMNPAVSFGPALVS-WSWTHQWVYWVGPLIGGGLAGVIYEVLFISHT-----HEQLP-- 246
SiTIP1;2     ASMNPAVSFGPALVS-WSWGYQWVYWVGPLIGGGLAGVIYEVLFISHT-----HEQLP-- 245
OsTIP1;1     ASMNPAVSFGPALVS-WSWESQWVYWVGPLIGGGLAGVIYEVLFISHT-----HEQLP-- 246
SbTIP1;1     ASMNPAVSFGPALVS-WEWGYQWVYWVGPLIGGGLAGVIYELLFISQT-----HEQLP-- 246
ZmTIP1;1     ASMNPAVSFGPALVS-WEWGYQWVYWVGPLIGGGLAGVIYELLFISHT-----HEQLP-- 246
BdTIP1;1     ASMNPAVSFGPALVS-WEWGYQWVYWVGPLIGGGLAGVIYEVLFISHT-----HEQLP-- 246
PtTIP1;7     AALNPAVPFGPALVS-WNWHHHWVYWAGPLIGGGLAGVVYELIFISH-----THEPLA-- 251
PtTIP1;8     AALNPAVPFGPALVS-WNWYHHWVYWAGPLIGGGLAGIVYELIFMSHS----THEPLP-- 252
PvTIP3;1     AGMNPARVFGPALVG-WRWRHHWVYWLGPFLGAGVAGLVYEYLVIPSADAAPHAHG---- 254
PvTIP3;2     AGMNPARVFGPALVG-WRWRHHWVYWLGPFLGAGLAGLVYEYLVIPSADAAPLAHGH--- 255
SiTIP3;2     AGMNPARVFGPALVG-WRWRHHWVYWLGPFLGAGVAGLVYEYLVIPSADAA--APHA--- 253
ZmTIP3;1     AGMNPARVFGPALVG-WRWRHHWVYWLGPFLGAGLAGLVYEYLVIPSADAA--VPHA--- 253
SbTIP3;1     AGMNPARVFGPALVG-WRWRHHWVYWLGPFLGAGLAGLVYEYLVIPSADAAPPHSTH--- 257
ZmTIP3;2     AGMNPARVFGPALVG-WRWRHHWVYWLGPFLGAGLAGLVYEYLLIPPADAVP---HT--- 257
OsTIP3;1     AGMNPARVFGPALVG-WRWRHHWVYWLGPFVGAGLAGLLYEYLVIPSADAAP-HGGA--- 255
BdTIP3;1     AGMNPARVFGPALVG-WRWGHHWVYWVGPFLGAGIAGLLYEYLVIPASETAA--AHT--- 256
SiTIP3;1     AVMNPARAFGPAIVGSRRWSNHWVYWAGPMVGAGLSGFFYEHLVVTPADEEEPAAAP--- 249
SbTIP3;3     AVMNPARAFGPAVVGSRRWGNHWVYWVGPMVGAGLSGVLYEHLVAG---GEEAEPAP--- 263
PvTIP3;3     AAMNPARAFGPALVG-WSWRHHWVYWVGPLIGAGLAGALYESVMVEQPSEAPPAAAAGPR 264
SbTIP3;2     AAMNPARAFGPALVG-WSWRHHWVYWVGPLIGAGLAGALYESVMVEQEPEAAPAAAP-PR 260
BdTIP3;2     AAMNPARAFGPALVG-WNWSHHWVYWVGPMIGAGLAGALYEFVVGEQPDQAPPAAAR--- 254
OsTIP3;2     AAMNPARAFGPALVG-WNWRHHWVYWLGPLIGAGMAGALYEFVMAEQPEPPAAADTR--- 257
AtTIP3;1     ASMNPARAFGPALVG-WRWHDHWIYWVGPFIGSALAALIYEYMVIP-TEPPTHHAHG--V 259
AtTIP3;2     ASMNPARAFGPALVG-WRWSNHWIYWVGPFIGGALAALIYEYMIIPSVNEPPHHST---- 258
PtTIP3;1     ASMNPARAFGPALVG-WRWRNHWIYWVGPFLGGGLAALIYEYIVIS-AEPVAHHTH---Q 249
PtTIP3;2     ASMNPARAFGPALIG-WRWRNHWIYWVGPFLGGGLAALIYEYIVIP-TEPVPRHAH---Q 249
SmTIP6;1     GSMNPARSFGPALVT-FDWTNHWIYWVGPLIGGGLAGLVYNEILITPPP----PEEY--- 244
SmTIP7;1     ASMNPARSLGAALWS-GQWKNHWIYWAAPLLGATAAALIYTALFLPTVSSTQKQSTNDLI 250
             . :**.  :*.*:        :
```

```
PpTIP6;1        ------TEF--------------------------------- 250
PpTIP6;2        ------TEF--------------------------------- 250
PpTIP6;3        ------SDF--------------------------------- 251
PpTIP6;4        ------SDF--------------------------------- 251
PvTIP5;1        SRHGNVETVVV------------------------------- 270
PvTIP5;2        SRHGSVETVVV------------------------------- 266
SiTIP5;2        SCHGNVETVVV------------------------------- 259
BdTIP5;1        VRHGSVETVVA------------------------------- 263
OsTIP5;1        ARHGSVQTVVV------------------------------- 269
ZmTIP5;1        SVTGNVEAVVV------------------------------- 260
SiTIP5;1        LRPGSVETVVV------------------------------- 259
PtTIP5;1        ------------------------------------------
PtTIP5;2        ------------------------------------------
AtTIP5;1        DRGSSTGDAIGV------------------------------ 256
PtTIP1;1        -----STDF--------------------------------- 252
PtTIP1;2        -----SSDF--------------------------------- 252
GhTIP1;2        -----SADF--------------------------------- 252
GhTIP1;7        -----SADF--------------------------------- 252
PvTIP1;3        -----ATDY--------------------------------- 252
PvTIP1;4        -----ATDY--------------------------------- 250
SiTIP1;1        -----TTDY--------------------------------- 268
OsTIP1-2        -----TADY--------------------------------- 252
ZmTIP1;2        ----TAADY--------------------------------- 254
SbTIP1;2        -----TTDY--------------------------------- 258
BdTIP1;2        -----SAEY--------------------------------- 252
PtTIP1;3        -----TNDF--------------------------------- 252
PtTIP1;4        -----TNDF--------------------------------- 252
AtTIP1;3        -----SNDF--------------------------------- 252
AtTIP1;1        ------TDY--------------------------------- 251
AtTIP1;2        ------TDY--------------------------------- 253
GhTIP1;1        ------TDY--------------------------------- 251
GhTIP1;3        ------TDY--------------------------------- 251
GhTIP1;8        ------TDY--------------------------------- 251
GhTIP1;4        ------TDY--------------------------------- 251
GhTIP1;6        ------TDYWKQFNFIMKNGFFPFPFSGGKVFLVEAFSFLGV 284
GhTIP1;5        -------NY--------------------------------- 249
PtTIP1;5        -----TTDY--------------------------------- 252
PtTIP1;6        -----TADY--------------------------------- 252
PvTIP1;1        -----TTDY--------------------------------- 250
```

```
PvTIP1;2        -----TTDY--------------------------------- 250
SiTIP1;2        -----TTDY--------------------------------- 249
OsTIP1;1        -----TTDY--------------------------------- 250
SbTIP1;1        -----TTDY--------------------------------- 250
ZmTIP1;1        -----STDY--------------------------------- 250
BdTIP1;1        -----TTDY--------------------------------- 250
PtTIP1;7        -----VVEY--------------------------------- 255
PtTIP1;8        -----GGEF--------------------------------- 256
PvTIP3;1        HQPLAPEDY--------------------------------- 263
PvTIP3;2        HQPLAPEDY--------------------------------- 264
SiTIP3;2        HQPLAPEDY--------------------------------- 262
ZmTIP3;1        HQPLAPEDY--------------------------------- 262
SbTIP3;1        HQPLAPEDY--------------------------------- 266
ZmTIP3;2        HQPLAPEDY--------------------------------- 266
OsTIP3;1        HQPLAPEDY--------------------------------- 264
BdTIP3;1        HQPLAPEDY--------------------------------- 265
SiTIP3;1        SRGSSRRA---------------------------------- 257
SbTIP3;3        SCGGRRRE---------------------------------- 271
PvTIP3;3        MLAAAAEDY--------------------------------- 273
SbTIP3;2        MP-LASEDY--------------------------------- 268
BdTIP3;2        -LPEPVEDY--------------------------------- 262
OsTIP3;2        -LPVAAEDY--------------------------------- 265
AtTIP3;1        HQPLAPEDY--------------------------------- 268
AtTIP3;2        HQPLAPEDY--------------------------------- 267
PtTIP3;1        HQPLAPEDY--------------------------------- 258
PtTIP3;2        HQPLAPEDY--------------------------------- 258
SmTIP6;1        ------------------------------------------
SmTIP7;1        KNIKNEKHTEAVV----------------------------- 263
```
