# Supplementary material for: Genome-Wide Characterization of Major Intrinsic Proteins in Four Grass Plants and Their Non-Aqua Transport Selectivity Profiles with Comparative Perspective
Source: PLoS One. 2016 Jun 21;11(6):e0157735. doi: 10.1371/journal.pone.0157735 (PMC4915720; doi:10.1371/journal.pone.0157735)
Supplement: S10 Fig — Multiple sequence alignment of silicon transporter (A) and silicon non-transporter (B) NIPs of the twelve plants. The conserved pore lining F/L-x-H-F-P motif in loop B is shown in the blue boxes. The description of the figure legend is as for S9 Fig. (PDF) [file pone.0157735.s010.pdf]

Figure S10 (A)

```
ZmNIP2;1        ---------------------------------------------MST-----NSRSNSR 10
SbNIP2;1        ---------------------------------------------MST-----NSRSNSR 10
PvNIP2;1        ---------------------------------------------MST-----NSRSNSR 10
SiNIP2;3        MTATMPLDQCKQSKRAVSFFFLLLLLLFPVGTHVCSRTHVARSPEMST-----NSRSNSR 55
BdNIP2;1        ---------------------------------------------MST-----NSRSNSR 10
OsNIP2;1        --------------------------------------------MASN-----NSRTNSR 11
PvNIP2;3        ------------------------------------------MAAAST-----ASRANSR 13
PvNIP2;4        ------------------------------------------MAAAST-----PSRANSR 13
SiNIP2;2        ------------------------------------------MAASTA-----PSRTNSR 13
ZmNIP2;2        ------------------------------------------MAAAST-----TSRTNSR 13
ZmNIP2;3        -------------------------------------------MAAST-----TSRTNSR 12
OsNIP2;2        ------------------------------------------MASTTA-----PSRTNSR 13
BdNIP2;2        ------------------------------------------MAASGTG---TPTRANSR 15
PtNIP3;2        ----------------------------------MDNEEVPSAPSTPAT-----PGTPGA 21
GhNIP6;1        ----------------------------------MENEDVPSAPSTPVT-----PGTPGA 21
GmNIP6;2        ----------------------------------MDNNEE--IPSTPAT-----PGTPGA 19
OsNIP3;1        -----------------------------------------MQLVFFP--------YLML 11
PvNIP3;10       -----------------------------------------MPVLISSS---RLQSFAVL 16
PvNIP3;9        ----------------------------------MEPAAGSTPPNGSAP---ATPGTPAP 23
SiNIP3;5        ----------------------------------MEP--GSTPPNGSAP---ATPGTPAP 21
ZmNIP3;1        ----------------------------------MEP--GSTPPNGSAP---ATPGTPAP 21
BdNIP3;1        ----------------------------------MEG---AATPNMSAP---ATPGTPAP 20
PtNIP3;3        ----------------------------------MP-ESEAGTPAVSAP---NTPGTPGG 22
PtNIP3;4        ----------------------------------MPGPEEAGTPTVTAP---NTPGTPGG 23
AtNIP5;1        ---------------------------------MAPPEAEVGAVMVMAPPTPGTPGTPGG 27
OsNIP3;2        --------------------------MEGGKMSSMGMDAASASVTVPPMQMQAGDQSNRI 34
```

```
                                                               ↓
ZmNIP2;1        ANFNNEIHDIGTAQNSS-------MPPTYY-D-----------RSLADIFP-------PH 44
SbNIP2;1        ANFNNEIHDIGTVQNSTM------MPPTYY-D-----------RSLADIFP-------PH 45
PvNIP2;1        ANFNNEIHDIATPQNST-------MPPMYYSD-----------RSLADFFP-------PH 45
SiNIP2;3        ANFNNEIHDISTVQNST-------MPPMYYSD-----------RSLADFFP-------PH 90
BdNIP2;1        ANFSNEIHDMATPQNSNM------PNMMYYNE-----------RSLADFFP-------PH 46
OsNIP2;1        ANYSNEIHDLSTVQNGT-------MPTMYYGE-----------KAIADFFP-------PH 46
PvNIP2;3        VNYSNEIHDLSTVQSG------SAVPTMYYPE-----------KSLADIFP-------PH 49
PvNIP2;4        VNYSNEIHDLSTVQSGG-----SAVPTMYYPE-----------KSLADIFP-------PH 50
SiNIP2;2        VNYSNEIHDLSTVQSGG-----SAVPTMYYPE-----------KSLADIFP-------PH 50
ZmNIP2;2        VNYSNEIHDLSTVQSG------SVVPTLFYPD-----------KSIADIFP-------PH 49
ZmNIP2;3        VNYSNEIHDLSTVQGGSAA---AAAAALFYPDS----------KSIADIFP-------PH 52
OsNIP2;2        VNYSNEIHDLSTVQSVS------AVPSVYYPE-----------KSFADIFP-------PN 49
BdNIP2;2        VNYSNEIHDLSTVQDGAP----SLAPSMYYQE-----------KSFADFFP-------PH 53
PtNIP3;2        PLFGGFKGE-RGVHGKKS-----LLRSCKCFGVEEWAMEEGRLPPVSCSLP----PPPVS 71
GhNIP6;1        PLFGGFKGDHRGGFNKKS-----LLKSCKCFSVED-SMEEGRLPPVSCSLP----PPPVS 71
GmNIP6;2        PLFGGFSNG-RNNNSKKS-----LLKSCRCFSVEEWSLEDGGLPAVSCSLPLPSPPPVVP 73
OsNIP3;1        HVC--------------------------------------------------------- 14
PvNIP3;10       HCCCLQTKFPFFSKKRKW-------KQQCGA--PVADSNGGAHVCV-------------- 53
PvNIP3;9        LFS-GGPRVDSLSYERKS------MPRCRCLP--AVEGWGLATHTCVVEIP----APDVS 70
SiNIP3;5        LFS-GGPRVDSLSYERKS------MPRCRCLP--AVEGWGLATHTCVVEIP----APDVS 68
ZmNIP3;1        LFSSGGPRVDSLSYERKS------MPRCKCLPLPAVEGWGVATHTCVVEIP----APDVS 71
BdNIP3;1        LFP--GARVDSMSYERKSS---MSVPRCRCLP---VEAWMSSQHACVLEIP----APDVS 68
PtNIP3;3        PLF-TGLRVDSLSYSDRK-----IMPKCKCLP--VTAPTWGQPHTCFLDFP----APDVS 70
PtNIP3;4        PLF-TGLRVDSLSYSDRK-----IMPKCKCLP--VTAPNWGQPHTCFLDIP----SPDVS 71
AtNIP5;1        PLI-TGMRVDSMSFDHRK-----PTPRCKCLP--VMGSTWGQHDTCFTDFP----SPDVS 75
OsNIP3;2        AIIISPRAGSSKILPFELVNGAANAGSQRHADPAESTPEAHHHLWHPVDLP--KIKPPVP 92
```

```
                TM1                                 TM2
                ↓  ↓          ↓↓  ↓↓     ↓        ↓  ↓↓  ↓↓  ↓  ↓↓    ↓ ↓↓↓↓
ZmNIP2;1        LLKKVVSEVVSTFLLVFVTCGAAGIYGSDKDRISQLGQSVAGGLIVTVMIYAVGHISGAH 104
SbNIP2;1        LLKKVVSEVVSTFLLVFVTCGAAGIYGSDKDRISQLGQSVAGGLIVTVMIYAVGHISGAH 105
PvNIP2;1        LLKKVVSEVVSTFLLVFVTCGASAISGSDLHRISQLGQSVAGGLIVTVMIYAVGHISGAH 105
SiNIP2;3        LLKKVVSEVVSTFLLVFVTCGAAAISASDLNRISQLGQSVAGGLIVTVMIYAVGHISGAH 150
BdNIP2;1        LLKKMVSEVVSTFLLVFVTCGASAINGNDPSRISQLGQSVAGGLIVTVMIYSVGHISGAH 106
OsNIP2;1        LLKKVVSEVVATFLLVFMTCGAAGISGSDLSRISQLGQSIAGGLIVTVMIYAVGHISGAH 106
PvNIP2;3        LLKKVISEVVATFLLVFVTCGAASIYGEDLKRISQLGQSVAGGLIVTVMIYATGHISGAH 109
PvNIP2;4        LLKKVISEVVATFLLVFVTCGAASIYGEDLKRISQLGQSVAGGLIVTVMIYATGHISGAH 110
SiNIP2;2        LGKKVISEVVATFLLVFVTCGAASIYGEDLKRISQLGQSVAGGLIVTVMIYATGHISGAH 110
ZmNIP2;2        LGKKVISEVVATFLLVFVTCGAASIYGEDNRRISQLGQSVAGGLIVTVMIYATGHISGAH 109
ZmNIP2;3        LGKKVISEVVATFLLVFVTCGAASIYGEDNARISQLGQSVAGGLIVTVMIYATGHISGAH 112
OsNIP2;2        LLKKVISEVVATFLLVFVTCGAASIYGEDMKRISQLGQSVVGGLIVTVMIYATGHISGAH 109
BdNIP2;2        LLKKVISEVVATFLLVFVTCGAASIYGADVTRVSQLGQSLVGGLIVTVMIYATGHISGAH 113
PtNIP3;2        LARKLGAEFMGTLILIFAGTATAIVNQKTQGSETLIGLAASTGLAAMIVILSTGHISGAH 131
GhNIP6;1        LTRKVGAEFIGTFILIFAGTATAIVNQKTQGSETLIGLAASTGLAVMIVILSTGHISGAH 131
GmNIP6;2        LARKIGAEFIGTFILMFAGTAAAIVNQKTNGSETLIGCAATTGLAVMIVILATGHISGAH 133
OsNIP3-1        ----LGAEFVGTFILIFFATAAPIVNQKYGGAISPFGNAACAGLAVTTIILSTGHISGAH 70
PvNIP3;10       ---QLGAEFVGTFILIFFATAAPIVNQKYGGAISPFGNAACAGLAVTIIILSTGHISGAH 110
PvNIP3;9        LTRKLGAEFVGTFILIFFATAAPIVNQKYGGAISPFGNAACAGLAVTIIILSTGHISGAH 130
SiNIP3;5        LTRKLGAEFMGTFILIFFATAAPIVNQKYGGVISPFGNAACAGLAVTIIILSTGHISGAH 128
ZmNIP3;1        LTRKLGAEFVGTFILIFFATAAPIVNQKYGGAISPFGNAACAGLAVATVILSTGHISGAH 131
BdNIP3;1        LTRKLGAEFVGTFILIFFATAAPIVNQKYNNAISPFGNAACAGLAVTTIILSTGHISGAH 128
PtNIP3;3        LTRKLGAEFVGTFILIFAATAGPIVNQKYNNAETLIGNAACAGLAVMIIILSTGHISGAH 130
PtNIP3;4        LTRKLGAEFVGTFILIFMATAGPIVNQKYDHAETLIGNAACAGLAVMIIILSTGHISGAH 131
AtNIP5;1        LTRKLGAEFVGTFILIFTATAGPIVNQKYDGAETLIGNAACAGLAVMIIILSTGHISGAH 135
OsNIP3;2        LVKKVGAEFFGTFTLIFTVLSTIIMDEQHKGVESLLGIATSAGLAVTVLVLSLIHISGCH 152
                    : :*...*: *:*   .   :        : :* :   ** .  :: :  ****.*
```

```
                LB                        TM3
ZmNIP2;1    MNPAVTLAFAVFRHFPWIQVPFYWAAQFTGSICASFVLKAVLHPIAVLGTTTP--TGPHW 162
SbNIP2;1    MNPAVTLAFAVFRHFPWIQVPFYWAAQFTGAICASFVLKAVLHPISVLGCTTP--TGPHW 163
PvNIP2;1    MNPAVTLAFAVFRHFPWIQVPFYWAAQFTGAICASFVLKAVLHPITVLGTTTP--TGPHW 163
SiNIP2;3    MNPAVTLAFAVFRHFPWIQVPFYWAAQFTGAICASFVLKAVLHPITVIGTTTP--TGPHW 208
BdNIP2;1    MNPAVTTAFAVFRHFPWIQVPFYWASQFTGAICASFVLKAVLHPIEVLGTTTP--VGPHW 164
OsNIP2;1    MNPAVTLAFAVFRHFPWIQVPFYWAAQFTGAICASFVLKAVIHPVDVIGTTTP--VGPHW 164
PvNIP2;3    MNPAVTLSFACFRHFPWIQVPFYWAAQFTGAMCAAFVLKAVLHPITVIGTTTP--TGPHW 167
PvNIP2;4    MNPAVTLSFACFRHFPWIQVPFYWAAQFTGAMCAAFVLKAVLHPIAVIGTTTP--TGPHW 168
SiNIP2;2    MNPAVTLSFACFRHFPWIQVPFYWAAQFTGAMCAAFVLKAVLHPIEVIGTTTP--TGPHW 168
ZmNIP2;2    MNPAVTLSFACFRHFPWIQVPFYWAAQFTGAMCAAFVLKAVLHPIAVIGTTTP--SGPHW 167
ZmNIP2;3    MNPAVTLSFACFRHFPWIQVPFYWAAQFTGAMCAAFVLKAVLQPIAVIGTTTP--SGPHW 170
OsNIP2;2    MNPAVTLSFAFFRHFPWIQVPFYWAAQFTGAMCAAFVLRAVLYPIEVLGTTTP--TGPHW 167
BdNIP2;2    MNPAVTLSFACFRHFPWIQVPFYWAAQFTGAMCAAFVLRAVLHPITVLGTTTP--TGPHW 171
PtNIP3;2    LNPSITIAFAALKHFPWKHVPVYIGAQVLASLCAAFALKVIFHPMMGGGVTVP--SGGHG 189
GhNIP6;1    LNPAVTIAFAALKHFPRKHVPVYIGAQVMASLCAAFGLKGVFHPMMGGGVTVP--SGGFG 189
GmNIP6;2    LNPAVTISFAALKHFPWKHVPMYIGAQVLASICAGFALKGVYHPFMSGGVTVP--SGGYG 191
OsNIP3;1    LNPSLTIAFAALRHFPWLQVPAYVAVQVLGSICAGFALKGVFHPFLSGGVTVPDPTISTA 130
PvNIP3;10   LNPSLTIAFAALRHFPWLQVPAYVSVQVLGSICASFALKGVFHPFLSGGVTVPDVTISTA 170
PvNIP3;9    LNPSLTIAFAALRHFPWLQVPAYVSVQVLGSICASFALKGVFHPFLSGGVTVPDVTISTA 190
SiNIP3;5    LNPSLTIAFAALRHFPWLQVPAYVSVQVLGSICASFALKGVFHPFLSGGVTVPDVTISTA 188
ZmNIP3;1    LNPSLTIAFAALRHFPWLQVPAYVAVQALASVCAAFALKGVFHPFLSGGVTVPDATVSTA 191
BdNIP3;1    LNPSLTIAFAALRHFPWLQVPAYVAVQSLASVCAGFALKGVFHPFLSGGVTVPDAAVSTA 188
PtNIP3;3    LNPSLTIAFAALRHFPWVQVPAYIAAQVSASICASFALKGVFHPFMSGGVTVP--SVSTG 188
PtNIP3;4    LNPSLTIAFAALRHFPWVQVPAYIAAQVSASICASFALKGVFHPFMSGGVTVP--SVSTG 189
AtNIP5;1    LNPSLTIAFAALRHFPWAHVPAYIAAQVSASICASFALKGVFHPFMSGGVTIP--SVSLG 193
            :**::: ::: : ***  ::  * . *  .:: *.* :: :  *.     * *
```

```
                  TM4                         TM5
ZmNIP2;1    HSLVIEIIVTFNMMFVTLAVATDTRAVGELAGLAVGSAVCITSIFAGAVSGGSMNPARTL 222
SbNIP2;1    HSLIIEIIVTFNMMFVTLAVATDTRAVGELAGLAVGSAVCITSIFAGAVSGGSMNPARTL 223
PvNIP2;1    HALVIEVIVTFNMMFVTLAVATDTRAVGELAGLAVGSAVCITSIFAGAVSGGSMNPARTL 223
SiNIP2;3    HALVIEVIVTFNMMFVTLAVATDTRAVGELAGLAVGSAVCITSIFAGAVSGGSMNPARTL 268
BdNIP2;1    HSLLIEIIVTFNMMFVTLAVATDTRAVGELAGLAVGSSVCITSIFAGAVSGGSMNPARTL 224
OsNIP2;1    HSLVVEVIVTFNMMFVTLAVATDTRAVGELAGLAVGSAVCITSIFAGAISGGSMNPARTL 224
PvNIP2;3    HALVIEIVVTFNMMFVTCAVATDSRAVGELAGLAVGSAVCITSIFAGPVSGGSMNPARTL 227
PvNIP2;4    HALVIEIVVTFNMMFVTCAVATDSRAVGELAGLAVGSAVCITSIFAGPVSGGSMNPARTL 228
SiNIP2;2    HALVIEIVVTFNMMFVTCAVATDSRAVGELAGLAVGSAVCITSIFAGPVSGGSMNPARTL 228
ZmNIP2;2    HALLIEIVVTFNMMFVTCAVATDSRAVGELAGLAVGSAVCITSIFAGPVSGGSMNPARTL 227
ZmNIP2;3    HALAIEIVVTFNMMFVTCAVATDSRAVGELAGLAVGSAVCITSIFAGPVSGGSMNPARTL 230
OsNIP2;2    HALVIEIVVTFNMMFVTCAVATDSRAVGELAGLAVGSAVCITSIFAGPVSGGSMNPARTL 227
BdNIP2;2    HALVIEIVVTFNMMFVTCAVATDSRAVGELAGLAVGAAVCITSIFAGPVSGGSMNPARTL 231
PtNIP3;2    QAFALEFIISFILMFVVTAVATDTRAVGELAGIAVGATVMLNILIAGETTGASMNPVRTL 249
GhNIP6;1    QAFALEFIISFNLMFVVTAVATDTRAVGELAGIAVGATVMLNILIAGPITGASMNPVRTL 249
GmNIP6;2    QSFALEFIIGFNLMFVVTAVATDTRAVGELAGIAVGATVMLNILIAGPVSGGSMNPVRTL 251
OsNIP3;1    QAFFTEFIITFNLLFVVTAVATDTRAVGELAGIAVGAAVTLNILIAGPTTGGSMNPVRTL 190
PvNIP3;10   QAFFTEFIITFNLLFVITAVATDTRAVGELAGIAVGAAVTLNILVAGPTTGGSMNPVRTL 230
PvNIP3;9    QAFFTEFIITFNLLFVVTAVATDTRAVGELAGIAVGAAVTLNILVAGTTTGGSMNPVRTL 250
SiNIP3;5    QAFFTEFIITFNLLFVVTAVATDTRAVGELAGIAVGAAVTLNILVAGPTTGGSMNPVRTL 248
ZmNIP3;1    QAFFTEFIISFNLLFVVTAVATDTRAVGELAGIAVGAAVTLNILVAGPTTGGSMNPVRTL 251
BdNIP3;1    QAFFTEFIITFNLLFVVTAVATDTRAVGELAGIAVGAAVTLNILIAGPTTGGSMNPVRTL 248
PtNIP3;3    QAFALEFLITFNLLFVVTAVATDTRAVGELAGIAVGATVMLNILVAGPSSGGSMNPVRSL 248
PtNIP3;4    QAFALEFFITFNLLFVVTAVATDTRAVGELAGIAVGATVMLNILVAGPSTGGSMNPVRTL 249
AtNIP5;1    QAFALEFIITFILLFVVTAVATDTRAVGELAGIAVGATVMLNILVAGPSTGGSMNPVRTL 253
OsNIP3;2    EAFFLEFVTTFVLLFIITALATDPNAVKELIAVAVGATIMMNALVAGPSTGASMNPARTL 270
            .::  *..  * ::*:  *:***..** ** .:***::: :. :.**  :*.****.*:*
```

```
                  ↓    ↓         TM2
ZmNIP2;1         GPALASNLYTGLWIYFLGPVLGTLSGAWTYTYIRFEEAPS-HK-DMSQKLSSFKLRRLQS 280
SbNIP2;1         GPALASNLYTGLWIYFLGPVLGTLSGAWTYTYIRFEEAPSTHK-DMSQKLSSFKLRRLQS 282
PvNIP2;1         GPALASNLYTGLWIYFLGPVLGTLSGAWTYTYIRFEDAPS-AK-DASQKLSSFKLRRLQS 281
SiNIP2;3         GPALASNLYTGLWIYFLGPVLGTLSGAWTYTYIRFEEAPS-NK-DAPQKLSSFKLRRLQS 326
BdNIP2;1         GPALASNRYTGLWLYFLGPILGTLSGAWTYTFIRFEDSPK----DAPQKLSSFKLRRLQS 280
OsNIP2;1         GPALASNKFDGLWIYFLGPVMGTLSGAWTYTFIRFEDTPK--E-GSSQKLSSFKLRRLRS 281
PvNIP2;3         APAVASNVYTGLWIYFLGPVIGTLSGAWVYTYIRFEEAPAKD---GPQRLSSFKLRRMQS 284
PvNIP2;4         APAVASNVYTGLWIYFLGPVIGTLSGAWVYTYIRFEEAPAKD---GPQRLSSFKLRRMQS 285
SiNIP2;2         APAVASNVYTGLWIYFLGPVIGTLSGAWVYTYIRFEEAPAKD---APQRLSSFKLRRMQS 285
ZmNIP2;2         APAVASNVFTGLWIYFLGPVIGTLSGAWVYTYIRFEEAPAAK---DTQRLSSFKLRRMQS 284
ZmNIP2;3         APAVASNVFTGLWIYFLGPVVGTLSGAWVYTYIRFEEAPAAAK-PDTQRLSSFKLRRMQS 289
OsNIP2;2         APAVASNVYTGLWIYFLGPVVGTLSGAWVYTYIRFEEAPAAAGGAAPQKLSSFKLRRLQS 287
BdNIP2;2         APAVASGVYSGLWIYFLGPVIGTLSGAWVYTYIRFEEAPSVKD--GPQKLSSFKLRRLQS 289
PtNIP3;2         GPAIAANNYKAIWVYLTAPILGALCGAGTYSAVKLPEEDGDTN-EKTSATRSFRR----- 303
GhNIP6;1         GPAIAANNYKAIWVYFTAPILGSPCGCRTYT----------------------------- 280
GmNIP6;2         GPAVAANNYKAIWVYLVAPILGALAGAGTYTAVKLPEEDDDAK-AKTSIS-SFRR----- 304
OsNIP3;1         GPAVAAGNYRQLWIYLIAPTLGAVAGAGVYTAVKLRDENG----ETPRPQRSFRR----- 241
PvNIP3;10        GPAVAAGNYRQLWIYLLAPTLGALAGAGVYTAVKLRDENG----ETPRAQRSFRR----- 281
PvNIP3;9         GPAVAAGNYRQLWIYLLAPTLGALAGAGVYTAVKLRDENG----ETPRTQRSFRR----- 301
SiNIP3;5         GPAVAAGNYRQLWIYLLAPTLGAVAGAGVYTAVKLRDENG----ETPRTQRSFRR----- 299
ZmNIP3;1         GPAVAAGNYRQLWIYLLAPTLGALAGASVYKAVKLRDENG----ETPRTQRSFRR----- 302
BdNIP3;1         GPAVAAGNYRQLWIYLVAPTLGAVAGAGVYTAVKLRDVDGGG--EAPRPQRSFRR----- 301
PtNIP3;3         GPAVAAGTYKDIWIYLVAPTLGALVGAATYTAVKLREEEA----DPPRQVRSFRR----- 299
PtNIP3;4         GPAIAAGNYKKIWIYLVAPTLGAVVGAGAYTLVKLRDDET----DPPRPVRSFRR----- 300
AtNIP5;1         GPAVASGNYRSLWVYLVAPTLGAISGAAVYTGVKLNDSVT----DPPRPVRSFRR----- 304
OsNIP3;2         GPAIATGRYTQIWVYLVATPLGAVAGEGFYFAIKL------------------------- 305
                 .**:*:. :  :*:*: .. :*:  *   *
```

```
ZmNIP2;1        Q--SVAVDDD-ELDHIQV 295
SbNIP2;1        Q--SVAAEDD-ELDHIQV 297
PvNIP2;1        Q--SVAADDD-ELDHIQV 296
SiNIP2;3        Q--SVAADDD-ELDHIQV 341
BdNIP2;1        Q--SVAAEDDDVLDHIPV 296
OsNIP2;1        QQ-SIAADDVDEMENIQV 298
PvNIP2;3        Q--SLAADEFDTV----- 295
PvNIP2;4        Q--SLAADEFDTV----- 296
SiNIP2;2        QS-ALAADEFDTV----- 297
ZmNIP2;2        Q---LAADEFDTV----- 294
ZmNIP2;3        QS-ALAADEFDTV----- 301
OsNIP2;2        Q--SMAADEFDNV----- 298
BdNIP2;2        QRSMANVDEFDHV----- 302
PtNIP3;2        ------------------
GhNIP6;1        ------------------
GmNIP6;2        ------------------
OsNIP3;1        ------------------
PvNIP3;10       ------------------
PvNIP3;9        ------------------
SiNIP3;5        ------------------
ZmNIP3;1        ------------------
BdNIP3;1        ------------------
PtNIP3;3        ------------------
PtNIP3;4        ------------------
AtNIP5;1        ------------------
OsNIP3;2        ------------------
```

Figure S10 B)

```
                                                                             ↓
ZmNIP1;1    ---MAGGGDHS--QTNGGHVDQRALEEGRKE------------------EFAD--QGCAA 35
SbNIP1;3    ---MAGGGDHNSAQTNGGGHDQRAMEEGRKEA-----------------EYADH-QGCAA 39
SiNIP1;1    ---MAGGGDNS----TNGARDQRAMEEGRKE------------------EFATD-QGCAA 34
PvNIP1;5    ---MAGGGDSS---QTNGARDQRAMEEGRKD------------------EFAGD-RGCGA 35
BdNIP1;1    ---MAGGGDNAQ-TNGAAARDQAAMEEGRKD--------------------DYD-QGCGL 35
OsNIP1-1    ---MAGGDNNSQTTNGGSGHEQRAMEEGRKQE-----------------EFAADGQGCGL 40
SbNIP1;4    ---MARRE-DDSYTNGSVFEVS--VEEGRKDKSAEAYAIDDLQPAAAAEAEVDDDDALCG 54
SiNIP1;3    ---MARRE-DDSYTNGSVFEVS--VEEGRKDK-SEAYADASKQPE---EANDGIDDAVCG 50
PvNIP1;6    ---MARRE-DDSYTNGSVYEAS--VEEGMKDK-SEAYAEADDVGGQPEEASDG--DALCG 51
PvNIP1;7    ---MARRE-DDSYTNGSVFEAS--VEEGVRDK-SEAYAEAD-VGGQPEEADGG--DALCG 50
OsNIP1-4    ---MARREVDDSYTNGSVVEVVS-IEEGSKMD-----KEDDHQNP---QAPDGGDVVVCG 48
BdNIP1;3    ---MARRE-DDSYTNGSVSMNDFSVEDGRKEK--EVYDHDE-------PEQDG----LCG 43
OsNIP1-2    ---MAGRE-DGA--------AAGAMEEGQ-DSKEVKCESSEDGSSS----SSSSRCHG-N 42
OsNIP1-5    ---MAGRE-DGA--------AAGAMEEGQ-DSKEVKCENSEDGSN------TSRRCQG-N 40
PvNIP1;3    ---MAGSEDGGG--------GTGAMEEGRDAGRQARYESSEDGGG------GSDRCSGGN 43
PvNIP1;4    ---MAGREDGGG--------GAGAMEEGRDAGRQPRYESSEDGGG------GSDRCSGGN 43
SbNIP1;1    ---MSGR--ASA--------AAGAMEEGQ-AGYQSSEDGSHGSGS------ASNRCN--D 38
PvNIP1;1    ---MAGAELANG----LHES-TVSMEEGRGG-DEAYRESSEQDGA-------V-----SC 39
SiNIP1;2    ---MAGAELANG----LHES-TVAMEEGRGGGDEACRESSEQDGA-------G-----SR 40
PvNIP1;2    ---MAGAELANG----LHES-TVAMEEGRRG----GGESSEQDVA-------V-----SR 36
SbNIP1;2    ---MAGAEVANGA---VHEGGALALEEGRGG-DEARCESSEQDGA-------GR----SR 42
OsNIP1-3    ---MAGGEHGVNG---QHEE-TRAMEEGSRD-HQARCENSEQDGG-------SKSSSNNH 45
BdNIP1;2    ---MPGGEHGGSN---GLQEHAGALEEGRGG-GGNEAEDPEKSPN-------SSG---KH 43
AtNIP4-1    ---MSSHSDEIE------EEQISRIEKGKGK-----------------DCQGGIETVICT 34
AtNIP4-2    ---MTSHGEEIE------DEQISRIEKGNCK-----------------DSQGGMETAICS 34
PtNIP1-4    ---MARKSDGIE------SQEITSMEEGLATP---------------TDPKENGKFDCCT 36
PtNIP1-3    ---MPWNNEFGD------DTEGGKKTESSDE-----------------DSPPE------- 27
GmNIP4;1    ---MEENGGNIH------ADST-----------------------------------FCG 16
GhNIP1;1    ---MAEISGCN----GNHEVVLNVNGETTHPPP----PPSS-----------APKRKDSD 38
GmNIP1;1    ---MDENSATN----GTHEVVLDVNRDVS------------------------RTTQASR 29
GmNIP1;2    ---MDENSATN----GTHEVILDVNKDVS------------------------RTTQPSR 29
GmNIP1;5    ---MADYSAGT----ESQEVVVNVTKNTS------------------------ETIQRSD 29
GmNIP1;3    MSVVADNSANN----GSHQVVLNVNGDAP------------------------KKCDDSA 32
GmNIP1;4    ------MYTNN----GSHQVVLNVNGDAS------------------------KKCDDSS 26
AtNIP1-1    ---MADISGNGYGNAREEVVMVNLKDEVEHQQEMEDIHNPR-----------PLKKQDSL 46
AtNIP1-2    ---MAEISGNG-GDARDGAVVVNLKEEDEQQQQQQAIHKP-------------LKKQDSL 43
AtNIP2-1    ---MDDISVSKSNHGNVVVLNIKASSLADTSLP------------------SNKHESSSP 39
PtNIP1-5    --MSSSNSITEPSPKFQLPTRRSIMAEAKAAS--------------------PAPEWLST 38
```

TM1

```
                       ↓  ↓                       ↓↓  ↓↓  ↓↓ ↓    ↓   ↓↓ ↓↓
ZmNIP1;1    MVVSVPFIQK-------------IIAEIFGTYFLMFAGCGAVTINA--SKNGQITFPGVA 80
SbNIP1;3    MVVSVPFIQK-------------IIAEIFGTYFLMFAGCGAVTINA--SKNGQITFPGVA 84
SiNIP1;1    L--SVPFIQK-------------IIAEIFGTYFLIFAGCGAVTINA--SRNGQITFPGVA 77
PvNIP1;5    A-ISIPFVQK-------------IIAEIFGTYFLIFAGCGAVTINA--SKNGAITFPGVA 79
BdNIP1;1    A-ISVPFVQK-------------IIAEIFGTYFLIFAGCGAVTINA--SRNGQITFPGVA 79
OsNIP1-1    A-FSVPFIQK-------------IIAEIFGTYFLIFAGCGAVTINQ--SKNGQITFPGVA 84
SbNIP1;4    MSASVAFIQQ-------------LIAEFLATFFLIFAGCGVITVN---DDNGMATFPGVA 98
SiNIP1;3    MPASISYIQQ-------------LIAEFLATFFLIFAGCGVITVN---DKNGMATFPGIA 94
PvNIP1;6    VQASVAFIQQ-------------LIAEFLATFFLIFAGCGVSTVN---EKNGMATFPGVA 95
PvNIP1;7    KRASVAFVQQ-------------LIAEFLATFFLIFAGCGVSTVN---EKNGQATFPGVA 94
OsNIP1-4    MPMSFTFLQM-------------LLAEFLATFFLMFAGLGAITVE---EKKGAVTFPGVA 92
BdNIP1;3    MPVSVPFLQM-------------LLAEFFSTYFLLFAGMGAIVVNN--EKDGALTFPGIT 88
OsNIP1-2    DVISVQFMQKVHPWCMCMNKNLLILAEILGTYFMIFAGCGAVVVNQ--STGGAVTFPGIC 100
OsNIP1-5    DMISVQFMQK-------------ILTEILGTYFMIFAGCGAVVVNL--STGGAVMFPGIC 85
PvNIP1;3    DMMSVQFMQK-------------IIAEILGTYFMIFAGCGSVVVNL--STAGTVTFPGIC 88
PvNIP1;4    DMMSVQFMQK-------------IIAEILGTYFMIFAGCGSVVVNL--STAGTVTFPGIC 88
SbNIP1;1    DMISVQFMQK-------------IIAEVLGTYFMIFAGCGSVVVNL--STNGTVTFPGIC 83
PvNIP1;1    PMFSVPFVQK-------------ILAEIFGTFFLIFAGCAAVAVNL--RTGGTVTFPGIC 84
SiNIP1;2    PMFSVPFVQK-------------IIAEIFGTYFLIFAGCAAVAVNL--RTGGTVTFPGIC 85
PvNIP1;2    PMFSVPFVQK-------------ILAEMFGTYFLIFAGCAAVAVNL--RTGGTVTFPGIC 81
SbNIP1;2    PMFSVPFVQK-------------IVAEVLGTYFLIFAGCAAVAVNL--RTGGTVTFPGIC 87
OsNIP1-3    PMFSVQFAQK-------------VIAEILGTFFLIFAGCAAVAVNK--RTGGTVTFPGIC 90
BdNIP1;2    PMLSVQFVQK-------------ILAEIFGTYFLIFAGCAAVAVNQ--RTAGTVTFPGIC 88
AtNIP4-1    SPSIVCLTQK-------------LIAEMIGTYFIVFSGCGVVVVN--VLYGGTITFPGIC 79
AtNIP4-2    SPSIVCLTQK-------------LIAEMIGTYFIIFSGCGVVVVN--VLYGGTITFPGIC 79
PtNIP1-4    SPAAVTITQK-------------LIAEVIGTYFVIFAGCGSVAVN--NIYG-SVTFPGVC 80
PtNIP1-3    --TTVQIIQK-------------IIAEMIGTFFLIFMGCGSVVVN--QMYG-SVTFPGVC 69
GmNIP4;1    SPAVVQVIQK-------------VIAELIGTYFLIFAGCCSVIINNAEETKGRITFPGIC 63
GhNIP1;1    LGFSVPFIQK-------------LMAEVLGTYFLIFAGCAAVVVNV--NNEKVVSLPGIS 83
GmNIP1;1    SCVNVSFLQK-------------LVAEVVGTYFLIFAGSASVVVNK--NNNNVVTLPGIS 74
GmNIP1;2    SCVNVSFLQK-------------LVAEVVGTYFLIFAGCASVVVNK--NNNNVVTHPGIS 74
GmNIP1;5    SLVSVPFLQK-------------LVAEAVGTYFLIFAGCASLVVNE--NYYNMITFPGIA 74
GmNIP1;3    NQDCVPLLQK-------------LVAEVVGTYFLIFAGCASVVVNL--DKDKVVTQPGIS 77
GmNIP1;4    NQDCVPLLQK-------------LVAEVVGTYFLIFAGCASVVVNL--DKDKVVTQPGIS 71
AtNIP1-1    LSVSVPFLQK-------------LIAEFLGTYFLVFTGCASVVVNM--QNDNVVTLPGIA 91
AtNIP1-2    LSISVPFLQK-------------LMAEVLGTYFLIFAGCAAVAVNT--QHDKAVTLPGIA 88
AtNIP2-1    PLLSVHFLQK-------------LLAELVGTYYLIFAGCAAIAVNA--QHNHVVTLVGIA 84
PtNIP1-5    RNAALSNFQK-------------IVAELMGTYILVFVGCGAALTDK----VQRLNMLGIA 81
                                   :::*  .*: ::* *      :            *:
```

```
                 TM2                LB
ZmNIP1;1   IVWGLAVMVMVYAVGHISGAHFNPAVTLAFATSGR-----------------FPWRQ---- 120
SbNIP1;3   IVWGLAVMVMVYAVGHISGAHFNPAVTFAFATSGR-----------------FPWRQ---- 124
SiNIP1;1   IVWGLAVMVMVYAVGHISGAHFNPAVTFAFATCGR-----------------FPWRQ---- 117
PvNIP1;5   IVWGLAVMVMVYAVGHISGAHFNPAVTFAFATCGR-----------------FPWRQ---- 119
BdNIP1;1   IVWGLAVMVMIYAVGHISGAHFNPAVTFAFATVGR-----------------FPWRQ---- 119
OsNIP1-1   IVWGLAVMVMVYAVGHISGAHFNPAVTLAFATCRR-----------------FPWRQ---- 124
SbNIP1;4   LVWGMTVMAMIYAVGHVSGAHINPAVTVGFAISGR-----------------FPWRK---- 138
SiNIP1;3   VVWGMTVMAMVYAVGHVSGAHINPAVTVGFAVSGR-----------------FPWRK---- 134
PvNIP1;6   VVWGMTVMAMIYAVGHVSGAHINPAVTVGFAISGR-----------------FPWKKASRF 139
PvNIP1;7   VVWGMTVMAMIYAVGHVSGAHINPAVTVGFAISGR-----------------FPWKK---- 134
OsNIP1-4   VAWGAAVMAMVYAVGHVSGAHLNPAVTLGFAVAGR-----------------FPWRR---- 132
BdNIP1;3   MVWGLAVMVMIYTVGHISGAHMNPAVSLGFAIAGR-----------------MPWKR---- 128
OsNIP1-2   AVWGLVVMVLVYTVSHISGAHFNPAVTVAFATCGR-----------------FRWKQ---- 140
OsNIP1-5   VVWGLVVTVLVYSVGHISGTHLNPAVTVAFATRGANDGSDYVSVKFTANNSFVVIN---- 141
PvNIP1;3   AVWGLVVMVLVYSVGHISGAHFNPAVTVAFATCGR-----------------FPWKQ---- 128
PvNIP1;4   AVWGLVVMVLVYSVGHISGAHFNPAVTVAFATCGR-----------------FPWKQ---- 128
SbNIP1;1   AVWGLVVMVLVYSVGHISGAHFNPAVTVAFATCGR-----------------FPWKQ---- 123
PvNIP1;1   IVWGLAVMVMVYSVGHISGAHLNPAVSVAFATCGR-----------------FPWWQ---- 124
SiNIP1;2   IVWGLAVMVMVYSVGHISGAHLNPAVSVAFATCGR-----------------FPWRQ---- 125
PvNIP1;2   IVWGLAVMVMVYSVGHISGAHLNPAVSVAFATCGR-----------------FPWWQ---- 121
SbNIP1;2   IVWGLAVMVMVYSVGHISGAHLNPAVSVAFATCGR-----------------FPWKQ---- 127
OsNIP1-3   ITWGLAVMVMVYSVGHISGAHLNPAVTLAFATCGR-----------------FPWRR---- 130
BdNIP1;2   ITWGLAVMVMVYSVGHISGAHFNPAVTFAFATCGR-----------------FPWKQ---- 128
AtNIP4-1   VTWGLIVMVMIYSTGHISGAHFNPAVTVTFAIFRR-----------------FPW------ 117
AtNIP4-2   VTWGLIVMVMIYSTGHISGAHFNPAVTVTFAVFRR-----------------FPW------ 117
PtNIP1-4   VTWGLIVMVMIYSLGHISGAHFNPAVTIAFAIFRR-----------------FPS------ 118
PtNIP1-3   VVWGLIVMVMVYSVGHISGAHFNPAVTVTFAIFRH-----------------FPY------ 107
GmNIP4;1   LVWGFSVTILVYSLAHVSGAHFNPAVTLSFAIYRH-----------------FPLRLAYIK 107
GhNIP1;1   MVWGLAVMVLVYSLGHISGAHFNPAVTIAFATCKR-----------------FPLKQ---- 123
GmNIP1;1   IVWGLVVMVLVYSVGHISGAHFNPAVTIAFASTKR-----------------FPLKQ---- 114
GmNIP1;2   IVWGLVVMVLVYSVGHISGAHFNPAVTIAFASTRR-----------------FPLKQ---- 114
GmNIP1;5   IVWGLVLTVLVYTVGHISGGHFNPAVTIAFASTRR-----------------FPLIQ---- 114
GmNIP1;3   IVWGLTVMVLVYSVGHISGAHFNPAVTIAHATTKR-----------------FPLKQ---- 117
GmNIP1;4   IVWGLTVMVLVYSVGHISGAHFNPAVTIAHATTKR-----------------FPLKQ---- 111
AtNIP1-1   IVWGLTIMVLIYSLGHISGAHINPAVTIAFASCGR-----------------FPLKQ---- 131
AtNIP1-2   IVWGLTVMVLVYSLGHISGAHFNPAVTIAFASCGR-----------------FPLKQ---- 128
AtNIP2-1   VVWGIVIMVLVYCLGHLS-AHFNPAVTLALASSQR-----------------FPLNQ---- 123
PtNIP1-5   IVWGAVLMAAIYALGHVSGAHFNPAVSIALAVVRK-----------------FSWKE---- 121
            .**  :   :*  .*:*  *:****:.   *                    :
```

```
                                                                  TM3
ZmNIP1;1        --------------------------------LPAYVLAQMLGATLASGTLRLMFGG---- 145
SbNIP1;3        --------------------------------LPAYVLAQMLGAVLASGTLRLMFGG---- 149
SiNIP1;1        --------------------------------LPAYVLAQMLGATLASGTLRLMFGG---- 142
PvNIP1;5        --------------------------------LPAYVLAQMLGSTLAAGTLRLMFGG---- 144
BdNIP1;1        --------------------------------VPAYVLAQMLGATLASGTLRLMFGG---- 144
OsNIP1-1        --------------------------------VPAYAAAQMLGATLAAGTLRLMFGG---- 149
SbNIP1;4        --------------------------------VPAYMVVQMVAATMASLVLRLMFGG---- 163
SiNIP1;3        --------------------------------VPAYMVVQTVAATFASLLLRQMFGR---- 159
PvNIP1;6        LLVICMVRVNIALITIIILMASFVYMIHARTQVPAYMVVQTVAATAASLMLRLMFGG---- 195
PvNIP1;7        --------------------------------VPAYMVVQTVAATAASLMLRLMFGG---- 159
OsNIP1-4        --------------------------------APAYALAQTAAATAASVVLRLMFGG---- 157
BdNIP1;3        --------------------------------VPAYMLVQVFAAIIVSVVLRLMFGG---- 153
OsNIP1-2        --------------------------------VPSYVVAQVLGSTMASLTLRVVFGGGGGG 169
OsNIP1-5        --------------------------------VPSYVVAQVLGSTMASLTLRVVFGGGGS- 169
PvNIP1;3        --------------------------------VPSYAVAQVLGSTLASLTLRVVFGG---- 153
PvNIP1;4        --------------------------------VPSYAVAQVLGSTLASLTLRVVFGG---- 153
SbNIP1;1        --------------------------------VPSYAVAQVLGSTLASLTLRVVFGG---- 148
PvNIP1;1        --------------------------------VPVYAAAQVMGATAASLTLRLLFGN---- 149
SiNIP1;2        --------------------------------VPAYAAAQVMGSTAASLTLRLLFGN---- 150
PvNIP1;2        --------------------------------VPSYAAAQVMGATAASLTLRLLFGN---- 146
SbNIP1;2        --------------------------------VPAYAAAQVMGATAASLTLRLLFGN---- 152
OsNIP1-3        --------------------------------VPAYAAAQVAGSAAASAALRALFGG---- 155
BdNIP1;2        --------------------------------VPAYAAAQLIGSTAAGLTLRLLFG----- 152
AtNIP4-1        ------------------------------HQVPLYIGAQFAGSLLASLTLRLMFKV---- 144
AtNIP4-2        ------------------------------YQVPLYIGAQLTGSLLASLTLRLMFNV---- 144
PtNIP1-4        ------------------------------WQVPLYIIAQLMGSILASGTLALALDV---- 145
PtNIP1-3        ------------------------------KQVPLYIAAQLLGSLLASGTLSLLFSV---- 134
GmNIP4;1        ------------------------------STVPLYFIAQVLGSFLASGTLYLLFEV---- 134
GhNIP1;1        --------------------------------VPAYVLAQVIGSTLAAGTLRLLF------ 146
GmNIP1;1        --------------------------------VPVYVVAQVVGSTLASGTLRLLF------ 137
GmNIP1;2        --------------------------------VPVYVVAQVVGSTLASATLRLLF------ 137
GmNIP1;5        --------------------------------VPAYVVAQLLGSILASGTLRLLF------ 137
GmNIP1;3        --------------------------------VPAYVIAQVVGATLASGTLRLIF------ 140
GmNIP1;4        --------------------------------VPAYVIAQVVGATLASGTLRLIF------ 134
AtNIP1-1        --------------------------------VPAYVISQVIGSTLAAATLRLLFGLDHDV 160
AtNIP1-2        --------------------------------VPAYVISQVIGSTLAAATLRLLFGLDQDV 157
AtNIP2-1        --------------------------------VPAYITVQVIGSTLASATLRLLFDLNNDV 152
PtNIP1-5        --------------------------------VPMYILAQVLGSTLASLTLRMLFHE---- 146
                                                 * *   *  .:  ..  :   :
```

```
                                 TM4
             ↓   ↓  ↓↓↓↓↓    ↓             ↓  ↓↓ ↓↓  ↓↓↓↓↓            ↓
ZmNIP1;1     ---RHEHFPGTLPT--GSEVQSLVIEIITTFYLMFVISGVATDNRA------------IG 188
SbNIP1;3     ---RHEHFPGTLPT--GSDVQSLVIEIITTFYLMFVISGVATDNRA------------IG 192
SiNIP1;1     ---RHEHFPGTLPT--GSDVQSLVLEIITTFYLMFVISGVATDNRA------------IG 185
PvNIP1;5     ---RHEHFPGTLPT--GSDVQSLVIEIITTFYLMFVISGVATDNRA------------IG 187
BdNIP1;1     ---RHEHFPGTLPG--GSEVQSLVLEFIITFYLMFVISGVATDNRA------------IG 187
OsNIP1-1     ---RHEHFPGTLPA--GSDVQSLVLEFIITFYLMFVISGVATDNRA------------IG 192
SbNIP1;4     ---EHLRASVTVHADGGSNIQSLVLEFFITFYLMFVIMGVATDDRA------------EG 208
SiNIP1;3     ---RHLVASVTVPS--GISSQSLVLEFIITFYLMFVIMAVATDDRA------------VG 202
PvNIP1;6     ---RHEPAPVTAPT--GSNMQSLVLEFIITFYLMFVIMAVATDDRA------------VG 238
PvNIP1;7     ---RHEPAPVTVPT--GSNMQSLVLEFIITFYLMFVIMAVATDDRA------------VG 202
OsNIP1-4     ---RHAPVPATLPG--GAHAQSLVIEFVITFYLMFVIMAVATDDQA------------VG 200
BdNIP1;3     ---RHEFVPVTAPT--GSNIQSLVTEFTTTFYLVFVVMAVATDDRA------------VG 196
OsNIP1-2     -ARGEHLFFGTTPA--GSMAQAAALEFVISFFLMFVVSGVATDNRA------------IG 214
OsNIP1-5     -ARGEHLFLGTTPA--GSMAQAAALEFVISFFLMFVVSSVATDNRA------------IG 214
PvNIP1;3     -ATAREHFFGTAPS--GSDAQAVALEFVISFYLMFVVSGVATDNRA------------IG 198
PvNIP1;4     -ATAREHFFGTAPS--GSDAQAVALEFLISFYLMFVVSGVATDNRA------------IG 198
SbNIP1;1     -ATAHEHFFGTAPS--GTVAQAVVLEFVISFYLMFVVSGVATDNRA------------IG 193
PvNIP1;1     ---AREHFFGTVPA--GSDVQSLVIELIISFNLMFVVCGVATDNRA------------IG 192
SiNIP1;2     ---AREHFFGTVPA--GSDVQSLVIELIISFNLMFVVSGVATDNRA------------IG 193
PvNIP1;2     ---AREHFFGTVPA--ASDAQSLVIEFIISFNLMFVVSGVATDNRA------------IG 189
SbNIP1;2     ---AREHFFGTVPA--GSDVQSLVIEFIISFNLMFVVSGVATDNRA------------IG 195
OsNIP1-3     ---APEHFFGTAPA--GSDVQSLAMEFIITFYLMFVVSGVATDNRA------------IG 198
BdNIP1;2     ----REHFVGTVPA--GSDVQSLVLEFIITFYLMFVVSGVATDNRA------------IG 194
AtNIP4-1     ---TPEAFFGTTPA--DSPARALVAEIIISFLLMFVISGVATDNRA------------VG 187
AtNIP4-2     ---TPKAFFGTTPT--DSSGQALVAEIIISFLLMFVISGVATDSRA------------TG 187
PtNIP1-4     ---TPEAFFGTVPV--GSDGQSLVLEIIISFLLMFVISGVSTDDRA------------VG 188
PtNIP1-3     ---TDEAYFGTIPV--GPDIRSFVTEIIISFLLMFVISGVATDNRA------------IG 177
GmNIP4;1     ---NEKTYFGTIPS--GSYIQSLVFEILTSFLLMFVVCAVSTDNRA------------IG 177
GhNIP1;1     -SGPHDVFAGTSPQ--GSDLQAFGIEFIITFYLMFIISGVATDNRA------------IG 191
GmNIP1;1     -SGKEAQFSGTLPS--GSNLQAFVIEFLITFFLMFVVSGVATDNRA------------IG 182
GmNIP1;2     -SGKETQFSGTLPS--GSNLQAFVIEFLITFFLMFVISGVATDDRA------------IG 182
GmNIP1;5     -MGNHDQFSGTVPN--GTNLQAFVFEFIMTFFLMFVICGVATDNRA------------VG 182
GmNIP1;3     -NGKNDHFAGTLPS--GSDLQSFVVEFIITFYLMFVISGVATDNRA------------IG 185
GmNIP1;4     -NGKSDHFTGTLPG--GSDLQSFVVEFIITFYLMFVISGVATDNRA------------IG 179
AtNIP1-1     CSGKHDVFIGSSPV--GSDLQAFTMEFIVTFYLMFIISGVATDNRAKLNIGTKCCNIQIG 218
AtNIP1-2     CSGKHDVFVGTLPS--GSNLQSFVIEFIITFYLMFVISGVATDNRA------------IG 203
AtNIP2-1     CSKKHDVFLGSSPS--GSDLQAFVMEFIITGFLMLVVCAVTTTKRT------------TE 198
PtNIP1-5     -QGNIQPIVNQYSDP-TSDLEAIVWEFIITFILMFTICGVATDPRA------------SK 192
                                .:    *:  :  *:: : .*:*  ::
```

```
                          TM5                                          TM6
                   ↓↓  ↓   ↓   ↓   ↓↓  ↓↓↓↓↓↓↓ ↓↓  ↓    ↓
ZmNIP1;1      ELAGLAVGATILLNVLIAGPVSGASMNPARSVGPALVSGEYTSIWVYVVGPVVGAVAGAW 248
SbNIP1;3      ELAGLAVGATILLNVLIAGPVSGASMNPARSVGPALVSGEYRSIWVYVVGPLVGAVAGAW 252
SiNIP1;1      ELAGLAVGATILLNVLIAGPVSGASMNPARSVGPALVSGQYRSIWVYVVGPVVGAVAGAW 245
PvNIP1;5      ELAGLAVGATILLNVLIAGPVSGASMNPARSVGPALVSGQYRSIWVYLVGPVVGAVAGAW 247
BdNIP1;1      ELAGLAVGATILLNVLIAGPISGASMNPARTVGPALVGSEYRSIWVYVVGPVAGAVAGAW 247
OsNIP1-1      ELAGLAVGATILLNVLIAGPISGASMNPARSLGPAMIGGEYRSIWVYIVGPVAGAVAGAW 252
SbNIP1;4      QMAGLAVGGTIILNALFAGPVSGASMNPARSIGPALVGNKYTSLWVYIFGPFAGAAAGAR 268
SiNIP1;3      QMAGLAVGGTIMLNALFAGPVSGASMNPVRSIGPALVGGKYTGLWVYIFGPFAGAAAGAW 262
PvNIP1;6      QMAGLAVGGTIMLNALFAGPVSGASMNPARSIGPALVGSKYRALWVYIFGPVAGAAAGAW 298
PvNIP1;7      QMAGLAVGGTIMLNALFAGPVSGASMNPARSIGPALVGSKYTALWVYIFGPVAGAAAGAW 262
OsNIP1-4      HMAGVAVGGTIMLNVLFAGPVSGASMNPARSIGPALVGSKYTALWVYILGPFAGAAAGAW 260
BdNIP1;3      SMAGVAVGATITLNALFSGPVTGASMNPARSIGPALVGGKYTSLWVYILGPFAGGAAGAW 256
OsNIP1-2      ELAGLAVGATVAVNVLFAGPVTGASMNPARSLGPAMVAGRYGGVWVYVAAPVSGTVCGAW 274
OsNIP1-5      ELAGLAVGATVAVNVLFAGPVTGASMNPARSLGPAMVAGRYGGVWVYVAAPVSGAVCGAW 274
PvNIP1;3      ELAGLAVGATVLLNVLFAGPITGASMNPARTLGPAIVAGRYRSVWVYVVGPVCGTVAGAW 258
PvNIP1;4      ELAGLAVGATVLLNVLFAGPITGASMNPARTLGPAIVAGRYRSIWVYVVGPVCGTVAGAW 258
SbNIP1;1      ELAGLAVGATVLLNVLVAGPITGASMNPARTLGPAIVAGRYRSIWVYMVGPVCGTVTGAW 253
PvNIP1;1      ELAGLAVGATVLLNVLFAGPISGASMNPARTLGPAIVAGRYAGIWVYFAGPIVGTVAGAW 252
SiNIP1;2      ELAGLAIGATVLLNVLFAGPISGASMNPARTLGPAIVAGRYAGIWVYFAGPILGTVAGAW 253
PvNIP1;2      ELAGLAVGATVLLNVLFAGPISGASMNPARTLGPAIVAGRYAGIWVYFAGPIVGTVAGAW 249
SbNIP1;2      ELAGLAVGATVLLNVLFAGPVSGASMNPARTLGPAIVVGRYAGIWVYFAGPICGTVAGAW 255
OsNIP1-3      ELAGLAVGATVLVNVLFAGPISGASMNPARTIGPAIILGRYTGIWVYIAGPVFGAVAGAW 258
BdNIP1;2      ELAGLAVGATVLLNVLFAGPISGASMNPARTLGPAMVAGRYKGIWVYIVGPVGGAVAGAW 254
AtNIP4-1      ELAGIAVGMTIMVNVFVAGPISGASMNPARSLGPALVMGVYKHIWVYIVGPVLGVISGGF 247
AtNIP4-2      ELAGIAVGMTIILNVFVAGPISGASMNPARSLGPAIVMGRYKGIWVYIVGPFVGIFAGGF 247
PtNIP1-4      DLAGIAVGMTILLNVFVAGPVSGASMNPARSIGPAVVKHQFKGLWVYIVGPIIGAIAGAF 248
PtNIP1-3      ELAGIAVGMTIMLNVFVAGPVSGASMNPARSLGPAIVMRQFKGIWVYIVGPPIGTILGAL 237
GmNIP4;1      KLGGIAVGMTIIVNVFIAGPISGASMNPARSLGPALVMWVYNGIWIYVVGPFVGAILGAT 237
GhNIP1;1      ELAGLAIGATVLINVMFAGPITGASMNPARSLGPAIVSNHYKGIWIYLMSPTLEAVSGAW 251
GmNIP1;1      ELAGIAVGSTVLLNVMFAGPITGASMNPARSIGPAIVHKEYRGIWIYLVSPTLGAVAGAW 242
GmNIP1;2      ELAGIAVGSTVLLNVMFAGPITGASMNPARSIGPAILHNEYRGIWIYIVSPTLGAVAGTW 242
GmNIP1;5      ELAGIAIGSTLLLNVIIGGPVTGASMNPARSLGPAFVHGEYEGIWIYLLAPVVGAIAGAW 242
GmNIP1;3      ELAGLAVGSTVLLNVMFAGPITGASMNPARSLGPAIVHHEYRGIWIYLVSPTLGAVAGTW 245
GmNIP1;4      ELAGLAVGSTVLLNVMFAGPITGASMNPARSLGPAIVHNEYKGIWIYLVSPTLGAVAGTW 239
AtNIP1-1      ELAGLAIGSTVLLNVLIAAPVSSASMNPGRSLGPALVYGCYKGIWIYLVAPTLGAIAGAW 278
AtNIP1-2      ELAGLAVGSTVLLNVIIAGPVSGASMNPGRSLGPAMVYSCYRGLWIYIVSPIVGAVSGAW 263
AtNIP2-1      ELEGLIIGATVTLNVIFAGEVSGASMNPARSIGPALVWGCYKGIWIYLLAPTLGAVSGAL 258
PtNIP1-5      DLSGVAIGGAVMFNAMIAGPITGASMNPARSLGPALVSGVYKNLWVYIVSPILGAMAAAA 252
               : *: :* :: .*.:... ::.***** *::***.:   :  :*:*. .*       .
```

```
ZmNIP1;1        AYNLIRFTNKPLREITKSTSFLK--STSRMNSAASA 282
SbNIP1;3        AYNLIRFTNKPLREITKSTSFLKSMSTNRMNSAAA- 287
SiNIP1;1        AYNLIRFTNKPLREITKSTSFLK--SMNRMNSASS- 278
PvNIP1;5        AYNLIRFTNKPLREITKSTSFLR--SMSRMNSTAV- 280
BdNIP1;1        SYNLIRFTNKPLREITKSTSFLR--SMSRMNSVAA- 280
OsNIP1-1        AYNIIRFTNKPLREITKSGSFLK--SMNRMNSST-- 284
SbNIP1;4        AYNLIRRTDKTLAEVTKSAS-RTN------------ 291
SiNIP1;3        AYNLIRHTDKTLAEITKSIS-RTNN----------- 286
PvNIP1;6        AYNLVRRTDRTLGEIKSAII-RPAN----------- 322
PvNIP1;7        AYNLIRRTDRTLGEIKSAIISRPTN----------- 287
OsNIP1-4        AYSLIRLTGDRTD----------------------- 273
BdNIP1;3        AYNLMRYTDKPAAVLSDVAKSTDRAA---------- 282
OsNIP1-2        AYNLLRFTDKPLRDIANTASFLRRSSRRS------- 303
OsNIP1-5        AYNLLRFTDKTLRVIAKSGSFLRRSSRRS------- 303
PvNIP1;3        AYNLVRFTDKPLREITKSGSFLRSARINGSTT---- 290
PvNIP1;4        AYNLVRFTDKPLREITKSGSFLRSARING------- 287
SbNIP1;1        AYNLVRFTDKPLREITKSGSFLRATGRTS------- 282
PvNIP1;1        AYNLIRFTDKPLREITQTSSFLRSARRN-------- 280
SiNIP1;2        AYNLIRFTDKPLREITQTSSFLRSARRN-------- 281
PvNIP1;2        AYNLIRFTDKPLREITQTSSFLRSVRRN-------- 277
SbNIP1;2        AYNLIRFTDKPLREITQTSSFLRSVRRT-------- 283
OsNIP1-3        AYNLIRFTDKPLREITMTASFIRSTRRN-------- 286
BdNIP1;2        AYNLIRFTNKPLREITRTGSFLRSARMG-------- 282
AtNIP4-1        VYNLIRFTDKPLRELTKSASFLRAVSPSHKGSSSKT 283
AtNIP4-2        VYNFMRFTDKPLRELTKSASFLRSVAQKDNASKSDG 283
PtNIP1-4        ACNLIRWTDKPLGELTKVGSFIKSGS-KNYAS---- 279
PtNIP1-3        CYNIIRFTDKPLREITKTASFLKSKN---------- 263
GmNIP4;1        CYNLIRYTDKPLREIGASSKIFKTSACTSAT----- 268
GhNIP1;1        VYNMVRYTDKPLREITKSASFPQSS---RNSG---- 280
GmNIP1;1        VYNSIRYTDKPLREITKSASFLKGV----ASR---- 270
GmNIP1;2        VYNTIRYTDKPLREITKSTSFLKGVGRSGSSR---- 274
GmNIP1;5        VYNIVRYTDKPLSEITKSASFLKGR---AASK---- 271
GmNIP1;3        AYNFIRYTNKPVREITKSASFLKGS----EAE---- 273
GmNIP1;4        AYNFIRYTNKPVREITKSASFLKGG----EAE---- 267
AtNIP1-1        VYNTVRYTDKPLREITKSGSFLKTVR--IGST---- 308
AtNIP1-2        VYNMVRYTDKPLREITKSGSFLKTVR--NGSSR--- 294
AtNIP2-1        IHKMLPSIQNAEPEFSKTGSSHKRVTDLPL------ 288
PtNIP1-5        VYSVLRVPEPAKPEDTNKSTYNNLNLHADP------ 282
                  . :
```
