# Supplementary material for: Genome-Wide Characterization of Major Intrinsic Proteins in Four Grass Plants and Their Non-Aqua Transport Selectivity Profiles with Comparative Perspective
Source: PLoS One. 2016 Jun 21;11(6):e0157735. doi: 10.1371/journal.pone.0157735 (PMC4915720; doi:10.1371/journal.pone.0157735)
Supplement: S11 Fig — The amino acid sequences were aligned using the Clustal Omega sequence alignment program. Two NPA motifs, the residues at H2, H5, LE1, and LE2 of the ar/R filter and FPs (P1-P5) are yellow, green and cyan, respectively. The SIP group-specific residues corresponding to structurally important residues in GlpF shown by Fu et al. (2000) are in open boxes. The group-specific residues at TM5, LE and TM6, which may also have structural and/or functional roles, are shown in blue boxes. The star (*) at the bottom of the alignment indicates the conserved residues. (PDF) [file pone.0157735.s011.pdf]

Figure S11

TM1

```
AQP1        ----MASEFKKKLFWRAVVAEFLATTLFVFISIGSALGFKYPVGNNQTAVQDN------- 49
GlpF        -----------MSQTSTLKGQCIAEFLGTGLLIFFGVGCVAALKVAGASFGQW------- 42
AtSIP1;1    ----------MMGVLKSAIGDMLMTFSWVVLSATFGIQTAAIISAGDFQAITW-----AP 45
AtSIP1;2    -----------MSAVKSALGDMVITFLWVILSATFGIQTAAIVSAVGFHGITW-----AP 44
GmSIP1;1    ----------MVSAIKAAIGDLVLTFLWVFFSSMLGLATNTITTALDLHHVSYNGFDYPS 50
GmSIP1;2    ----------MASAIKAAIGDLVLTFLWVFFSSMLGLVTNAITTALDLHHVSYNGFDYPS 50
GmSIP1;3    ----------MVGAIKAAIGDAVLTFMWVFCSSVLGIASGYITNALNLQHITYNGFPYAS 50
GmSIP1;4    ----------MVGAIKAAIGDAVLTFMWVFCSSVLGIASGYITNALNLQHITYNGFPYPS 50
PtSIP1;1    -----------MGAVKAAIGDAVFTFMWVFVSSMFGLFTNVIVTALGLQTLVW-----AP 44
PtSIP1;2    -----------MGAIKAASGDAVLTFMWVFVSSMFGLFTNLIVTALGLQTLVW-----AP 44
GhSIP1;2    -----------MRPIKIAFGDMLITFMWVFCSSMFGLFTSWIATAIGVQAISW-----AP 44
PvSIP1;1    --------MAMAAALRAAAADAVVTFLWVLCVSTLGASTAAVTSYLKLQGV------QYA 46
PvSIP1;2    --------MAMAAALRVAAADAVVTFLWVLCVSTLGASTAAVTSYLKLQGV------QYA 46
SiSIP1;1    --------MAIGAALRAAAADAVVTFLWVLCVSTLGASTAAVTSYLKLQGV------QYA 46
SbSIP1;2    --------MAMGPALRAAAADAVVTFLWVLCVSTLGASTAAVTSYLSLQGV------HYA 46
ZmSIP1;2    --------MAMGEALRAAAADAVVTFLWVLCVSTLGASTTAVTSYLRLQGV------HFA 46
BdSIP1;1    -------MAMAASAVKAAAADGVVTFLWVLCVSTLGASTAAVTRYLSLHEEGA----AYA 49
OsSIP1;1    ---------MAVAAVRAAAADAAVTFLWVLCVSTLGASTAAVTSYLRIHEG-I----HYA 46
SbSIP1;1    --------MAMGAAVRAAAADAVVTFLWVLCASALGASTAAVTSCLGVQEGAGG---HYA 49
ZmSIP1;1    --------MAMGATVRAAAADAVVTFLWVLCASALGASTAAVTSYLGVQEGAG----HYA 48
GmSIP1;5    -----------MGWIKAAIGDAILTSMWVFIISTLRIVTTEITVFLGLQPFFL-----AG 44
GmSIP1;6    -----------MGLIKAAIGDGVLTSMWVFIISTLRIVTTEVALFLGLQPLSL-----AG 44
GhSIP1;3    -----------MGVIKSAMADALLTSMWVFSMPFLKILTFEIAAFLGLRPFPL-----AA 44
PtSIP1;3    -----------MGAIKGAIVDGILTAMWVFSVPLLGVFSSIIATYVGVEAMSI-----AG 44
PtSIP1;4    -----------MGAIKGAIVDGILTCMWVFSVPLLGVFSSIIATYVGVEAMSI-----AG 44
PpSIP1;1    -----------MGLARKAVADASITFLWVFAMASLGAVSTSIAPSLGLDGP-----GKGK 44
PpSIP1;2    -----------MGLARKAVADASITFLWVFAMASLGAASTAIASSLGLDGP-----GKTK 44
SmSIP1.2    -----------MGMLKLILADAAISFLWVFCTSCIGAATEIIASCAGVE-------GDRK 42
PvSIP2;1    MSPAPPPPSRTRIRPWLVVGDLALAAAWVCAGALVKLLVYGPLGFG-GRPEAE------- 52
PvSIP2;2    MSPAPPPPSRARIRPWLVVGDLALAAAWVCAGALVKLLVYGPLGFG-GRPQAE------- 52
SiSIP2;1    MSPAPPPPSRARIRPWLVAGDLALAAAWVCAGALVKLLVYGPLGFG-GRPEAE------- 52
SbSIP2;1    MSPAP---SRPRIRPWLVVGDLALAAAWVCAGALVKLLVYGGLGLA-GRPEAE------- 49
ZmSIP2;1    MSPAP---SRPRIRPWLVVGDLALAAAWVCAGALVKLLVYGGLGLG-GRPEAE------- 49
OsSIP2;1    MSPAPPP-SRGRIRPWLVVGDLVVAAMWVCAGALVKLAVYGVLGLG-GRPEAD------- 51
PtSIP2;1    --------MVSKTR--LILSDFVVSLMWVWSGSLIKIFVFKVLGMG-HDSRGE------- 42
PtSIP2;2    --------MVSKTR--LIVSDFIVSIIWVWNGALIKMFVFKVLQMG-HDSRGE------- 42
AtSIP2;1    ---------MGRIG--LVVTDLVLSFMWIWAGVLVNILVHGVLGFSRTDPSGE------- 42
                              :
```

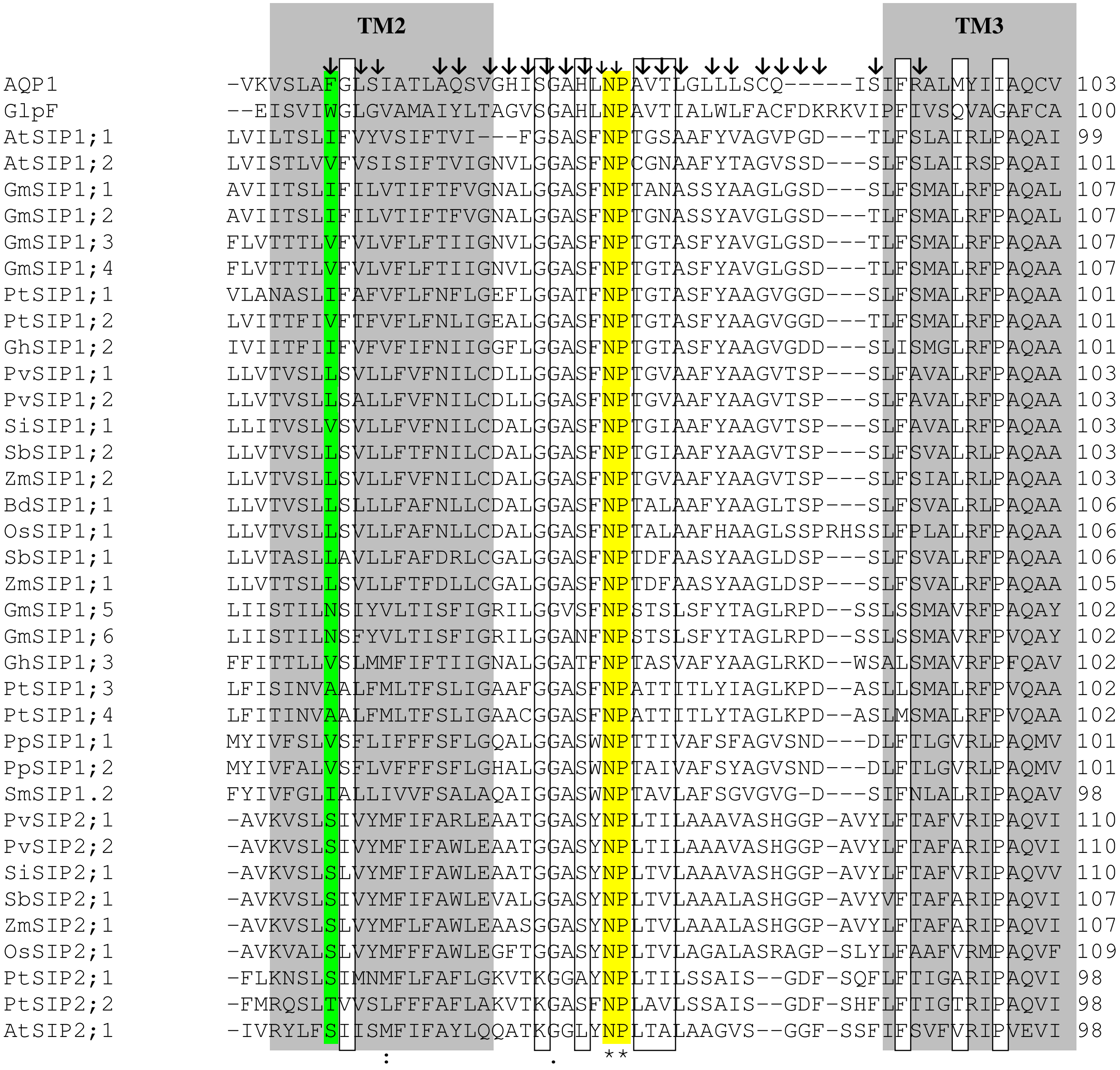
TM2
TM3
AQP1 -VKVSLAFGLSIATLAQSVGHISGAHLNPAVTLGLLLSCQ-----ISIFRALMYIIAQCV 103
GlpF --EISVIWGLGVAMAIYLTAGVSGAHLNPAVTIALWLFACFDKRKVIPFIVSQVAGAFCA 100
AtSIP1;1 LVILTSLIFVYVSIFTVI---FGSASFNPTGSAAFYVAGVPGD---TLFSLAIRLPAQAI 99
AtSIP1;2 LVISTLVVFVSISIFTVIGNVLGGASFNPCGNAAFYTAGVSSD---SLFSLAIRSPAQAI 101
GmSIP1;1 AVIITSLIFILVTIFTFVGNALGGASFNPTANASSYAAGLGSD---SLFSMALRFPAQAL 107
GmSIP1;2 AVIITSLIFILVTIFTFVGNALGGASFNPTGNASSYAVGLGSD---TLFSMALRFPAQAL 107
GmSIP1;3 FLVTTTLVFVLVFLFTIIGNVLGGASFNPTGTASFYAVGLGSD---TLFSMALRFPAQAA 107
GmSIP1;4 FLVTTTLVFVLVFLFTIIGNVLGGASFNPTGTASFYAVGLGSD---TLFSMALRFPAQAA 107
PtSIP1;1 VLANASLIFAFVFLFNFLGEFLGGATFNPTGTASFYAAGVGGD---SLFSMALRFPAQAA 101
PtSIP1;2 LVITTFIVFTFVFLFNLIGEALGGASFNPTGTASFYAAGVGGD---TLFSMALRFPAQAA 101
GhSIP1;2 IVIITFIIFVFVFIFNIIGGFLGGASFNPTGTASFYAAGVGDD---SLISMGLRFPAQAA 101
PvSIP1;1 LLVTVSLLSVLLFVFNILCDLLGGASFNPTGVAAFYAAGVTSP---SLFAVALRFPAQAA 103
PvSIP1;2 LLVTVSLLSALLFVFNILCDLLGGASFNPTGVAAFYAAGVTSP---SLFAVALRFPAQAA 103
SiSIP1;1 LLITVSLVSVLLFVFNILCDALGGASFNPTGIAAFYAAGVTSP---SLFAVALRFPAQAA 103
SbSIP1;2 LLVTVSLLSVLLFTFNILCDALGGASFNPTGIAAFYAAGVTSP---SLFSVALRLPAQAA 103
ZmSIP1;2 LLVTVSLLSVLLFVFNILCDALGGASFNPTGVAAFYAAGVTSP---SLFSIALRLPAQAA 103
BdSIP1;1 LLVTVSLLSLLLFAFNLLCDALGGASFNPTALAAFYAAGLTSP---SLFSVALRLPAQAA 106
OsSIP1;1 LLVTVSLLSVLLFAFNLLCDALGGASFNPTALAAFHAAGLSSPRHSSLFPLALRFPAQAA 106
SbSIP1;1 LLVTASLLAVLLFAFDRLCGALGGASFNPTDFAASYAAGLDSP---SLFSVALRFPAQAA 106
ZmSIP1;1 LLVTTSLLSVLLFTFDLLCGALGGASFNPTDFAASYAAGLDSP---SLFSVALRFPAQAA 105
GmSIP1;5 LIISTILNSIYVLTISFIGRILGGVSFNPSTSLSFYTAGLRPD--SSLSSMAVRFPAQAY 102
GmSIP1;6 LIISTILNSFYVLTISFIGRILGGANFNPSTSLSFYTAGLRPD--SSLSSMAVRFPVQAY 102
GhSIP1;3 FFITTLLVSLMMFIFTIIGNALGGATFNPTASVAFYAAGLRKD--WSALSMAVRFPFQAV 102
PtSIP1;3 LFISINVAALFMLTFSLIGAAFGGASFNPATTITLYIAGLKPD--ASLLSMALRFPVQAA 102
PtSIP1;4 LFITINVAALFMLTFSLIGAACGGASFNPATTITLYTAGLKPD--ASLMSMALRFPVQAA 102
PpSIP1;1 MYIVFSLVSFLIFFFSFLGQALGGASWNPTTIVAFSFAGVSND---DLFTLGVRLPAQMV 101
PpSIP1;2 MYIVFALVSFLVFFFSFLGHALGGASWNPTAIVAFSYAGVSND---DLFTLGVRLPAQMV 101
SmSIP1.2 FYIVFGLIALLIVVFSALAQAIGGASWNPTAVLAFSGVGVG-D---SIFNLALRIPAQAV 98
PvSIP2;1 -AVKVSLSIVYMFIFARLEAATGGASYNPLTILAAAVASHGGP-AVYLFTAFVRIPAQVI 110
PvSIP2;2 -AVKVSLSIVYMFIFAWLEAATGGASYNPLTILAAAVASHGGP-AVYLFTAFARIPAQVI 110
SiSIP2;1 -AVKVSLSLVYMFIFAWLEAATGGASYNPLTVLAAAVASHGGP-AVYLFTAFVRIPAQVV 110
SbSIP2;1 -AVKVSLSIVYMFIFAWLEVALGGASYNPLTVLAAALASHGGP-AVYVFTAFARIPAQVI 107
ZmSIP2;1 -AVKVSLSLVYMFLFAWLEAASGGASYNPLTVLAAALASHGGP-AVYLFTAFARIPAQVI 107
OsSIP2;1 -AVKVALSLVYMFFFAWLEGFTGGASYNPLTVLAGALASRAGP-SLYLFAAFVRMPAQVF 109
PtSIP2;1 -FLKNSLSIMNMFLFAFLGKVTKGGAYNPLTILSSAIS--GDF-SQFLFTIGARIPAQVI 98
PtSIP2;2 -FMRQSLTVVSLFFFAFLAKVTKGASFNPLAVLSSAIS--GDF-SHFLFTIGTRIPAQVI 98
AtSIP2;1 -IVRYLFSIISMFIFAYLQQATKGGLYNPLTALAAGVS--GGF-SSFIFSVFVRIPVEVI 98
: . **

```
                        TM3                                              TM4
AQP1        GAIVATAILSGITSSLTGNSLGRN-------------DLADGVNSGQGLGIEIIGTLQLV 150
GlpF        AALVYGLYYNLFFDFEQTHHIVRGSVESVDLAGTFSTYPNPHINFVQAFAVEMVITAILM 160
AtSIP1;1    GAAGGALAIMEFIPEKYKHMIGG---------------PSLQVDVHTGAIAETILSFGIT 144
AtSIP1;2    GAAGGAITIMEMIPEKYKTRIGGK--------------PSLQFGAHNGAISEVVLSFSVT 147
GmSIP1;1    GSVGGVLAVMEVMPPKYRHLIGG---------------PSLKVSLHTGAIAEGVLTFVIT 152
GmSIP1;2    GSVGGVLAVMEVMPPKYRHLIGG---------------PSLKVSLHTGAIAEGVLTFVIT 152
GmSIP1;3    GAAGGAMAIMEVIPAKYRHMIGG---------------PSLKVDLHTGAVAEGVLTFVIT 152
GmSIP1;4    GAAGGALAIMEVIPAKYRHMIGG---------------PSLKVDLHTGAVAEGVLTFVIT 152
PtSIP1;1    GSVGGSLAILEVMPLQYKHMLGG---------------PTLQVDLQTGGLAEGVLTFLMT 146
PtSIP1;2    GAVGGALAIMEVMPVQYKHMLGG---------------PTLQVDLHTGGLAEGVLTFLMS 146
GhSIP1;2    GAVGGALAITEVMPEQYKHMIVA---------------PSLKVDTHTGAIAEGVLTFVIT 146
PvSIP1;1    GAVGGALAISELMPAQYKHMLGG---------------PSLKVDPHTGAVAELVLTFVIT 148
PvSIP1;2    GAVGGALAISELMPAQYKHMLGG---------------PSLKVDPHTGAVAELVLTFVIT 148
SiSIP1;1    GAVCGALAISELMPAQYKHMLGG---------------PSLKVDPHTGAVAELVLTFVIT 148
SbSIP1;2    GAVGGALAISELMPAQYKHMLGG---------------PSLKVDPHTGAIAELVLTFVIT 148
ZmSIP1;2    GAVGGALAISELMPAQYRHMLGG---------------PSLKVDPHTGAGAELVLTFVIT 148
BdSIP1;1    GAVGGALAISELMPAQYKHMLGG---------------PSLKVDPHTGAVAEGLLTFVIT 151
OsSIP1;1    GAVGGAMAISELMPEQYKHMLGG---------------PSLKVDLHTGAAAELVLTFVIT 151
SbSIP1;1    GAVGGALAISELMPAQYKHTLAG---------------PSLKVDPHTGALAEGVLTFVVT 151
ZmSIP1;1    GAVGGALAISELMPAQYKHTLAG---------------PSLKVDPHTGALAEGVLTFVIT 150
GmSIP1;5    GGAVGIKTLLLVMPSHYKDMLKG---------------PFLKVDLHSGAVAEGLLTFIHN 147
GmSIP1;6    GGAVGVKTLLLVMPSKYNDMLKG---------------PFLKVDLHSGAVAEGVLTFTHN 147
GhSIP1;3    GGVVGVKTVLGVLPREYKETIKG---------------PSLKVDIQTGFLAEGLLTFGLC 147
PtSIP1;3    GGVGGAMAIRGVMPKHYRHVLKGG--------------PSLRVDLHTGAIAEGVLTFLIC 148
PtSIP1;4    GGVAGAMAITEVMPKQYRYVLRGG--------------PSLKVDLHTGAIAEGVLTFLIC 148
PpSIP1;1    GAVGGALTIWEVMPKKYKHTLGG---------------PKLKVPLETGVAAETILTFTIT 146
PpSIP1;2    GAVGGALAILEVMPKKYKHMLGG---------------PKLKVPLQTGVIAEAILTFTIT 146
SmSIP1.2    GSAAGALAIFELMPSSFKRTLGG---------------PSLKVDLRTGAIAEGLLSFIMS 143
PvSIP2;1    GAVLGVKLIQFTFPN-----VGKG--------------ARLSVGAHHGALAEGLATFMVV 151
PvSIP2;2    GAVLGVKLIQFTFPN-----VGKG--------------ARLSVGAHHGALAEGLATFMVV 151
SiSIP2;1    GAVLGVKLIQFTFPN-----VGKG--------------ARLSVGAHHGALAEGLATFMVV 151
SbSIP2;1    GAVLGVNLIQLTFPN-----VGKG--------------ARLSVGAHHGALAEGLATFMVV 148
ZmSIP2;1    GAVLGVKLIQVTFPN-----VGKG--------------ARLSVGAHHGALAEGLATFMVV 148
OsSIP2;1    GSILGVKLIRAALPK-----VGKG--------------APLSVGVHHGALAEGLATFMVV 150
PtSIP2;1    GSITGVRLFIDTFPE-----IGLG--------------PRLTVDIHKGALTEGLLTFAIV 139
PtSIP2;2    GSITAVRLLIDTFPE-----IGRG--------------PRLNVDIHKGALTEGLLAFGVV 139
AtSIP2;1    GSILAVKHIIHVFPE-----IGKG--------------PKLNVAIHHGALTEGILTFFIV 139
            ..                 :                 .      .     * : :
```

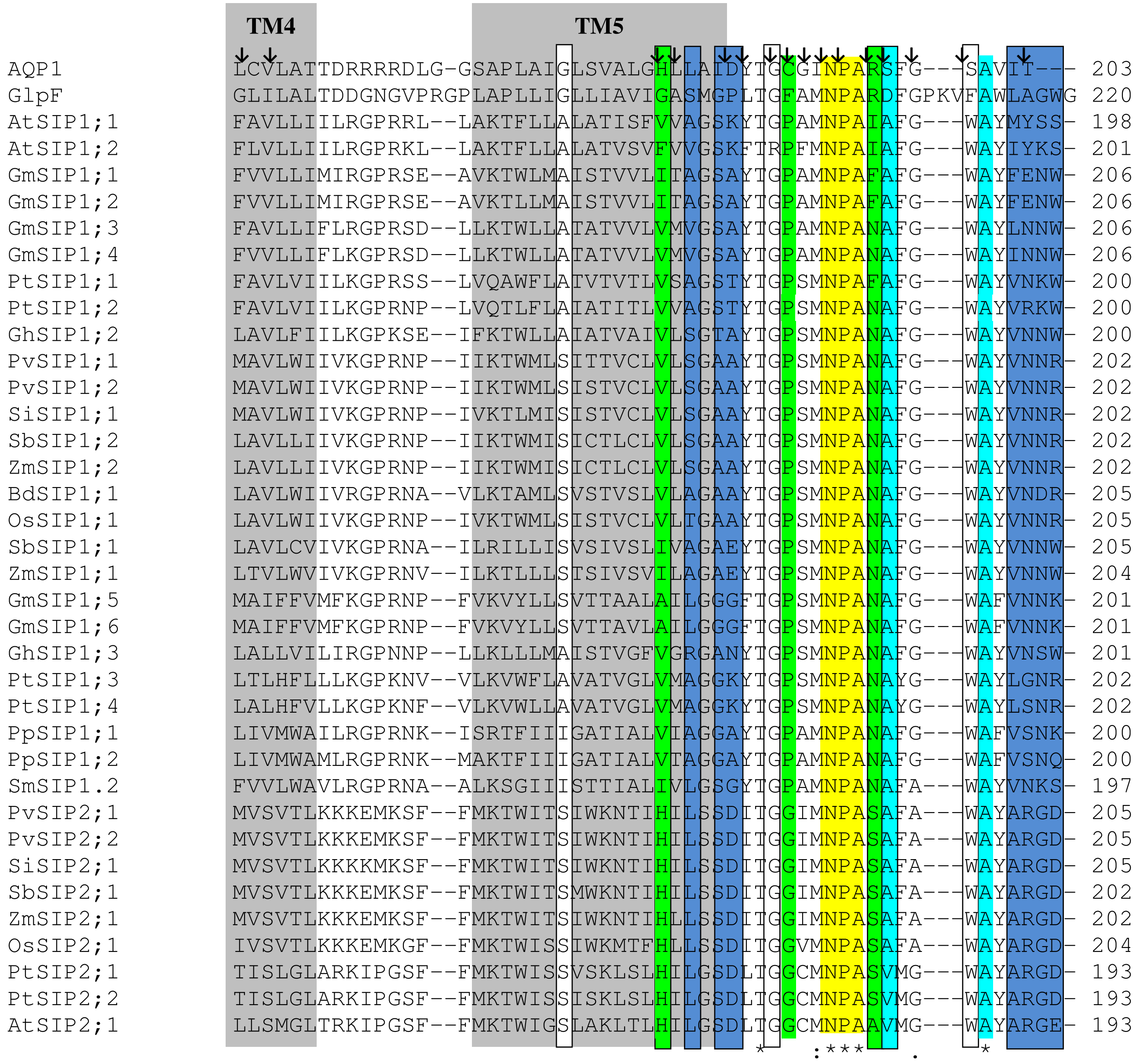
TM4
TM5
AQP1 LCVLATTDRRRRDLG-GSAPLAIGLSVALGHLLAIDYTGCGINPARSFG---SAVIT--- 203
GlpF GLILALTDDGNGVPRGPLAPLLIGLLIAVIGASMGPLTGFAMNPARDFGPKVFAWLAGWG 220
AtSIP1;1 FAVLLIILRGPRRL--LAKTFLLALATISFVVAGSKYTGPAMNPAIAFG---WAYMYSS- 198
AtSIP1;2 FLVLLIILRGPRKL--LAKTFLLALATVSVFVVGSKFTRPFMNPAIAFG---WAYIYKS- 201
GmSIP1;1 FVVLLIMIRGPRSE--AVKTWLMAISTVVLITAGSAYTGPAMNPAFAFG---WAYFENW- 206
GmSIP1;2 FVVLLIMIRGPRSE--AVKTLLMAISTVVLITAGSAYTGPAMNPAFAFG---WAYFENW- 206
GmSIP1;3 FAVLLIFLRGPRSD--LLKTWLLATATVVLVMVGSAYTGPAMNPANAFG---WAYLNNW- 206
GmSIP1;4 FVVLLIFLKGPRSD--LLKTWLLATATVVLVMVGSAYTGPAMNPANAFG---WAYINNW- 206
PtSIP1;1 FAVLVIILKGPRSS--LVQAWFLATVTVTLVSAGSTYTGPSMNPAFAFG---WAYVNKW- 200
PtSIP1;2 FAVLVIILKGPRNP--LVQTLFLAIATITLVVAGSTYTGPSMNPANAFG---WAYVRKW- 200
GhSIP1;2 LAVLFIILKGPKSE--IFKTWLLAIATVAIVLSGTAYTGPSMNPANAFG---WAYVNNW- 200
PvSIP1;1 MAVLWIIVKGPRNP--IIKTWMLSITTVCLVLSGAAYTGPSMNPANAFG---WAYVNNR- 202
PvSIP1;2 MAVLWIIVKGPRNP--IIKTWMLSISTVCLVLSGAAYTGPSMNPANAFG---WAYVNNR- 202
SiSIP1;1 MAVLWIIVKGPRNP--IVKTLMISISTVCLVLSGAAYTGPSMNPANAFG---WAYVNNR- 202
SbSIP1;2 LAVLLIIVKGPRNP--IIKTWMISICTLCLVLSGAAYTGPSMNPANAFG---WAYVNNR- 202
ZmSIP1;2 LAVLLIIVKGPRNP--IIKTWMISICTLCLVLSGAAYTGPSMNPANAFG---WAYVNNR- 202
BdSIP1;1 LAVLWIIVRGPRNA--VLKTAMLSVSTVSLVLAGAAYTGPSMNPANAFG---WAYVNDR- 205
OsSIP1;1 LAVLWIIVKGPRNP--IVKTWMLSISTVCLVLTGAAYTGPSMNPANAFG---WAYVNNR- 205
SbSIP1;1 LAVLCVIVKGPRNA--ILRILLISVSIVSLIVAGAEYTGPSMNPANAFG---WAYVNNW- 205
ZmSIP1;1 LTVLWVIVKGPRNV--ILKTLLLSTSIVSVILAGAEYTGPSMNPANAFG---WAYVNNW- 204
GmSIP1;5 MAIFFVMFKGPRNP--FVKVYLLSVTTAALAILGGGFTGPSMNPANAFG---WAFVNNK- 201
GmSIP1;6 MAIFFVMFKGPRNP--FVKVYLLSVTTAVLAILGGGFTGPSMNPANAFG---WAFVNNK- 201
GhSIP1;3 LALLVILIRGPNNP--LLKLLLMAISTVGFVGRGANYTGPSMNPANAFG---WAYVNSW- 201
PtSIP1;3 LTLHFLLLKGPKNV--VLKVWFLAVATVGLVMAGGKYTGPSMNPANAYG---WAYLGNR- 202
PtSIP1;4 LALHFVLLKGPKNF--VLKVWLLAVATVGLVMAGGKYTGPSMNPANAYG---WAYLSNR- 202
PpSIP1;1 LIVMWAILRGPRNK--ISRTFIIIGATIALVIAGGAYTGPAMNPANAFG---WAFVSNK- 200
PpSIP1;2 LIVMWAMLRGPRNK--MAKTFIIIGATIALVTAGGAYTGPAMNPANAFG---WAFVSNQ- 200
SmSIP1.2 FVVLWAVLRGPRNA--ALKSGIIISTTIALIVLGSGYTGPAMNPANAFA---WAYVNKS- 197
PvSIP2;1 MVSVTLKKKEMKSF--FMKTWITSIWKNTIHILSSDITGGIMNPASAFA---WAYARGD- 205
PvSIP2;2 MVSVTLKKKEMKSF--FMKTWITSIWKNTIHILSSDITGGIMNPASAFA---WAYARGD- 205
SiSIP2;1 MVSVTLKKKKMKSF--FMKTWITSIWKNTIHILSSDITGGIMNPASAFA---WAYARGD- 205
SbSIP2;1 MVSVTLKKKEMKSF--FMKTWITSMWKNTIHILSSDITGGIMNPASAFA---WAYARGD- 202
ZmSIP2;1 MVSVTLKKKEMKSF--FMKTWITSIWKNTIHLLSSDITGGIMNPASAFA---WAYARGD- 202
OsSIP2;1 IVSVTLKKKEMKGF--FMKTWISSIWKMTFHLLSSDITGGVMNPASAFA---WAYARGD- 204
PtSIP2;1 TISLGLARKIPGSF--FMKTWISSVSKLSLHILGSDLTGGCMNPASVMG---WAYARGD- 193
PtSIP2;2 TISLGLARKIPGSF--FMKTWISSISKLSLHILGSDLTGGCMNPASVMG---WAYARGD- 193
AtSIP2;1 LLSMGLTRKIPGSF--FMKTWIGSLAKLTLHILGSDLTGGCMNPAAVMG---WAYARGE- 193
* :*** . *

```
                               TM6
AQP1        -------HNFSNHWIFWVGPFIGGALAVLIYDFILAPRSSDLTDRVKVWTSGQVEEYDLD 256
GlpF        NVAFTGGRDIPYFLVPLFGPIVGAIVGAFAYRKLIGRHLPCDICVVEEKETTTPSEQKAS 280
AtSIP1;1    ------HNTWDHIYVYWISSFVGALSAALLFRSIFPPPRPQKKKQKKA------------ 240
AtSIP1;2    ------HNTWDHFYVYWISSYTGAILSAMLFRIIFPAPPLVQKKQKKA------------ 243
GmSIP1;1    ------HNTWDQFYVYWICPFFGAILAAWLFRIVIPP-APRVVKQKKA------------ 247
GmSIP1;2    ------HNTWDQFYVYWICPFFGAILAAWLFRIVFP---PRVVKQKKA------------ 245
GmSIP1;3    ------HNTWDQFYVYWICPFTGAILAAWLFRAVFPPPPPPEVKQKKA------------ 248
GmSIP1;4    ------HNTWDQFYVYWICPFAGAILAAWLFRAVFPPPSPPEVKQKKA------------ 248
PtSIP1;1    ------HNTWEQLYVYWICPFIGAILAAWVFRVVFPPPAP--KQKKT------------- 239
PtSIP1;2    ------HNTWEQLYVYWICPFIGAILASWVFRAVFPPPAP--KQKKA------------- 239
GhSIP1;2    ------HNTWDQFYVYWICPFIGAILAAWVFRLFFPPPSPVKKAKKTRKPKRA------- 247
PvSIP1;1    ------HNTWEQFYVYWICPFIGAVLAAWIFRALFLAPPPKPKAKKA------------- 243
PvSIP1;2    ------HNTWEQFYVYWICPFIGAVLAAWIFRALFLAPPPKPKAKKA------------- 243
SiSIP1;1    ------HNTWEQFYVYWISPFIGAVLAAWIFKALFLAPPPKPKAKKA------------- 243
SbSIP1;2    ------HNTWEQFYVYWICPFIGAILAAWIFRALFLAPPPKPKAKKA------------- 243
ZmSIP1;2    ------HNTWEQFYVYWICPFIGAILAAWIFRAMFLTPPPKPKAKKA------------- 243
BdSIP1;1    ------HNTWEQLYVYWICPFVGATLAAWTFRAVFPPPAPKPKAKKA------------- 246
OsSIP1;1    ------HNTWEQFYVYWICPFVGAVLAAWVFRAVFPPPAPKPKAKKA------------- 246
SbSIP1;1    ------HNTWEQLYVYWICPFIGALLAGWIFRAVFLPPAPKPKTKKA------------- 246
ZmSIP1;1    ------HNTWEQLYVYWICPFIGAMLAGWIFRVVFLPPAPKPKTKKA------------- 245
GmSIP1;5    ------HNTWEQFYVYWIGPFIGASSAALIFRSMFMPP---IKQKKA------------- 239
GmSIP1;6    ------HNTWEQFYVYWICPFIGASSAALIFRSMFMPP---IKQKKA------------- 239
GhSIP1;3    ------HNSWEHYYVYWMGPLIGATLAAWVFRFLFSPSSS-IKEKKA------------- 241
PtSIP1;3    ------HTTWDFFYVYWICPFIGAILAAFVSKFLFKAAP--IKEKKA------------- 241
PtSIP1;4    ------HTTWDFFYVYWICPFIGATLAALISKFLFKAPP--IKDKKA------------- 241
PpSIP1;1    ------HTSWEHFAVYWAGPMIGTICAVLTFNLIFGRHQVKGQAT--KKSKAKKTKKPGS 252
PpSIP1;2    ------HTSWDHFAVYWAGPMIGTIFAVWAFNLLFGPHSQATQASDSKKLKANKAKKSGS 254
SmSIP1.2    ------HNTNEHLYVYWLTPLLGSLAASMVFKRMFALGQNKEKTA--------------- 236
PvSIP2;1    ------HTTFDHLLVYWLAPLQATLLGVWVVTFLTKPKKIKEQ----------------- 242
PvSIP2;2    ------HTTFDHLLVYWLAPLQATLLGVWVVTFLTKPKKIKEQ----------------- 242
SiSIP2;1    ------HTTFDHLLVYWLAPLQATLLGVWVVTFLTKPKKIKEQEADENKTKKE------- 252
SbSIP2;1    ------HTTFDHLLVYWLAPLQATLLGVWAVTYLTKSKKIKEQEADENKTKKE------- 249
ZmSIP2;1    ------HTTFDHLLVYWLAPLQATLLGVWAVTFFTKPKKIKEQKVDENKIKKE------- 249
OsSIP2;1    ------HTTFDHLLVYWLAPLQATLLGVWVVTLLTKPKKIEEE-ADESKTKKE------- 250
PtSIP2;1    ------HITKEHILVYWLAPIEGTLLAVWTFKLLFRPQKQDEKEKLKGKTE--------- 238
PtSIP2;2    ------HITKEHILVYWLAPIQGALLAAYTFKLLFRPQKQDEKEKLKGKTD--------- 238
AtSIP2;1    ------HITKEHILVYWLGPVKATLLAVWFFKVVFKPLTE-EQEKPKAKSE--------- 237
                       :    .  .   .       .
```

AQP1	ADDINSRVEMKPK------------	269
GlpF	L------------------------	281
AtSIP1;1	-------------------------	
AtSIP1;2	-------------------------	
GmSIP1;1	-------------------------	
GmSIP1;2	-------------------------	
GmSIP1;3	-------------------------	
GmSIP1;4	-------------------------	
PtSIP1;1	-------------------------	
PtSIP1;2	-------------------------	
GhSIP1;2	-------------------------	
PvSIP1;1	-------------------------	
PvSIP1;2	-------------------------	
SiSIP1;1	-------------------------	
SbSIP1;2	-------------------------	
ZmSIP1;2	-------------------------	
BdSIP1;1	-------------------------	
OsSIP1;1	-------------------------	
SbSIP1;1	-------------------------	
ZmSIP1;1	-------------------------	
GmSIP1;5	-------------------------	
GmSIP1;6	-------------------------	
GhSIP1;3	-------------------------	
PtSIP1;3	-------------------------	
PtSIP1;4	-------------------------	
PpSIP1;1	EGQAAKSKGLKKESTGNAGDKMKAS	277
PpSIP1;2	EGESAKDKKRGEGLSENAAGKVKAS	279
SmSIP1.2	-------------------------	
PvSIP2;1	-------------------------	
PvSIP2;2	-------------------------	
SiSIP2;1	-------------------------	
SbSIP2;1	-------------------------	
ZmSIP2;1	-------------------------	
OsSIP2;1	-------------------------	
PtSIP2;1	-------------------------	
PtSIP2;2	-------------------------	
AtSIP2;1	-------------------------	
