# Supplementary material for: Genome-Wide Characterization of Major Intrinsic Proteins in Four Grass Plants and Their Non-Aqua Transport Selectivity Profiles with Comparative Perspective
Source: PLoS One. 2016 Jun 21;11(6):e0157735. doi: 10.1371/journal.pone.0157735 (PMC4915720; doi:10.1371/journal.pone.0157735)
Supplement: S1 Table — (PDF) [file pone.0157735.s012.pdf]

Table S1: MIPs discarded from the four grass plants

Gene Name	Locus name	Phytozome accessions	Genomic Location	PPL (aa)	Maximum Identity with MIPs of the four plants (%)	SCL[y]	Reason(s) for exclusion from further studies
**psdo*PvPIP1;8***	Pavir.Gb01084	Pavir.Gb01084.1	Chr07b: 13931659 - 13933646	322	*PvPIP1;1*(100)	PLAS	Identical with *PvPIP1;1* except N-terminal extension
**psdo*PvPIP1;9***	Pavir.Gb01084	Pavir.Gb01084.2	Chr07b: 13931659 - 13933701	288	*PvPIP1;1*(100)	PLAS	Identical with *PvPIP1;1*
**psdo*PvPIP1;10***	Pavir.Gb01084	Pavir.Gb01084.4	Chr07b: 13931659 - 13933701	216	*PvPIP2;1*(100)	CYTO	Missing of first NPA motif
**psdo*PvPIP2;15***	Pavir.Aa01314	Pavir.Aa01314.1	Chr01a: 16966260 - 16967600	71	*PvPIP2;9*(96)	MITO	Short length
**psdo*PvPIP2;16***	Pavir.Bb01320	Pavir.Bb01320.3	Chr02b: 27409376 - 27411877	220	*PvPIP2;7*(94)	PLAS	Missing of first NPA motif
**psdo*PvPIP2;17***	Pavir.Ga01149	Pavir.Ga01149.2	Chr07a: 14124713 - 14126981	266	*PvPIP2;4*(100)	PLAS	Identical with *PvPIP2;4*
**psdo*PvPIP2;18***	Pavir.Ba02483	Pavir.Ba02483.1	Chr02a: 37691323 - 37694961	341	*PvPIP2;6*(100)	CYSK	Identical with PvPIP2;6 except extension
**psdo*PvPIP2;19***	Pavir.Ba02478	Pavir.Ba02478.2	Chr02a: 37576388 - 37578287	212	*PvPIP2;9*(96)	PLAS	Short length and missing 2nd NPA motif
**psdo*PvPIP2;20***	Pavir.J11885	Pavir.J11885.1	contig142748: 1772 - 2888	286	*PvPIP2;2*(78)	PLAS	Missing 2nd NPA motif
**psdo*PvPIP2;21***	Pavir.J38196	Pavir.J38196.1	contig74028: 61 - 648	196	*PvPIP2;8*(99)	PLAS	Short length and missing 2nd NPA motif
**psdo*PvPIP2;22***	Pavir.J11644	Pavir.J11644.1	contig140997: 365 - 1545	287	*PvPIP2;14*(100)	PLAS	Identical with *PvPIP2;14*
**psdo*PvPIP2;23***	Pavir.J11644	Pavir.J11644.1	contig140997: 365 - 1545	287	*PvPIP2;14*(100)	PLAS	Identical with *PvPIP2;14*
**psdo*PvPIP2;24***	Pavir.J16361	Pavir.J16361.1	contig181833: 21 - 636	175	*PvPIP2;13*(78)	VACU	Short length
**psdo*PvTIP2;6***	Pavir.Ab02612	Pavir.Ab02612.1	Chr01b: 48023571 - 48024395	195	*PvTIP2;3*(98)	VACU	Short length and missing 2nd NPA motif
**psdo*PvTIP2;7***	Pavir.Aa00837	Pavir.Aa00837.1	Chr01a: 10068382 - 10069265	179	*PvTIP2;3*(98)	PLAS	Short length and missing 2nd NPA motif
**osi*PvTIP4;7***	Pavir.Ea00003	Pavir.Ea00003.4	Chr05a: 159705 - 160973	250	*PvTIP4;1*(100)	VACU	Identical with *PvTIP4;1*

**osi*PvTIP4;8***	Pavir.Ea00003	Pavir.Ea00003.3	Chr05a: 159660 - 160975	250	*PvTIP4;1*(100)	VACU	Identical with *PvTIP4;1*
**osi*PvTIP4;9***	Pavir.Ea00003	Pavir.Ea00003.2	Chr05a: 157527 - 160973	250	*PvTIP4;1*(100)	VACU	Identical with *PvTIP4;1*
**osi*PvTIP4;10***	Pavir.J30482	Pavir.J30482.1	contig355910: 206 - 1208	256	*PvTIP4;2*(100)	CYTO	Identical with *PvTIP4;2*
**psdo*PvTIP4;11***	Pavir.Eb00022	Pavir.Eb00022.1	Chr05b: 510621 - 511227	108	*PvTIP4;3*(100)	VACU	Short length
**osi *PvTIP4;12***	Pavir.J20433	Pavir.J20433.1	contig222165: 1187 - 2025	239	*PvTIP4;3*(100)	VACU	Identical with *PvTIP4;3*
**psdo*PvTIP13;4***	Pavir.Ea00047	Pavir.Ea00047.1	Chr05a: 812852 - 813820	228	*PvTIP4;4*(87)	CYTO	Deletion in TM2
**psdo*PvNIP1;8***	Pavir.J36379	Pavir.J36379.2	contig59709: 2228 - 4657	275	*PvNIP1;2*(76)	PLAS	2nd NPA motif missing
**osi*PvNIP1;9***	Pavir.Eb00236	Pavir.Eb00236.1	Chr05b: 3774472 - 3776780	300	*PvNIP1;3*(100)	VACU	Identical with *PvNIP1;3*
**osi*PvNIP1;10***	Pavir.Eb00236	Pavir.Eb00236.4	Chr05b: 3774478 - 3776780	290	*PvNIP1;3*(100)	PLAS	Identical with *PvNIP1;3*
**iso*PvNIP1;11***	Pavir.Eb00236	Pavir.Eb00236.2	Chr05b: 3774233 - 3776780	298	*PvNIP1;3*(100)	PLAS	Identical with *PvNIP1;3*
**psdo*PvNIP1;12***	Pavir.Aa02820	Pavir.Aa02820.1	Chr01a: 60219865 - 60220905	81	*PvNIP1;5*(86)	CHLO	Short length
**psdo*PvNIP1;13***	Pavir.J09715	Pavir.J09715.1	contig125990: 80 - 2298	206	*PvNIP1;5*(97)	PLAS	2nd NPA motif missing
**psdo*PvNIP2;5***	Pavir.Ab02995	Pavir.Ab02995.2	Chr01b: 52353467 - 52356606	210	*PvNIP2;1*(100)	PLAS	2nd NPA motif missing
**psdo*PvNIP2;6***	Pavir.J30578	Pavir.J30578.1	contig357494: 1 - 1165	249	*PvNIP2;4*(32)	PLAS	Deletion in N-terminal and TM2
**psdo*PvNIP2;7***	Pavir.J25538	Pavir.J25538.1	contig282291: 242 - 1449	189	*PvNIP3;8*(25)	PLAS	Short length and both NPA motif missing
**psdo*PvNIP2;8***	Pavir.Aa00406	Pavir.Aa00406.2	Chr01a: 4613561 - 4614774	123	*PvNIP2;2*(100)	MITO	Short length and 2nd NPA motif missing
**psdo*PvNIP2;9***	Pavir.Db01588	Pavir.Db01588.2	Chr04b: 35917588 - 35920941	214	*PvNIP2;3*(100)	PLAS	Short length and 2nd NPA motif missing
**psdo*PvNIP2;10***	Pavir.J30123	Pavir.J30123.1	contig349457: 82 - 914	203	*PvNIP2;1*(62)	PLAS	Short length and 2nd NPA motif missing
**psdo*PvNIP2;11***	Pavir.J17718	Pavir.J17718.1	contig194795: 554 - 873	69	*PvNIP3;4*(94)	EXTR	Short length
**iso*PvNIP3;11***	Pavir.Fa01950	Pavir.Fa01950.3	Chr06a: 45024641 - 45028094	330	*PvNIP3;4*(100)	CYTO	Identical with *PvNIP3;4*
**osi*PvNIP3;12***	Pavir.Fa01950	Pavir.Fa01950.1	Chr06a: 45025793 - 45028094	338	*PvNIP3;4*(100)	CHLO	Identical with *PvNIP3;4*
**psdo*PvNIP3;13***	Pavir.Fb00241	Pavir.Fb00241.1	Chr06b: 4351292 - 4355053	260	*PvNIP3;5*(91)	VACU	First NPA motif missing
**psdo*PvNIP3;14***	Pavir.J33306	Pavir.J33306.1	contig406555: 94 - 518	127	*PvNIP3;5*(90)	EXTR	Short length
**osi*PvNIP3;15***	Pavir.J17719	Pavir.J17719.1	contig194795: 1290 - 2237	291	*PvNIP3;7*(100)	PLAS	Identical with *PvNIP3;7*

**iso*PvNIP3;16***	Pavir.J35034	Pavir.J35034.1	contig50657: 4142 - 5117	295	*PvNIP3;8*(100)	CYTO	Identical with *PvNIP3;8*
**psdo*PvNIP3;17***	Pavir.Fa01951	Pavir.Fa01951.1	Chr06a: 45028513 - 45028918	105	*PvNIP3;8*(99)	VACU	Short length
**psdo*PvNIP3;18***	Pavir.Gb00704	Pavir.Gb00704.1	Chr07b: 8126685 - 8137810	123	*PvNIP3;1*(23)	EXTR	Short length and both NPA missing
**osi*PvNIP4;3***	Pavir.Ea00764	Pavir.Ea00764.1	Chr05a: 10523338 - 10525337	331	*PvNIP4;1*(100)	PLAS	Identical with *PvNIP4;1*
**osi*PvNIP4;4***	Pavir.Ea00764	Pavir.Ea00764.4	Chr05a: 10523338 - 10525337	246	*PvNIP4;1*(100)	VACU	Identical with *PvNIP4;1*
**psdo*PvNIP4;5***	Pavir.J37827	Pavir.J37827.1	contig71022: 2 - 1506	211	*PvNIP4;2*(83)	PLAS	Deletion in C-terminal
**psdo*PvSIP1;3***	Pavir.Ha01154	Pavir.Ha01154.1	Chr08a: 31279124 - 31279924	107	*PvSIP1;1*(68)	CHLO	Short length
**osi*PvSIP2;3***	Pavir.J37350	Pavir.J37350.2	contig67361: 865 - 2938	242	*PvSIP2;2*(100)	PLAS	Identical with *PvSIP2;2*
**psdo*SiPIP1;7***	Si010758m.g	Si011040m	scaffold_7: 26986436 - 26988672	215	*SiPIP1;3*(97)	PLAS	2nd NPA missing
**psdo*SiPIP2;9***	Si030703m.g	Si030819m	scaffold_2: 13905399 - 13908725	266	*SiPIP2;5*(94)	PLAS	Deletion of C terminal sequences
**psdo*SiTIP3;3***	Si012227m.g	Si012227m	scaffold_7: 25550166 - 25551608	242	*SiTIP3;2*(56)	CHLO	Partial deletion in 2nd NPA
**psdo*SiNIP1;4***	Si005574m.g	Si005574m	scaffold_5: 12715473 - 12715961	64	*SiNIP1;2*(76)	CHLO	Both NPA missing
**psdo*SiNIP2;5***	Si036817m.g	Si037421m	scaffold_9: 13789012 - 13792948	222	*SiNIP2;4*(100)	PLAS	Identical to same length of SiNIP2;4
**psdo*SiNIP2;6***	Si007007m.g	Si007264m	scaffold_4: 8475698 - 8479630	215	*SiNIP2;2*(100)	PLAS	2nd NPA missing
**psdo*SiNIP3;5***	Si015350m.g	Si015350m	scaffold_6: 5263741 - 5265487	220	*SiNIP3;1*(82)	VACU	Deletion of sequence before TM1
**psdo*SiNIP3;6***	Si002921m.g	Si002921m	scaffold_5: 6519473 - 6520404	210	*SiNIP1;1*(30)	PLAS	2nd NPA missing
**psdo*SiSIP1;2***	Si027934m.g	Si027934m	scaffold_8: 10259530 - 10260237	200	*SiSIP1;1*(72)	CHLO	Deletion of sequence after 2nd NPA motif
**psdo*SbPIP2;11***	Sobic.002G281000	Sobic.002G281000.1	Chr02: 66251292 - 66252975	326	*SbPIP2;9*(71)	PLAS	Isoform of (*SbPIP2;9*) max match
**psdo*SbPIP2;10***	Sobic.002G125500	Sobic.002G125500.1	Chr02: 16944617 - 16946309	362	*SbPIP2;4*(90)	MITO	Extension of N-terminal region
**iso*SbTIP1;3***	Sobic.003G445300	Sobic.003G445300.2	Chr03: 74338257 - 74340195	258	*SbTIP1;2*(100)	CYTO	Isoform of *SbTIP1;2*
**iso*SbTIP3;4***	Sobic.001G535900	Sobic.001G535900.1	Chr01: 72753512 - 72755158	280	*SbTIP3;3*(100)	CYTO	Isoform of *SbTIP3;3*

**psdo*SbTIP5;1***	Sobic.007G124600	Sobic.007G124600.1	Chr07: 52131594 - 52132649	202	*SbTIP2;2*(33)	CHLO	Deletion after 1st NPA motif
**psdo*SbTIP5;2***	Sobic.006G170500	Sobic.006G170500.1	Chr06: 53647415 - 53648493	222	*SbTIP2;2*(40)	PLAS	Deletion in N terminal/partial
**psdo*BdPIP1;3***	Bradi3g51387	Bradi3g51387.1	Bd3: 52443309 - 52446451	280	*BdPIP1;1*(88)	PLAS	2nd NPA absent
**psdo*BdPIP2;9***	Bradi1g28760	Bradi1g28760.2	Bd1: 24084808 - 24087780	265	*BdPIP2;3*(95)	PLAS	Isoform of *BdPIP2;1*
**psdo*BdNIP2;3***	Bradi3g59390	Bradi3g59390.2	Bd1: 24084808 - 24087780	211	*BdNIP2;1*(100)	PLAS	Isoform of *BdNIP2;1* & deletion in TM6/absent 2nd NPA
**psdo*BdNIP3;2***	Bradi4g03330	Bradi4g03330.1	Bd4: 2684363 - 2685050	187	*BdNIP3;1*(35)	CYTO	2nd NPA absent & deletion in TM6
**psdo*BdNIP3;3***	Bradi3g10880	Bradi3g10880.1	Bd3: 9129671 - 9130428	178	*BdNIP3;1*(38)	PLAS	2nd NPA absent and deletion in TM6
**Psdo*BdNIP4;2***	Bradi3g17100	Bradi3g17100.1	Bd3: 15213749 - 15215385	134	*BdNIP4;1*(28)	CYTO	deletion in N terminal
**psdo*BdSIP1;2***	Bradi1g64000	Bradi1g64000.1	Bd1: 63199737 - 63202053	194	*BdSIP1;1*(22)	CYTO	NPA absent & deletion in N terminal region
**psdo*BdSIP2;1***	Bradi1g35130	Bradi1g35130.1	Bd1: 30670879 - 30671412	177	*BdNIP4;1*(15)	CHLO	NPA absent & deletion in N terminal region

PPL: polypeptide length, aa: amino acid, SCL: subcellular localization, Plas: plasma membrane. Vacu: vacuolar membrane, Cyto: cytosol, ER: endoplasmic reticulum, Mito: mitochondrion, Nucl: Nucleous and Chlo: chloroplast.

[y]The same abbreviations as in Tables 1-4.
