# Supplementary material for: Genome-Wide Characterization of Major Intrinsic Proteins in Four Grass Plants and Their Non-Aqua Transport Selectivity Profiles with Comparative Perspective
Source: PLoS One. 2016 Jun 21;11(6):e0157735. doi: 10.1371/journal.pone.0157735 (PMC4915720; doi:10.1371/journal.pone.0157735)
Supplement: S2 Table — (PDF) [file pone.0157735.s013.pdf]

Table S2. Structurally important SIP group-specific amino acids and the role of residues in the corresponding positions in the structure of GlpF and AQP1 (or both)

Residues of GlpF[a]	Residues of AQP1[b]	Residues of SIP1s[b]	Residues of SIP2s[b]	Role in GlpF/AQP1[c]
E14	T	T	A/S	Stabilizing loop B
T18	V	V	V	Mid-membrane plane
G49	G	F/S	I/L	Mid-membrane plane
S63	S	G	G/K	Packing core near N68
G64	G	G	G	Buried, binds, and orients the vestibule
H66	H	S	S	Stabilizing loop B
A70	A	T	L	Packing core near N68
V71	V	G/T	T	Packing core near N68
T72	T	T	V/I	Packing core near N68
F89	F	F	F	Stabilizing loop B
Q93	M	L/V	V/A	Packing core near N68
G96	I	P	P	Mid-membrane plane
E152	E	E	E	Buried, binds, and orients the vestibule
T156	T	T	T	Mid-membrane plane
L159	L	I	V	Channel wall near G3
P180	P	T/V	T	Packaging helix 2 and 5
G184	G	A/S	S	Mid-membrane plane
I187	V	T	K	Hydrophobic lining
G199	G	G	G	Buried, binds, and orients the vestibule
F200	C	P	G	Hydrophobic corner
R206	R	N	S	Selectivity filter G2
D207	S	A	A/V	Packaging corner near N203
P210	S	W	W	Packaging corner near N203
P240	P	P	P	Packaging corner near N203
G243	G	G	A	Mid-membrane plane
**G191**	H	V	H	*
**S193**	L	S/A/V	L	[d]
**G195**	I	S/G/A	S	[d]
**P196**	D	A/K	D	[d]
**L216**	I	V/L	A	[d]
**A217**	T	N/S	R	[d]
**G218**	-	N/K	G	[d]
**W219**	-	W/R	D	[d]
**Y232**	N	Q	H	[d]
**L234**	W	Y	L	[d]

[a] Amino acid and positions in GlpF [35]. The bold residues in GlpF corresponds to conserved residues in H5, LE and H6 of SIP2s (Figure S13). [b] Corresponding amino acid residues in APQ1, SIP1s and SIP2s, from the multiple alignment (Figure S13), are shown for comparison. [c] Structural role in GlpF and/or AQP1[35,36] except bold residues in GlpF. [d] Residues may have SIP group-specific structural and/or functional roles. The star (*) indicates the residue at H5 position of the ar/R selectivity filter.
